# Supplementary material for: The effectiveness of smoking cessation, alcohol reduction, diet and physical activity interventions in changing behaviours during pregnancy: A systematic review of systematic reviews
Source: PLoS One. 2020 May 29;15(5):e0232774. doi: 10.1371/journal.pone.0232774 (PMC7259673; doi:10.1371/journal.pone.0232774)
Supplement: S2 Table — (DOCX) [file pone.0232774.s002.docx]

**S2 Table: Search terms**

| **Database: Medline <1974 to 26^th^ November 2019>** |
| --- |
| 1 exp Obesity/ |
| 2 obes*.tw. |
| 3 body mass.tw. |
| 4 exp Body Composition/ |
| 5 body composition.tw. |
| 6 exp Body Size/ |
| 7 body siz*.tw. |
| 8 bodysiz*.tw. |
| 9 exp Body Weight/ |
| 10 body weight.tw. |
| 11 fat.tw. |
| 12 fatness.tw. |
| 13 exp Overnutrition/ |
| 14 overnutrition.tw. |
| 15 exp Overweight/ |
| 16 overweight.tw. |
| 17 over weight.tw. |
| 18 weight.tw. |
| 19 exp Weight Gain/ |
| 20 weight gain.tw. |
| 21 weight maintenance.tw. |
| 22 weight management.tw. |
| 23 1 or 2 or 3 or 4 or 5 or 6 or 7 or 8 or 9 or 10 or 11 or 12 or 13 or 14 or 15 or 16 or 17 or 18 or 19 or 20 or 21 or 22 |
| 24 exp Fasting/ |
| 25 intermittent fast*.tw. |
| 26 alternate-day fast*.tw. |
| 27 intermittent energy restriction*.tw. |
| 28 intermittent calori* restriction*.tw. |
| 29 intermittent restrictive diet*.tw. |
| 30 continuous energy restriction*.tw. |
| 31 continuous calori* restriction*.tw. |
| 32 continuous restrictive diet*.tw. |
| 33 fasting calorie restriction intervention*.tw. |
| 34 very low calorie diet*.tw. |
| 35 periodic fasting*.tw. |
| 36 extreme diet*.tw. |
| 37 800* kcal.tw. |
| 38 500 calorie*.tw. |
| 39 sporadic fast*.tw. |
| 40 24 or 25 or 26 or 27 or 28 or 29 or 30 or 31 or 32 or 33 or 34 or 35 or 36 or 37 or 38 or 39 |
| 41 23 and 40 |
| 42 exp Adiposity/ |
| 43 exp Adipose Tissue/ |
| 44 (adverse adj (event* or inciden*)).tw. |
| 45 bio-impedance.tw. |
| 46 bioimpedance.tw. |
| 47 bioelectrical impedance analysis.tw. |
| 48 exp Blood Glucose/ |
| 49 blood glucose.tw. |
| 50 exp Blood Pressure/ |
| 51 blood pressure*.tw. |
| 52 exp Body Mass Index/ |
| 53 body mass index.tw. |
| 54 BMI.tw. |
| 55 bodpod.tw. |
| 56 exp Cholesterol/ |
| 57 cholesterol.tw. |
| 58 exp Diet/ |
| 59 diet.tw. |
| 60 exp Absorptiometry, Photon/ |
| 61 dexa scan*.tw. |
| 62 dxa.tw. |
| 63 exp Exercise/ |
| 64 exercise.tw. |
| 65 hydrostatic.tw. |
| 66 exp Magnetic Resonance Imaging/ |
| 67 magnetic resonance imag*.tw. |
| 68 MRI.tw. |
| 69 exp Skinfold Thickness/ |
| 70 skin-fold.tw. |
| 71 exp Waist Circumference/ |
| 72 waist circumference.tw. |
| 73 exp Weight Loss/ |
| 74 weight loss.tw. |
| 75 slim.tw. |
| 76 slimming.tw. |
| 77 thin.tw. |
| 78 thinness.tw. |
| 79 42 or 43 or 44 or 45 or 46 or 47 or 48 or 49 or 50 or 51 or 52 or 53 or 54 or 55 or 56 or 57 or 58 or 59 or 60 or 61 or 62 or 63 or 64 or 65 or 66 or 67 or 68 or 69 or 70 or 71 or 72 or 73 or 74 or 75 or 76 or 77 or 78 |
| 80 23 and 40 and 79 |
| 81 limit 80 to english language |
| 82 80 not 81 |
| 83 exp Randomized Controlled Trials as Topic/ |
| 84 exp Randomized Controlled Trial/ |
| 85 exp Random Allocation/ |
| 86 exp Double-Blind Method/ |
| 87 exp Single-Blind Method/ |
| 88 exp Clinical Trial/ |
| 89 clinical trial, phase i.pt. |
| 90 clinical trial, phase ii.pt. |
| 91 clinical trial, phase iii.pt. |
| 92 clinical trial, phase iv.pt. |
| 93 controlled clinical trial.pt. |
| 94 randomized controlled trial.pt. |
| 95 multicenter study.pt. |
| 96 clinical trial.pt. |
| 97 exp Clinical Trials as topic/ |
| 98 or/83-97 |
| 99 (clinical adj trial*).tw. |
| 100 ((singl* or doubl* or treb* or tripl*) adj (blind* or mask*)).tw. |
| 101 exp Placebos/ |
| 102 placebo$.tw. |
| 103 randomly allocated.tw. |
| 104 (allocated adj2 random$).tw. |
| 105 or/99-104 |
| 106 98 or 105 |
| 107 case report.tw. |
| 108 letter/ |
| 109 historical article/ |
| 110 or/107-109 |
| 111 106 not 110 |
| 112 81 and 111 |
|  |
| **Database: Embase <1974 to 26^th^ November 2019>** |
| 1 exp obesity/ |
| 2 obes*.tw. |
| 3 exp body mass/ |
| 4 body mass.tw. |
| 5 exp body composition/ |
| 6 body composition.tw. |
| 7 exp body size/ |
| 8 body siz*.tw. |
| 9 bodysiz*.tw. |
| 10 exp body weight/ |
| 11 body weight.tw. |
| 12 exp fat body/ |
| 13 fat.tw. |
| 14 fatness.tw. |
| 15 exp overnutrition/ |
| 16 overnutrition.tw. |
| 17 overweight.tw. |
| 18 over weight.tw. |
| 19 weight.tw. |
| 20 exp weight gain/ |
| 21 weight gain.tw. |
| 22 weight maintenance.tw. |
| 23 weight management.tw. |
| 24 1 or 2 or 3 or 4 or 5 or 6 or 7 or 8 or 9 or 10 or 11 or 12 or 13 or 14 or 15 or 16 or 17 or 18 or 19 or 20 or 21 or 22 or 23 |
| 25 exp diet restriction/ |
| 26 fasting.tw. |
| 27 intermittent fast*.tw. |
| 28 alternate-day fast*.tw. |
| 29 exp caloric restriction/ |
| 30 intermittent energy restriction*.tw. |
| 31 intermittent calori* restriction*.tw. |
| 32 intermittent restrictive diet*.tw. |
| 33 continuous energy restriction*.tw. |
| 34 continuous calori* restriction*.tw. |
| 35 continuous restrictive diet*.tw. |
| 36 fasting calorie restriction intervention*.tw. |
| 37 very low calorie diet*.tw. |
| 38 periodic fasting*.tw. |
| 39 extreme diet*.tw. |
| 40 800* kcal.tw. |
| 41 500 calorie*.tw. |
| 42 sporadic fast*.tw. |
| 43 25 or 26 or 27 or 28 or 29 or 30 or 31 or 32 or 33 or 34 or 35 or 36 or 37 or 38 or 39 or 40 or 41 or 42 |
| 44 24 and 43 |
| 45 adiposity.tw. |
| 46 exp adipose tissue/ |
| 47 (adverse adj (event* or inciden*)).tw. |
| 48 bio-impedance.tw. |
| 49 bioimpedance.tw. |
| 50 bioelectrical impedance analysis.tw. |
| 51 exp glucose blood level/ |
| 52 blood glucose.tw. |
| 53 exp blood pressure/ |
| 54 blood pressure*.tw. |
| 55 body mass index.tw. |
| 56 BMI.tw. |
| 57 bodpod.tw. |
| 58 exp cholesterol/ |
| 59 cholesterol.tw. |
| 60 exp diet/ |
| 61 diet.tw. |
| 62 exp photon absorptiometry/ |
| 63 exp dual energy X ray absorptiometry/ |
| 64 dexa scan*.tw. |
| 65 dxa.tw. |
| 66 exp exercise/ |
| 67 exercise.tw. |
| 68 hydrostatic.tw. |
| 69 exp nuclear magnetic resonance imaging/ |
| 70 magnetic resonance imag*.tw. |
| 71 MRI.tw. |
| 72 exp skinfold thickness/ |
| 73 skin-fold.tw. |
| 74 exp waist circumference/ |
| 75 waist circumference.tw. |
| 76 exp weight reduction/ |
| 77 weight loss.tw. |
| 78 slim.tw. |
| 79 slimming.tw. |
| 80 thin.tw. |
| 81 thinness.tw. |
| 82 45 or 46 or 47 or 48 or 49 or 50 or 51 or 52 or 53 or 54 or 55 or 56 or 57 or 58 or 59 or 60 or 61 or 62 or 63 or 64 or 65 or 66 or 67 or 68 or 69 or 70 or 71 or 72 or 73 or 74 or 75 or 76 or 77 or 78 or 79 or 80 or 81 |
| 83 24 and 43 and 82 |
| 84 limit 83 to english |
| 85 83 not 84 |
| 86 limit 83 to (conference abstract or conference paper or conference proceeding or "conference review") |
| 87 83 not 86 |
| 88 clinical trial/ |
| 89 randomized controlled trial/ |
| 90 exp randomization/ |
| 91 single blind procedure/ |
| 92 double blind procedure/ |
| 93 crossover procedure/ |
| 94 exp placebo/ |
| 95 randomi?ed controlled trial*.tw. |
| 96 RCT.tw. |
| 97 random allocation.tw. |
| 98 randomly allocated.tw. |
| 99 allocated randomly.tw. |
| 100 (allocated adj2 random).tw. |
| 101 single blind*.tw. |
| 102 double blind*.tw. |
| 103 (treble adj blind*).tw. |
| 104 (triple adj blind*).tw. |
| 105 placebo*.tw. |
| 106 exp prospective study/ |
| 107 or/88-106 |
| 108 exp case study/ |
| 109 case report.tw. |
| 110 abstract report/ or letter/ |
| 111 or/108-110 |
| 112 107 not 111 |
| 113 87 and 112 |
|  |
| **Database: CINAHL (Cumulative Index of Nursing and Allied Health Literature <1981 to 26^th^ November 2019>** |
| S1 (MH "Obesity+") |
| S2 TI obes* OR AB obes* |
| S3 TI body mass OR AB body mass |
| S4 (MH "Body Composition+") |
| S5 TI body composition OR AB body composition |
| S6 (MH "Body Size") |
| S7 TI body siz* OR AB body siz* |
| S8 TI bodysiz* OR AB bodysiz* |
| S9 (MH "Body Weight+") |
| S10 TI body weight OR AB body weight |
| S11 TI fat OR AB fat |
| S12 TI fatness OR AB fatness |
| S13 TI overnutrition OR AB overnutrition |
| S14 TI overweight OR AB overweight |
| S15 TI over weight OR AB over weight |
| S16 TI weight OR AB weight |
| S17 (MH "Weight Gain+") |
| S18 TI weight gain OR AB weight gain |
| S19 (MH "Weight Control") |
| S20 TI weight maintenance OR AB weight maintenance |
| S21 TI weight management OR AB weight management |
| S22 S1 OR S2 OR S3 OR S4 OR S5 OR S6 OR S7 OR S8 OR S9 OR S10 OR S11 OR S12 OR S13 OR S14 OR S15 OR S16 OR S17 OR S18 OR S19 OR S20 OR S21 |
| S23 (MH "Fasting") |
| S24 TI intermittent fast* OR AB intermittent fast* |
| S25 TI alternate-day fast* OR AB alternate-day fast* |
| S26 (MH "Restricted Diet+") |
| S27 TI intermittent energy restriction* OR AB intermittent energy restriction* |
| S28 TI intermittent calori* restriction* OR AB intermittent calori* restriction* |
| S29 TI intermittent restrictive diet* OR AB intermittent restrictive diet* |
| S30 TI continuous energy restriction* OR AB continuous energy restriction* |
| S31 TI continuous calori* restriction* OR AB continuous calori* restriction* |
| S32 TI continuous restrictive diet* OR AB continuous restrictive diet* |
| S33 TI fasting calorie restriction intervention* OR AB fasting calorie restriction intervention* |
| S34 TI very low calorie diet* OR AB very low calorie diet* |
| S35 TI periodic fasting* OR AB periodic fasting* |
| S36 TI extreme diet* OR AB extreme diet* |
| S37 TI 800* kcal OR AB 800* kcal |
| S38 TI 500 calorie* OR AB 500 calorie* |
| S39 TI sporadic fast* OR AB sporadic fast* |
| S40 S23 OR S24 OR S25 OR S26 OR S27 OR S28 OR S29 OR S30 OR S31 OR S32 OR S33 OR S34 OR S35 OR S36 OR S37 OR S38 OR S39 |
| S41 S22 AND S40 |
| S42 TI adiposity OR AB adiposity |
| S43 (MH "Adipose Tissue+") |
| S44 TI "adverse event*" OR AB "adverse event*" |
| S45 TI "adverse inciden*" OR AB "adverse inciden*" |
| S46 TI bio-impedance OR AB bio-impedance |
| S47 TI bioimpedance OR AB bioimpedance |
| S48 TI bioelectrical impedance analysis OR AB bioelectrical impedance analysis |
| S49 (MH "Blood Glucose") |
| S50 TI blood glucose OR AB blood glucose |
| S51 (MH "Blood Pressure+") |
| S52 TI blood pressure* OR AB blood pressure* |
| S53 (MH "Body Mass Index") |
| S54 TI "body mass index" OR AB "body mass index" |
| S55 TI BMI OR AB BMI |
| S56 TI bodpod OR AB bodpod |
| S57 (MH "Cholesterol+") |
| S58 TI cholesterol OR AB cholesterol |
| S59 (MH "Diet+") |
| S60 TI diet OR AB diet |
| S61 (MH "Absorptiometry, Photon") |
| S62 TI dexa scan* OR AB dexa scan* |
| S63 TI dxa OR AB dxa |
| S64 (MH "Exercise+") |
| S65 TI exercise OR AB exercise |
| S66 TI hydrostatic OR AB hydrostatic |
| S67 (MH "Magnetic Resonance Imaging+") |
| S68 TI magnetic resonance imag* OR AB magnetic resonance imag* |
| S69 TI MRI OR AB MRI |
| S70 (MH "Skinfold Thickness") |
| S71 TI skin-fold OR AB skin-fold |
| S72 (MH "Waist Circumference") |
| S73 TI waist circumference OR AB waist circumference |
| S74 (MH "Weight Loss+") |
| S75 TI weight loss OR AB weight loss |
| S76 TI slim OR AB slim |
| S77 TI slimming OR AB slimming |
| S78 TI thin OR AB thin |
| S79 TI thinness OR AB thinness |
| S80 S42 OR S43 OR S44 OR S45 OR S46 OR S47 OR S48 OR S49 OR S50 OR S51 OR S52 OR S53 OR S54 OR S55 OR S56 OR S57 OR S58 OR S59 OR S60 OR S61 OR S62 OR S63 OR S64 OR S65 OR S66 OR S67 OR S68 OR S69 OR S70 OR S71 OR S72 OR S73 OR S74 OR S75 OR S76 OR S77 OR S78 OR S79 |
| S81 S22 AND S40 AND S80 |
|  |
| **Database: Cochrane Library** Date Run: March 2018 |
| #1 MeSH descriptor: [Obesity] explode all trees |
| #2 obes*:ti,ab |
| #3 body mass:ti,ab |
| #4 MeSH descriptor: [Body Composition] explode all trees |
| #5 body composition:ti,ab |
| #6 MeSH descriptor: [Body Size] explode all trees |
| #7 body siz*:ti,ab |
| #8 bodysiz*:ti,ab |
| #9 MeSH descriptor: [Body Weight] explode all trees |
| #10 body weight:ti,ab |
| #11 fat:ti,ab |
| #12 fatness:ti,ab |
| #13 MeSH descriptor: [Overnutrition] explode all trees |
| #14 overnutrition:ti,ab |
| #15 MeSH descriptor: [Overweight] explode all trees |
| #16 overweight:ti,ab |
| #17 over weight:ti,ab |
| #18 weight:ti,ab |
| #19 MeSH descriptor: [Weight Gain] explode all trees |
| #20 weight gain:ti,ab |
| #21 weight maintenance:ti,ab |
| #22 weight management:ti,ab |
| #23 {or #1-#22} |
| #24 MeSH descriptor: [Fasting] explode all trees |
| #25 intermittent fast*:ti,ab |
| #26 alternate-day fast*:ti,ab |
| #27 intermittent energy restriction*:ti,ab |
| #28 intermittent calori* restriction*:ti,ab |
| #29 intermittent restrictive diet*:ti,ab |
| #30 continuous energy restriction*:ti,ab |
| #31 continuous calori* restriction*:ti,ab |
| #32 continuous restrictive diet*:ti,ab |
| #33 fasting calorie restriction intervention*:ti,ab |
| #34 very low calorie diet*:ti,ab |
| #35 periodic fasting*:ti,ab |
| #36 extreme diet*:ti,ab |
| #37 800* kcal:ti,ab |
| #38 500 calorie*:ti,ab |
| #39 sporadic fast*:ti,ab |
| #40 {or #24-#39} |
| #41 #23 and #40 |
| #42 MeSH descriptor: [Adiposity] explode all trees |
| #43 MeSH descriptor: [Adipose Tissue] explode all trees |
| #44 adverse event*:ti,ab |
| #45 adverse inciden*:ti,ab |
| #46 bio-impedance:ti,ab |
| #47 bioimpedance:ti,ab |
| #48 bioelectrical impedance analysis:ti,ab |
| #49 MeSH descriptor: [Blood Glucose] explode all trees |
| #50 blood glucose:ti,ab |
| #51 MeSH descriptor: [Blood Pressure] explode all trees |
| #52 blood pressure*:ti,ab |
| #53 MeSH descriptor: [Body Mass Index] explode all trees |
| #54 body mass index:ti,ab |
| #55 BMI:ti,ab |
| #56 bodpod:ti,ab |
| #57 MeSH descriptor: [Cholesterol] explode all trees |
| #58 cholesterol:ti,ab |
| #59 MeSH descriptor: [Diet] explode all trees |
| #60 diet:ti,ab |
| #61 MeSH descriptor: [Absorptiometry, Photon] explode all trees |
| #62 dexa scan*:ti,ab |
| #63 dxa:ti,ab |
| #64 MeSH descriptor: [Exercise] explode all trees |
| #65 exercise:ti,ab |
| #66 hydrostatic:ti,ab |
| #67 MeSH descriptor: [Magnetic Resonance Imaging] explode all trees |
| #68 magnetic resonance imag*:ti,ab |
| #69 MRI:ti,ab |
| #70 MeSH descriptor: [Skinfold Thickness] explode all trees |
| #71 skin-fold:ti,ab |
| #72 MeSH descriptor: [Waist Circumference] explode all trees |
| #73 waist circumference:ti,ab |
| #74 MeSH descriptor: [Weight Loss] explode all trees |
| #75 weight loss:ti,ab |
| #76 slim:ti,ab |
| #77 slimming:ti,ab |
| #78 thin:ti,ab |
| #79 thinness:ti,ab |
| #80 {or #42-#79} |
| #81 #23 and #40 and #80 |
