# Supplementary material for: The effectiveness of smoking cessation, alcohol reduction, diet and physical activity interventions in changing behaviours during pregnancy: A systematic review of systematic reviews
Source: PLoS One. 2020 May 29;15(5):e0232774. doi: 10.1371/journal.pone.0232774 (PMC7259673; doi:10.1371/journal.pone.0232774)
Supplement: S3 Table — (DOCX) [file pone.0232774.s003.docx]

**S3 Table: Screening tool based on the inclusion criteria of this Umbrella review**

| **Author and Year:** |
| --- |
| **Journal:** |
| **Title:** |
| **Name of reviewer:** |
| **Design:** This article is/ contains a systematic review or meta-analysis  **Yes / No** |
| **Language/Date:**  The review is published in English and was published since 2008  **Yes / No** |
| **Review type:**  The review is an effectiveness review considering quantitative evidence  For mixed methods reviews: the review contains quantitative evidence focusing on effectiveness  **Yes / No** |
| **Participants:** Does the review include **any** types of pregnant women (regardless of socio demographic factors; such as age, ethnicity, parity, socioeconomic status and so forth), and NOT focus solely on women in the preconception/ postnatal phases.  **Yes / No** |
| **Interventions:**  Interventions of interest to the review focus on one of the following target behaviours; weight management interventions**,** smoking cessation interventions, and alcohol reduction interventions (or to behaviours that relate to these target behaviours i.e. physical activity or diet)  **Yes / No** |
| **Outcomes:**  Does the review report on any of the following outcomes: effectiveness on behaviour change (explicit)^1^, or behaviour change (proxy), reach or usage of the intervention  **Yes / No** |
| **If you have not answered YES to all of the above questions, you should exclude the study. If you answered yes to all, please continue.** |

Footnote:

1. The screening form was developed for the wider programme of systematic reviews of systematic reviews; only systematic reviews of systematic reviews reporting on the effectiveness of interventions on explicit behaviour change were included for this paper.
