# Supplementary material for: The effectiveness of smoking cessation, alcohol reduction, diet and physical activity interventions in changing behaviours during pregnancy: A systematic review of systematic reviews
Source: PLoS One. 2020 May 29;15(5):e0232774. doi: 10.1371/journal.pone.0232774 (PMC7259673; doi:10.1371/journal.pone.0232774)
Supplement: S4 Table — (DOCX) [file pone.0232774.s004.docx]

**S4 Table: JBI Critical Appraisal Checklist for Systematic Reviews and Research Syntheses (Amended)**

| Reviewer ___________________________ | Date _______________________ | |
| --- | --- | --- |
| Author _____________________________ | Year _________ | Record number _____ |

|  |  | Yes | No | Unclear | Not applicable |
| --- | --- | --- | --- | --- | --- |
| 1. | Is the review question clearly and explicitly stated? | □ | □ | □ | □ |
| 2. | Were the inclusion criteria appropriate for the review question?  ***(Are all the PICO components clearly stated: Population, Intervention, Comparator, Outcome)*** | □ | □ | □ | □ |
| 3. | Was the search strategy appropriate?  ***(Search strategy/keywords must be stated)*** | □ | □ | □ | □ |
| 4. | Were the sources and resources used to search for studies adequate?  ***(At least 2 relevant databases should have been searched and at least one other data source [e.g. grey literature, reference list of included studies])*** | □ | □ | □ | □ |
| 5. | Were the criteria for appraising studies appropriate?  ***(Authors must have described an appropriate method for assessing quality of included studies, e.g. CASP tool)*** | □ | □ | □ | □ |
| 6. | Was critical appraisal conducted by two or more reviewers independently? | □ | □ | □ | □ |
| 7. | Were there methods to minimize errors in data extraction?  ***(Either all data extraction was performed in duplicate or data extraction with validation methods employed)*** | □ | □ | □ | □ |
| 8. | Were the methods used to combine studies appropriate?  ***(If meta-analysis performed did the authors justify their choice of method; if no meta-analysis is justification for this given?)*** | □ | □ | □ | □ |
| 9. | Was the likelihood of publication bias assessed?  ***(Evidence of statistical testing for publication bias given and/or funnel plot presented)*** | □ | □ | □ | □ |
| 10. | Were recommendations for policy and/or practice supported by the reported data? | □ | □ | □ | □ |
| 11. | Were the specific directives for new research appropriate? | □ | □ | □ | □ |
|  |  |  |  |  |  |

Note: Adaptations to the JBI critical appraisal checklist [1] were made after piloting its use and checking for inconsistencies between reviewers in their interpretation of the critical review question. The further detail was added to aid consistency in interpretation, and used some if the descriptors from the AMSTAR critical appraisal tool [2].

**References:**

1. The Joanna Briggs Institute. The Joanna Briggs Institute reviewers' manual 2014: methodology for JBI umbrella reviews. Adelaide, Australia: The Joanna Briggs Institute; 2014.

2. Shea BJ, Reeves BC, Wells G, Thuku M, Hamel C, Moran J, et al. AMSTAR 2: a critical appraisal tool for systematic reviews that include randomised or non-randomised studies of healthcare interventions, or both. BMJ (Clinical research ed). 2017;358:j4008.
