# Supplementary material for: The effectiveness of smoking cessation, alcohol reduction, diet and physical activity interventions in changing behaviours during pregnancy: A systematic review of systematic reviews
Source: PLoS One. 2020 May 29;15(5):e0232774. doi: 10.1371/journal.pone.0232774 (PMC7259673; doi:10.1371/journal.pone.0232774)
Supplement: S5 Table — (DOCX) [file pone.0232774.s005.docx]

**S5 Table: Description of included systematic reviews**

**S5a Table: Alcohol Reviews**

| **Alcohol Review** | **Aim of the review** | **Search strategy** | **Inclusion criteria** | **Exclusion criteria** | **Included studies** | **Study locations** |
| --- | --- | --- | --- | --- | --- | --- |
| Gilinsky  *et al.* 2011 [1] | To determine whether both randomised and non-RCTs of alcohol-reduction interventions resulted in reduced alcohol consumption or abstinence from alcohol during pregnancy. | Databases: Medline, Embase, PsycInfo, CINAHL, Cochrane Register for Controlled Trials and Systematic Reviews  Supplementary searches: None reported | - RCTs and non-RCTs testing an intervention delivered during pregnancy to reduce alcohol consumption. - Outcomes: short- and long-term maternal alcohol consumption during pregnancy, neonatal outcomes. - Studies that measured alcohol consumption in the postnatal period and/or subsequent pregnancies | - Unpublished studies - Studies not published in English. - Studies including women who used illicit drugs (unless alcohol outcomes were reported separately). - No identifiable alcohol consumption measure during pregnancy - Studies not considered potentially transferable to UK populations | - 10 studies (8 trials) - Published: 1982-2007 - Study design: RCT n=6; Non-RCTs n=2 - n=3380 women | USA n=5  UK n=2  Norway n=1 |
| Gebara  *et al.* 2013 [2] | To examine data from recent scientific publications on the use of Brief Interventions (BI) toward reducing alcohol consumption among women | Databases: Web of Science, PubMed (Medline) and PsycInfo.  Supplementary searches: None reported | - Studies performed and/or evaluated the effectiveness of a BI - Performed a BI toward alcohol consumption (not other drugs) - Presented women as part of the studied sample | - Non-English language | - 8 studies - Published: 2006-2011 - Study design: RCT n=5; Pilot n=1; Clinical trial n=1; Cohort n=1 - n=3494 (data not reported for 1 study) | USA n=7  Sweden n=1 |
| Lui  *et al.* 2008 [3] | To determine the effectiveness of psychosocial interventions in pregnant women who are enrolled in alcohol treatment programmes when compared to other psychosocial interventions, placebo, non-intervention, pharmacological treatment and pharmacological treatment in association with psychosocial treatment on improving birth and neonatal outcomes as well as maternal and neonatal alcohol abstinence and on treatment retention and alcohol reduction. | Databases: The Cochrane Library (Cochrane Drug and Alcohol Group trials register), MEDLINE, PsycINFO, EMBASE; CINAHL; SPECTR  Supplementary searches: hand searching, clinical trials register, personal contact, experts in the field, conference proceeding and personal communications. | - RCTs or quasi-methods of participant allocation. - Studies comparing psychosocial interventions versus either pharmacological intervention or placebo or no intervention or with another psychosocial intervention | - Not reported | - 0 studies | Not applicable |
| Stade  *et al.* 2009 [4] | To determine the effectiveness of psychological and educational interventions to reduce alcohol consumption during pregnancy in pregnant women or women planning pregnancy. | Databases: Cochrane Pregnancy and Childbirth Group’s Trials Register, CENTRAL, MEDLINE, EMBASE, CINAHL, Counsel.Lit, PsycLIT and PsycINFO  Supplementary searches: references from retrieved articles and reviewed abstracts. Letters to editors to identify unpublished RCTs. We contacted the primary investigator when further data were required | - Pregnant women or women planning pregnancy who consume alcohol - RCTs that compare the effectiveness of psychological and/or educational interventions for reducing prenatal consumption of alcohol - Studies where interventions have been compared with no intervention; with ’routine care’; or, different educational and/or psychological interventions have been compared - Psychological and/or educational interventions during pregnancy or 12 months before conception for women planning pregnancy. - Psychological interventions include cognitive behavioural therapy, brief psychodynamic psychotherapy, interpersonal psychotherapy and supportive counselling/therapy. - Educational interventions include brief educational counselling sessions, structured long-term educational programs with motivational enhancement interventions (greater than five sessions), individual focused educational strategies, family-focused programs, professional. | - Not reported | - 4 studies - Published: 1995 to 2007 - Study design: RCT n=4 - n=715 women | USA n=2  Mexico n=1  Unknown n=1 |

**S5b Table: Smoking Reviews**

| **Smoking Review** | **Aim of the review** | **Search strategy** | **Inclusion criteria** | **Exclusion criteria** | **Included studies** | **Study locations** |
| --- | --- | --- | --- | --- | --- | --- |
| Agboola  *et al.* 2010 [5] | To carry out a systematic review of the effectiveness of relapse prevention interventions among abstinent smokers who had completed an initial course of treatment or who had abstained unassisted, pooling only outcome data from similar time points. | Databases: Cochrane Tobacco Addiction Group register of trials; MEDLINE; Cochrane Central Register of Controlled Trials; EMBASE; PsycINFO; Science Citation Index; Social Science Citation Index  Supplementary searches: abstracts of the annual meeting of the Society for Research on Nicotine and Tobacco | - Randomized controlled trials - Participants had quit smoking on their own (unassisted abstainers), undergone enforced abstinence from smoking or had enrolled in smoking cessation treatment programmes (assisted abstainers) - Interventions intended to prevent relapse to smoking - Comparators either no intervention, a comparable intervention given for a shorter period of time or an intervention not orientated towards relapse prevention. - Trials with at least 6 months follow-up. | - Not reported | - 10 studies - Published: 1995-2008 - Study design: RCT n=10 - n=2550 women | USA n=9  UK n=1 |
| Chamberlain  *et al.* 2013 [6] | To assess the effects of smoking cessation interventions during pregnancy on smoking behaviour and perinatal health outcomes. | Databases: Cochrane Pregnancy and Childbirth Group’s Trials Register which contains trials identified from: monthly searches of the Cochrane Central Register of Controlled Trials; weekly searches of MEDLINE; weekly searches of Embase; hand searches of 30 journals and the proceedings of major conferences; weekly current awareness alerts for a further 44 journals plus monthly BioMed Central email alerts.  Supplementary searches: cited studies in the trial reports and key reviews, contacted trial authors to locate additional unpublished data. | - Randomised controlled trials, cluster-randomised controlled trials, and randomised cross-over trials - Quasi-randomised studies if there was a very low risk of interference with the sequence generation - Psychosocial interventions for smoking cessation in pregnancy. - Women currently smoking or recently quit smoking, in any care setting. - Trials of implementation strategies of psychosocial interventions to support pregnant women to stop smoking. - Interventions that offered pharmacological therapies as part of a tailored intervention where there were higher levels of psychosocial support provided - Smokeless tobacco use, environmental tobacco smoke exposure or partner smoking in conjunction with interventions addressing the aim of supporting pregnant women to stop smoking. - Dissemination studies including strategies to disseminate smoking cessation interventions in pregnancy care settings | - Studies with infant outcome measures such as birthweight, preterm birth, breastfeeding and perinatal mortality - Trials where the sole aim was to reduce: smokeless tobacco use; environmental tobacco smoke exposure - The primary population was not pregnant women - The intervention was not primarily aimed at cessation during pregnancy | - 86 studies - Published: - 1976 – 2012 - Study design: RCTs n=86 - n= >29,000 women | USA n=57  Canada n=1  UK n=13  Norway n=3  Sweden n=1  Holland n=1  Spain n=1  Australia n=5  New Zealand n=2 |
| Filion  *et al.* 2011 [7] | To conduct a meta-analysis of RCTs examining counselling in pregnant smokers. | Databases: CDC Tobacco Information and Prevention, Cochrane Library, EMBASE, Medline and PsycINFO  Supplementary searches: references of published RCTs, relevant reviews and previous meta-analyses. | - RCTs in pregnant smokers - Efficacy of smoking cessation counselling, including minimal clinical intervention, individual counselling, group counselling or telephone counselling - Biochemically validated point prevalence or continuous smoking abstinence at 6 or 12 months follow up - Published in English. | - Randomised physicians, therapists, or centres rather than women - RCTs that examined self-help or educational interventions other than counselling - RCTs that did not include a usual care control group. - RCTs that included multicomponent interventions or that had co-interventions if the co- intervention was not used in both treatment groups. | - 8 studies - Published: 1994-2009 - Study design: RCT n=8 - n=3290 women | USA n=6  UK n=2 |
| Hemsing  *et al.* 2012 [8] | Do interventions that involve partners ’ support of their pregnant partners lead to effective smoking cessation among pregnant partners during pregnancy and postpartum? Are there interventions that are effective in encouraging partners who smoke to stop smoking? | Databases: CINAHL, EMBASE, MEDLINE, PsycINFO , and NHS EED  Supplementary searches: None reported | - Interventions which examined the impact of partner support or partner smoking on smoking cessation among pregnant women and/or the partners | - Non-English language | - 4 studies - Published: 1994-2006 - Study design: RCT n=2; CRCT=1; Pre-post-test design n=1 - N participants not reported | USA n=1  UK n=2  Netherlands n=1 |
| Hettema  *et al.* 2010 [9] | To investigate the efficacy of motivational interviewing (MI) for smoking cessation | Databases: PubMed and PsycINFO  Supplementary searches: Bibliographies of included studies and previously conducted meta-analyses. | - Studies examining at least one intervention condition that included MI - Examine at least one comparison condition that did not include MI - Indicate use of a procedure to ensure the equivalence of groups - Report an abstinence-related outcome measure. | - Not reported | - 8 studies - Published: 1999-2008 - Study designs not reported - n=2381 | Not reported |
| Kintz  *et al.* 2014 [10] | To describe the degree of variability in the methodological approaches and theoretical frameworks of behavioral intervention for smoking cessation during pregnancy. | Databases: CINAHL and MEDLINE  Supplementary searches: bibliographies of related studies | - No definitive inclusion/exclusion, key words were used: pregnancy, smoking, behavioural modification, interventions, cessation and program | - Not reported | - 24 studies - Published: 2001-2011 - Study designs not reported - n=31958 women | Not reported |
| Naughton  *et al.* 2008 [11] | To assess the efficacy of self-help interventions for pregnant smokers and to investigate whether self-help material intensity, type or delivery  are associated with cessation | Databases: MEDLINE, EMBASE, PsycINFO, CENTRAL  Supplementary searches: Bibliographies of relevant reviews and published studies were hand-searched | - Controlled trials with a randomized or quasi-randomized allocation - Including pregnant smokers at any stage of care - Aged 16 years and over | - The sample consisted only of pregnant quitters or those with a history of substance misuse. | - 15 studies - Published: 1985 - 2005 - Study design: not reported - n=4721 included in primary meta-analysis (12 studies) - n=1487 included in secondary meta-analysis (7 studies) | USA n=10  UK n=2  Sweden n=1  Norway n=1  Australia n=1 |
| Su  *et al.* 2014 [12] | To investigate the prevention of postpartum smoking relapse for nonspontaneous quitters. | Databases: PubMed, the Cochrane Library, and CINAHL  Supplementary searches: Hand searches of bibliographies | - Smoking cessation interventions that were begun during pregnancy and that continued following participants for at least 1 month postpartum. - Clinical trial - English language - Randomized control trial | - Studies that: were qualitative or descriptive, - Were still in trial - Did not include an intervention - Began postpartum only - Did not follow participants past 1 month postpartum | - 32 studies - Published: 1992-2012 - Study design: RCT n= 27; Non-RCT n= 5 - n=24,095 women | Not reported |
| Washio  *et al.* 2016 [13] | To systematically review controlled studies of smoking cessation during pregnancy that predominantly included minority women and suggest ways to expand studies on minority pregnant smokers and improve smoking cessation rates based on the findings. | Databases: PubMed, MEDLINE, PsycINFO, and EBSCOhost  Supplementary searches: none reported | - Randomized and quasi-randomized controlled studies - Published in English and in peer-reviewed journals - Studies that aimed at smoking cessation during pregnancy. - Studies that treated pregnant women who were smoking at the time of entering the study - Studies that included minority pregnant smokers as more than fifty percent of the participants - Studies that included minority pregnant smokers in the U.S. - Studies that reported smoking cessation outcomes, including: biochemically-verified smoking abstinence with breath, saliva, or urine samples and/or self-reported smoking abstinence. | - Not reported | - 9 studies - Published: 1993-2012 - Study design: RCT n=8; Non-RCT n=1 - n=1690 women | USA n=9 |
| Chamberlain *et al.* 2017 [14] | To assess the effects of smoking cessation interventions during pregnancy on smoking behaviour and perinatal health outcomes. | Databases:  Cochrane Pregnancy and Childbirth’s Trials Register  Supplementary Searches:  Checked cited studies while reviewing the trial reports and key reviews. We contacted the authors of any published protocols or ’ongoing studies’ to ask if studies have been finalised. Where necessary, we contacted trial authors to locate additional unpublished data. | - All individually-randomised controlled trials, cluster-randomised controlled trials, and randomised cross-over trials - Psychosocial interventions where a primary aim of the study was smoking cessation in pregnancy. - Quasi-randomised studies were only considered for inclusion if there was a very low risk of interference with the sequence generation (e.g. allocation by odd or even maternal or infant birth date or hospital record number). | - None reported | - 88 studies - Published: 1976-2015 - Study design: RCT n=88 - n>26,000 women | USA n=68  UK n=18  Europe n=11 (Norway, Holland, Netherlands, Sweden, Spain, Poland)  Australia/ New Zealand n=9  South America n=1  Canada n=2  Greece n=1  Multiple countries n=1 (Argentina,  Brazil, Cuba and Mexico) |
| Griffiths *et al.* 2018 [15] | 1.Are digital interventions more effective in increasing smoking cessation rates in pregnancy than  usual care/other control groups?  2. Is the platform of delivery of digital interventions associated with smoking cessation in pregnancy?  3. Which BCTs/combinations of BCTs, when included in digital interventions, are associated with?  4. Are the number of BCTs used in digital interventions associated with smoking cessation in pregnancy?  smoking cessation in pregnancy? | Databases:  Academic Search Complete; ASSIA, CINAHL; Cochrane library; EMBASE; Medline: PsycINFO; Scopus and Web of Science  Supplementary searches: Research registers of NIHR UK Clinical Trials Gateway; ClinicalTrials.gov and Current Controlled trials through ISRCTN were searched. Lead investigators were contacted where necessary to ask whether trial results were available or near completion. Reference lists of screened studies meeting the inclusion criteria and relevant published reviews were searched by hand. Reference lists of papers citing included studies were also examined. | - Randomised and quasi-randomised controlled trials were included. Articles were included if they were written in English. - No restrictions on publication date were applied in the initial search in September 2016. For the updated search carried out in May 2017, parameters were added to include research from 2016 to 2017 only. - Participants were women at any stage of pregnancy, reporting to be current cigarette smokers. - digital interventions included any intervention delivered largely through a computer (PC or laptop), video or DVD, mobile telephone or portable handheld device (e.g., tablet or iPad). This included email, video, DVDs, websites or web-based games, mobile or tablet applications and SMS text messages or MMS multimedia messages. - Standard usual care for smoking cessation in pregnancy typically consists of brief cessation advice delivered by a healthcare professional. - Trials using the same method for the comparison group, e.g., usual care, were pooled into a subgroup meta-analysis. Trials with more than one comparator arm were included only if at least one of the experimental arms met the inclusion criteria for a digital intervention, - Only trials reporting smoking abstinence were included. | - Studies with only ex-smokers or post-natal participants. | - 12 studies - Published: 1991 to 2017 - Study design: RCT and quasi-RCT (numbers not reported) - n=2306 women | USA n=8  UK n=4 |
| Hand *et al.* 2017 [16] | How CM has been used among pregnant women with substance use disorders, including its effectiveness in reducing maternal substance use, increasing treatment attendance, and improving maternal and child health | Databases: PsycINFO, web of Science and PubMed.  Supplementary searches: All available reviews and cited references were examined to ensure that all relevant studies were extracted based on the search strategies | - The target population was pregnant or perinatal women with current substance use disorders; - The study included or reported contingency management outcomes; - The study included an independent sample for whom results were not previously published elsewhere; and - Reinforcers were provided for abstinence or treatment attendance. | - Non peer reviewed studies | - 27 studies - Published: 1992 to 2015 - Study design: RCTs n=17, non-RCTS n=7, cohort studies n=3 - n= 2,827 women | Not reported |
| Heminger *et al.* 2016 [17] | To summarize mHealth programs (text messaging and app-based interventions) currently available for pregnant smokers and, where available, to summarize study results and outcomes. | Databases: PubMed, PsychINFO and Medline  Supplementary searches: The research team conducted a scan of the iTunes and GooglePlay app stores to identify mobile apps related to pregnancy and smoking cessation. The research team also performed gray literature searches using Google; | - Include a mHealth element (text messaging or mobile app) explicitly designed to promote smoking cessation among women during and/or after pregnancy - Report on at least one smoking cessation outcome. - Cessation programs aimed at a larger target audience (ie, “adults”) but with an explicit pregnancy component (ie, topical listing or menu for pregnant women or ability to tailor results based on pregnant status) were also included - There were neither date nor language exclusions set. | - General text messaging programs and applications that made no mention of pregnancy. | - 7 studies - Published: 2012-2015 - Study design: RCT n=3, other design n=4 - n=1,009 women when reported, plus two studies reporting their study sample as “10,000-50,000 downloads” and “100-500 downloads” | USA n=2  UK n=2  Australia n=1  Canada n=2 |
| Veisani *et al.* 2017 [18] | To obtain a pooled estimate the effect of prenatal smoking cessation on birth weight. (To assess the effects of smoking cessation in pregnancy period on the birth weight in Randomized Controlled Trial studies (RCTs). | Databases:.  Pub Med, Scopus, and Web of Science  Supplementary searches: cross-referring publications | - RCTs - Assess the prenatal smoking cessation intervention - Effects on birth weight | - Duplicate articles - Poor-quality articles | - 16 studies - Published: 1999 to 2016 - Study design: RCTs n=16 - n=6,192 women | UK n=4  USA n=7  France n=1  Poland n=1  Denmark n=1  Netherlands n==1  Australia n=1 |
| Hubbard *et al.* 2016 [19] | To identify, describe, and synthesise the evidence about family-based interventions for smoking cessation. | Databases: Cochrane Library, Campbell Library, EBSCO HOST (CINAHL, PsycINFO, Psychology and behavioural sciences collection, EconLit), Ovid Medline, Ovid HMIC, Ovid Embase, ProQuest (Applied Social Sciences Index and Abstracts, Social Services Abstracts, Sociological Abstracts, Australian Education Index, British Education Index, Education Resources Information Center), Prospero, PubMed, SCOPUS, Web of Science (Science Citations Index, Social Sciences Citation Index, Arts and Humanities Citation Index, Conference Proceedings Citation Index-Science, Conference Proceedings Citation Index- Social Science & Humanities, Book Citation Index-Science, Book Citation Index – Social Science and Humanities)  Supplementary searches: None reported | - Trials of family-based interventions - Targeting smoking in adults - Published in English language - No date restriction | - Not in English language | - 3 studies (targeting pregnant women) - Published: 2004-2008 - Study design: RCT n=1, cluster RCT n=1, cohort n=1 - n=5,323 women | USA n=1  Netherlands n=1  Norway n=1 |
| Wilson *et al.* 2018 [20] | To compare the effect sizes of two intensive interventions for prenatal smoking cessation: contingency management (i.e., financial incentives for abstinence) and psychotherapy | Databases: PubMed, PsycINFO, Web of Science, the Cochrane Library, EMBASE  Supplementary searches: bibliographies of included trials and applicable systematic reviews | - Women who were pregnant - RCTs - Biochemically verified smoking status at a follow-up of at least 3 months post-randomization - Point prevalence smoking abstinence reported - Active treatment compared to standard of care, treatment as usual, best practice treatment, or another less-intensive treatment (e.g., non-contingent incentives) | - Non-experimental design - Both groups received financial incentives for abstinence | - 22 studies - Published: 1994-2016 - Study design: RCT n=22 - n=6,581 women | Not reported |

**S5c Table: Diet and/or Physical Activity Reviews**

| **Diet/Physical Activity Review** | **Aim of the review** | **Search strategy** | **Inclusion criteria** | **Exclusion criteria** | **Included studies** | **Study locations** |
| --- | --- | --- | --- | --- | --- | --- |
| Bain  *et al.* 2015 [21] | To assess the effects of dietary interventions in combination with physical exercise interventions for pregnant women for preventing gestational diabetes mellitus, and associated adverse health consequences for the mother and her infant/child. | Databases: Cochrane Central Register of Controlled Trials (CENTRAL); MEDLINE; Embase; CINAHL  Supplementary searches: reference lists of retrieved studies, hand searches of 30 journals and the proceedings of major conferences; alerts for a further 44 journals, monthly BioMed Central alerts | - Pregnant women regardless of age, gestation, parity or plurality. | - Studies involving women with pre-existing type I or type II diabetes. | - 13 studies (39 records) - Published: 2002-2014 - Study design: RCT n=11; Cluster RCT n=2 - n=4983 women | USA n=3  Finland n=2  Australia n=2  UK n=1  Canada n=1  Italy n=1  Germany n=1  Denmark n=1  Egypt n=1 |
| Brown  *et al.* 2012 [22] | To explore the use of goal setting within healthy lifestyle interventions for the prevention of excess gestational weight gain (GWG). | Databases: Medline, Embase, British Nursing Index, CINHAL, Cochrane-Central Library, PubMed-National Library of Medicine and PsycINFO.  Supplementary searches: Hand- searches of relevant journals and reference lists along with citation tracking | - Healthy pregnant women ≥18 years old. - Interventions using goal setting alongside modification to diet and physical activity/exercise levels with an aim to prevent excess GWG. - Randomised controlled trials of strong or moderate methodological quality | - Only abstracts available - Studies aimed at modifying diet and/or exercise during pregnancy for the primary purpose of improving or managing a specific disease (e.g. gestational diabetes) - Studies that focused on teenage mothers | - 5 studies - Published: 2002-2011 - Study design: RCT n=5 - n=860 women | Not reported |
| Flynn  *et al.* 2016 [23] | To evaluate the adequacy and effectiveness of the methodological designs implemented in dietary intervention trials for obesity in pregnancy. | Databases: Medline, EMBASE, BIOSIS, LILACS, Pascal, Science Citation Index, Cochrane Database of Systematic Reviews, Cochrane Central Register of Controlled Trials, DARE, and HTA, Inside Conferences Systems for Information in Grey Literature, dissertation abstracts, and clinicaltrials.gov  Supplementary searches: Internet searches including OMNI Medical Search Google, and Copernic. | - Randomized controlled trial that evaluated dietary and/or lifestyle interventions in pregnancy compared with standard ante- natal care - Participants who were overweight (BMI 25 kg/m^2^ or 23 kg/m^2^ if high risk ethnicity) or obese (BMI 30kg/m^2^); - Defined dietary intervention implemented as part of intervention(s) that were based on diet or a mixed approach comprising diet and physical activity components - Data reporting outcomes for the mother and their infants. | - Nonrandomized and observational studies - Participants aged less than 18 years - Multiple pregnancies - Participants with a normal/healthy BMI (<25 kg/m^2^ or ethnic-specific cut-offs); - Studies designed to treat gestational diabetes mellitus - Studies in which antenatal advice or the intervention focused solely on physical activity - Studies not reported in English. | - 13 studies - Published: 2008-2015 - Study design: RCT n= 13 - n=4276 women | USA n=3  Belgium n =2  Australia n=3  Italy n=1  UK n=1  Denmark n=3 |
| Gardner  *et al.* 2011 [24] | To meta-analyse behaviour based interventions that have targeted diet and/or physical activity (PA) changes to reduce gestational weight gain (GWG), and explore moderators of intervention effectiveness | Databases: PsycInfo, Medline, Embase, AMED, HMIC, Cochrane Central Controlled Trials Register, Cochrane Health Technology Assessment  Supplementary searches: All corresponding authors were contacted and asked to provide further written description of the intervention content. | - Studies reported an evaluation, based on quantitative data - Efficacy of an intervention to improve diet and/or increase PA so as to prevent excessive weight gain - Pregnant women aged 18+ years - Differences between an intervention and a control group on self-reported or objective behaviour or weight gain, measured prior to delivery, were reported | - Interventions based on information provision only, or non-psychological interventions (i.e. medical or nutritional interventions) - Studies where participants had known pre-pregnancy mental or physical health problems | - 12 trials of 11 interventions reported in 10 papers - Published: 2000-2010 - Study design: RCT n=5; Non RCT n= 2; Time series control trial n=1; Historical cohort n=2 - n= 1656 women | USA n=4  Canada n=3  Denmark n=1  Sweden n=1  Belgium n=1 |
| Webb-Girard *et al.* 2011 [25] | The effect of Nutrition Education Counselling (NEC) on maternal, neonatal and infant health outcomes including gestational weight gain, maternal anaemia, birthweight, low birthweight and preterm delivery | Databases: PubMed, Popline, Web of Science, CINAHL and EMBASE  Supplementary searches: Journals (Journal of Nutrition, Social Science and Medicine, American Journal of Clinical Nutrition, African Journal of Food Agriculture and Nutrition, Public Health Nutrition, Nutrition Journal, Food and Nutrition Bulletin) and hand-searched references from published articles and reviews. | - Experimental studies utilising a control group - Randomised controlled trials, cluster RCTs and quasi-experimental interventions. - Delivered to pregnant adults or adolescents - The NEC intervention focused on improving maternal diet and nutritional status - The comparison group was a concurrent control or comparison group that did not receive NEC. In the event that both groups received NEC, the system of delivery, number of sessions and/or intensity differed. | - Studies targeting pregnant women with rare or congenital diseases (i.e. inborn errors of metabolism) or those with pre-existing chronic conditions (i.e. obesity, diabetes, rheumatoid arthritis). - Studies utilising historical controls. - Studies prescribing special diets (i.e. reduced sodium, low glycaemic index) or that focused on improving infant-feeding practices. - Studies in a language other than English. | - 34 studies - Published: 1952-2012 - Study design: RCT n=16; Quasi-Experimental n=6; Cluster quasi-experimental n=4; Cluster RCT n=2 - N participants not reported | Not reported |
| Mohd Yusof  *et al.* 2014 [26] | To evaluate the effectiveness of low GI dietary intervention for the treatment of GDM specifically from the Asian perspective. | Databases: MEDLINE, SCOPUS, ISI Web of Science and Google scholar  Supplementary searches: Review of references of included studies | - Women with a diagnosis of GDM or impaired glucose tolerance of pregnancy - Clinical trials (controlled and randomised controlled), prospective observational studies, cross-sectional observational studies or case control studies. | - Healthy pregnant women - Women at risk of gestational diabetes - Animal models | - 3 studies - Published: 2009 - 2011 - Study design: RCT n=3 - n=209 women | Australia n=2 Canada n=1 |
| Muktabhant  *et al.* 2015 [27] | To determine whether diet or exercise measures, or both, could prevent excessive gestational weight gain, and if they were safe | Databases: Cochrane Pregnancy and Childbirth Group’s Trials Register  (includes CENTRAL, MEDLINE, Embase)  Supplementary searches: Hand searched 30 journals and conference proceedings; weekly current awareness alerts for a further 44 journals; monthly BioMed Central email alerts; contacted investigators of the previously identified ongoing studies by email to enquire about any new or imminent publications.  Reference lists of retrieved studies | - Pregnant women of any BMI - Randomised controlled trials | - Not reported | - 65 studies - Published: 1990-2014 - Study design: RCT n=65 - n=>13, 523 women (2 studies did not report number of participants) - 49 RCTs (n=11,444 women) included in meta-analysis | Australia n=10 Belgium n=2 Canada n=5 Denmark n=3 Finland n=3 Germany n=1 Ireland n=1  Italy n=3  Norway n=1 Sweden n=2 Netherlands n=2  Spain n=3  UK n=1  USA n=20  Brazil n=4  Columbia n=1  Taiwan n=1 |
| Nasciment  *et al.* 2012 [28] | To provide an update on the latest evidence concerning exercise during pregnancy including effects for mother and fetus and the type, frequency, intensity, duration and rate of progression of exercise performed. | Databases: PubMed, ISI Web of Knowledge  Supplementary searches: Reference lists of identified studies | - Randomised controlled trials - Conducted in healthy pregnant women - Any form of physical exercise program - Maternal or fetal outcomes being assessed, - Published July 2010 to July 2012 - Published in English | - Not reported | - 19 studies - Published: 2010 - 2012 - Study design: RCT n=19 - n=4441 women | Not reported |
| O’Brien  *et al.* 2014 [29] | To systematically review the literature examining technology-supported lifestyle interventions among healthy pregnant women, and to determine the potential impact that such interventions could have on modern antenatal care | Databases: PubMed, MEDLINE, CINAHL, EMBASE, Cochrane Database, Online trial registries (www. controlled-trials.com and www.clinicaltrials.gov)  Supplementary searches: Journals (American Journal of Obstetrics and Gynaecology; British Journal of Obstetrics and Gynaecology). References of included manuscripts searched. | - English language articles - Healthy pregnant women - RCTs, cross-sectional observational studies, feasibility studies and ongoing trials were included | - Diagnosed with GDM prior to intervention. | - 7 studies - Published: 2009 - 2013 - Study design: RCT n=5, observational n=2 - n=4,500 women (RCTs) - n=15,328 women (observational) | USA n=4  Netherlands n=2  Australia n=1 |
| Lau *et al.* 2017 [30] | “The purpose of this review is to synthesize the best evidence to assess the effectiveness of e-based lifestyle interventions in improving maternal and neonatal outcomes among perinatal overweight or obese women.” | Databases: CINAHL, Cochrane Library, EMBASE, ProQuest Dissertations and Theses, PsycINFO, PubMed and Scopus  Supplementary searches: search for  ongoing trials in the clinical trial registries and hand search of the reference lists of the eligible studies and systematic reviews of the topic for additional studies | - Overweight (BMI ≥ 25 kg m2) and/or obese (BMI ≥ 30 kg m2) women during the perinatal period (starting from pregnancy to 1 year postpartum - E-based lifestyle interventions comprising at least one component of dietary control, physical activity and weight management that were delivered through at least one of the following means: website, Internet, Apps, SMS, email, computer or video player - As a comparator a minimal intervention or usual care was considered - Primary outcomes included GWG, postnatal weight change, moderate and vigorous physical activity (MVPA) and calorie intake. In addition, the secondary outcomes included activity-related outcomes (exercise or walking), diet-related outcomes (fruit and vegetable or saturated fat intake), obstetric complications (antenatal depression, gestational diabetes, preeclampsia or caesarean delivery) and neonatal outcomes (birth weight, gestational age at birth or macrosomia). | - Studies on overweightness or obesity among the general population or pregnant women with comorbidities. - Studies that had no lifestyle components or e-based elements in the intervention group. - Studies which used clinical controlled trials, non-experimental methods, utilized qualitative designs, contained only abstracts or that were study protocols, reviews or conference papers. | - 14 studies - Published: 2006 - 2016 - Study design: RCT n=14 - n=3,169 women | USA n=9  Australia n=2  Sweden n=1  UK n=1  Canada n=1 |
| Shepherd *et al* 2017 [31] | To assess the effects of diet interventions in combination with exercise interventions for pregnant women for preventing gestational diabetes mellitus (GDM), and associated adverse health consequences for the mother and her infant/child. | Databases: We searched Cochrane Pregnancy and Childbirth’s Trials Register by contacting their Information Specialist. For full search methods used to populate Pregnancy and Childbirth’s Trials Register including the detailed search strategies for CENTRAL, MED- LINE, Embase and CINAHL.  Supplementary searches: The list of hand searched journals and conference proceedings, and the list of journals reviewed via the current awareness service,  We searched the reference lists of retrieved trials. | - Studies: all published randomised controlled trials assessing the effects of combined diet and exercise interventions for pre- venting gestational diabetes mellitus (GDM). We included cluster-randomised trials, and trials published as abstracts only - Participants: trials of pregnant women regardless of age, gestation, parity or plurality. - Intervention: interventions that incorporated any type of diet intervention with any type of exercise intervention. We included trials where such interventions were compared with no intervention (i.e. standard care), and planned to include where they were compared with a different diet and exercise intervention. - Outcome: standard outcomes agreed by consensus between review authors of Cochrane Pregnancy and Childbirth systematic reviews for prevention and treatment of GDM and pre-existing diabetes. | - Quasi-randomised controlled trials. Cross-over trials were not eligible for inclusion. - Trials involving women with pre- existing GDM, type 1 or type 2 diabetes. | - 23 studies - Published: 2002 - 2017 - Study design: cluster RCT n=2, Individually RCT n=21 - n=8918 women | USA n=5  Finland n=3  Australia n=2  UK n=2  Canada n=2  Italy n=2  China n=2  Brazil n=1  Denmark n=1  Egypt n=1  Germany n=1  Norway n=1 |
| Sherifali *et al* 2017 [32] | The primary objective of this systematic review was to assess the effectiveness of eHealth technologies for managing weight (loss, gain, or maintenance) during pregnancy and the postpartum period. The secondary objectives were to assess the effectiveness of eHealth technologies on other clinical outcomes, including (1) glycemic parameters and (2) health behaviors (ie, nutrition and physical activity). | Databases:  MEDLINE, EMBASE, Cochrane database of systematic reviews (CDSR), Cochrane central, register of controlled trials (CENTRAL), CINAHL (Cumulative Index to Nursing and Allied Health Literature), and PsycINFO.  Supplementary searches: Reference lists and associated paper citations were reviewed to identify other potential eligible papers that may have been missed during the initial search. | - Study design: All relevant primary studies that involved randomized controlled trials (RCTs), non-RCTs such as clinical controlled trials (CCTs), pre-post studies, historically controlled studies, and pilot studies were included. - Population: adult women of childbearing age (≥18 years) either during pregnancy or the postpartum period. - Intervention: The intervention must have included a health behavior component (nutrition or physical activity) in the eHealth technology, with a specific goal of targeting either GWG during pregnancy or weight loss during the postpartum period . Eligible eHealth technologies included the following: mobile phone (text-messaging or short message service [SMS] or mobile phone app), Web-based, email, personal digital assistant, handheld computer, home computer, or tablet app. A minimum intervention duration of 3 months was required. - Comparator: Three different reference groups were considered as comparators: (1) in-person interventions, (2) other health technology interventions, and (3) no intervention (ie, standard care or usual health care environment). - Outcomes: The primary outcome was weight management with specific targets of GWG, measured in kilograms (kg) in pregnant women or weight loss (measured in kg) in postpartum women. In both populations, we also investigated changes in glycemic status (eg, fasting and 2-hour glucose levels), nutritional measures (eg, total energy intake), and physical activity (eg, minutes of physical activity). | - All study protocols without preliminary results for data extraction were excluded. - All other study designs not part of the inclusion criteria were excluded. | - 6 studies - Published: 2010-2016 - Study design: RCTs n=5, CCTs n=1 - n=363 women (only including interventions during pregnancy) | UK n= 1  Spain n=2  USA n= 3  (only including interventions during pregnancy) |
| Tieu *et al* 2017 [33] | To assess the effects of dietary advice interventions for preventing GDM and associated adverse health outcomes for women and their babies. | Databases:  Cochrane Pregnancy and Childbirth’s Trials Register  CENTRAL, MEDLINE, Embase and CINAHL  Supplementary searches:  Hand searched journals and conference proceedings, journals reviewed via the current awareness service. Reference lists of retrieved articles | - randomised and quasi-randomised controlled trials involving pregnant women - interventions that assessed any type of dietary advice before testing for GDM - studies involving pregnant women regardless of age, gestation, parity or plurality. - core outcome set agreed by consensus between review authors of Cochrane Pregnancy and Childbirth systematic reviews for prevention and treatment of gestational diabetes mellitus (GDM) and pre-existing diabetes | - excluded cross-over trials - trials presented only as abstracts - studies involving women with pre-existing type 1 or type 2 diabetes | - 11 studies - Published: 1983 to 2016 - Study design: RCTs n=11 - n=2786 women | USA n=2  UK n=1  Finland n=1  Australia n=4  Brazil n=1  Ireland n=1  Denmark n=1 |
| Currie *et al* 2013 [34] | To evaluate the content of PA interventions to reduce the decline of PA in pregnant women with a specific emphasis on the behaviour change techniques that were employed  to elicit this change | Databases: EMBASE (1980–2012),  Medline (1946–2012), AMED (1985–2012), PsycInfo (1806–2012), SportDiscus (1984–2012), CINAHL (1934–2012), PEDro  (1929–2012), Cochrane CENTRAL library.  Supplementary searches: Current trials or unpublished/grey literature were searched including Index to Thesis, DART Europe, ClinicalTrials.gov and the National Institute for Health Research.  Hand searches of relevant journals were performed as well as citation searches using Web of Knowledge (1972–2012). | - RCTs - Pregnant women - No known medical or obstetric condition - BMI in normal, overweight or obese category - Interventions designed to maintain or increase PA during pregnancy as an outcome measure | - Only abstract available - Inclusion of women with diabetes - Intervention designed for underweight pregnant women | - 14 studies - Published: 2002-2012 - Study design: RCT n=14 - n=2,553 women | Not reported |
| Chan *et al.* 2019 [35] | To provide an in-depth review on the major components of physical activity interventions involving land-based exercises that are dedicated for pregnant women and to examine the effectiveness of the interventions on improving exercise self-efficacy, physical activity levels, and pregnancy-related outcomes including depression, pregnancy-related pain, and gestational weight gain. | Databases: PubMed, OVID MEDLINE, EMBASE, PsycINFO, and CINAHL  Supplementary searches: None reported | - RCTs or controlled clinical trials (CCT) - Effectiveness of physical activity interventions or lifestyle interventions that contain a physical activity component on one or more of the following outcomes:   (1) exercise self-efficacy, (2) physical activity levels, (3) depression, (4) lumbopelvic pain, and/or (5) gestational weight gain   - Healthy pregnant women, aged 18 or above, singleton pregnancy, free from medical or obstetrical contraindication against physical activity in pregnancy. - Published before September 2018 | - Pregnant teenage women - Not published in English - Study protocols, case reports, or qualitative studies | - 29 studies - Published: 2000-2018 - Study design: RCT n=28, CCT n=1 - n= 8,257 women | Brazil n=2  Canada n=2  Columbia n=2  Croatia n=1  Finland n=1  Iran n=2  Norway n=9  South Africa n=1  Spain n=2  Sweden n=1  Taiwan n=2  Thailand n=1  Turkey n=2  USA n=1 |
| Flannery *et al.* 2019 [36] | To identify and summarise the evidence for effectiveness of PA interventions on PA levels for pregnant women with overweight and obesity, with an emphasis on the BCTs employed | Databases: MEDLINE, EMBASE, PsychInfo, CINAHL, Cochrane Library, PEDro, SportDiscus, PubMed  Supplementary searches: Manual searches of reference lists were conducted on all eligible articles | - Pilot randomised controlled trials, randomised control trials (RCTs), non-randomised control trials, quasi RCTs, and quasi-experimental studies - PA interventions, aimed at maintaining or increasing PA levels conducted in any setting - Target pregnant women with overweight and obesity (BMI) ≥25 kg/m^2^ - At least one component focusing explicitly on PA, and include a discernible BCT in the intervention description. - Studies were included regardless of treatment intensity, duration or mode of delivery of the intervention. - English language | - Grey literature (non-peer reviewed or without scientific credibility) | - 19 studies - Published: 2009-2018 - Study design: RCT n=16, pilot RCT n=3 - n=7,822 women | Australia n=4  Belgium n=1  Brazil n=2  Denmark n=2  Finland n=1  Ireland n=1  Italy n=1  Netherlands n=1  New Zealand n=1  Norway n=1  UK n=1  USA n=3 |

**S5 References:**

1. Gilinsky A, Swanson V, Power K. Interventions delivered during antenatal care to reduce alcohol consumption during pregnancy: A systematic review. Addiction Research & Theory. 2011;19(3):235-50.

2. Gebara CF, Bhona FM, Ronzani TM, Lourenco LM, Noto AR. Brief intervention and decrease of alcohol consumption among women: a systematic review. Substance abuse treatment, prevention, and policy. 2013;8:31.

3. Lui S, Terplan M, Smith EJ. Psychosocial interventions for women enrolled in alcohol treatment during pregnancy. The Cochrane database of systematic reviews. 2008;(3):Cd006753.

4. Stade BC, Bailey C, Dzendoletas D, Sgro M, Dowswell T, Bennett D. Psychological and/or educational interventions for reducing alcohol consumption in pregnant women and women planning pregnancy. The Cochrane database of systematic reviews. 2009;(2):Cd004228.

5. Agboola S, McNeill A, Coleman T, Leonardi Bee J. A systematic review of the effectiveness of smoking relapse prevention interventions for abstinent smokers. Addiction (Abingdon, England). 2010;105(8):1362-80.

6. Chamberlain C, O'Mara-Eves A, Oliver S, Caird JR, Perlen SM, Eades SJ, et al. Psychosocial interventions for supporting women to stop smoking in pregnancy. The Cochrane database of systematic reviews. 2013;(10):Cd001055.

7. Filion KB, Abenhaim HA, Mottillo S, Joseph L, Gervais A, O'Loughlin J, et al. The effect of smoking cessation counselling in pregnant women: a meta-analysis of randomised controlled trials. BJOG : an international journal of obstetrics and gynaecology. 2011;118(12):1422-8.

8. Hemsing N, Greaves L, O'Leary R, Chan K, Okoli C. Partner support for smoking cessation during pregnancy: a systematic review. Nicotine & tobacco research : official journal of the Society for Research on Nicotine and Tobacco. 2012;14(7):767-76.

9. Hettema JE, Hendricks PS. Motivational interviewing for smoking cessation: a meta-analytic review. Journal of consulting and clinical psychology. 2010;78(6):868-84.

10. Kintz T, Pryor C, Shemami H, Kridli SA-O. Nursing interventions to promote smoking cessation during pregnancy: An integrative review Journal of Nursing Education and Practice. 2014;4(9).

11. Naughton F, Prevost AT, Sutton S. Self-help smoking cessation interventions in pregnancy: a systematic review and meta-analysis. Addiction (Abingdon, England). 2008;103(4):566-79.

12. Su A, Buttenheim AM. Maintenance of smoking cessation in the postpartum period: which interventions work best in the long-term? Maternal and child health journal. 2014;18(3):714-28.

13. Washio Y, Cassey H. Systematic Review of Interventions for Racial/Ethnic-Minority Pregnant Smokers. Journal of smoking cessation. 2016;11(1):12-27.

14. Chamberlain C, O'Mara-Eves A, Porter J, Coleman T, Perlen SM, Thomas J, et al. Psychosocial interventions for supporting women to stop smoking in pregnancy. The Cochrane database of systematic reviews. 2017;2:Cd001055.

15. Griffiths SE, Parsons J, Naughton F, Fulton EA, Tombor I, Brown KE. Are digital interventions for smoking cessation in pregnancy effective? A systematic review and meta-analysis. Health psychology review. 2018;12(4):333-56.

16. Hand D, Ellis J, Carr M, Abatemarco D, Ledgerwood D. Contingency Management Interventions for Tobacco and Other Substance Use Disorders in Pregnancy. Psychology of Addictive Behaviors. 2017;31.

17. Heminger CL, Schindler-Ruwisch JM, Abroms LC. Smoking cessation support for pregnant women: role of mobile technology. Substance abuse and rehabilitation. 2016;7:15-26.

18. Veisani Y, Jenabi E, Delpisheh A, Khazaei S. Effect of prenatal smoking cessation interventions on birth weight: meta-analysis. The journal of maternal-fetal & neonatal medicine : the official journal of the European Association of Perinatal Medicine, the Federation of Asia and Oceania Perinatal Societies, the International Society of Perinatal Obstet. 2019;32(2):332-8.

19. Hubbard G, Gorely T, Ozakinci G, Polson R, Forbat L. A systematic review and narrative summary of family-based smoking cessation interventions to help adults quit smoking. BMC family practice. 2016;17:73.

20. Wilson SM, Newins AR, Medenblik AM, Kimbrel NA, Dedert EA, Hicks TA, et al. Contingency Management Versus Psychotherapy for Prenatal Smoking Cessation: A Meta-Analysis of Randomized Controlled Trials. Women's health issues : official publication of the Jacobs Institute of Women's Health. 2018;28(6):514-23.

21. Bain E, Crane M, Tieu J, Han S, Crowther CA, Middleton P. Diet and exercise interventions for preventing gestational diabetes mellitus. The Cochrane database of systematic reviews. 2015;(4):Cd010443.

22. Brown MJ, Sinclair M, Liddle D, Hill AJ, Madden E, Stockdale J. A systematic review investigating healthy lifestyle interventions incorporating goal setting strategies for preventing excess gestational weight gain. PloS one. 2012;7(7):e39503.

23. Flynn A, Dalrymple K, Barr S, Poston L, Goff L, Rogozińska E, et al. Dietary interventions in overweight and obese pregnant women: A systematic review of the content, delivery, and outcomes of randomized controlled trials. Nutrition Reviews. 2016;74:312-28.

24. Gardner B, Wardle J, Poston L, Croker H. Changing diet and physical activity to reduce gestational weight gain: a meta-analysis. Obesity reviews : an official journal of the International Association for the Study of Obesity. 2011;12(7):e602-20.

25. Girard AW, Olude O. Nutrition education and counselling provided during pregnancy: effects on maternal, neonatal and child health outcomes. Paediatric and perinatal epidemiology. 2012;26 Suppl 1:191-204.

26. Mohd Yusof BN, Firouzi S, Mohd Shariff Z, Mustafa N, Mohamed Ismail NA, Kamaruddin NA. Weighing the evidence of low glycemic index dietary intervention for the management of gestational diabetes mellitus: an Asian perspective. International journal of food sciences and nutrition. 2014;65(2):144-50.

27. Muktabhant B, Lawrie TA, Lumbiganon P, Laopaiboon M. Diet or exercise, or both, for preventing excessive weight gain in pregnancy. The Cochrane database of systematic reviews. 2015;(6):Cd007145.

28. Nascimento SL, Surita FG, Cecatti JG. Physical exercise during pregnancy: a systematic review. Current opinion in obstetrics & gynecology. 2012;24(6):387-94.

29. O'Brien OA, McCarthy M, Gibney ER, McAuliffe FM. Technology-supported dietary and lifestyle interventions in healthy pregnant women: a systematic review. European journal of clinical nutrition. 2014;68(7):760-6.

30. Lau Y, Klainin-Yobas P, Htun TP, Wong SN, Tan KL, Ho-Lim ST, et al. Electronic-based lifestyle interventions in overweight or obese perinatal women: a systematic review and meta-analysis. Obesity reviews : an official journal of the International Association for the Study of Obesity. 2017;18(9):1071-87.

31. Shepherd E, Gomersall JC, Tieu J, Han S, Crowther CA, Middleton P. Combined diet and exercise interventions for preventing gestational diabetes mellitus. The Cochrane database of systematic reviews. 2017;11:Cd010443.

32. Sherifali D, Nerenberg KA, Wilson S, Semeniuk K, Ali MU, Redman LM, et al. The Effectiveness of eHealth Technologies on Weight Management in Pregnant and Postpartum Women: Systematic Review and Meta-Analysis. Journal of medical Internet research. 2017;19(10):e337.

33. Tieu J, Shepherd E, Middleton P, Crowther CA. Dietary advice interventions in pregnancy for preventing gestational diabetes mellitus. The Cochrane database of systematic reviews. 2017;1:Cd006674.

34. Currie S, Sinclair M, Murphy MH, Madden E, Dunwoody L, Liddle D. Reducing the decline in physical activity during pregnancy: a systematic review of behaviour change interventions. PloS one. 2013;8(6):e66385.

35. Chan CWH, Au Yeung E, Law BMH. Effectiveness of Physical Activity Interventions on Pregnancy-Related Outcomes among Pregnant Women: A Systematic Review. Int J Environ Res Public Health. 2019;16(10):1840.

36. Flannery C, Fredrix M, Olander EK, McAuliffe FM, Byrne M, Kearney PM. Effectiveness of physical activity interventions for overweight and obesity during pregnancy: a systematic review of the content of behaviour change interventions. International Journal of Behavioral Nutrition and Physical Activity. 2019;16(1):97.
