# Supplementary material for: The effectiveness of smoking cessation, alcohol reduction, diet and physical activity interventions in changing behaviours during pregnancy: A systematic review of systematic reviews
Source: PLoS One. 2020 May 29;15(5):e0232774. doi: 10.1371/journal.pone.0232774 (PMC7259673; doi:10.1371/journal.pone.0232774)
Supplement: S6 Table — (DOCX) [file pone.0232774.s006.docx]

**S6 Table: Critical appraisal results for each behavioural domain**

**S6a: Alcohol Reviews**

| Author, year | Quality assessment question | | | | | | | | | | | Total score | **Quality category** |
| --- | --- | --- | --- | --- | --- | --- | --- | --- | --- | --- | --- | --- | --- |
|  | 1 | 2 | 3 | 4 | 5 | 6 | 7 | 8 | 9 | 10 | 11 |  |  |
| Gilinsky  *et al.* 2011 [1] | Yes | Yes | Yes | Yes | Yes | Yes | Yes | Yes | No | Unclear | Yes | 9 | **High** |
| Gebara  *et al.* 2013 [2] | Yes | Yes | Yes | No | No | No | Yes | Yes | No | Yes | Yes | 7 | **Moderate** |
| Lui  *et al.* 2008 [3] | yes | yes | yes | yes | yes | yes | N/A | N/A | N/A | N/A | yes | 7 | **Moderate** |
| Stade  *et al.* 2009 [4] | Yes | Yes | Yes | Yes | Yes | Yes | Yes | Yes | Yes | Yes | Yes | 11 | **High** |
| **Subtotal** | **100%**  **(4/4)** | **100%**  **(4/4)** | **100%**  **(4/4)** | **75%**  **(3/4)** | **75%**  **(3/4)** | **75%**  **(3/4)** | **75%**  **(3/4)** | **75%**  **(3/4)** | **25%**  **(1/4)** | **50%**  **(2/4)** | **100%**  **(4/4)** | **Range**  **7-11** | **50% moderate**  **50% high** |

**S6b: Smoking Reviews**

| Author, year | Quality assessment question | | | | | | | | | | | Total score | **Quality category** |
| --- | --- | --- | --- | --- | --- | --- | --- | --- | --- | --- | --- | --- | --- |
|  | 1 | 2 | 3 | 4 | 5 | 6 | 7 | 8 | 9 | 10 | 11 |  |  |
| Agboola  *et al.* 2010 [5] | Yes | Yes | Yes | Yes | Yes | Yes | Yes | Yes | Yes | Unclear | Yes | 10 | **High** |
| Chamberlain  *et al.* 2013 [6] | Yes | Yes | Yes | Yes | Yes | Unclear | Yes | Yes | No | No | Yes | 8 | **High** |
| Filion  *et al.* 2011 [7] | Yes | Yes | Yes | Yes | Yes | Yes | Yes | Yes | No | Yes | Yes | 10 | **High** |
| Hemsing  *et al.* 2012 [8] | Yes | Yes | No | No | Yes | Yes | Yes | Yes | No | Yes | Yes | 8 | **High** |
| Hettema  *et al.* 2010 [9] | Yes | Yes | Yes | Yes | Yes | Yes | Yes | Yes | Yes | Yes | Yes | 11 | **High** |
| Kintz  *et al.* 2014 [10] | yes | unclear | yes | yes | yes | yes | yes | yes | no | yes | yes | 9 | **High** |
| Naughton  *et al.* 2008 [11] | Yes | Yes | Yes | Yes | Yes | Unclear | Yes | Yes | Yes | Yes | Yes | 10 | **High** |
| Su  *et al.* 2014 [12] | Yes | No | No | Yes | Yes | Yes | No | No | No | Yes | Yes | 6 | **Moderate** |
| Washio  *et al.* 2016 [13] | Yes | No | Yes | No | No | No | Unclear | No | No | Yes | Yes | 4 | **Moderate** |
| Chamberlain et al 2017 [14] | Yes | Yes | Yes | Yes | Yes | Unclear | Yes | Yes | Yes | Yes | Yes | 10 | **High** |
| Griffiths et al 2018 [15] | Yes | Yes | Yes | Yes | Yes | Yes | Yes | Yes | Yes | Yes | Yes | 11 | **High** |
| Hand et al 2017 [16] | Yes | Yes | Unclear | Yes | Unclear | Unclear | Yes | Yes | No | Unclear | Yes | 6 | **Moderate** |
| Heminger et al 2016 [17] | Yes | Yes | Yes | Yes | Unclear | Unclear | No | Yes | No | Yes | Yes | 7 | **Moderate** |
| Veisani et al 2017 [18] | yes | yes | yes | yes | No | no | Yes | yes | yes | no | no | 7 | **Moderate** |
| Hubbard et al 2016 [19] | Yes | Yes | Yes | No | Yes | Yes | Yes | Yes | No | Yes | Yes | 9 | **High** |
| Wilson et al 2018 [20] | Yes | Yes | Yes | Yes | Yes | Yes | Yes | Yes | No | Yes | Yes | 10 | **High** |
| **Subtotal** | **100%**  **(16/16)** | **81% (13/16)** | **81% (13/16)** | **81% (13/16)** | **75% (12/16)** | **56% (9/16)** | **81% (13/16)** | **88% (14/16)** | **38% (6/16)** | **75% (12/16)** | **94% (15/16)** | **Range 4-11** | **68.7% high**  **31.3% medium** |

**S6c: Diet and/or Physical Activity Reviews**

| Author, year | Quality assessment question | | | | | | | | | | | Total score | **Quality category** |
| --- | --- | --- | --- | --- | --- | --- | --- | --- | --- | --- | --- | --- | --- |
|  | 1 | 2 | 3 | 4 | 5 | 6 | 7 | 8 | 9 | 10 | 11 |  |  |
| Bain  *et al.* 2015 [21] | Yes | Yes | Yes | Yes | Yes | Yes | Yes | Yes | Yes | Yes | Yes | 11 | **High** |
| Brown  *et al.* 2012 [22] | Yes | Yes | No | Yes | Yes | Yes | Yes | No | Yes | Yes | Yes | 9 | **High** |
| Flynn  *et al.* 2016 [23] | Yes | Yes | Yes | Yes | No | No | Yes | No | No | No | Yes | 6 | **Moderate** |
| Gardner  *et al.* 2011 [24] | Yes | Yes | No | Yes | Yes | No | Yes | Yes | Yes | Yes | Yes | 9 | **High** |
| Webb-Girard *et al.* 2011 [25] | Yes | Yes | Yes | Yes | Yes | No | Yes | Yes | No | Yes | Yes | 9 | **High** |
| Mohd Yusof  *et al.* 2014 [26] | Yes | Yes | Yes | Yes | No | Unclear | Unclear | Yes | No | Yes | Yes | 7 | **Moderate** |
| Muktabhant  *et al.* 2015 [27] | Yes | Yes | Yes | Yes | Yes | Yes | Yes | Yes | Yes | Yes | Yes | 11 | **High** |
| Nasciment  *et al.* 2012 [28] | Yes | Yes | Yes | No | No | Yes | Yes | No | No | Yes | Yes | 7 | **Moderate** |
| O’Brien  *et al.* 2014 [29] | Yes | No | Yes | Yes | Unclear | Unclear | Unclear | Yes | No | Yes | Yes | 6 | **Moderate** |
| Lau et al 2017 [30] | Yes | Yes | Yes | Yes | Yes | Yes | Yes | Yes | Yes | Yes | Yes | 11 | **High** |
| Shepherd et al 2017 [31] | Yes | Yes | Yes | Yes | Yes | Yes | Yes | Yes | Yes | Yes | Yes | 11 | **High** |
| Sherifali et al 2017 [32] | Yes | Yes | Yes | Yes | Yes | Unclear | Yes | Yes | No | Yes | Yes | 9 | **High** |
| Tieu et al 2017 [33] | Yes | Yes | Yes | Yes | Yes | Yes | Yes | Yes | Yes | Yes | Yes | 11 | **High** |
| Currie et al 2013 [34] | Yes | Yes | Yes | Yes | Yes | Unclear | Unclear | No | No | Yes | Yes | 7 | **Moderate** |
| Chan et al 2019 [35] | Yes | Yes | Yes | No | Yes | No | Yes | Yes | No | Yes | Yes | 8 | **High** |
| Flannery et al 2019 [36] | Yes | Yes | Yes | Yes | Yes | No | Yes | Yes | Yes | Yes | Yes | 10 | **High** |
| **Subtotal** | **100% (16/16)** | **94% (15/16)** | **88% (14/16)** | **88% (14/16)** | **75% (12/16)** | **44% (7/16)** | **75% (12/16)** | **75% (12/16)** | **50% (8/16)** | **94% (15/16)** | **100% (16/16)** | **Range 6-11** | **68.7% high**  **31.3% medium** |

**S6 References:**

1. Gilinsky A, Swanson V, Power K. Interventions delivered during antenatal care to reduce alcohol consumption during pregnancy: A systematic review. Addiction Research & Theory. 2011;19(3):235-50.

2. Gebara CF, Bhona FM, Ronzani TM, Lourenco LM, Noto AR. Brief intervention and decrease of alcohol consumption among women: a systematic review. Substance abuse treatment, prevention, and policy. 2013;8:31.

3. Lui S, Terplan M, Smith EJ. Psychosocial interventions for women enrolled in alcohol treatment during pregnancy. The Cochrane database of systematic reviews. 2008;(3):Cd006753.

4. Stade BC, Bailey C, Dzendoletas D, Sgro M, Dowswell T, Bennett D. Psychological and/or educational interventions for reducing alcohol consumption in pregnant women and women planning pregnancy. The Cochrane database of systematic reviews. 2009;(2):Cd004228.

5. Agboola S, McNeill A, Coleman T, Leonardi Bee J. A systematic review of the effectiveness of smoking relapse prevention interventions for abstinent smokers. Addiction (Abingdon, England). 2010;105(8):1362-80.

6. Chamberlain C, O'Mara-Eves A, Oliver S, Caird JR, Perlen SM, Eades SJ, et al. Psychosocial interventions for supporting women to stop smoking in pregnancy. The Cochrane database of systematic reviews. 2013;(10):Cd001055.

7. Filion KB, Abenhaim HA, Mottillo S, Joseph L, Gervais A, O'Loughlin J, et al. The effect of smoking cessation counselling in pregnant women: a meta-analysis of randomised controlled trials. BJOG : an international journal of obstetrics and gynaecology. 2011;118(12):1422-8.

8. Hemsing N, Greaves L, O'Leary R, Chan K, Okoli C. Partner support for smoking cessation during pregnancy: a systematic review. Nicotine & tobacco research : official journal of the Society for Research on Nicotine and Tobacco. 2012;14(7):767-76.

9. Hettema JE, Hendricks PS. Motivational interviewing for smoking cessation: a meta-analytic review. Journal of consulting and clinical psychology. 2010;78(6):868-84.

10. Kintz T, Pryor C, Shemami H, Kridli SA-O. Nursing interventions to promote smoking cessation during pregnancy: An integrative review Journal of Nursing Education and Practice. 2014;4(9).

11. Naughton F, Prevost AT, Sutton S. Self-help smoking cessation interventions in pregnancy: a systematic review and meta-analysis. Addiction (Abingdon, England). 2008;103(4):566-79.

12. Su A, Buttenheim AM. Maintenance of smoking cessation in the postpartum period: which interventions work best in the long-term? Maternal and child health journal. 2014;18(3):714-28.

13. Washio Y, Cassey H. Systematic Review of Interventions for Racial/Ethnic-Minority Pregnant Smokers. Journal of smoking cessation. 2016;11(1):12-27.

14. Chamberlain C, O'Mara-Eves A, Porter J, Coleman T, Perlen SM, Thomas J, et al. Psychosocial interventions for supporting women to stop smoking in pregnancy. The Cochrane database of systematic reviews. 2017;2:Cd001055.

15. Griffiths SE, Parsons J, Naughton F, Fulton EA, Tombor I, Brown KE. Are digital interventions for smoking cessation in pregnancy effective? A systematic review and meta-analysis. Health psychology review. 2018;12(4):333-56.

16. Hand D, Ellis J, Carr M, Abatemarco D, Ledgerwood D. Contingency Management Interventions for Tobacco and Other Substance Use Disorders in Pregnancy. Psychology of Addictive Behaviors. 2017;31.

17. Heminger CL, Schindler-Ruwisch JM, Abroms LC. Smoking cessation support for pregnant women: role of mobile technology. Substance abuse and rehabilitation. 2016;7:15-26.

18. Veisani Y, Jenabi E, Delpisheh A, Khazaei S. Effect of prenatal smoking cessation interventions on birth weight: meta-analysis. The journal of maternal-fetal & neonatal medicine : the official journal of the European Association of Perinatal Medicine, the Federation of Asia and Oceania Perinatal Societies, the International Society of Perinatal Obstet. 2019;32(2):332-8.

19. Hubbard G, Gorely T, Ozakinci G, Polson R, Forbat L. A systematic review and narrative summary of family-based smoking cessation interventions to help adults quit smoking. BMC family practice. 2016;17:73.

20. Wilson SM, Newins AR, Medenblik AM, Kimbrel NA, Dedert EA, Hicks TA, et al. Contingency Management Versus Psychotherapy for Prenatal Smoking Cessation: A Meta-Analysis of Randomized Controlled Trials. Women's health issues : official publication of the Jacobs Institute of Women's Health. 2018;28(6):514-23.

21. Bain E, Crane M, Tieu J, Han S, Crowther CA, Middleton P. Diet and exercise interventions for preventing gestational diabetes mellitus. The Cochrane database of systematic reviews. 2015;(4):Cd010443.

22. Brown MJ, Sinclair M, Liddle D, Hill AJ, Madden E, Stockdale J. A systematic review investigating healthy lifestyle interventions incorporating goal setting strategies for preventing excess gestational weight gain. PloS one. 2012;7(7):e39503.

23. Flynn A, Dalrymple K, Barr S, Poston L, Goff L, Rogozińska E, et al. Dietary interventions in overweight and obese pregnant women: A systematic review of the content, delivery, and outcomes of randomized controlled trials. Nutrition Reviews. 2016;74:312-28.

24. Gardner B, Wardle J, Poston L, Croker H. Changing diet and physical activity to reduce gestational weight gain: a meta-analysis. Obesity reviews : an official journal of the International Association for the Study of Obesity. 2011;12(7):e602-20.

25. Girard AW, Olude O. Nutrition education and counselling provided during pregnancy: effects on maternal, neonatal and child health outcomes. Paediatric and perinatal epidemiology. 2012;26 Suppl 1:191-204.

26. Mohd Yusof BN, Firouzi S, Mohd Shariff Z, Mustafa N, Mohamed Ismail NA, Kamaruddin NA. Weighing the evidence of low glycemic index dietary intervention for the management of gestational diabetes mellitus: an Asian perspective. International journal of food sciences and nutrition. 2014;65(2):144-50.

27. Muktabhant B, Lawrie TA, Lumbiganon P, Laopaiboon M. Diet or exercise, or both, for preventing excessive weight gain in pregnancy. The Cochrane database of systematic reviews. 2015;(6):Cd007145.

28. Nascimento SL, Surita FG, Cecatti JG. Physical exercise during pregnancy: a systematic review. Current opinion in obstetrics & gynecology. 2012;24(6):387-94.

29. O'Brien OA, McCarthy M, Gibney ER, McAuliffe FM. Technology-supported dietary and lifestyle interventions in healthy pregnant women: a systematic review. European journal of clinical nutrition. 2014;68(7):760-6.

30. Lau Y, Klainin-Yobas P, Htun TP, Wong SN, Tan KL, Ho-Lim ST, et al. Electronic-based lifestyle interventions in overweight or obese perinatal women: a systematic review and meta-analysis. Obesity reviews : an official journal of the International Association for the Study of Obesity. 2017;18(9):1071-87.

31. Shepherd E, Gomersall JC, Tieu J, Han S, Crowther CA, Middleton P. Combined diet and exercise interventions for preventing gestational diabetes mellitus. The Cochrane database of systematic reviews. 2017;11:Cd010443.

32. Sherifali D, Nerenberg KA, Wilson S, Semeniuk K, Ali MU, Redman LM, et al. The Effectiveness of eHealth Technologies on Weight Management in Pregnant and Postpartum Women: Systematic Review and Meta-Analysis. Journal of medical Internet research. 2017;19(10):e337.

33. Tieu J, Shepherd E, Middleton P, Crowther CA. Dietary advice interventions in pregnancy for preventing gestational diabetes mellitus. The Cochrane database of systematic reviews. 2017;1:Cd006674.

34. Currie S, Sinclair M, Murphy MH, Madden E, Dunwoody L, Liddle D. Reducing the decline in physical activity during pregnancy: a systematic review of behaviour change interventions. PloS one. 2013;8(6):e66385.

35. Chan CWH, Au Yeung E, Law BMH. Effectiveness of Physical Activity Interventions on Pregnancy-Related Outcomes among Pregnant Women: A Systematic Review. Int J Environ Res Public Health. 2019;16(10):1840.

36. Flannery C, Fredrix M, Olander EK, McAuliffe FM, Byrne M, Kearney PM. Effectiveness of physical activity interventions for overweight and obesity during pregnancy: a systematic review of the content of behaviour change interventions. International Journal of Behavioral Nutrition and Physical Activity. 2019;16(1):97.
