# Supplementary material for: The effectiveness of smoking cessation, alcohol reduction, diet and physical activity interventions in changing behaviours during pregnancy: A systematic review of systematic reviews
Source: PLoS One. 2020 May 29;15(5):e0232774. doi: 10.1371/journal.pone.0232774 (PMC7259673; doi:10.1371/journal.pone.0232774)
Supplement: S7 Table — (DOCX) [file pone.0232774.s007.docx]

**S7 Tables:** **Overlap of included studies in the systematic reviews**

**S7a: Included studies in alcohol reviews**

| **Review** | **References of included behavioural interventions delivered in pregnancy** |
| --- | --- |
| Gilinsky  *et al.* 2011[1] | 1. Chang et al., 2005 Chang, G., McNamara, T.K., Orav, E.J., Koby, D., Lavigne, A., & Ludman, B. (2005). Brief intervention for prenatal alcohol use: A randomized trial. Obstetrics & Gynecology, 105, 991–998. 2. Chang, Wilkins-Haug, Berman, & Goetz, 1999 Chang, G., Wilkins-Haug, L., Berman, S., & Goetz, M.A. (1999). Brief intervention for alcohol use in pregnancy: A randomised trial. Addiction, 94, 1499–1508. 3. Handmaker, Miller, & Manicke, 1999 Handmaker, N.S., Miller, W.R., & Manicke, M. (1999). Findings of a pilot study of motivational interviewing with pregnant drinkers. Journal of Studies on Alcohol, 60, 285–287. 4. Meberg et al., 1986 Meberg, A., Halvorsen, B., Holter, B., Ek, I.J., Askeland, A., Gaaserud, W., & Steinsvag, J. (1986). Moderate alcohol consumption – Need for intervention programs in pregnancy? Acta Obstetricia et Gynecologica Scandinavica, 65, 861–864. 5. O’Connor & Whaley, 2007 O’Connor, M.J., & Whaley, S.E. (2007). Brief intervention for alcohol use by pregnant women. American Journal of Public Health, 97, 252–258. 6. Reading, Campbell, Cox, & Sledmere, 1982 Reading, A.E., Campbell, S., Cox, D.N., & Sledmere, C.M. (1982). Health beliefs and health care behaviour in pregnancy. Psychological Medicine, 12, 379–383. 7. Reynolds, Coombs, Lowe, Peterson, & Gayoso, 1996 Reynolds, K.D., Coombs, D.W., Lowe, J.B., Peterson, P.L., & Gayoso, E. (1996). Evaluation of a self-help program to reduce alcohol consumption among pregnant women. International Journal of the Addictions, 30, 427–443. 8. Waterson & Murray-Lyon, 1990 Waterson, E.J., & Murray-Lyon, I.M. (1990). Preventing fetal alcohol effects: A trial of three methods of giving information in the antenatal clinic. Health Education Research, 5, 53–61.   Supplementary data:   1. Chang, G., Wilkins-Haug, L., Berman, S., & Goetz, M.A. (1999). Brief intervention for alcohol use in pregnancy: A randomised trial. Addiction, 94, 1499–1508. 2. Chang, G., McNamara, T.K., Orav, E.J., & Wilkins-Haug, L. (2006). Brief intervention for prenatal alcohol use: The role of drinking goal selection. Journal of Substance Abuse Treatment, 31, 419–424. |
| Gebara  *et al.* 2013[2] | 1. Nilsen P, Holmqvist M, Bendtsen P, Hultgren E, Cedergren M: Is questionnaire-based alcohol counseling more effective for pregnant women than standard maternity care? J Womens Health (Larchmt) 2010, 19: 161–167 2. Armstrong MA, Kaskutas LA, Witbrodt J, Taillac CJ, Hung YY, Osejo VM, Escobar GJ: Using drink size to talk about drinking during pregnancy: a randomized clinical trial of early start plus. Soc Work Health Care 2009, 48: 90 –103 3. Chang G, McNamara TK, Wilkins-Haug L, Orav EJ: Brief intervention for prenatal alcohol use: the role of drinking goal selection. J Subst Abuse Treat 2006, 31: 419–424 4. Witbrodt J, Kaskutas LA, Diehl S, Armstrong MA, Escobar GJ, Taillac C, Osejo V:Using drink size to talk about drinking during pregnancy: early startplus. J Addict Nurs 2007, 18:199–206 5. O’Connor MJ, Whaley SE: Brief intervention for alcohol use by pregnant women. Am J Public Health 2007, 97:252–258. 6. Chang G, McNamara T, Wilkins-Haug L, Orav EJ: Stages of change and prenatal alcohol use. J Subst Abuse Treat 2007,32: 105–109. 7. Yonkers KA, Howell HB, Allen AE, Ball AS, Pantalon MV, Rounsaville BJ: A treatment for substance abusing pregnant women. Arch Womens Ment Health 2009, 12:221–227 8. Tzilos GK, Sokol RJ, Ondersma SJ: A randomized phase I trial of a brief computer-delivered intervention for alcohol use during pregnancy. J Womens Health 2011, 20: 1517–1524 |
| Stade  *et al.* 2009[3] | - 1. Chang G. Personal communication. Email communication received 2008 August.   2. Chang G, Goetz MA, Wilkins-Haug L, Berman S. A brief intervention for prenatal alcohol use: an in-depth look. Journal of Substance Abuse Treatment 2000;18:365–9.   3. Chang G, Wilkins-Haug L, Berman S, Goetz MA. Brief intervention for alcohol use in pregnancy: randomized trial. Addiction 1999;94(10):1499–508.   4. Handmaker N, Miller W, Manicke M. Findings of a pilot study of motivational interviewing with pregnant drinkers. *Journal of Studies on Alcohol* 1999;**60**: 285–7.   5. O’Connor M, Whaley S. Brief intervention for alcohol use by pregnant women. *American Journal of Public Health* 200; **97**(2):252–8.   6. O’Connor MJ. Personal communication. Email communication received 2008 July.   7. Reynolds KD, Coombs DW, Lowe JB, Peterson PL, Gayoso E. Evaluation of a self-help program to reduce alcohol consumption among pregnant women. *International* *Journal of the Addictions* 1995;**30**(4):427–43. |
| Lui  *et al.* 2008[4] | No studies included in the review |

**S7b: Unique publications inlcuded in alcohol reviews**

| **Alcohol unique publications** |
| --- |
| 1. Armstrong MA, Kaskutas LA, Witbrodt J, Taillac CJ, Hung YY, Osejo VM, Escobar GJ: Using drink size to talk about drinking during pregnancy: a randomized clinical trial of early start plus. Soc Work Health Care 2009, 48: 90 –103 |
| 1. Chang G, McNamara T, Wilkins-Haug L, Orav EJ: Stages of change and prenatal alcohol use. J Subst Abuse Treat 2007,32: 105–109 |
| 1. Chang G, McNamara TK, Wilkins-Haug L, Orav EJ: Brief intervention for prenatal alcohol use: the role of drinking goal selection. J Subst Abuse Treat 2006, 31: 419–424 |
| 1. Chang et al., 2005 Chang, G., McNamara, T.K., Orav, E.J., Koby, D., Lavigne, A., & Ludman, B. (2005). Brief intervention for prenatal alcohol use: A randomized trial. Obstetrics & Gynecology, 105, 991–998 |
| 1. Chang G, Goetz MA, Wilkins-Haug L, Berman S. A brief intervention for prenatal alcohol use: an in-depth look. Journal of Substance Abuse Treatment 2000;18:365–9 |
| 1. Chang G, Wilkins-Haug L, Berman S, Goetz MA. Brief intervention for alcohol use in pregnancy: randomized trial. Addiction 1999;94(10):1499–508 |
| 1. Handmaker, Miller, & Manicke, 1999 Handmaker, N.S., Miller, W.R., & Manicke, M. (1999). Findings of a pilot study of motivational interviewing with pregnant drinkers. Journal of Studies on Alcohol, 60, 285–287 |
| 1. Meberg et al., 1986 Meberg, A., Halvorsen, B., Holter, B., Ek, I.J., Askeland, A., Gaaserud, W., & Steinsvag, J. (1986). Moderate alcohol consumption – Need for intervention programs in pregnancy? Acta Obstetricia et Gynecologica Scandinavica, 65, 861–864 |
| 1. Nilsen P, Holmqvist M, Bendtsen P, Hultgren E, Cedergren M: Is questionnaire-based alcohol counseling more effective for pregnant women than standard maternity care? J Womens Health (Larchmt) 2010, 19: 161–167 |
| 1. O’Connor MJ, Whaley SE: Brief intervention for alcohol use by pregnant women. Am J Public Health 2007, 97:252–258 |
| 1. Reading, Campbell, Cox, & Sledmere, 1982 Reading, A.E., Campbell, S., Cox, D.N., & Sledmere, C.M. (1982). Health beliefs and health care behaviour in pregnancy. Psychological Medicine, 12, 379–383 |
| 1. Reynolds, Coombs, Lowe, Peterson, & Gayoso, 1996 Reynolds, K.D., Coombs, D.W., Lowe, J.B., Peterson, P.L., & Gayoso, E. (1996). Evaluation of a self-help program to reduce alcohol consumption among pregnant women. International Journal of the Addictions, 30, 427–443 |
| 1. Tzilos GK, Sokol RJ, Ondersma SJ: A randomized phase I trial of a brief computer-delivered intervention for alcohol use during pregnancy. J Womens Health 2011, 20: 1517–1524 |
| 1. Waterson & Murray-Lyon, 1990 Waterson, E.J., & Murray-Lyon, I.M. (1990). Preventing fetal alcohol effects: A trial of three methods of giving information in the antenatal clinic. Health Education Research, 5, 53–61 |
| 1. Witbrodt J, Kaskutas LA, Diehl S, Armstrong MA, Escobar GJ, Taillac C, Osejo V:Using drink size to talk about drinking during pregnancy: early startplus. J Addict Nurs 2007, 18:199–206 |
| 1. Yonkers KA, Howell HB, Allen AE, Ball AS, Pantalon MV, Rounsaville BJ: A treatment for substance abusing pregnant women. Arch Womens Ment Health 2009, 12:221–227 |

**S7c: Included studies in smoking reviews**

| **Review** | **References of included behavioural interventions delivered in pregnancy** |
| --- | --- |
| Agboola  *et al.* 2010[5] | 1. Lowe J. B., Windsor R., Balanda K. P., Woodby L. Smoking relapse prevention methods for pregnant women: a formative evaluation. Am J Health Promot 1997; 11: 244–6. 2. Ershoff D. H., Quinn V. P., Patricia D. M. Relapse prevention among women who stop smoking early in pregnancy: a randomized clinical trial of a self-help intervention. Am J Prev Med 1995; 11: 178–84. 3. Hajek P., West R., Lee A., Foulds J., Owen L., Eiser J. R. Randomized controlled trial of a midwife-delivered brief smoking cessation intervention in pregnancy. Addiction 2001; 96: 485–94. 4. Pbert L., Ockene J. K., Zapka J.,Ma Y., Goins K. V., Oncken C. et al. A community health center smoking-cessation intervention for pregnant and postpartum women. Am J Prev Med 2004; 26: 377–85. 5. Ruger J. P.,Weinstein M. C., Hammond S. K., KearneyM. H., Emmons K. M. Cost-effectiveness of motivational interviewing for smoking cessation and relapse prevention among low-income pregnant women: a randomized controlled trial. Value Health 2008; 11: 191–8. 6. Secker-Walker R. H., Solomon L. J., Flynn B. S., Skelly J. M., Mead P. B. Smoking relapse prevention during pregnancy. AmJ Prev Med 1998; 15: 25–31. 7. McBride C. M., Curry S. J., Lando H. A., Pirie P. L., Grothaus L. C., Nelson J. C. Prevention of relapse in women who quit smoking during pregnancy. Am J Public Health 1999; 89: 706–11. 8. McBride C. M., Baucom D. H., Peterson B. L., Pollak K. I., Palmer C., Westman E. et al. Prenatal and postpartum smoking abstinence a partner-assisted approach. Am J Prev Med 2004; 27: 232–8. 9. Morasco B. J., Dornelas E. A., Fischer E. H., Oncken C., Lando H. A. Spontaneous smoking cessation during pregnancy among ethnic minority women: a preliminary investigation. Addict Behav 2006; 31: 203–10. 10. Secker-Walker R. H., Solomon L. J., Flynn B. S., Skelly J. M. Smoking relapse prevention counseling during prenatal and early postnatal care. AmJ Prev Med 1995; 11: 86–93. |
| Chamberlain  *et al.* 2013[6] | 1. Albrecht 1998 *{published data only}*Albrecht S, Cassidy B, Salamie D, Reynolds M. What’s happening. A smoking cessation intervention for pregnant adolescents: implications for nurse practitioners. *Journal of American Academy of Nurse Practitioners* 1999;11(4):155–9. 2. Albrecht S, Cornelius M, Braxter B, Reynolds M, Stone C, Cassidy B. An assessment of nicotine dependence among pregnant adolescents. *Journal of Substance Abuse Treatment* 1999;16(4):337–44. ∗ 3. Albrecht S, Stone CA, Payne L, Reynolds MD. A preliminary study of the use of peer support in smoking cessation programs for pregnant adolescents. *Journal of the American Academy of Nurse Practitioners* 1998;10:119–25. 4. Albrecht SA, Higgins LW, Stone C. Factors relating to pregnant adolescents’ decisions to complete a smoking cessation intervention. *Journal of Pediatric Nursing* 1999;14 (5):322–8. 5. Albrecht 2006 *{published data only}*Albrecht SA, Caruthers D. Characteristics of inner-city pregnant Smoking teenagers. *Journal of Obstetric Gynecologic and Neonatal Nursing* 2002;31:462–9. ∗ 6. Albrecht SA, Caruthers D, Patrick T, Reynolds M, Salamie D, Higgins LW, et al. A randomised controlled trial of a smoking cessation intervention for pregnant adolescents. *Nursing Research* 2006;55(6):402–10. 7. Albrecht SA, Higgins LW, Lebow H. Knowledge about the deletrious effects of smoking and its relationship to smoking cessation among pregnant adolescents. *Adolescence* 2000;35 (140):709–16. 8. Albrecht SA, Patrick T, Kim Y, Caruthers D. A randomised controlled trial of a smoking cessation intervention for pregnant adolescents. Society for Research on Nicotine and Tobacco 9th Annual Meeting; 2003 February 19-23; New Orleans, Louisiana. 2003:91. 9. Baric 1976 *{published data only}*Baric L, MacArthur C. Health norms in pregnancy. *British Journal of Preventive and Social Medicine* 1977;31:30–8. 10. Baric L, MacArthur C, Sherwood M. A study of health education aspects of smoking in pregnancy. *International Journal of Health Education* 1976;19(2 Suppl):1–17. 11. Bauman 1983 *{published data only}*Bauman KE, Koch GG, Dent CW, Bryan ES. The influence of observing carbon monoxide level on cigarette smoking by public prenatal patients. *American Journal of Public Health* 1983;73:1089–91. 12. Belizan 1995 *{published data only}*∗ Belizan JM, Villar J, Victora C, Farnot U, Langer A, Barros F. Impact of health education during pregnancy on behavior and utilization of health resources. *American Journal of Obstetrics and Gynecology* 1995;173:894–9. 13. Victora CG, Langer A, Barros F, Belizan J, Farnot U, Villar J, et al. The Latin American Multicenter Trial on psychosocial support during pregnancy: methodology and baseline comparability. *Controlled Clinical Trials* 1994;15: 379–94. 14. Villar J, Farnot U, Barros F, Victora C, Langer A, Belizan JM. A randomized trial of psychosocial support during high-risk pregnancies. *New England Journal of Medicine* 1992;327:1266–71. 15. Bullock 1995 *{published data only}*Bullock LF, Hornblow AR, Duff GB, Wells JE. Telephone support for pregnant women: outcome in late pregnancy. *New Zealand Medical Journal* 1995;108:476–8. 16. Bullock 2009 *{published data only}*∗ Bullock L, Everett KD, Mullen PD, Geden E, Longo DR, Madsen R. Baby BEEP: A randomized controlled trial of nurses’ individualized social support for poor rural pregnant smokers. *Maternal and Child Health Journal* 2009;13(3): 395–406. 17. Bullock LF, Everett KD, Mullen PD. Baby beep: a randomized clinical trial of smoking cessation for low- income rural pregnant women using nurse-delivered social support. *Annals of Behavioral Medicine* 2008;35:S99. 18. Burling 1991 *{published data only}*Burling TA, Bigelow GE, Robinson JC, Mead AM. Smoking during pregnancy: reduction via objective assessment and directive advice. *Behavior Therapy* 1991;22:31–40. 19. Byrd 1993 *{published data only}*Byrd JC, Meade CD. Smoking cessation among pregnant women in an urban setting. *Wisconsin Medical Journal* 1993;92:609–12. 20. Campbell 2006 *{published data only}*∗ Campbell E, Walsh RA, Sanson-Fisher, Burrows S, Stojanovski E. A group randomised trial of two methods for disseminating a smoking cessation programme to public antenatal clinics: effects on patient outcomes. *Tobacco Control* 2006;15(2):97–102. 21. Cooke M, Mattick R, Campbell E. The influence of individual and organisational factors on the reported smoking intervention practices of staff in 20 antenatal clinics. *Drug and Alcohol Review* 1998;17(2):175–85. 22. Cooke M, Mattick RP, Campbell E. A description of the adoption of the ’Fresh Start’ smoking cessation program by antenatal clinic managers. *Australian Journal of Advanced Nursing* 2000;18(1):13–21 23. Cooke M, Mattick RP, Campbell E. The dissemination of a smoking cessation program to 23 antenatal clinics: the predictors of initial program adoption by managers. *Australian and New Zealand Journal of Public Health* 1999; 23(1):99–103. 24. Cooke M, Mattick RP, Walsh RA. Differential uptake of a smoking cessation programme disseminated to doctors and midwives in antenatal in antenatal clinics. *Addiction* 2001; 96(3):495–505. 25. Cooke M, Mattick RP, Walsh RA. Implementation of the ’Fresh Start’ smoking cessation programme to 23 antenatal clinics: a randomized controlled trial investigating two methods of dissemination. *Drug and Alcohol Review* 2001; 20:19–28. 26. Cinciripini 2000 *{published data only}*Blalock JA, Fouladi RT, Wetter DW, Cinciripini PM. Depression in pregnant women seeking smoking cessation treatment. *Addictive Behaviours* 2005;30(6):1195–208. 27. Blalock JA, Robinson JD, Wetter DW, Cinciripini PM. Relationship of DSM-IV-Based depressive disorders to smoking cessation and smoking reduction in pregnant women. *American Journal on Addictions* 2006;15(4): 268–77. ∗ 28. Cinciripini PM, McClure JB, Wetter DW, Perry J, Blalock JA, Cinciripini LG, et al. An evaluation of videotaped vignettes for smoking cessation and relapse prevention during pregnancy: The Very Important Pregnant Smokers (VIPS) Program. *Tobacco Control* 2000;9(3):iii61–iii63. 29. Cinciripini 2010 *{published data only}*Cinciripini PM, Blalock JA, Minnix JA, Robinson JD, Brown VL, Lam C, et al. Effects of an intensive depression- focused intervention for smoking cessation in pregnancy. *Journal of Consulting & Clinical Psychology* 2010;78(1): 44–54. 30. Cook 1995 *{published data only}*Cook C, Ward S, Myers S, Spinnato J. A prospective, randomized evaluation of intensified therapy for smoking reduction in pregnancy. *American Journal of Obstetrics and Gynecology* 1995;172:290. 31. Cope 2003 *{published data only}*Cope G, Nayyar P, Holder R, Gibbons J, Brunce R. A simple near patient test for nicotine and its metabolites in urine to assess smoking habit. *Clinical Chimica Acta* 1996; 256:135–49. Cope GF. Smoking status and pregnancy: point of care cotinine test. *BMJ* 2009;339:b5652. 32. Cope GF, Nayyar P, Holder R. Feedback from a point- of-care test for nicotine intake to reduce smoking during pregnancy. *Annals of Clinical Biochemistry* 2003;40(Pt 6): 674–9. 33. Cope GF, Nayyar P, Holder R. Measurement of nicotine intake in pregnant women - associations to changes in blood cell count. *Nicotine & Tobacco Research* 2001;3(2):119–22. 34. Donatelle 2000 *{published data only}*Donatelle RJ, Hudson D. Using 5 A’s and incentives to promote prenatal smoking cessation. National Conference of Tobacco or Health; 2002 November 19-21; San Francisco, California, USA. 2002. 35. Donatelle RJ, Hudson D, Dobie S, Goodall A, Hunsberger M, Oswald K. Incentives in smoking cessation: status of the field and implications for research and practice with pregnant smokers. *Nicotine and Tobacco Research* 2004;6 (S2):S163–S179. 36. Donatelle RJ, Prows SL, Champeau D, Hudson D. Randomised controlled trial using social support and financial incentives for high risk pregnant smokers: Significant Other Supporter (SOS) program. *Tobacco Control* 2000;9 Suppl 3:iii67–iii69. 37. Donovan 1977 *{published data only}*∗ Donovan J. Randomised controlled trial of anti-smoking advice in pregnancy. *British Journal of Preventive and Social Medicine* 1977;31(1):6–12. 38. Donovan JW. Randomised controlled trial of anti-smoking advice in pregnancy. *Journal of Epidemiology and Community Health* 1996;50(3):232–6. 39. Donovan JW, Burgess PL, Hossack CM, Yudkin GD. Routine advice against smoking in pregnancy. *Journal of the Royal College of General Practitioners* 1975;25(153):264–8. 40. Dornelas 2006 *{published data only}* Dornelas EA, Magnavita J, Beazoglou T, Fischer EH, Oncken C, Lando H, et al. Efficacy and cost-effectiveness of a clinic-based counseling intervention tested in an ethnically diverse sample of pregnant smokers. *Patient Education and Counseling* 2006;64(1-3):342–9. 41. Morasco BJ, Dornelas EA, Fischer EH, Oncken C, Lando HA. Spontaneous smoking cessation during pregnancy among ethnic minority women: a preliminary investigation. *Addictive Behaviors* 2006;31(2):203–10. 42. Dunkley 1997 *{published data only}*Dunkley J. Training midwives to help pregnant women stop smoking. *Nursing Times* 1997;93(5):64–6. 43. Eades 2012 *{published data only}*∗ Eades SJ, Sanson-Fisher RW, Wenitong M, Panaretto K, D’Este C, Gilligan C, et al. An intensive smoking intervention for pregnant Aboriginal and Torres Strait Islander women: a randomised controlled trial. *Medical Journal of Australia* 2012;197(1):42–6. Gilligan C. *A pilot randomised controlled trial to test the effectiveness of an intervention to help Aboriginal and Torres Strait Islander women to quit smoking during pregnancy: study design and preliminary results [thesis]*. Newcastle, Australia: University of Newcastle, 2008. 44. Gilligan C, Sanson-Fisher R, Eades S, Wenitong M, Panaretto K, D’Este C. Assessing the accuracy of self- reported smoking status and impact of passive smoke exposure among pregnant Aboriginal and Torres Strait Islander women using cotinine biochemical validation. *Drug and Alcohol Review* 2010;29:35–40. 45. Gilligan C, Sanson-Fisher RW, D-Este C, Eades S, Wenitong M. Knowledge and attitudes regarding smoking during pregnancy among Aboriginal and Torres Strait Islander women. *Medical Journal of Australia* 2009;190(10): 557–61. 46. Panaretto KS, Mitchell MR, Anderson L, Gilligan C, Buettner P, Larkins SL, et al. Tobacco use and measuring nicotine dependence among urban Indigenous pregnant women. *Medical Journal of Australia* 2009;191(10):554–7. 47. El-Mohandes 2011 *{published data only}*Blake S, El-Mohandes A, Schwartz D, El-Khorazaty N, Gantz M, Joseph J, et al. Promoting smoking cessation during pregnancy and preventing postpartum relapse [abstract]. Pediatric Academic Societies Annual Meeting; 2005 May 14-17; Washington DC, USA. 2005:Abstract no: 3074. 48. Blake S, Joseph J, Schwartz D, El-Khorazaty N, Gantz M, El-Mohandes A, et al. Preventing prenatal and postpartum environmental tobacco smoke (ETS) exposure [abstract]. Pediatric Academic Societies Annual Meeting; 2005 May 14-17; Washington DC, USA. 2005:Abstract no: 2353. 49. Blake SM, Murray KD, El-Khorazaty MN, Gantz MG, Kiely M, Best D, et al. Environmental tobacco smoke avoidance among pregnant African-American nonsmokers. *American Journal of Preventive Medicine* 2009;36(3): 225–34. 50. El-Khorazaty MN, Johnson AA, Kiely M, El-Mohandes AA, Subramanian S, Laryea HA, et al. Recruitment and retention of low-income minority women in a behavioral intervention to reduce smoking, depression, and intimate partner violence during pregnancy. *BMC Public Health* 2007;7:233. [PUBMED: 17822526] 51. El-Mohandes AA, El-Khorazaty MN, Kiely M, Gantz MG. Smoking cessation and relapse among pregnant African-American smokers in Washington, DC. *Maternal & Child Health Journal* 2011;15 Suppl 1:S96–S105. El-Mohandes AA, Kiely M, Blake SM, Gantz MG, El- Khorazaty MN. An intervention to reduce environmental tobacco smoke exposure improves pregnancy outcomes. *Pediatrics* 2010;125(4):721–8. 52. El-Mohandes AA, Kiely M, Joseph JG, Subramanian S, Johnson AA, Blake SM, et al. An intervention to improve postpartum outcomes in African-American mothers: a randomized controlled trial. *Obstetrics & Gynecology* 2008; 112(3):611–20. 53. El-Mohandes AAE. A psycho-behavioral intervention on African American pregnant women with a history of intimate partner violence (IPV) improves birth weight distribution of their newborns [abstract]. Pediatric Academic Societies Annual Meeting; 2006 April 29-May 2; San Francisco, CA, USA. 2006. 54. El-Mohandes AAE. An integrated behavioral intervention reduces rates of moderate and extreme prematurity in African American (AA) mothers with a history of smoking during pregnancy [abstract]. Pediatric Academic Societies Annual Meeting; 2006 April 29-May 2; San Francisco, CA, USA. 2006. 55. El-Mohandes AAE, Kiely M, Gantz MG, El-Khorazaty N. A multiple risk factor behavioral intervention reduces environmental tobacco smoke exposure. Pediatric Academic Societies Annual Meeting; 2007 May 5-8; Toronto, Canada 2007. 56. El-Mohandes AAE, for the NIH-DC initiative to reduce infant mortality. An integrated psycho-behavioral intervention during pregnancy has significant effects in reducing risks during the post-partum period in African- American women. Pediatric Academic Societies Annual Meeting; 2005 May 14-17; Washington DC, USA. 2005: Abstract no: 39. 57. El-Mohandes AEE, Kiely M, Gantz MG, El-Khorazaty N. Very preterm birth is reduced in women receiving an integrated behavioural intervention: A randomized controlled trial. *Maternal Child Health Journal* 2011;15: 19–28. Joseph J, for NIH-DC initiative to reduce infant mortality. Randomized trial to reduce 4 behaviors linked to adverse pregnancy outcomes among 1048 inner-city African American women [abstract]. Pediatric Academic Societies Annual Meeting; 2005 May 14-17; Washington DC, USA. 2005:Abstract no: 1701. 58. Joseph JG, El-Mohandes AA, Kiely M, El-Khorazaty MN, Gantz MG, Johnson AA, et al. Reducing psychosocial and behavioral pregnancy risk factors: results of a randomized clinical trial among high-risk pregnant African American women. *American Journal of Public Health* 2009;99(6): 1053–61. 59. Katz, KS, Blake SM, Milligan RA, Sharps PW, White DB, Rodan MF, et al. The design, implementation and acceptability of an integrated intervention to address multiple behavioural and psychosocial risk factors among pregnant African American women. *BMC Pregnancy and Childbirth* 2008;8(22):doi:10.1186/1471–2393-8-22. 60. Kiely M, El-Khorazaty MN, El-Mohandes AAE. Depression and smoking during pregnancy impact the efficacy of an integral behavioral intervention to resolve risks. Pediatric Academic Societies Annual Meeting; 2007 May 5-8; Toronto, Canada 2007. 61. Subramanian S, Katz KS, Rodam M, Gantz MG, El- Khorazaty NM, Johnson A, et al. An integrated randomized intervention to reduce behavioural and psychosocial risks: Pregnancy and neonatal outcomes. *Maternal Child Health Journal* 2012;16:545–54. 62. Tan S, Courtney LP, El-Mohandes AAE, Gantz MG, Blake SM, Thornberry J, et al. Relationships between self- reported smoking, household environmental tobacco smoke exposure and depressive symptoms in a pregnant minority population. *Maternal Child Health Journal* 2011;15:S65- S74. 63. Ershoff 1989 *{published data only}*Ershoff DH, Lairson DR, Mullen PD, Quinn VP. Pregnancy and medical cost outcomes of a self-help prenatal smoking cessation program in an HMO. *Public Health Reports* 1990; 105(4):340–7. 64. Ershoff DH, Quinn VP, Mullen PD. A randomized trial of a serialized self-help smoking cessation program for pregnant women in an HMO. *American Journal of Public Health* 1989;79(2):182–7. 65. Ershoff DH, Quinn VP, Mullen PD. Relapse prevention among women who stop smoking early in pregnancy: a randomized clinical trial of a self-help intervention. *American Journal of Preventive Medicine* 1995;11(3): 178–84. Mullen PD, Carbonari JP, Tabak ER, Glenday MC. Improving disclosure of smoking by pregnant women. *American Journal of Obstetrics and Gynecology* 1991;165: 409–13. 66. Quinn VP, Mullen PD, Ershoff DH. Women who stop smoking spontaneously prior to prenatal care and predictors of relapse before delivery. *Addictive Behaviour* 1991;16(1- 2):29–40. 67. Ershoff 1999 *{published data only}*∗ Ershoff DH, Quinn VP, Boyd NR, Stern J, Gregory M, Wirtschafter D. The Kaiser Permanente prenatal smoking cessation trial. When more isn’t better, what is enough?. *American Journal of Preventive Medicine* 1999;17(3):161–8. 68. Ershoff DH, Quinn VP, Boyd NR, Stern J, Gregory M, Wirtschafter D. The Kaiser Permanente prenatal smoking cessation trial: when more isn’t better, what is enough?. *Tobacco Control* 2000;9(Suppl 3):iii60. 69. Ershoff DH, Solomon LJ, Dolan-Mullen P. Predictors of intentions to stop smoking early in prenatal care. *Tobacco Control* 2000;9(3):41. 70. Gielen 1997 *{published data only}*Gielen AC, Windsor R, Faden RR, O’Campo P, Repke J, Davis M. Evaluation of a smoking cessation intervention for pregnant women in an urban prenatal clinic. *Health Education Research* 1997;12(2):247–54. 71. Graham 1992 *{published data only}*Graham AV, Reeb KG, Kitson GC, Zyzanski SJ, Frank SH. A clinical trial to reduce the rate of low birth weight in an inner-city black population. *Family Medicine* 1992;24: 439–46. 72. Haddow 1991 *{published data only}*Haddow JE, Polomak JE, Sepulveda D. Smoking cessation during routine public prenatal care. *American Journal of Public Health* 1995;85(10):1451–2. ∗ Haddow JE, Wald NJ, Palomaki GE, Kloza EM, Knight GJ. Cotinine-assisted intervention in pregnancy to reduce smoking and low birthweight delivery. *British Journal of Obstetrics and Gynaecology* 1991;98(9):859–65. 73. Hajek 2001 *{published data only}*Hajek P, West R, Lee A, Foulds J, Owen L, Eiser JR, et al. Randomized controlled trial of a midwife-delivered brief smoking cessation intervention in pregnancy. *Addiction* 2001;96(3):485–94. 74. Hartmann 1996 *{published data only}*Hartmann K, Thorp J, Pahel-Short L, Koch M. A randomized controlled trial of smoking cessation intervention in pregnancy. *American Journal of Obstetrics and Gynecology* 1995;172:287. 75. Hartmann KE, Koch MA, Pahel-Short L, Thorp JM. A randomized controlled trial of smoking cessation intervention in pregnancy in an academic clinic. *Obstetrics & Gynecology* 1996;87:621–6. 76. Haug 1994 *{published data only}*Haug K, Fugelli P, Aaro LE. Recruitment and participation of General Practitioners in a multipractice study of smoking cessation. *Scandinavian Journal of Primary Health Care* 1992;10(3):206–10. 77. Haug K, Fugelli P, Aaro LE, Foss OP. Is smoking intervention in general practice more successful among pregnant than non-pregnant women?. *Family Practice* 1994; 11:111–6. 78. Haug 2004 *{published data only (unpublished sought but not used)}* Haug NA, DiClemente C, Svikis DS. Motivational enhancement therapy for nicotine dependence in methadone-maintained pregnant women. *Psychology of Addictive Behaviours* 2004;18(3):289–92. 79. Hegaard 2003 *{published data only}* Hegaard H, Hjaergaard H, Moller L, Wachmann H, Ottesen B. Multimodel intervention raises smoking cessation rate during pregnancy. *Acta Obstetricia et Gynecologica Scandinavica* 2003;82:813–9. 80. Hegaard HK, Kjaergaard H, Moller LF, Wachmann H, Ottesen B. Determination of a saliva cotinine cut-off to distinguish pregnant smokers from pregnanct non-smokers. *Acta Obstetricia et Gynecologica* 2007;86:401–6. 81. Hegaard HK, Kjaergaard H, Moller LF, Wachmann H, Ottesen B. Long-term nicotine replacement therapy. *British Journal of Midwifery* 2004;12(4):214–20. 82. Heil 2008 *{published data only}*Bernstein IM, Mongeon JA, Badger GJ, Solomon L, Heil SH, Higgins ST. Maternal smoking and its association with birthweight. *Obstetrics & Gynecology* 2005;106(5 Pt 1): 986–91. 83. Heil SH, Higgins ST. Characterizing nicotine withdrawal and craving in pregnant cigarette smokers. 66th Annual Scientific Meeting of the College on Problems of Drug Dependence; 2004 June 12-17; San Juan, Puerto Rico. 2004. 84. Heil SH, Higgins ST, Bernstein IM, Solomon LJ, Rogers RE, Thomas CS, et al. Effects of voucher-based incentives on abstinence from cigarette smoking and fetal growth among pregnant women. *Addiction* 2008;103(6):1009–18. 85. Heil SH, Higgins ST, Solomon LJ, Lynch ME, McHale L, Dumeer A, et al. Voucher-based incentives for abstinence from cigarette smoking in pregnant and postpartum women. Society for Research on Nicotine and Tobacco 13th Annual Meeting; 2007 Feb 21-24; Austin, Texas. 2007:25, Abstract no: PA6-1. 86. Heil SH, Tidey JW, Holmes HW, Badger GJ, Higgins ST. A contingent payment model of smoking cessation: effects on abstinence and withdrawal. *Nicotine and Tobacco Research* 2003;5(2):205–13. 87. Higgins ST, Bernstein IM, Washio Y, Heil SH, Badger GJ, Skelly JM, et al. Effects of smoking cessation with voucher- based contingency management on birth outcomes. *Addiction* 2010;105:2023–30. 88. Higgins ST, Heil SH, Badger GJ, Mongeon JA, Solomon LJ, McHale L, et al. Biochemical verification of smoking status in pregnant and recently postpartum women. *Experimental and Clinical Psychopharmacology* 2007;15(1):58–66. 89. Higgins ST, Heil SH, Dumeer AM, Thomas CS, Solomon LJ, Bernstein IM. Smoking status in the initial weeks of quitting as a predictor of smoking-cessation outcomes in pregnant women. *Drug and Alcohol Dependence* 2006;85: 138–41. 90. Higgins ST, Heil SH, Solomon LJ, Bernstein IM, Lussier JP, Abel RL, et al. A pilot study on voucher-based incentives to promote abstinence from cigarette smoking during pregnancy and postpartum. *Nicotine and Tobacco Research* 2004;6(6):1015–20. 91. Higgins TM, Higgins ST, Heil SH, Badger GJ, Skelly JM, Bernstein IM, et al. Effects of cigarette smoking cessation on breastfeeding duration. *Nicotine & Tobacco Research* 2010;12(5):483–8. 92. Linares Scott TJ, Heil SH, Higgins ST, Badger GJ, Bernstein IM. Depressive symptoms predict smoking status among pregnant women. *Addictive Behaviours* 2009;34:705–8. 93. Lussier JP, Heil SH, Mongeon JA, Badger GJ, Higgins ST. A meta-analysis of voucher-based reinforcement therapy for substance use disorders. *Addiction* 2006;101:192–203. 94. Solomon LJ, Higgins ST, Heil SH, Badger GJ, Thomas CS, Bernstein IM. Predictors of postpartum relapse to smoking. *Drug and Alcohol Dependence* 2007;90:224–7. 95. Washio Y, Higgins ST, Heil SH, Badger GJ, Skelly J, Bernstein IM, et al. Examining maternal weight gain during contingency-management treatment for smoking cessation among pregnant women. *Drug and Alcohol Dependence* 2011;114(1):73–6. 96. Yoon JH, Higgins ST, Heil SH, Sugarbaker RJ, Thomas CS, Badger GJ. Delay discounting predicts postpartum relapse to cigarette smoking among pregnant women. *Experimental and Clinical Psychopharmacology* 2007;15(2):176–86. 97. Hennrikus 2010 *{published data only}*Hennrikus D, Pirie P, Hellerstedt W, Lando HA, Steele J, Dunn C. Increasing support for smoking cessation during pregnancy and postpartum: results of a randomized controlled pilot study. *Preventive Medicine* 2010;50(3): 134–7. 98. Hiett 2000 *{published data only}*Hiett A, Brazus S, Hedberg J, Brown H. Smoking cessation program effectiveness during pregnancy. *American Journal of Obstetrics and Gynecology* 2000;182(1 Pt 2):S150. 99. Hjalmarson 1991 *{published data only}*∗ Hjalmarson AIM, Svanberg B, Hahn L. Stopping smoking in pregnancy: effect of a self-help manual in a controlled trial. *British Journal of Obstetrics and Gynaecology* 1991;98: 260–4. Svanberg B. Smoking during pregnancy: possibilities of prevention in antenatal care. *International Journal of Technology Assessment in Health Care* 1992;8(Suppl 1): 96–100. 100. Hughes 2000 *{published data only}*∗ Hughes E, Lamont D, Beecroft M, Wilson D. Randomized trial of a “stage-of-change” orientated smoking cessation intervention in infertile and pregnant women. *Fertility and Sterility* 2000;74(3):498–503. 101. Hughes EG, Beecroft ML, Lamont D, Rice S, Wilson D, Freebury M, et al. A randomised controlled trial of a “State- of Change” smoking cessation intervention for subfertile and pregnant patients. *Fertility and Sterility* 1999;72(3 Suppl 1):S61–S62. 102. Kendrick 1995 *{published data only}*England LJ, Kendrick JS, Wilson HG, Merritt RK, Gargiullo PM, Zahniser SC. Effects of smoking reduction during pregnancy on the birth weight of term infants. *American Journal of Epidemiology* 2001;154:694–701. ∗ 103. Kendrick JS, Metzger RW, Sexton M, Spierto FW, Floyd RL, Gargiullo PM, et al. Integrating smoking cessation into routine public prenatal care: the Smoking Cessation in Pregnancy project. *American Journal of Public Health* 1995; 85:217–22. 104. Spierto FW, Hannon WH, Kendrick JS, Bernert JT, Pirkle J, Gargiullo PM. Urinary cotinine levels in women enrolled in a smoking cessation study during and after pregnancy. *Journal of Smoking-Related Disease* 1994;5:65–76. 105. Lawrence 2003 *{published data only}*Aveyard P, Lawrence T, Cheng KK, Griffin C, Croghan E, Johnson C. A randomized controlled trial of smoking cessation for pregnant women to test the effect of a transtheoretical model-based intervention on movement in stage and interaction with baseline stage. *British Journal of Health Psychology* 2006;11:263–78. 106. Aveyard P, Lawrence T, Croghan E, Evans O, Cheng KK. Is advice to stop smoking from a midwife stressful for pregnant women who smoke? Data from a randomized controlled trial. *Preventive Medicine* 2004;40:575–82. 107. Aveyard P, Lawrence T, Evans O, Cheng KK. The influence of in-pregnancy smoking cessation programmes on partner quitting and women’s social support mobilization: a randomized controlled trial. *BMC Public Health* 2005;5:80. Aveyard P, West R. Managing smoking cessation. *BMJ* 2007;335:37–41. 108. Lawrence T, Aveyard P, Cheng KK, Griffin C, Johnson C, Croghan E. Does stage-based smoking advice in pregnancy result in long-term quitters? 18-month post-partum follow up of a randomised controlled trial. Society for Research on Nicotine and Tobacco 11th Annual Meeting; 2005 March 20-23; Prague, Czech Republic. 2005. 109. Lawrence T, Aveyard P, Cheng KK, Griffin C, Johnson C, Croghan E. Does stage-based smoking cessation advice in pregnancy result in long term quitters? 18-month postpartum follow-up of a randomized controlled trial. *Addiction* 2005;100:107–16. 110. Lawrence T, Aveyard P, Croghan E. What happens to women’s self-reported cigarette consumption and urinary cotinine levels in pregnancy?. *Addiction* 2003;98:1315–20. ∗ Lawrence T, Aveyard P, Evans O, Cheng KK. A cluster randomised controlled trial of smoking cessation in pregnant women comparing interventions based on the transtheoretical (stages of change) model to standard care. *Tobacco Control* 2003;12:168–77. 111. LeFevre 1995 *{published data only}*Ewigman B, Crane J, Frigoletto F, LeFevre M, Bain R, McNellis D. Effect of prenatal ultrasound screening on perinatal outcome. *New England Journal of Medicine* 1993; 329:821–9. 112. LeFevre ML, Ewigman B, Evans JK. Is smoking an indication for prenatal ultrasonography? RADIUS Study Group. *Archives of Family Medicine* 1995;4:120–3. 113. Lilley 1986 *{published data only}*Lilley J, Forster DP. A randomised controlled trial of individual counselling of smokers in pregnancy. *Public Health* 1986;100:309–15. 114. Lillington 1995 *{published data only}*Lillington L, Chlebowski R, Ruvalcaba M, Novak D, Royce J. Evaluation of a smoking cessation program for pregnant minority smokers. *Cancer Practice* 1995;3(3):157–63. 115. Loeb 1983 *{published data only}*Bailey JW, Loeb BK, Waage G. A randomized trial of smoking intervention during pregnancy. Proceedings of the American Public Health Association 111th Annual Meeting; 1983 Nov 15; Dallas, Texas, USA. 1983:58. 116. Loeb BK, Waage G, Bailey J. Smoking intervention in pregnancy. Proceedings of the Fifth World Conference on Smoking and Health; 1983 July; Winnipeg, Canada. Ottawa: Canadian Council on Smoking and Health, 1983: 389–95. 117. Lowe 1997 *{published data only}*Lowe JB, Windsor R, Balanda K, Woodby L. Smoking relapse prevention methods for pregnant women: a formative evaluation. *American Journal of Health Promotion* 1997;11:244–6. 118. Lowe 2002 *{published data only}*∗ Lowe JB, Balanda KP, Stanton WR, Del Mar C, O’Connor V. Dissemination of an efficacious antenatal smoking cessation program in public hospitals in Australia: a randomised controlled trial. *Health Education & Behavior* 2002;29(5):608–19. 119. Stanton WR, Lowe JB, Moffatt J, DelMar CB. Randomised control trial of a smoking cessation intervention directed at men whose partners are pregnant. *Preventive Medicine* 2004;38:6–9. 120. Malchodi 2003 *{published data only}*Malchodi CS, Oncken C, Dornelas EA, Caramanica L, Gregonis E. The effects of peer counselling on smoking cessation and reduction. *Obstetrics & Gynecology* 2003;101 (3):504–10. 121. Manfredi 1999 *{published data only}*∗ Manfredi C, Crittenden KS, Warnecke R, Engler J, Cho YI, Shaligram C. Evaluation of a motivational smoking cessation intervention for women in public health clinics. *Preventive Medicine* 1999;28:51–60. 122. Crittenden, KS, Manfredi C, Cho YI, Dolecek TA. Smoking cessation processes in low-SES women: the impact of time- varying pregnancy status, healthcare messages, stress and health concerns. *Addictive Behaviors* 2007;32:1347–66. 123. Manfredi C, Cho YI, Warnecke R, Saunders S, Sullivan M. Dissemination strategies to improve implementation of the PHS smoking cessation guideline in MCH public health clinics: experimental evaluation results and contextual factors. *Health Education Research* 2011;26(2):348–60. 124. Manfredi C, Crittenden KS, Cho YI, Gao S. Long-term effects (up to 18 months) of a smoking cessation program among women smokers in public health clinics. *Preventive Medicine* 2004;38:10–9. 125. Mayer 1990 *{published data only}*Mayer JP, Todd R, Hawkins B. A randomised evaluation of smoking cessation interventions for pregnant women at a WIC clinic. *American Journal of Public Health* 1990;80: 76–7. 126. McBride 1999 *{published data only}*Curry SJ, McBride C, Grothus L, Lando H, Pirie P. Motivation for smoking cessation among pregnant women. *Psychology of Addictive Behaviors* 2001;15(2):126–32. 127. Lando HA, Valanis BG, Lichtenstein E, Curry SJ, McBride CM, Pirie PL, et al. Promoting smoking abstinence in pregnant and postpartum patients: a comparison of 2 approaches. *American Journal of Managed Care* 2001;7: 685–93. 128. McBride CM, Curry SJ, Grothaus LC, Nelson JC. Partner smoking status and pregnant smokers’ perception of support for and likelihood of smoking cessation. *Health Psychology* 1998;17:63–9. ∗ 129. McBride CM, Curry SJ, Lando HA, Pirie PL, Grothaus LC, Nelson JC. Prevention of relapse in women who quit smoking during pregnancy. *American Journal of Public Health* 1999;89:706–11. 130. McBride 2004 *{published data only}*McBride CM, Baucom DH, Peterson BL, Pollak KI, Palmer C, Westman E, et al. Prenatal and postpartum smoking abstinence: a partner assisted approach. *American Journal of Preventive Medicine* 2004;27(3):232–8. 131. McLeod 2004 *{published data only}*McLeod D, Benn C, Pullon S, Viccars A, White S, Cookson T, et al. The midwife’s role in facilitating smoking behaviour change during pregnancy. *Midwifery* 2003;19(4):285–97. 132. McLeod D, Pullon S, Benn C, Cookson T, Dowell A, Viccars A, et al. Can support and education for smoking cessation and reduction be provided effectively by midwives within primary maternity care?. *Midwifery* 2004;20:37–50. Pullon S, McCleod D, Benn C, Viccars A, White S, Cookson T, et al. Smoking cessation in New Zealand: education and resources for use by midwives for women who smoke during pregnancy. *Health Promotion International* 2003;18(4):315–24. 133. Messimer 1989 *{published data only}*Messimer SR, Henry RC, Hickner JM. A comparison of two antismoking interventions among pregnant women in eleven primary care practices. *Journal of Family Practice* 1989;28(3):283–8. 134. Moore 1998 *{published data only}*∗ Moore ML, Meis PJ, Ernest JM, Wells HB, Zaccaro DJ, Terrell T. A randomized trial of nurse intervention to reduce preterm and low birth weight births. *Obstetrics & Gynecology* 1998;91:656–61. 135. Moore ML, Zaccaro DJ. Cigarette smoking, low birth weight, and preterm births in low-income African-American women. *Journal of Perinatology* 2000;3:176–80. 136. Moore 2002 *{published data only}*Moore LO, Campbell R, Whelan A, Mills N, Lupton P, Misselbrook E, et al. Self help smoking cessation in pregnancy: cluster randomised controlled trial. *BMJ* 2002; 325:1383–6. 137. Naughton 2012 *{published data only}*Naughton F, Prevost AT, Gilbert H, Sutton S. Randomized controlled trial evaluation of a tailored leaflet and SMS text message self-help intervention for pregnant smokers (MiQuit). *Nicotine and Tobacco Research* 2012;14(5): 569–77. 138. Olds 1986 *{published data only}*Olds DL, Henderson CR, Chamberlin R, Tatelbaum R. Preventing child abuse and neglect: a randomized trial of nurse home visitation. *Pediatrics* 1986;78:65–78. 139. Olds DL, Henderson CR, Tatelbaum R. Prevention of intellectual impairment in children of women who smoke cigarettes during pregnancy. *Pediatrics* 1994;93:228–33. 140. Olds DL, Henderson CR, Tatelbaum R, Chamberlin R. Improving the delivery of prenatal care and outcomes of pregnancy: a randomized trial of nurse home visitation. *Pediatrics* 1986;77:16–28. 141. Olds 2002 *{published data only}*Olds D, Robinson J, O’Brien R, Luckey D, Pettit L, Henderson C, et al. Home visiting by paraprofessionals and by nurses: a randomized, controlled trial. *Pediatrics* 2002; 110(3):486–96. 142. Ondersma 2012 *{published data only}*Ondersma S, Svikis DS, Beatty JR, Lockhart N. A randomized clinical trial of a computer delivered brief intervention for post-partum drug, alcohol, and tobacco use: three-month outcomes. Proceedings of the 73rd Annual Scientific Meeting of the College on Problems of Drug Dependence; 2011 June 18-23, Hollywood, Florida. 2011; Vol. http://www.cpdd.vcu.edu/Pages/Meetings/ Meetings ̇PDFs/2011Programbook.pdf. Accessed 22/8/ 2013:134 , Abstract no: 534. Ondersma SJ. Computer-assisted Intervention for smoking during pregnancy (HPP). ClinicalTrials.gov 2009. 143. Ondersma SJ, Chase SK, Svikis DS, Schuster CR. Computer-based brief motivational intervention for perinatal drug use. *Journal of Substance Abuse Treatment* 2005;28:305–12. ∗ Ondersma SJ, Svikis DS, Lam PK, Connors-Burge VS, Ledgerwood DM, Hopper JA. A randomized trial of computer-delivered brief intervention and low-intensity contingency management for smoking during pregnancy. Nicotine & Tobacco Research 2012; Vol. 14, issue 3: 351–60. 144. Panjari 1999 *{published data only}*Panjari M, Bell R, Astbury J, Bishop S, Dalais F, Rice G. Women who spontaneously quit smoking in early pregnancy. *Australian New Zealand Journal of Obstetrics and Gynaecology* 1997;37(3):271. ∗ 145. Panjari M, Bell R, Bishop S, Astbury J, Rice G, Doery J. A randomized controlled trial of a smoking cessation intervention during pregnancy. *Australian and New Zealand Journal of Obstetrics and Gynaecology* 1999;39(3):312–7. 146. Parker 2007 *{published data only}*Parker DR, Roberts MB, Windsor RA, Lasater TM. Telephone-based smoking cessation interventions effective in high-risk, underserved pregnant women. Joint Conference of SRNT and SRNT-Europe; 2009 April 27-30 Dublin, Ireland. 2099. 147. ∗ Parker DR, Windsor RA, Roberts MB, Hecht J, Hardy NV, Strolla LO, et al. Feasibility, cost, and cost- effectiveness of a telephone-based motivational intervention for underserved pregnant smokers. *Nicotine and Tobacco Research* 2007;9(10):1043–51. 148. Patten 2009 *{published data only}*Patten CA. Tobacco cessation intervention during pregnancy among Alaska Native women. *Journal of Cancer Education* 2012;27(Supp 1):S86–90. Patten CA. Tobacco cessation treatment for pregnant Alaska natives. ClinicalTrials.gov (http://clinicaltrials.gov/) (accessed 21 June 2007). 149. ∗ Patten CA, Windsor RA, Renner CC, Enoch C, Hochreiter A, Nevak C, et al. Feasibility of a tobacco cessation intervention for pregnant Alaska Native women. *Nicotine & Tobacco Research* 2009;12(2):79–87. 150. Pbert 2004 *{published data only}*Bonollo DP, Zapka JG, Stoddard AM, Ma Y, Pbert L, Ockene JK. Treating nicotine dependence during pregnancy and postpartum: understanding clinician knowledge and performance. *Patient Education and Counselling* 2002;48: 265–74. Ma Y, Goins KV, Pbert L, Ockene JK. Predictors of smoking cessation in pregnancy and maintenance postpartum in low- income women. *Maternal and Child Health Journal* 2005;9 (4):393–402. 151. ∗ Pbert L, Ockene JK, Zapka J, Ma Y, Goins KV, Oncken C, et al. A community health center smoking cessation intervention for pregnant and postpartum women. *American Journal of Preventive Medicine* 2004;26(5): 377–85. Zapka J, Goins KV, Pbert L, Ockene JK. Translating efficacy research into effectiveness studies in practice: lessons from research to promote smoking cessation in community health centers. *Health Promotion Practice* 2004;5(3):245–55. 152. Zapka JG, Pbert L, Stoddard AM, Ockene JK, Goins KV, Bonollo D. Smoking cessation counseling with pregnant and postpartum women: a survey of community health center providers. *American Journal of Public Health* 2000; 90:78–84. 153. Petersen 1992 *{published data only}*Peterson L, Rosen A, Podedworny T, Kotch J, Handel J. Smoking reduction during pregnancy by a program of self- help and clinical support. *Obstetrics & Gynecology* 1992;79: 924–30. 154. Polanska 2004 *{published data only}*Polanska K, Hanke W, Sobala. Characteristic of the smoking habit among pregnant women on the base of the test “Why am I a smoker?” [Charakterystyka nagogu palenia papierosow wsrod kobiet ciezarnych na podstawie testu Dlaczego pale?]. *Przeglad Lekarski* 2005;62(10):1095–8. 155. Polanska K, Hanke W, Sobala W. Smoking relapse one year after delivery among women who quit smoking during pregnancy. *International Journal of Occupational Medicine and Environmental Health* 2005;18(2):159–65. 156. ∗ Polanska K, Hanke W, Sobala W, Lowe JB. Efficacy and effectiveness of the smoking cessation program for pregnant women. *International Journal of Occupational Medicine and Environmental Health* 2004;17(3):369–77. 157. Price 1991 *{published data only}*Price JH, Snyder FF, Roberts SM, Losh DP, Desmond SM, Krol RA. Comparison of three antismoking interventions among pregnant women in an urban setting: a randomized trial. *Psychological Reports* 1991;68:595–604. 158. Reading 1982 *{published data only}*Reading AE, Cox DN. The effects of ultrasound on maternal anxiety. *Journal of Behavioral Medicine* 1982;5(2): 237–47. 159. ∗ Reading AE, Sledmere CM, Cox DNB, Campbell S. Health beliefs and health care behaviour in pregnancy. *Psychological Medicine* 1982;12:379–83. 160. Rigotti 2006 *{published data only}*Berg CJ, Park ER, Chang Y, Rigotti NA. Is concern about post-cessation weight gain a barrier to smoking cessation among pregnant women?. *Nicotine & Tobacco Research* 2008;10(7):1159–63. 161. Park ER, Quinn VP, Chang Y, Regan S, Loudin B, Cummins S, et al. Recruiting pregnant smokers into a clinical trial: using a network-model managed care organization versus community-based practices. *Preventive Medicine* 2007;44: 223–9. Rigotti N, Park E, Regan S, Chang Y, Perry K, Loudin B, et al. Efficacy of proactive telephone counseling for pregnant smokers: a randomized trial. Society for Research on Nicotine and Tobacco 12th Annual Meeting; 2006 February 15-18, Orlando, Florida, USA 2006:22. 162. Rigotti N, Park E, Regan S, Chang Y, Perry K, Loudin B, et al. The efficacy of telephone couseling for pregnant smokers: a randomized controlled trial [abstract]. 13th World Conference on Tobacco or Health; 2006 July 12-15; Washington DC, USA. 2006. 163. ∗ Rigotti N, Park ER, Regan S, Chang Y, Perry K, Loudin B, et al. Efficacy of telephone counseling for pregnant smokers. *Obstetrics & Gynecology* 2006;108(1):83–92. Rigotti NA, Park ER, Chang Y, Regan S. Smoking cessation medication use among pregnant and postpartum smokers. *Obstetrics & Gynecology* 2008;111(2 Pt 1):348–55. 164. Secker-Walker 1994 *{published data only}*∗ Secker-Walker RH, Mead PB, Goodwin GD, Lepage SS, Skelly JM, Flynn BS, et al. Individualised smoking cessation counseling during prenatal and early postnatal care. *American Journal of Obstetrics and Gynecology* 1994; 71:1347–55. 165. Secker-Walker RH, Solomon LJ, Flynn BS, Skelly JM, Lepage SS, Goodwin GD, et al. Smoking relapse prevention counseling during prenatal and early postnatal care. *American Journal of Preventive Medicine* 1995;11(2):86–93. 166. Secker-Walker 1997 *{published data only}*Secker-Walker RH, Solomon LJ, Flynn BS, LePage SS, Crammond JE, Worden JK, et al. Training obstetric and family practice residents to give smoking cessation advice during prenatal care. *American Journal of Obstetrics and Gynecology* 1992;166:1356–63. 167. ∗ Secker-Walker RH, Solomon LJ, Geller BM, Flynn BS, Worden JK, Skelly JM, et al. Modeling smoking cessation: exploring the use of a videotape to help pregnant women quit smoking. *Women & Health* 1997;25:23–35. 168. Secker-Walker 1998 *{published data only}*Secker-Walker RH, Solomon LJ, Flynn BS, LePage SS, Crammond JE, Worden JK, et al. Training obstetric and family practice residents to give smoking cessation advice during prenatal care. *American Journal of Obstetrics and Gynecology* 1992;166:1356–63. 169. ∗ Secker-Walker RH, Solomon LJ, Flynn BS, Skelly JM, Mead PB. Reducing smoking during pregnancy and postpartum: physician’s advice supported by individual counseling. *Preventive Medicine* 1998;27:422–30. 170. Secker-Walker RH, Solomon LJ, Flynn BS, Skelly JM, Mead PB. Smoking relapse prevention during pregnancy. A trial of coordinated advice from physicians and individual counseling. *American Journal of Preventive Medicine* 1998; 15:25–31. 171. Secker-Walker RH, Vacek PM, Flynn BS, Mead PB. Estimated gains in birth weight associated with reductions in smoking during pregnancy. *Journal of Reproductive Medicine* 1998;43(11):967–74. 172. Secker-Walker RH, Vacek PM, Flynn BS, Mead PB. Smoking in pregnancy, exhaled carbon monoxide, and birth weight. *Obstetrics and Gynecology* 1997;89:648–53. 173. Solomon LJ, Secker-Walker RH, Skelly JM, Flynn BS. Stages of change in smoking during pregnancy in low risk women. *Journal of Behavioral Medicine* 1996;19:333–4. 174. Sexton 1984 *{published data only}*Fox NL, Sexton M, Hebel JR, Thompson B. The reliability of self-reports of smoking and alcohol consumption by pregnant women. *Addictive Behaviors* 1989;14(2):187–95. 175. Fox NL, Sexton MJ, Hebel JR. Alcohol consumption among pregnant smokers: effects of a smoking cessation intervention program. *American Journal of Public Health* 1987;77:211–3. 176. Hamilton BH. Estimating treatment effects in randomized clinical trials with non-compliance: the impact of maternal smoking on birthweight. *Health Economics* 2001;10(5): 399–410. 177. Hebel JR, Sexton M, Nowicki P. The effect of antismoking intervention during pregnancy: an assessment of interactions with maternal characteristics. *American Journal of Epidemiology* 1985;12 178. owicki P, Gintzig L, Hebel JR, Lathem R, Miller V, Sexton M. Effective smoking intervention during pregnancy. *Birth* 1984;11:217–24. ∗ Sexton M, Hebel JR. A clinical trial of change in maternal smoking and its effect on birth weight. *JAMA* 1984;251: 911–5. Sexton M, Nowicki P, Hebel JR. Verification of smoking status by thiocyanate in unrefrigerated, mailed saliva samples. *Preventive Medicine* 1986;15(1):28–34. 179. Solomon 2000 *{published data only}*Solomon LJ, Secker-Walker RH, Flynn BS, Skelly JM, Capeless EL. Proactive telephone peer support to help pregnant women stop smoking. *Tobacco Control* 2000;9 Suppl 3:iii72–iii74. 180. Stotts 2002 *{published data only}*Stotts AL, Schmitz JM, Shipley SL, Delaune KA, Grabowski J. Impact of a motivational interviewing intervention on mechanisms of change in low-income pregnant smokers (POS4-32) [abstract]. Society for Research on Nicotine and Tobacco 9th Annual Meeting; 2003 February 19-22; New Orleans, Louisiana. 2003:89. 181. ∗ Stotts S, DiClemente CC, Dolan-Mullen P. One-to-One: a motivational intervention for resistant pregnant smokers. *Addictive Behaviors* 2002;27:275–92. 182. Stotts 2004 *{published data only}*Stotts AL, DeLaune KA, Schmitz JM, Grabowski J. Impact of a motivational intervention on mechanisms of change in low-income pregnant women. *Addictive Behaviors* 2004;29 (8):1649–57. 183. Stotts 2009 *{published data only}*Groff J, Stotts A, Velasquez M, Benjamin-Garner R, Green C, Mastrobattista J. Ultrasound and motivational enhancement for prenatal smoking cessation. Annual Meeting of the Society for Behavioural Medicine; 2005 April 13-16; Boston, MA. 2005. Groff JY. Ultrasound and motivational enhancement for prenatal smoking cessation. ClinicalTrials.gov (http:// clinicaltrials.gov/) (accessed March 2006). 184. ∗ Stotts AL, Groff JY, Velasquez MM, Benjamin-Garner R, Green C, Carbonari JP, et al. Ultrasound feedback and motivational interviewing targeting smoking cessation in the second and third trimesters of pregnancy. *Nicotine & Tobacco Research* 2009;11(8):961–8. 185. Strecher 2000 *{published data only}*Strecher VJ, Bishop KR, Bernhardt J, Thorp JM, Cheuvrout B, Potts P. Quits for keeps: tailored smoking cessation guides for pregnancy and beyond. *Tobacco Control* 2000;9 (Suppl 3):iii78–iii79. 186. Tappin 2000 *{published data only}*∗ Tappin DM, Lumsden MA, McIntyre D, McKay C, Gilmour WH, Webber R, et al. A pilot study to establish a randomized trial methodology to test the efficacy of a behavioural intervention. *Health Education Research* 2000; 15(4):491–502. 187. Tappin DM, Lumsden MA, McKay C, McIntyre D, Gilmour H, Webber R, et al. The effect of home-based motivational interviewing on the smoking behaviour of pregnant women: a pilot randomised controlled efficacy study. *Ambulatory Child Health* 2000;6(Suppl 1):34–5. 188. Tappin 2005 *{published data only}*Tappin DM, Lumsden MA, Gilmour WH, Crawford F, McIntyre D, Stone DH, et al. Randomised controlled trial of home based motivational interviewing by midwives to help pregnant smokers quit or cut down. *BMJ* 2005;331: 373–7. 189. Thornton 1997 *{published data only}*∗ Thornton L. *Smoking and pregnancy: feasibility and effectiveness of a smoking intervention programme among pregnant women [thesis]*. Dublin: Dept of Public Health, 1997. 190. Thornton L, Gogan C, McKenna P. The rotunda stop smoking programme [abstract]. *Irish Journal of Medical Science* 1998;167(Suppl 9):28. 191. Tsoh 2010 *{published data only}*Calderón SH, Gilbert P, Jackson R, Kohn MA, Gerbert B. Cueing prenatal providers: effects on discussions of intimate partner violence. *American Journal of Preventive Medicine* 2008;34(2):134-7. 192. Gilbert P. The health in pregnancy (HIP) study. ClinicalTrials.gov (http://clinicaltrials.gov/) (accessed 20 February 2008) 2008. 193. ∗ Tsoh JY, Kohn MA, Gerbert B. Promoting smoking cessation in pregnancy with Video Doctor plus provider cueing: a randomized trial. *Acta Obstetricia et Gynecologica Scandinavica* 2010;89(4):515–23. 194. Tuten 2012 *{published data only}*Tuten M, Fitzsimons H, Chisolm MS, Nuzzo PA, Jones HE. Contingent incentives reduce cigarette smoking among pregnant, methadone-maintained women: results of an initial feasibility and efficacy randomized clinical trial. *Addiction* 2012;107(10):1868–77. 195. Valbo 1994 *{published data only}*Valbo A, Nylander G. Smoking cessation in pregnancy: intervention among heavy smokers. *Acta Obstetricia et Gynecologica Scandinavica* 1994;73:215–9. 196. Valbo 1996 *{published data only}*Valbo A, Eide T. Smoking cessation in pregnancy: the effect of hypnosis in a randomized study. *Addictive Behaviors* 1996;21:29–35. 197. Vilches 2009 *{published and unpublished data}*Aranda Regules JM, Mateos Vilchez P, Gonzalez Villalba A, Sanchez F, de Dios Luna del Castillo J. Validity of smoking measurements during pregnancy: specificity, sensitivity and cut-off points. *Revista Espanola de Salud Publica* 2008;82 (5):535–45. 198. ∗ Vilches P. *Consumption of tobacco in pregnant women: Proposal of a psychological intervention model in the public health system of Andalucia. [Consumo de tabaco en mujeres gestantes: propuesta de un modelo de intervención psicológica en el sistema sanitario público de Andalucía]. [Doctoral Thesis]*. Univesidad di Malaga, 2009. 199. Walsh 1997 *{published data only}*∗ Walsh RA, Melmeth A, Byrne JM, Brinsmead MW, Redman S. A smoking cessation program at a public antenatal clinic. *American Journal of Public Health* 1997;87: 1201–4. 200. RA, Redman S, Byrne JM, Melmeth A, Brinsmead MW. Process measures in an antenatal smoking cessation trial: another part of the picture. *Health Education Research* 2000;15(4):469–83. 201. Windsor 1985 *{published data only}*Windsor RA. The efficacy and cost-effectiveness of smoking cessation methods for pregnant women. *Southern Medical Journal* 1986;79:34. 202. ∗ Windsor RA, Spanos D, Samuelsson C, Bartlett EE, Manzella B, Reese Y, et al. The effectiveness of smoking cessation methods for smokers in public health maternity clinics: a randomized trial. *American Journal of Public Health* 1985;75:1389–92. 203. Windsor RA, Warner KE, Cutter GR. A cost-effectiveness analysis of self-help smoking cessation methods for pregnant women. *Public Health Reports* 1988;103(1):83–8. 204. Windsor 1993 *{published data only}*Windsor RA, Contreras L, Artz L, Lowe JB. Smoking cessation and pregnancy intervention trial: preliminary mid-trial results. *Progress in Clinical and Biological Research* 1990;339:107–17. 205. Windsor RA, Li CQ, Boyd NR, Hartmann KE. The use of significant reduction rates to evaluate health education methods for pregnant smokers: a new harm reduction behavioural indicator. *Health Education and Behavior* 1999; 26:648–61. 206. ∗ Windsor RA, Lowe JB, Perkins LL, Smith-Yoder D, Artz L, Crawford M, et al. Health education for pregnant smokers: its behavioral impact and cost benefit. *American Journal of Public Health* 1993;83:201–6. 207. Windsor 2011 *{published data only}*Windsor R, Woodby L, Miller T, Hardin M. Effectiveness of Smoking Cessation and Reduction in Pregnancy Treatment (SCRIPT) methods in Medicaid-supported prenatal care: Trial III. *Health Education & Behavior* 2011;38(4):412–22. |
| Filion  *et al.* 2011[7] | 1. Malchodi CS, Oncken C, Dornelas EA, Caramanica L, Gregonis E,  Curry SL. The effects of peer counseling on smoking cessation and reduction. Obstet Gynecol 2003;101:504–10. 2. Secker-Walker RH, Solomon LJ, Flynn BS, Skelly JM, Lepage SS,  Goodwin GD, et al. Individualized smoking cessation counseling during prenatal and early postnatal care. Am J Obstet Gynecol 1994;171:1347–55. 3. Secker-Walker RH, Solomon LJ, Flynn BS, Skelly JM, Mead PB. Reduc- ing smoking during pregnancy and postpartum: physician’s advice supported by individual counseling. Prev Med 1998;27:422–30. 4. Tappin DM, Lumsden MA, Gilmour WH, Crawford F, McIntyre D, Stone DH, et al. Randomised controlled trial of home based motiva- tional interviewing by midwives to help pregnant smokers quit or cut down. BMJ 2005;331:373–7. 5. Tappin DM, Lumsden MA, McIntyre D, Mckay C, Gilmour WH, Web- ber R, et al. A pilot study to establish a randomized trial methodol- ogy to test the efficacy of a behavioural intervention. Health Educ Res 2000;15:491–502. 6. Ruger JP, Weinstein MC, Hammond SK, Kearney MH, Emmons KM. Cost-effectiveness of motivational interviewing for smoking cessation and relapse prevention among low-income pregnant women: a randomized controlled trial. Value Health 2008;11:191– 8. 7. Rigotti NA, Park ER, Regan S, Chang Y, Perry K, Loudin B, et al. Effi- cacy of telephone counseling for pregnant smokers: a randomized controlled trial. Obstet Gynecol 2006;108:83–92. 8. Bullock L, Everett KD, Mullen PD, Geden E, Longo DR, Madsen R. Baby BEEP: a randomized controlled trial of nurses’ individualized social support for poor rural pregnant smokers. Matern Child Health J 2009;13:395–406. |
| Hemsing  *et al.* 2012[8] | 1. Aveyard , P. , Lawrence , T. , Evans , O. , & Cheng , K. K. ( 2005 ). The infl uence of in-pregnancy smoking cessation programmes on partner quitting and women’s social support mobilization: A randomized controlled trial [ISRCTN89131885] . BMC Public Health , 5 , 80 – 88 . doi:10.1186/1471-2458-5-80 2. Campion , P. , Owen , L. , McNeill , A. , & McGuire , C. ( 1994 ). Evaluation of a mass media campaign on smoking and pregnancy . Addiction , 89 , 1245 – 1254 . doi:10.1111/j.1360- 0443.1994.tb03303.x 3. de Vries , H. , Bakker , M. , Mullen , P. D. , & van Breukelen , G. ( 2006 ). The effects of smoking cessation counseling by midwives on Dutch pregnant women and their partners . Patient Education and Counseling , 63 , 177 – 188 . doi:10.1016/j.pec.2005.10.002 4. McBride , C. M. , Baucom , D. H. , Peterson , B. L. , Pollak , K. I. , Palmer , C. , Westman , E. , et al. ( 2004 ). Prenatal and postpartum smoking abstinence a partner-assisted approach . American Journal of Preventive Medicine , 27 , 232 – 238 . doi:10.1016/j.amepre. 2004.06.005 |
| Hettema  *et al.* 2010[9] | 1. Ershoff, D. H., Quinn, V. P., Boyd, N. R., Stern, J., Gregory, M., & Wirtschafter, D. (1999). The Kaiser Permanente prenatal smoking- cessation trial: When more isn’t better, what is enough? *American Journal of Preventive Medicine, 17,*161–168. 2. Rigotti, N. A., Park, E. R., Regan, S., Chang, Y., Perry, K., Loudin, B., & Quinn, V. (2006). Efficacy of telephone counseling for pregnant smokers: A randomized controlled trial. *Obstetrics and Gynecology, 108,* 83–92. 3. Ruger, J. P., Weinstein, M. C., Hammond, S. K., Kearney, M. H., & Emmons, K. M. (2008). Cost-effectiveness of motivational interviewing for smoking cessation and relapse prevention among low-income pregnant women: A randomized controlled trial. *Value in Health, 11,* 191–198. 4. Stotts, A. L., DeLaune, K. A., Schmitz, J. M., & Grabowski, J. (2004). Impact of a motivational intervention on mechanisms of change in low-income pregnant smokers. *Addictive Behaviors, 29,* 1649 –1657. 5. Stotts, A. L., Diclemente, C. C., & Dolan-Mullen, P. (2002). One-to-one: A motivational intervention for resistant pregnant smokers. *Addictive Behaviors, 27,* 275–292. 6. Suplee, P. D. (2005). The importance of providing smoking relapse counseling during the postpartum hospitalization. *Journal of Obstetrics and Gynecology in Neonatal Nursing, 34,* 703–712 7. Tappin, D. M., Lumsden, M. A., Gilmour, W. H., Crawford, F., McIntyre, D., Stone, D. H., . . . Mohammed, E. (2005). Randomised controlled trial of home based motivational interviewing by midwives to help pregnant smokers quit or cut down. *British Medical Journal, 13,* 373–377. 8. Tappin, D. M., Lumsden, M. A., McIntyre, D., Mckay, C., Gilmour, W. H., Webber, R., . . . Currie, F. (2000). A pilot study to establish a randomized trial methodology to test the efficacy of a behavioral intervention. *Health Education Research, 15,* 491–502 |
| Kintz  *et al.* 2014[10] | 1. Edwards, M. J., Geiser, T., Chafin, C., Weatherby, N. L., & Smith, C. S.M.A.R.T. mothers are resisting tobacco: Prenatal smoking cessation in WIC mothers. Journal of Allied Heatlh. 2009; 38(3): 170-176. Available from: MEDLINE database PMid:19753429 2. Dornelas, E. A., Magnavita, J., Beazoglou, T., Fischer, E. H., Oncken, C., Lando, H., et al. Efficacy and cost-effectiveness of a clinic-based counseling intervention tested in an ethnically diverse sample of pregnant smokers. Patient Education and Counseling. 2006; 64(1-3): 342-349. PMid:16859864 <http://dx.doi.org/10.1016/j.pec.2006.03.015> 3. Malchodi, J. B., Filho, P. V., Peterson, G. O., & Chatkin, J. M. Quantitative effects of tobacco smoking exposure on the maternal-fetal circulation. Biomed Central. 2011; 11(24): 1-6. 4. Kataray, G., Kublay, G., & Emiroglu, O. N. Effect of motivational interviewing on smoking cessation in pregnant women. Journal of Advanced Nursing. 2010; 66(6): 1328-1337. http://dx.doi.org/10.1111/j.1365-2648.2010.05267.x 5. Lawrence, T., Aveyard, P., Evans, O., & Cheng, K. K. A cluster randomized controlled trial of smoking cessation in pregnant women comparing interventions based on the transtheoretical (stages of change) model to standard care. Tobacco Control. 2003; 12(2): 168-177. PMid:12773727 http://dx.doi.org/10.1136/tc.12.2.168 6. Albrecht, S. A., Caruthers, D., Patrick, T., Reynolds, M., Salamie, D., Higgins, L. W., et al. A randomized controlled trial of a smoking cessation intervention for pregnant adolescents. Nursing Research. 2006; 55(6): 402-410. PMid:17133147 http://dx.doi.org/10.1097/00006199-200611000-00004 7. Peden, A. R., Rayens, M. K., Hall, L. A., Hahn, E., Riker, C., Ashford, K., et al. Nicotine addiction in pregnancy: Preliminary efficacy of a mental health intervention. Addictive Disorders & Their Treatment. 2008; 7(4): 179-189. http://dx.doi.org/10.1097/ADT.0b013e3181484768 8. Vries, H., Bakker, M., Mullen, P. D., & Van Breukelen, G. The effects of smoking cessation counseling by midwives on dutch pregnant women and their partners. Patient Education and Counseling. 2005; 63(1-2): 177-187. PMid:16406475 <http://dx.doi.org/10.1016/j.pec.2005.10.002> 9. Bullock, L., Everett, K. D., Dolan Mullen, P., Geden, E., Longo, D. R., & Madsen, R. Baby BEEP: A randomized controlled trial of nurses’ individualized social support for poor rural pregnant smokers. Maternal & Child Health Journal. 2009; 13(3): 395-406.PMid:18496746 http://dx.doi.org/10.1007/s10995-008-0363-z 10. Ferreira-Borges, C. Effectiveness of a brief counseling and behavioral intervention for smoking cessation in pregnant women. Preventive Medicine. 2005; 41(1): 295-302. PMid:15917025 http://dx.doi.org/10.1016/j.ypmed.2004.11.013 11. Heil, S. H., Stephen, H. T., Bernstein, I. M., Solomon, L. J., Rogers, R. E., Thomas, C. S., et al. Effects of voucher-based incentives on abstinence from cigarette smoking and fetal growth among pregnant women. Addiction. 2008; 103(6): 1009-1018.PMid:18482424 http://dx.doi.org/10.1111/j.1360-0443.2008.02237.x 12. Hennrikus, D., Pirie, P., Hellerstedt, W., Lando, H. A., Steele, J., & Dunn, C. Increasing support for smoking cessation during pregnancy and postpartum: Results of a randomized controlled pilot study. Preventive Medicine. 2010; 50(3): 134-137.PMid:20079760 http://dx.doi.org/10.1016/j.ypmed.2010.01.003 13. Ondersma, S.J., Svikis, D. S., Lam, P.K., Connors-Burge, V.S., Ledgerwood, D.M., & Hopper, J.A. A randomized trial of computer-delivered brief intervention and low-intensity contingency management for smoking during pregnancy. Nicotine & tobacco research. 2011; (14)3: 351-360. 14. Patten, C. A., Windsor, R. A., Renner, C. C., Enoch, C., Hochreiter, A., Nevak, C., et al. Feasibility of a tobacco cessation intervention for pregnant Alaska native women. Nicotine & Tobacco Research. 2010; 12(2): 79-87. PMid:20018946 http://dx.doi.org/10.1093/ntr/ntp180 15. Bryce, A., Butler, C., Gnich, W., Sheehy, C., & Tappin, D. CATCH: development of a home-based midwifery intervention to support young pregnant smokers to quit. Midwifery. 2009; 25(5): 473-482. PMid:18280015 http://dx.doi.org/10.1016/j.midw.2007.10.006 16. Campbell, E., Walsh, R., Sanson-Fisher, R., Burrows, S., & Stojanovski, E. A group randomised trial of two methods for disseminating a smoking cessation programme to public antenatal clinics: effects on patient outcomes. Tobacco Control. 2006; 15(20): 97-102. PMid:16565456 http://dx.doi.org/10.1136/tc.2004.011049 17. Cope, G. F., Nayyar, P., & Holder, R. Feedback from a point-of-care test for nicotine intake to reduce smoking during pregnancy. The Association of Clinical Biochemists. 2003; 40(6): 674-679. 18. Jaakkola, N., Zahlsen, K., & Jaakkola, J. K. Effects of a population-based smoking cessation programme on smoking in pregnancy. European Journal of Public Health. 2001; 11(4): 446-449. PMid:11766488 http://dx.doi.org/10.1093/eurpub/11.4.446 19. Lando, H. A., Valanis, B. G., Lichtenstein, E., Curry, S. J., McBride, C. M., Pirie, P. L., et al. Promoting smoking abstinence in pregnant and postpartum patients: A comparison of 2 approaches. The American Journal of Managed Care. 2001; 7(7): 685-693. PMid:11464427 20. McGowan, A., Hamilton, S., Barnett, D., Nsofor, M., Proudfoot, J., & Tappin, D. 'Breathe': the stop smoking service for pregnant women in Glasgow. Midwifery. 2010; 26(3): 1-13. PMid:18692285 http://dx.doi.org/10.1016/j.midw.2008.05.005 21. McLeod, D, Pullon, S, Benn, C, Cookson, T, Dowell, A, Viccars, A, et al. Can support and education for smoking cessation and reduction be provided effectively by midwives within primary maternity care? Midwifery. 2013; 20(10): 37-50. 22. Oien, T., Storro, O., Jenssen, J. A., Johnsen, R. The impact of a minimal smoking cessation intervention for pregnant women and their partners on a perinatal smoking behavior in primary health care: A real-life controlled study. BMC Public Health. 2008; 8: 325-335. PMid:18808705 http://dx.doi.org/10.1186/1471-2458-8-325 23. Hajek, P., West, R., Lee, A., Foulds, J., Owen, L., Eiser, J. R., et al. Randomized controlled trial of a midwife-delivered brief smoking cessation intervention in pregnancy. Addiction. 2001; 96(3): 485-494. PMid:11255587 <http://dx.doi.org/10.1046/j.1360-0443.2001.96348511.x> 24. Moore, L., Campbell, R., Whelan, A., Mills, N., Lupton, P., Misselbrook, E., et al. Self help smoking cessation in pregnancy: cluster randomized controlled trial. BMJ. 2002; 325(7377): 1383. PMid:12480850 http://dx.doi.org/10.1136/bmj.325.7377.1383 |
| Naughton  *et al.* 2008[11] | 1. Gielen A. C., Windsor R., Faden R. R., O’Campo P., Repke J., Davis M. Evaluation of a smoking cessation intervention for pregnant women in an urban prenatal clinic. Health Educ Res 1997; 12: 247–54. 2. Windsor R. A., Cutter G., Morris J., Reese Y., Manzella B., Bartlett E. E. et al. The effectiveness of smoking cessation methods for smokers in public health maternity clinics: a randomized trial. Am J Public Health 1985; 75: 1389–92. 3. Windsor R. A., Lowe J. B., Perkins L. L., Smith-Yoder D., Artz L., Crawford M. et al. Health education for pregnant smokers: its behavioral impact and cost benefit. Am J Public Health 1993; 83: 201–6. 4. Altman D., Ashby D., Birks J., Borenstein M., Campbell M., 578 Felix 5. Cinciripini P. M., McClure J. B., Wetter D. W., Perry J., Blalock J. A., Cinciripini L. G. et al. An evaluation of videotaped vignettes for smoking cessation and relapse prevention during pregnancy: the very important pregnant smokers (VIPS) program. Tob Control 2000; 9: III61–3. 6. Valbo A., Schioldborg P. Smoking cessation in pregnancy: the effect of self-help manuals. J Matern Fetal Invest 1994; 4: 167–70. 7. Ershoff D. H., Mullen P. D., Quinn V. P. A randomized trial of a serialized self-help smoking cessation program for pregnant women in an HMO. Am J Public Health 1989; 79: 182–7. 8. Hjalmarson A. I., Hahn L., Svanberg B. Stopping smoking in pregnancy: effect of a self-help manual in controlled trial. Br J Obstet Gynaecol 1991; 98: 260–4. 9. Lowe J. B., Balanda K. P., Clare G. Evaluation of antenatal smoking cessation programs for pregnant women. Aust NZ J Public Health 1998; 22: 55–9. 10. Price J. H., Krol R. A., Desmond S. M., Losh D. P., Roberts S. M., Snyder F. F. Comparison of three antismoking interventions among pregnant women in an urban setting: a randomized trial. Psychol Rep 1991; 68: 595–604. 11. Secker-Walker R. H., Solomon L. J., Geller B. M., Flynn B. S., Worden J. K., Skelly J. M. et al. Modeling smoking cessation: exploring the use of a videotape to help pregnant women quit smoking. Women Health 1997; 25: 23–35. 12. Petersen L., Handel J., Kotch J., Podedworny T., Rosen A. Smoking reduction during pregnancy by a program of selfhelp and clinical support. Obstet Gynecol 1992; 79: 924–30. 13. Ershoff D. H., Quinn V. P., Boyd N. R., Stern J., Gregory M., Wirtschafter D. The Kaiser Permanente prenatal smokingcessation trial: when more isn’t better, what is enough? Am J Prev Med 1999; 17: 161–8. 14. Lawrence T., Aveyard P., Evans O., Cheng K. K. A cluster randomised controlled trial of smoking cessation in pregnant women comparing interventions based on the transtheoretical (stages of change) model to standard care. Tob Control 2003; 12: 168–77. 15. Moore L., Campbell R., Whelan A., Mills N., Lupton P., Misselbrook E. et al. Self help smoking cessation in pregnancy: cluster randomised controlled trial. BMJ 2002; 325: 1383. |
| Su  *et al.* 2014[12] | 1. Higgins, S. T., Heil, S. H., Solomon, L. J., et al. (2004). A pilot study on voucher-based incentives to promote abstinence from cigarette smoking during pregnancy and postpartum. Nicotine & Tobacco Research, 6(6), 1015–1020. 2. Gadomski, A., Adams, L., Tallman, N., Krupa, N., & Jenkins, P. (2011). Effectiveness of a combined prenatal and postpartum smoking cessation program. Maternal and Child Health Journal, 15(2), 188–197 3. Peden, A. R., Rayens, M. K., Hall, L. A., et al. (2008). Nicotine addiction in pregnancy: Preliminary efficacy of a mental health intervention. Addictive Disorders and Their Treatment, 7(4), 179–189. 4. Valanis, B., Lichtenstein, E., Mullooly, J. P., et al. (2001). Maternal smoking cessation and relapse prevention during health care visits. American Journal of Preventive Medicine, 20(1), 1–8. 5. Pollak, K. I., Oncken, C. A., Lipkus, I. M., et al. (2007). Nicotine replacement and behavioral therapy for smoking cessation in pregnancy. American Journal of Preventive Medicine, 33(4), 297–305. 6. Wisborg, K., Henriksen, T. B., Jespersen, L. B., & Secher, N. J. (2000). Nicotine patches for pregnant smokers: A randomized controlled study. Obstetrics and Gynecology, 96(6), 967–971. 7. Albrecht, S. A., Caruthers, D., Patrick, T., et al. (2006). A randomized controlled trial of a smoking cessation intervention for pregnant adolescents. Nursing Research, 55(6), 402–410. 8. McBride, C. M., Baucom, D. H., Peterson, B. L., et al. (2004). Prenatal and postpartum smoking abstinence a partner-assisted approach. American Journal of Preventive Medicine, 27(3), 232–238. 9. Hennrikus, D., Pirie, P., Hellerstedt, W., Lando, H. A., Steele, J., & Dunn, C. (2010). Increasing support for smoking cessation during pregnancy and postpartum: Results of a randomized controlled pilot study. Preventive Medicine, 50(3), 134–137. 10. Lillington, L., Royce, J., Novak, D., Ruvalcaba, M., & Chlebowski, R. (1995). Evaluation of a smoking cessation program for pregnant minority women. Cancer Practice, 3(3), 157–163. 11. Pbert, L., Ockene, J. K., Zapka, J., et al. (2004). A community health center smoking-cessation intervention for pregnant and postpartum women. American Journal of Preventive Medicine, 26(5), 377–385. 12. de Vries, H., Bakker, M., Mullen, P. D., & van Breukelen, G. (2006). The effects of smoking cessation counseling by midwives on Dutch pregnant women and their partners. Patient Education and Counseling, 63(1–2), 177–187. 13. O’Connor, A. M., Davies, B. L., Dulberg, C. S., et al. (1992). Effectiveness of a pregnancy smoking cessation program. Journal of Obstetric, Gynecologic, and Neonatal Nursing, 21(5), 385–392. 14. Polan´ska, K., Hanke, W., & Sobala, W. (2005). Smoking relapse one year after delivery among women who quit smoking during pregnancy. International Journal of Occupational Medicine and Environmental Health, 18(2), 159–165. 15. Secker-Walker, R. H., Solomon, L. J., Flynn, B. S., et al. (1994). Individualized smoking cessation counseling during prenatal and early postnatal care. American Journal of Obstetrics and Gynecology, 171(5), 1347–1355. 16. Walsh, R. A., Redman, S., Brinsmead, M. W., Byrne, J. M., & Melmeth, A. (1997). A smoking cessation program at a public antenatal clinic. American Journal of Public Health, 87(7), 1201–1204. 17. Wall, M. A., Severson, H. H., Andrews, J. A., Lichtenstein, E., & Zoref, L. (1995). Pediatric office-based smoking intervention: Impact on maternal smoking and relapse. Pediatrics, 96(4 Pt 1), 622–628. 18. Lawrence, T., Aveyard, P., Cheng, K. K., Griffin, C., Johnson, C., & Croghan, E. (2005). Does stage-based smoking cessation advice in pregnancy result in long-term quitters? 18-month postpartum follow-up of a randomized controlled trial. Addiction, 100(1), 107–116. 19. McBride, C. M., Curry, S. J., Lando, H. A., Pirie, P. L., Grothaus, L. C., & Nelson, J. C. (1999). Prevention of relapse in women who quit smoking during pregnancy. American Journal of Public Health, 89(5), 706–711. 20. Secker-Walker, R. H., Solomon, L. J., Flynn, B. S., Skelly, J. M., & Mead, P. B. (1998). Reducing smoking during pregnancy and postpartum: Physician’s advice supported by individual counseling. Preventive Medicine, 27(3), 422–430. 21. Bullock, L., Everett, K. D., Mullen, P. D., Geden, E., Longo, D. R., Madsen, R., et al. (2009). A randomized controlled trial of nurses’ individualized social support for poor rural pregnant smokers. Maternal and Child Health Journal, 13(3), 395–406. 22. Morasco, B. J., Dornelas, E. A., Fischer, E. H., Oncken, C., & Lando, H. A. (2006). Spontaneous smoking cessation during pregnancy among ethnic minority women: A preliminary investigation. Addictive Behaviors, 31(2), 203–210. 23. Reitzel, L. R., Vidrine, J. I., Businelle, M. S., et al. (2010). Preventing postpartum smoking relapse among diverse lowincome women: A randomized clinical trial. Nicotine & Tobacco Research, 12(4), 326–335. 24. Rigotti, N. A., Park, E. R., Regan, S., et al. (2006). Efficacy of telephone counseling for pregnant smokers: A randomized controlled 25. trial. Obstetrics and Gynecology, 108(1), 83–92. 26. Mullen, P. D., Quinn, V. P., & Ershoff, D. H. (1990). Maintenance of nonsmoking postpartum by women who stopped 27. smoking during pregnancy. American Journal of Public Health, 80(8), 992–994. 28. Donatelle, R. J., Prows, S. L., Champeau, D., & Hudson, D. (2000). Randomised controlled trial using social support and financial incentives for high risk pregnant smokers: Significant other supporter (SOS) program. Tobacco Control, 9(Suppl 3), III67–III69. 29. Heil, S. H., Higgins, S. T., Bernstein, I. M., et al. (2008). Effects of voucher-based incentives on abstinence from cigarette smoking and fetal growth among pregnant women. Addiction, 103(6), 1009–1018. 30. Cinciripini, P. M., Blalock, J. A., Minnix, J. A., et al. (2010). Effects of an intensive depression-focused intervention for smoking cessation in pregnancy. Journal of Consulting and Clinical Psychology, 78(1), 44–54. 31. Stotts, A. L., Diclemente, C. C., & Dolan-Mullen, P. (2002). Oneto- one: A motivational intervention for resistant pregnant smokers. Addictive Behaviors, 27(2), 275–292. 32. Petersen, L., Handel, J., Kotch, J., Podedworny, T., & Rosen, A. (1992). Smoking reduction during pregnancy by a program of self-help and clinical support. Obstetrics and Gynecology, 79(6), 924–930 33. Brandon, T. H., Simmons, V. N., Meade, C. D., et al. (2012). Self-help booklets for preventing postpartum smoking relapse: A randomized trial. American Journal of Public Health, 102(11), 2109–2115. 34. El-Mohandes, A. A., El-Khorazaty, M. N., Kiely, M., & Gantz, M. G. (2011). Smoking cessation and relapse among pregnant African-American smokers in Washington, DC. Maternal and Child Health Journal, 15(Suppl 1), S96–S105. |
| Washio  *et al.* 2016[13] | 1. Cinciripini PM, Blalock JA, Minnix JA, Robinson JD, Brown VL, Lam C, Karam-Hage M. Effects of an intensive depression-focused intervention for smoking cessation in pregnancy. Journal of Consulting and Clinical Psychology. 2010; 78(1):44–54.10.1037/a0018168. 2. El-Mohandes AA, Windsor R, Tan S, Perry DC, Gantz MG, Kiely M. A randomized clinical trial of trans-dermal nicotine replacement in pregnant African-American smokers. Maternal and Child Health Journal. 2012; 17(5):897–906.10.1007/s10995-012-1069-9 [ 3. Gielen AC, Windsor R, Faden RR, O’Campo P, Repke J, Davis M. Evaluation of a smoking cessation intervention for pregnant women in an urban prenatal clinic. Health Education Research. 1997; 12(2):247–254. http://dx.doi.org/10.1093/her/12.2.247. [ 4. Lillington L, Royce J, Novak D, Ruvalcaba M, Chlebowski R. Evaluation of a smoking cessation program for pregnant minority women. Cancer Practice. 1995; 3(3):157–163. 5. Ondersma SJ, Svikis DS, Lam PK, Connors-Burge VS, Ledgerwood DM, Hopper JA. A randomized trial of computer-delivered brief intervention and low-intensity contingency management for smoking during pregnancy. Nicotine & Tobacco Research. 2012; 14(3):351–360.10.1093/ntr/ntr221 6. Windsor RA, Lowe JB, Perkins LL, Smith-Yoder D, Artz L, Crawford M, Boyd NR. Health education for pregnant smokers: Its behavioral impact and cost benefit. American Journal of Public Health. 1993; 83(2):201–206.10.2105/AJPH.83.2.201 7. Dornelas EA, Magnavita J, Beazoglou T, Fischer EH, Oncken C, Lando H, Gregonis E. Efficacy and cost-effectiveness of a clinic-based counseling intervention tested in an ethnically diverse sample of pregnant smokers. Patient Education and Counseling. 2006; 64(1–3):342–349.10.1016/j.pec.2006.03.015 8. Malchodi CS, Oncken C, Dornelas EA, Caramanica L, Gregonis E, Curry SL. The effects of peer counseling on smoking cessation and reduction. Obstetrics and Gynecology. 2003; 101(3):504–510.10.1016/s0029-7844(02)03070-3 9. Patten CA, Windsor RA, Renner CC, Enoch C, Hochreiter A, Nevak C, Brockman T. Feasibility of a tobacco cessation intervention for pregnant Alaska Native women. Nicotine & Tobacco Research. 2010; 12(2):79–87.10.1093/ntr/ntp180 |
| Chamberlain *et al.* 2017[14] | 1. Albrecht S, Cassidy B, Salamie D, Reynolds M. What’s happening A smoking cessation intervention for pregnant adolescents: implications for nurse practitioners. Journal of American Academy of Nurse Practitioners 199911(4):155–9 2. Albrecht S, Stone CA, Payne L, Reynolds MD. A preliminary study of the use of peer support in smoking cessation programs for pregnant adolescents. Journal of the American Academy of Nurse Practitioners 199810:119–25 3. Albrecht SA, Caruthers D, Patrick T, Reynolds M, Salamie D, Higgins LW, et al. A randomised controlled trial of a smoking cessation intervention for pregnant adolescents. Nursing Research 200655(6):402–10 4. Baric L, MacArthur C, Sherwood M. A study of health education aspects of smoking in pregnancy. International Journal of Health Education 197619(2 Suppl):1–17 5. Bauman 1983 Bauman KE, Koch GG, Dent CW, Bryan ES. The influence of observing carbon monoxide level on cigarette smoking by public prenatal patients. American Journal of Public Health 198373:1089–91 6. Belizan JM, Villar J, Victora C, Farnot U, Langer A, Barros F. Impact of health education during pregnancy on behavior and utilization of health resources. American Journal of Obstetrics and Gynecology 1995173:894–9 7. Bullock L, Everett KD, Mullen PD, Geden E, Longo DR, Madsen R. Baby BEEP: A randomized controlled trial of nurses’ individualized social support for poor rural pregnant smokers. Maternal and Child Health Journal 200913(3): 395–406 8. Bullock LF, Everett KD, Mullen PD. Baby beep: a randomized clinical trial of smoking cessation for lowincome rural pregnant women using nurse-delivered social support. Annals of Behavioral Medicine 200835:S99 9. Bullock LF, Hornblow AR, Duff GB, Wells JE. Telephone support for pregnant women: outcome in late pregnancy. New Zealand Medical Journal 1995108:476–8 10. Burling TA, Bigelow GE, Robinson JC, Mead AM. Smoking during pregnancy: reduction via objective assessment and directive advice. Behavior Therapy 199122:31–40 11. Byrd JC, Meade CD. Smoking cessation among pregnant women in an urban setting. Wisconsin Medical Journal 199392:609–12 12. Campbell E, Walsh RA, Sanson-Fisher R, Burrows S, Stojanovski E. A group randomised trial of two methods for disseminating a smoking cessation programme to public antenatal clinics: effects on patient outcomes. Tobacco Control 200615(2):97–102 13. Cinciripini 2000 Blalock JA, Fouladi RT, Wetter DW, Cinciripini PM. Depression in pregnant women seeking smoking cessation treatment. Addictive Behaviours 200530(6):1195–208 14. Cinciripini PM, Blalock JA, Minnix JA, Robinson JD, Brown VL, Lam C, et al. Effects of an intensive depression focused intervention for smoking cessation in pregnancy. Journal of Consulting & Clinical Psychology 201078(1): 44–54 15. Cook C, Ward S, Myers S, Spinnato J. A prospective, randomized evaluation of intensified therapy for smoking reduction in pregnancy. American Journal of Obstetrics and Gynecology 1995172:290 16. Cope GF, Nayyar P, Holder R. Feedback from a point of-care test for nicotine intake to reduce smoking during pregnancy. Annals of Clinical Biochemistry 2003.40(Pt 6): 674–9 17. Donatelle RJ, Prows SL, Champeau D, Hudson D. Randomised controlled trial using social support and financial incentives for high risk pregnant smokers: Significant Other Supporter (SOS) program. Tobacco Control 2000.9 Suppl 3:iii67–iii69 18. Donovan J. Randomised controlled trial of anti-smoking advice in pregnancy. British Journal of Preventive and Social Medicine 1977.31(1):6–12 19. Dornelas EA, Magnavita J, Beazoglou T, Fischer EH, Oncken C, Lando H, et al. Efficacy and cost-effectiveness of a clinic-based counseling intervention tested in an ethnically diverse sample of pregnant smokers. Patient Education and Counseling 2006.64(1-3):342–9 20. Dunkley J. Training midwives to help pregnant women stop smoking Nursing Times 1997.93(5):64–6 21. Eades SJ, Sanson-Fisher RW, Wenitong M, Panaretto K, D’Este C, Gilligan C, et al. An intensive smoking intervention for pregnant Aboriginal and Torres Strait Islander women: a randomised controlled trial. Medical Journal of Australia 2012.197(1):42–6 22. El-Mohandes AA, El-Khorazaty MN, Kiely M, Gantz MG. Smoking cessation and relapse among pregnant African-American smokers in Washington, DC. Maternal & Child Health Journal 2011.15 Suppl 1:S96–S105 23. Ershoff DH, Quinn VP, Boyd NR, Stern J, Gregory M, Wirtschafter D. The Kaiser Permanente prenatal smoking cessation trial. When more isn’t better, what is enough? American Journal of Preventive Medicine 1999.17(3):161–8 24. Ershoff DH, Quinn VP, Mullen PD. A randomized trial of a serialized self-help smoking cessation program for pregnant women in an HMO. American Journal of Public Health 1989.79(2):182–7 25. Gielen AC, Windsor R, Faden RR, O’Campo P, Repke J, Davis M. Evaluation of a smoking cessation intervention for pregnant women in an urban prenatal clinic. Health Education Research 1997.12(2):247–54 26. Glover M, Kira A, Walker N, Bauld L. Using incentives to encourage smoking abstinence among pregnant indigenous women? A feasibility study. Maternal and Child Health Journal 2014.19(6):1393–9 27. Graham AV, Reeb KG, Kitson GC, Zyzanski SJ, Frank SH. A clinical trial to reduce the rate of low birth weight in an inner-city black population. Family Medicine 1992.24: 439–46 28. Haddow JE, Wald NJ, Palomaki GE, Kloza EM, Knight GJ. Cotinine-assisted intervention in pregnancy to reduce smoking and low birthweight delivery. British Journal of Obstetrics and Gynaecology 1991.98(9):859–65 29. Hajek P, West R, Lee A, Foulds J, Owen L, Eiser JR, et al. Randomized controlled trial of a midwife-delivered brief smoking cessation intervention in pregnancy. Addiction 2001.96(3):485–94 30. Harris M, Reynolds B. A pilot study of home-based smoking cessation programs for rural, Appalachian, pregnant smokers. Journal of Obstetric, Gynecologic, and Neonatal Nursing 2015 [EPub ahead of print] 31. Hartmann KE, Koch MA, Pahel-Short L, Thorp JM. A randomized controlled trial of smoking cessation intervention in pregnancy in an academic clinic. Obstetrics & Gynecology 1996.87:621–6 32. Haug K, Fugelli P, Aaro LE, Foss OP. Is smoking intervention in general practice more successful among pregnant than non-pregnant women? Family Practice 1994 11:111–6 33. Haug NA, DiClemente C, Svikis DS. Motivational enhancement therapy for nicotine dependence in methadone-maintained pregnant women. Psychology of Addictive Behaviours 2004.18(3):289–92 34. Hegaard H, Hjaergaard H, Moller L, Wachmann H, Ottesen B. Multimodel intervention raises smoking cessation rate during pregnancy. Acta Obstetricia et Gynecologica Scandinavica 2003.82:813–9 35. Heil SH, Higgins ST, Bernstein IM, Solomon LJ, Rogers RE, Thomas CS, et al. Effects of voucher-based incentives on abstinence from cigarette smoking and fetal growth among pregnant women. Addiction. 2008.103(6):1009–18 36. Hennrikus D, Pirie P, Hellerstedt W, Lando HA, Steele J, Dunn C. Increasing support for smoking cessation during pregnancy and postpartum: results of a randomized controlled pilot study. Preventive Medicine 2010.50(3): 134–7 37. Herbec A, Brown J, Tombor I, Michie S, West R. Pilot randomized controlled trial of an internet-based smoking cessation intervention for pregnant smokers (’MumsQuit’). Drug & Alcohol Dependence 2014.140:130–6 38. Hiett A, Brazus S, Hedberg J, Brown H. Smoking cessation program effectiveness during pregnancy. American Journal of Obstetrics and Gynecology 2000.182(1 Pt 2):S150 39. Higgins ST, Washio Y, Lopez AA, Heil SH, Solomon LJ, Lynch ME, et al. Examining two different schedules of financial incentives for smoking cessation among pregnant women. Preventive Medicine 2014 Vol 68:51–7 40. Hjalmarson AIM, Svanberg B, Hahn L. Stopping smoking in pregnancy: effect of a self-help manual in a controlled trial. British Journal of Obstetrics and Gynaecology 1991.98: 260–4 41. Hughes E, Lamont D, Beecroft M, Wilson D. Randomized trial of a “stage-of-change” orientated smoking cessation intervention in infertile and pregnant women. Fertility and Sterility 2000.74(3):498–503 42. Kemp L, Harris E, McMahon C, Matthey S, Vimpani G, Anderson T, Schmied V, Aslam H, Zapart S. Child and family outcomes of a long-term nurse home visitation programme: a randomised controlled trial. Archives of Disease in Childhood 2011.96(6):533–540 43. Kendrick JS, Metzger RW, Sexton M, Spierto FW, Floyd RL, Gargiullo PM, et al. Integrating smoking cessation into routine public prenatal care: the Smoking Cessation in Pregnancy project. American Journal of Public Health 1995 85:217–22 44. Lawrence T, Aveyard P, Croghan E. What happens to women’s self-reported cigarette consumption and urinary cotinine levels in pregnancy? Addiction 2003.98:1315–20 45. Lee M, Miller SM, Wen KY, Hui SA, Roussi P, Hernandez E. Cognitive-behavioral intervention to promote smoking cessation for pregnant and postpartum inner city women. Journal of Behavioral Medicine 2015.38(6):923–43 46. LeFevre ML, Ewigman B, Evans JK. Is smoking an indication for prenatal ultrasonography? RADIUS Study Group. Archives of Family Medicine 1995.4:120–3 47. Lilley J, Forster DP. A randomised controlled trial of individual counselling of smokers in pregnancy. Public Health 1986.100:309–15 48. Lillington L, Chlebowski R, Ruvalcaba M, Novak D, Royce J. Evaluation of a smoking cessation program for pregnant minority smokers. Cancer Practice 1995.3(3):157–63 49. Loeb BK, Waage G, Bailey J. Smoking intervention in pregnancy. Proceedings of the Fifth World Conference on Smoking and Health 1983 July Winnipeg, Canada. 1983: 389–95 50. Loukopoulou A, Vardavas C, Tzatzarakis M, Farmakides G, Rossolymos C, Chrelias C, et al. Urinary nicotine and cotinine as a method for assessing the effectiveness of an intervention to aid smoking cessation during pregnancy. Chest 2012.142(4 Suppl 1):1081A 51. Lowe JB, Balanda KP, Stanton WR, Del Mar C, O’Connor V. Dissemination of an efficacious antenatal smoking cessation program in public hospitals in Australia: a randomised controlled trial. Health Education & Behavior 2002.29(5):608–19 52. Lowe JB, Windsor R, Balanda K, Woodby L. Smoking relapse prevention methods for pregnant women: a formative evaluation. American Journal of Health Promotion 1997.11:244–6 53. Malchodi CS, Oncken C, Dornelas EA, Caramanica L, Gregonis E. The effects of peer counselling on smoking cessation and reduction. Obstetrics & Gynecology 2003.101 (3):504–10 54. Manfredi C, Crittenden KS, Warnecke R, Engler J, Cho YI, Shaligram C. Evaluation of a motivational smoking cessation intervention for women in public health clinics. Preventive Medicine 1999.28:51–60 55. Mauriello L, Dyment S, Prochaska J, Gagliardi A, Weingrad-Smith J. Acceptability and feasibility of a multiple-behaviour, computer-tailored intervention for underserved pregnant women. Journal of Midwifery and Women’s Health 2011.56:75–80 56. Mayer JP, Todd R, Hawkins B. A randomised evaluation of smoking cessation interventions for pregnant women at a WIC clinic. American Journal of Public Health 1990.80: 76–7 57. McBride CM, Baucom DH, Peterson BL, Pollak KI, Palmer C, Westman E, et al. Prenatal and postpartum smoking abstinence: a partner assisted approach. American Journal of Preventive Medicine 2004.27(3):232–8 58. McBride CM, Curry SJ, Lando HA, Pirie PL, Grothaus LC, Nelson JC. Prevention of relapse in women who quit smoking during pregnancy. American Journal of Public Health 1999.89:706–11 59. McLeod D, Pullon S, Benn C, Cookson T, Dowell A, Viccars A, et al. Can support and education for smoking cessation and reduction be provided effectively by midwives within primary maternity care? Midwifery 2004.20:37–50 60. Mejdoubi J, van den Heijkant SCCM, van Leerdam FJM, Crone M, Crijnen A, HiraSing RA. Effects of nurse home visitation on cigarette smoking pregnancy outcomes: a randomized controlled trial. Midwifery 2014.30:688–95 61. Messimer SR, Henry RC, Hickner JM. A comparison of two antismoking interventions among pregnant women in eleven primary care practices. Journal of Family Practice 1989.28(3):283–8 62. Moore LO, Campbell R, Whelan A, Mills N, Lupton P, Misselbrook E, et al. Self help smoking cessation in pregnancy: cluster randomised controlled trial. BMJ 2002 325:1383–6 63. Moore ML, Meis PJ, Ernest JM, Wells HB, Zaccaro DJ, Terrell T. A randomized trial of nurse intervention to reduce preterm and low birth weight births. Obstetrics & Gynecology 1998.91:656–61 64. Naughton F, Prevost AT, Gilbert H, Sutton S. Randomized controlled trial evaluation of a tailored leaflet and SMS text message self-help intervention for pregnant smokers (MiQuit). Nicotine and Tobacco Research 2012.14(5): 569–77 65. Olds D, Robinson J, O’Brien R, Luckey D, Pettit L, Henderson C, et al. Home visiting by paraprofessionals and by nurses: a randomized, controlled trial. Pediatrics 2002 110(3):486–96 66. Olds DL, Henderson CR, Chamberlin R, Tatelbaum R. Preventing child abuse and neglect: a randomized trial of nurse home visitation. Pediatrics 1986.78:65–78 67. Ondersma SJ, Svikis DS, Lam PK, Connors-Burge VS, Ledgerwood DM, Hopper JA. A randomized trial of computer-delivered brief intervention and low-intensity contingency management for smoking during pregnancy. Nicotine & Tobacco Research 2012 Vol 14, issue 3: 351–60 68. Oude Wesselink SF, Lingsma HF, Reulings PG, Wentzel HR, Erasmus V, Robben PB, et al. Does government supervision improve stop-smoking counseling in midwifery practices? Nicotine & Tobacco Research 2015 Vol 17, issue 5:572–9 69. Panjari M, Bell R, Bishop S, Astbury J, Rice G, Doery J. A randomized controlled trial of a smoking cessation intervention during pregnancy. Australian and New Zealand Journal of Obstetrics and Gynaecology. 1999.39(3):312–7 70. Parker DR, Windsor RA, Roberts MB, Hecht J, Hardy NV, Strolla LO, et al. Feasibility, cost, and costeffectiveness of a telephone-based motivational intervention for underserved pregnant smokers. Nicotine and Tobacco Research 2007.9(10):1043–51 71. Patten CA, Windsor RA, Renner CC, Enoch C, Hochreiter A, Nevak C, et al. Feasibility of a tobacco cessation intervention for pregnant Alaska Native women. Nicotine & Tobacco Research 2009.12(2):79–87 72. Pbert L, Ockene JK, Zapka J, Ma Y, Goins KV, Oncken C, et al. A community health center smoking cessation intervention for pregnant and postpartum women. American Journal of Preventive Medicine 2004.26(5): 377–85 73. Peterson L, Rosen A, Podedworny T, Kotch J, Handel J. Smoking reduction during pregnancy by a program of selfhelp and clinical support. Obstetrics & Gynecology 1992.79: 924–30 74. Polanska K, Hanke W, Sobala W, Lowe JB. Efficacy and effectiveness of the smoking cessation program for pregnant women. International Journal of Occupational Medicine and Environmental Health 2004.17(3):369–77 75. Pollak KI, Lyna P, Bilheimer A, Farrell D, Gao X, Swamy GK, et al. A pilot study testing SMS text delivered scheduled gradual reduction to pregnant smokers. Nicotine & Tobacco Research 2013.15(10):1773–6 76. Prapavessis H, De Jesus S, Harper T, Cramp A, Fitzgeorge L, Mottola MF, et al. The effects of acute exercise on tobacco cravings and withdrawal symptoms in temporary abstinent pregnant smokers. Addictive Behaviors 2014.39(3):703–8 77. Price JH, Snyder FF, Roberts SM, Losh DP, Desmond SM, Krol RA. Comparison of three antismoking interventions among pregnant women in an urban setting: a randomized trial. Psychological Reports 1991.68:595–604 78. Reading AE, Cox DN. The effects of ultrasound on maternal anxiety. Journal of Behavioral Medicine 1982.5(2): 237–47 79. Rigotti N, Park ER, Regan S, Chang Y, Perry K, Loudin B, et al. Efficacy of telephone counseling for pregnant smokers. Obstetrics & Gynecology 2006.108(1):83–92 80. Robling M, Bekkers MJ, Bell K, Butler CC, CanningsJohn R, Channon S, et al. Effectiveness of a nurse-led intensive home-visitation programme for first-time teenage mothers (Building Blocks): a pragmatic randomised controlled trial. Lancet 2016.387(10014):146–55 81. Secker-Walker RH, Mead PB, Goodwin GD, Lepage SS, Skelly JM, Flynn BS, et al. Individualised smoking cessation counseling during prenatal and early postnatal care. American Journal of Obstetrics and Gynecology 1994 71:1347–55 82. Secker-Walker RH, Solomon LJ, Flynn BS, Skelly JM, Mead PB. Reducing smoking during pregnancy and postpartum: physician’s advice supported by individual counselling. Preventive Medicine 1998.27:422–30 83. Secker-Walker RH, Solomon LJ, Geller BM, Flynn BS, Worden JK, Skelly JM, et al. Modeling smoking cessation: exploring the use of a videotape to help pregnant women quit smoking. Women & Health 1997.25:23–35 84. Sexton M, Hebel JR. A clinical trial of change in maternal smoking and its effect on birth weight. JAMA 1984.251: 911–5 85. Solomon LJ, Secker-Walker RH, Flynn BS, Skelly JM, Capeless EL. Proactive telephone peer support to help pregnant women stop smoking. Tobacco Control 2000.9 Suppl 3:iii72–iii74 86. Stotts AL, DeLaune KA, Schmitz JM, Grabowski J. Impact of a motivational intervention on mechanisms of change in low-income pregnant women. Addictive Behaviors 2004.29 (8):1649–57 87. Stotts AL, Groff JY, Velasquez MM, Benjamin-Garner R, Green C, Carbonari JP, et al. Ultrasound feedback and motivational interviewing targeting smoking cessation in the second and third trimesters of pregnancy. Nicotine & Tobacco Research 2009.11(8):961–8 88. Stotts S, DiClemente CC, Dolan-Mullen P. One-to-One: a motivational intervention for resistant pregnant smokers. Addictive Behaviors 2002.27:275–92 89. Strecher VJ, Bishop KR, Bernhardt J, Thorp JM, Cheuvrout B, Potts P. Quits for keeps: tailored smoking cessation guides for pregnancy and beyond. Tobacco Control 2000.9 (Suppl 3):iii78–iii79 90. Tappin D, Bauld L, Purves D, Boyd K, Sinclair L, MacAskill S, et al. Financial incentives for smoking cessation in pregnancy: randomised controlled trial. BMJ (Clinical Research Ed.) 2015.350:h134 91. Tappin DM, Lumsden MA, Gilmour WH, Crawford F, McIntyre D, Stone DH, et al. Randomised controlled trial of home based motivational interviewing by midwives to help pregnant smokers quit or cut down. BMJ 2005.331: 373–7 92. Tappin DM, Lumsden MA, McIntyre D, McKay C, Gilmour WH, Webber R, et al. A pilot study to establish a randomized trial methodology to test the efficacy of a behavioural intervention. Health Education Research 2000 15(4):491–502 93. Thornton L. Smoking and Pregnancy: Feasibility and Effectiveness of a Smoking Intervention Programme Among Pregnant Women [thesis]. Dublin: Dept of Public Health, 1997 94. Tsoh JY, Kohn MA, Gerbert B. Promoting smoking cessation in pregnancy with Video Doctor plus provider cueing: a randomized trial. Acta Obstetricia et Gynecologica Scandinavica 2010.89(4):515–23 95. Tuten M, Fitzsimons H, Chisolm MS, Nuzzo PA, Jones HE. Contingent incentives reduce cigarette smoking among pregnant, methadone-maintained women: results of an initial feasibility and efficacy randomized clinical trial. Addiction 2012.107(10):1868–77 96. Ussher M, Lewis S, Aveyard P, Manyonda I, West R, Lewis B, et al. Physical activity for smoking cessation in pregnancy: randomised controlled trial. BMJ (Clinical Research Ed.) 2015.350:h2145 97. Valbo A, Eide T. Smoking cessation in pregnancy: the effect of hypnosis in a randomized study. Addictive Behaviors 1996.21:29–35 98. Valbo A, Nylander G. Smoking cessation in pregnancy: intervention among heavy smokers. Acta Obstetricia et Gynecologica Scandinavica 1994.73:215–9 99. Vilches P. Consumption of Tobacco in Pregnant Women: Proposal of a Psychological Intervention Model in the Public Health System of Andalucia [thesis] Malaga: Univesidad di Malaga, 2009 100. Walsh RA, Melmeth A, Byrne JM, Brinsmead MW, Redman S. A smoking cessation program at a public antenatal clinic. American Journal of Public Health 1997.87: 1201–4 101. Wilkinson SA, McIntyre HD. Evaluation of the ’healthy start to pregnancy’ early antenatal health promotion workshop: a randomized controlled trial. BMC Pregnancy and Childbirth 2012.0.590972222222222 102. Windsor R, Woodby L, Miller T, Hardin M. Effectiveness of Smoking Cessation and Reduction in Pregnancy Treatment (SCRIPT) methods in Medicaid-supported prenatal care: Trial III. Health Education & Behavior 2011.38(4):412–22 103. Windsor RA, Lowe JB, Perkins LL, Smith-Yoder D, Artz L, Crawford M, et al. Health education for pregnant smokers: its behavioral impact and cost benefit. American Journal of Public Health 1993.83:201–6 104. Windsor RA, Spanos D, Samuelsson C, Bartlett EE, Manzella B, Reese Y, et al. The effectiveness of smoking cessation methods for smokers in public health maternity clinics: a randomized trial. American Journal of Public Health 1985.75:1389–92 |
| Griffiths *et al.* 2016[15] | 1. Abroms, L. C., Johnson, P. R., Leavitt, L. E., Cleary, S. D., Bushar, J., Brandon, T. H., & Chiang, S. C. (2017). A randomized trial of text messaging for smoking cessation in pregnant women. American Journal of Preventive Medicine, 53(6), 781–790. doi:10.1016/j.amepre.2017.08.002 2. Cinciripini, P. M., McClure, J. B., Wetter, D. W., Perry, J., Blalock, J. A., Cinciripini, L. G., … Skaar, K. (2000). An evaluation of videotaped vignettes for smoking cessation and relapse prevention during pregnancy: The Very Important Pregnant Smokers (VIPS) program. Tobacco Control, 9(suppl 3), iii61–iii63. doi:10.1136/tc.9.suppl_3.iii61 3. Ershoff, D. H., Quinn, V. P., Boyd, N. R., Stern, J., Gregory, M., & Wirtschafter, D. (1999). The Kaiser Permanente prenatal smoking-cessation trial: When more isn’t better, what is enough? American Journal of Preventive Medicine, 17(3), 161– 168. doi:10.1016/S0749-3797(99)00071-9 4. Harris, M., & Reynolds, B. (2015). A pilot study of home-based smoking cessation programs for rural, Appalachian, pregnant smokers. Journal of Obstetric, Gynecologic, & Neonatal Nursing, 44(2), 236–245. doi:10.1111/1552-6909.12547 5. Herbec, A., Brown, J., Tombor, I., Michie, S., & West, R. (2014). Pilot randomized controlled trial of an internet-based smoking cessation intervention for pregnant smokers (‘MumsQuit’). Drug and Alcohol Dependence, 140, 130–136. doi:10.1016/j.drugalcdep.2014.04.010 6. Lawrence, T., Aveyard, P., Evans, O., & Cheng, K. (2003). A cluster randomised controlled trial of smoking cessation in pregnant women comparing interventions based on the transtheoretical (stages of change) model to standard care. Tobacco Control, 12(2), 168–177. doi:10.1136/tc.12.2.168 7. Naughton, F., Cooper, S., Foster, K., Emery, J., Leonardi-Bee, J., Sutton, S., … Leighton, M. (2017). Large multi-centre pilot randomized controlled trial testing a low-cost, tailored, self-help smoking cessation text message intervention for pregnant smokers (MiQuit). Addiction, 112(7), 1238–1249. doi:10.1093/ntr/ntr254 8. Naughton, F., Prevost, A. T., Gilbert, H., & Sutton, S. (2012). Randomized controlled trial evaluation of a tailored leaflet and SMS text message self-help intervention for pregnant smokers (MiQuit). Nicotine & Tobacco Research, 14(5), 569–577. doi:10.1093/ntr/ntr254 9. Ondersma, S. J., Svikis, D. S., Lam, P. K., Connors-Burge, V. S., Ledgerwood, D. M., & Hopper, J. A. (2012). A randomized trial of computer-delivered brief intervention and low-intensity contingency management for smoking during pregnancy. Nicotine & Tobacco Research, 14(3), 351–360. doi:10.1093/ntr/ntr221 10. Pollak, K. I., Lyna, P., Bilheimer, A., Farrell, D., Gao, X., Swamy, G. K., & Fish, L. J. (2013). A pilot study testing SMS text delivered scheduled gradual reduction to pregnant smokers. Nicotine & Tobacco Research, 15(10), 1773–1776. doi:10.1093/ ntr/ntt045 11. Price, J. H., Desmond, S. M., Roberts, S. M., Krol, R. A., Losh, D. P., & Snyder, F. F. (1991). Comparison of three antismoking interventions among pregnant women in an urban setting: A randomized trial. Psychological Reports, 68(2), 595–604. doi:10.2466/PR0.68.2.595-604 12. Secker-Walker, R. H., Solomon, L. J., Geller, B. M., Flynn, B. S., Worden, J. K., Skelly, J. M., & Mead, P. B. (1997). Modeling smoking cessation: Exploring the use of a videotape to help pregnant women quit smoking. Women & Health, 25(1), 23–35. doi:10.1300/J013v25n01_02 |
| Hand *et al.* 2017[16] | 1. Carroll, K. M., Chang, G., Behr, H., Clinton, B., & Kosten, T. R. (1995). Improving treatment outcome in pregnant, methadone-maintained women: Results from a randomized clinical trial. The American Journal on Addictions, 4(1), 56-59. Doi: 10.1111/j.1521-0391.1995.tb00259.x 2. Chang, G., Carroll, K. M., Behr, H. M., & Kosten, T. R. (1992). Improving treatment outcome in pregnant opiate-dependent women. Journal of Substance Abuse Treatment, 9(4), 327-330 3. Donatelle, R. J., Prows, S. L., Champeau, D., & Hudson, D. (2000). Randomised controlled trial using social support and financial incentives for high risk pregnant smokers: significant other supporter (SOS) program. Tobacco Control, 9 (Suppl. 3), S67-69 4. Elk, R., Mangus, L., Rhoades, H., Andres, R., & Grabowski, J. (1998). Cessation of cocaine use during pregnancy: effects of contingency management interventions on maintaining abstinence and complying with prenatal care. Addictive Behaviors, 23(1), 57-64 5. Elk, R., Schmitz, J., Spiga, R., Rhoades, H., Andres, R., & Grabowski, J. (1995). Behavioral treatment of cocaine-dependent pregnant women and TB-exposed patients. Addictive Behaviors, 20(4), 533-542 6. Gadomski, A., Adams, L., Tallman, N., Krupa, N., & Jenkins, P. (2011). Effectiveness of a combined prenatal and postpartum smoking cessation program. Maternal and Child Health Journal, 15(2), 188-197. doi: 10.1007/s10995-010-0568-9 7. Glover, M., Kira, A., Walker, N., & Bauld, L. (2015). Using incentives to encourage smoking abstinence among pregnant indigenous women? A feasibility study. Maternal and Child Health Journal, 19(6), 1393-1399. doi: 10.1007/s10995-014- 1645-2 8. Harris, M., & Reynolds, B. (2015). A pilot study of home?based smoking cessation programs for rural, Appalachian, pregnant smokers. Journal of Obstetric, Gynecologic, & Neonatal Nursing, 44(2), 236-245 9. Heil, S. H., Higgins, S. T., Bernstein, I. M., Solomon, L. J., Rogers, R. E., Thomas, C. CONTINGENCY MANAGEMENT AMONG PREGNANT WOMEN 31 S., Badger, G.J. Lynch, M. E. (2008). Effects of voucher-based incentives on abstinence from cigarette smoking and fetal growth among pregnant women. Addiction, 103(6), 1009-1018. doi: 10.1111/j.1360-0443.2008.02237.x 10. Higgins, S. T., Heil, S. H., Solomon, L. J., Bernstein, I. M., Lussier, J. P., Abel, R. L., Lynch, M.E.Badger, G. J. (2004). A pilot study on voucher-based incentives to promote abstinence from cigarette smoking during pregnancy and postpartum. Nicotine & Tobacco Research, 6(6), 1015-1020 11. Higgins, S. T., Washio, Y., Lopez, A. A., Heil, S. H., Solomon, L. J., Lynch, M. E., Hanson, J. D., Higgins, T. M., Skelly, J.M., Redner, R. & Bernstein, I. M. (2014). Examining two different schedules of financial incentives for smoking cessation among pregnant women. Preventive Medicine, 68, 51-57. doi: 10.1016/j.ypmed.2014.03.024 12. Ierfino, D., Mantzari, E., Hirst, J., Jones, T., Aveyard, P., & Marteau, T. M. (2015). Financial incentives for smoking cessation in pregnancy: a single-arm intervention study assessing cessation and gaming. Addiction, 110(4), 680-688. doi: 10.1111/add.12817 13. Jones, H. E., Haug, N., Silverman, K., Stitzer, M., & Svikis, D. (2000). Improving treatment outcomes for pregnant drug-dependent women using low-magnitude voucher incentives Addictive Behaviors, 25(2), 263-267 14. Jones, H. E., Haug, N., Silverman, K., Stitzer, M., & Svikis, D. (2001). The effectiveness of incentives in enhancing treatment attendance and drug abstinence in methadone-maintained pregnant women. Drug and Alcohol Dependence, 61(3), 297-306 15. Jones, H. E., Svikis, D., & Tran, G. (2002). Patient compliance and maternal/infant outcomes in pregnant drug-using women. Substance Use & Misuse, 37(11), 1411-1422 16. Jones, H. E., Svikis, D., Rosado, J., Tuten, M., & Kulstad, J. L. (2004). What if they do not want treatment?: Lessons learned from intervention studies of non-treatment seeking, drug-using pregnant women. American Journal on Addictions, 13(4), 342-357 17. Ker, M., Leischow, S., Markowitz, I. B., & Merikle, E. (1996). Involuntary smoking cessation: a treatment option in chemical dependency programs for women and children. Journal of Psychoactive Drugs, 28(1), 47-60. doi: 10.1080/02791072.1996.10471714 18. Ondersma, S. J., Svikis, D. S., Lam, P. K., Connors-Burge, V. S., Ledgerwood, D. M., & Hopper, J. A. (2012). A randomized trial of computer-delivered brief intervention and low-intensity contingency management for smoking during pregnancy. Nicotine & Tobacco Research, 14(3), 351-360. doi: 10.1093/ntr/ntr221 19. Radley, A., Ballard, P., Eadie, D., MacAskill, S., Donnelly, L., & Tappin, D. (2013). Give it up for baby: Outcomes and factors influencing uptake of a pilot smoking cessation incentive scheme for pregnant women. BMC public health, 13(1), 343. doi: 10.1186/1471-2458-13-343 20. Schottenfeld, R. S., Moore, B., & Pantalon, M. V. (2011). Contingency management with community reinforcement approach or twelve-step facilitation drug counseling for cocaine dependent pregnant women or women with young children. Drug and Alcohol Dependence, 118(1), 48-55 |
| Heminger *et al.* 2016[17] | 1. Abroms LC, Johnson PR, Heminger CL, et al. Quit4baby: results from a pilot test of a mobile smoking cessation program for pregnant women. JMIR Mhealth Uhealth. 2015;3(1):e10 2. Cooper S, Foster K, Naughton F, et al. Pilot study to evaluate a tailored text message intervention for pregnant smokers (MiQuit): study protocol for a randomised controlled trial. Trials. 2015;16:29. 29 3. Naughton F, Jamison J, Sutton S. Attitudes towards SMS text message smoking cessation support: a qualitative study of pregnant smokers. Health Educ Res. 2013;28(5):911–922. doi: 10.1093/her/cyt057 4. Naughton F, Prevost AT, Gilbert H, Sutton S. Randomized controlled trial evaluation of a tailored leaflet and SMS text message self-help intervention for pregnant smokers (MiQuit). Nicotine Tob Res. 2012; 14(5):569–577. 27 5. Pollack KI, Lyna P, Bilheimer A, et al. A pilot study testing SMS text delivered scheduled gradual reduction to pregnant smokers. Nicotine Tob Res. 2013;15(10):1773–1776 6. van Mierlo T, Fournier R, Jean-Charles A, Hovington J, Ethier I, Selby P. I’ll txt u if i have a problem: how the Société? Canadienne du cancer in Quebec applied behavior-change theory, data mining and agile software development to help young adults quit smoking. PLoS One. 2014;9(3):e91832. 30 |
| Veisani *et al.* 2017[18] | 1. Benjamin-Garner R, Stotts A. Impact of smoking exposure change on infant birth weight among a cohort of women in a Prenatal Smoking Cessation Study. Nicotine Tob Res. 2013;15(3):685–692 2. Berlin I, Grang e G, Jacob N, et al. Nicotine patches in pregnant smokers: randomised, placebo controlled, multicentre trial of efficacy. BMJ. 2014;348:g1622 3. England LJ, Kendrick JS, Wilson HG, et al. Effects of smoking reduction during pregnancy on the birth weight of term infants. Am J Epidemiol. 2001;154(8): 694–701 4. Higgins ST, Bernstein IM, Washio Y, et al. Effects of smoking cessation with voucher-based contingency management on birth outcomes. Addiction. 2010; 105(11):2023–2030 5. Higgins ST, Washio Y, Lopez AA, et al. Examining two different schedules of financial incentives for smoking cessation among pregnant women. Prev Med. 2014;68:51–57 6. MacArthur C, Knox EG, Lancashire RJ. Effects at age nine of maternal smoking in pregnancy: experimental and observational findings. BJOG. 2001;108(1):67–73 7. Mejdoubi J, van den Heijkant SCCM, van Leerdam FJM, et al. Effects of nurse home visitation on cigarette smoking, pregnancy outcomes and breastfeeding: a randomized controlled trial. Midwifery. 2014;30(6): 688–695 8. Oncken C, Dornelas E, Greene J, et al. Nicotine gum for pregnant smokers: a randomized controlled trial. Obstet Gynecol. 2008;112(4):859–867 9. Panjari M, Bell R, Bishop S, et al. A Randomized Controlled Trial of a Smoking Cessation intervention during pregnancy. Aust NZ J Obstet Gynaecol. 1999;39(3):312–317 10. Polanska K, Hanke W, Sobala W, et al. Efficacy and effectiveness of the smoking cessation program for pregnant women. Int J Occup Med Environ Health. 2004;17(3):369–377 11. Ram A, Tuten M, Chisolm MS. Cigarette smoking reduction in pregnant women with opioid use disorder. J Addict Med. 2016;10(1):53–59 12. Ruger JP, Weinstein MC, Hammond SK, et al. Costeffectiveness of motivational interviewing for Smoking Cessation and relapse prevention among low-income pregnant women: a randomized controlled trial. Value Health J Int Soc Pharmacoecon Outcomes Res. 2008;11(2):191–198. 13. Tappin D, Bauld L, Purves D, et al. Financial incentives for smoking cessation in pregnancy: randomised controlled trial. BMJ. 2015;350:h134 14. Tappin DM, Lumsden MA, Gilmour WH, et al. Randomised controlled trial of home based motivational interviewing by midwives to help pregnant smokers quit or cut down. BMJ. 2005;331(7513): 373–377 15. Ussher M, Lewis S, Aveyard P, et al. The London Exercise and Pregnant smokers (LEAP) trial: a randomised controlled trial of physical activity for smoking cessation in pregnancy with an economic evaluation. Health Technol Assess. 2015;19(84):vii–xxiv, 1 16. Wisborg K, Henriksen TB, Jespersen LB, et al. Nicotine patches for pregnant smokers: a randomized controlled study. Obstet Gynecol. 2000;96(6):967–971 |
| Hubbard *et al.* 2016[19] | 1. de Vries H, Bakker M, Dolan Mullen P, van Breukelen G. The effects of smoking cessation counseling by midwives on Dutch pregnant women and their partners. Patient Educ Couns. 2006;63:177–87 2. Hjermann I, Velve Byre K, Holme I, Leren P. Effect of diet and smoking intervention on the incidence of coronary heart disease: report from the Oslo Study Group or a randomized trial in healthy men. Lancet. 1981;12: 1303–10 3. McBride CM, Baucom DH, Peterson BL, Pollak KI, Palmer C, Westman E, et al. Prenatal and postpartum smoking abstinence: a partner-assisted approach. Am J Prev Med. 2004;27:232–8 4. McIntyre-Kingsolver K, Lichtenstein E, Mermelstein RJ. Spouse training in a multicomponent smoking-cessation program. Behav Ther. 1986;17: 67–74 5. Nyborg KF, Nevid JS. Couples who smoke: a comparison of couples training versus individual training for smoking cessation. Behav Ther. 1986;17:620–5. 6. Øien T, Storrø O, Jenssen JA, Johnsen R. The impact of a minimal smoking cessation intervention for pregnant women and their partners on perinatal smoking behavior in primary health care: a real-life controlled study. BMC Pub Health. 2008;8:325 7. Patten CA, Hughes CA, Lopez KN, Thomas JL, Brockman TA, Smith CM, et al. Web-based intervention for adolescent nonsmokers to help parents stop smoking: a pilot feasibility study. Addict Behav. 2012;37:85–91 8. Wood DA, Kotseva K, Connolly S, Jennings C, Mead A, Jones J, Holden A, De Bacquer D, Collier T, De Backer G, Faergeman O, EUROACTION Study Group. Nurse-coordinated multidisciplinary, family based cardiovascular disease prevention programme (EUROACTION) for patients with coronary heart disease and asymptomatic individuals at high risk of cardiovascular disease: a paired, cluster-randomised controlled trial. Lancet. 2008;371:199–2012 |
| Wilson et al 2018[20] | 1. Bradizza, C. M., Stasiewicz, P. R., Zhuo, Y., Ruszczyk, M., Maisto, S. A., Lucke, J. F., . Giarratano, P. (2017). Smoking cessation for pregnant smokers: Development and pilot test of an emotion regulation treatment supplement to standard smoking cessation for negative affect smokers. Nicotine & Tobacco Research, 19(5), 578–584 2. Bullock, L., Everett, K. D., Mullen, P. D., Geden, E., Longo, D. R., & Madsen, R. (2009). Baby BEEP: A randomized controlled trial of nurses’ individualized social support for poor rural pregnant smokers. Maternal and Child Health Journal, 13(3), 395–406 3. Cinciripini, P. M., Blalock, J. A., Minnix, J. A., Robinson, J. D., Brown, V. L., Lam, C., . Karam-Hage, M. (2010). Effects of an intensive depression-focused intervention for smoking cessation in pregnancy. Journal of Consulting and Clinical Psychology, 78(1), 44–54. 4. Dornelas, E. A., Magnavita, J., Beazoglou, T., Fischer, E. H., Oncken, C., Lando, H., . Gregonis, E. (2006). Efficacy and cost-effectiveness of a clinic-based counseling intervention tested in an ethnically diverse sample of pregnant smokers. Patient Education and Counseling, 64(1-3), 342– 349 5. Ershoff, D. H., Quinn, V. P., Boyd, N. R., Stern, J., Gregory, M., & Wirtschafter, D. (1999). The Kaiser Permanente prenatal smoking-cessation trial: when more isn’t better, what is enough? American Journal of Preventive Medicine, 17(3), 161–168. 6. Lee, M., Miller, S. M., Wen, K. Y., Hui, S. K., Roussi, P., & Hernandez, E. (2015). Cognitive-behavioral intervention to promote smoking cessation for pregnant and postpartum inner city women. Journal of Behavioral Medicine, 38, 932–943 7. McBride, C. M., Baucom, D. H., Peterson, B. L., Pollak, K. I., Palmer, C., Westman, E., & Lyna, P. (2004). Prenatal and postpartum smoking abstinence a partnerassisted approach. American Journal of Preventive Medicine, 27(3), 232–238. 8. Panjari, M., Bell, R., Bishop, S., Astbury, J., Rice, G., & Doery, J. (1999). A randomized controlled trial of a smoking cessation intervention during pregnancy. Australian & New Zealand Journal of Obstetrics & Gynaecology, 39(3), 312–317. 9. Rigotti, N. A., Park, E. R., Regan, S., Chang, Y., Perry, K., Loudin, B., & Quinn, V. (2006). Efficacy of telephone counseling for pregnant smokers: A randomized controlled trial. Obstetrics and Gynecology, 108(1), 83–92. 10. Ruger, J. P., Weinstein, M. C., Hammond, S. K., Kearney, M. H., & Emmons, K. M. (2008). Cost-effectiveness of motivational interviewing for smoking cessation and relapse prevention among low-income pregnant women: a randomized controlled trial. Value in Health, 11(2), 191–198. 11. Secker-Walker, R. H., Solomon, L. J., Flynn, B. S., Skelly, J. M., Lepage, S. S., Goodwin, G. D., & Mead, P. B. (1994). Individualized smoking cessation counseling during prenatal and early postnatal care. American Journal of Obstetrics and Gynecology, 171(5), 1347–1355 12. Secker-Walker, R. H., Solomon, L. J., Flynn, B. S., Skelly, J. M., & Mead, P. B. (1998). Reducing smoking during pregnancy and postpartum: Physician’s advice supported by individual counseling. Preventive Medicine, 27(3), 422–430. 13. Stotts, A. L., DiClemente, C. C., & Dolan-Mullen, P. (2002). One-to-one: A motivational intervention for resistant pregnant smokers. Addictive Behaviors, 27(2), 275–292 14. Stotts, A. L., Groff, J. Y., Velasquez, M. M., Benjamin-Garner, R., Green, C., Carbonari, J. P., & DiClemente, C. C. (2009). Ultrasound feedback and motivational interviewing targeting smoking cessation in the second and third trimesters of pregnancy. Nicotine & Tobacco Research, 11(8):961-968 15. Tappin, D. M., Lumsden, M. A., Gilmour, W. H., Crawford, F., McIntyre, D., Stone, D. H., . Mohammed, E. (2005). Randomised controlled trial of home based motivational interviewing by midwives to help pregnant smokers quit or cut down. BMJ, 331, 373–377 16. Tappin, D. M., Lumsden, M. A., McIntyre, D., McKay, C., Gilmour, W. H., Webber, R., . Currie, F. (2000). A pilot study to establish a randomized trial methodology to test the efficacy of a behavioural intervention. Health Education Research, 15(4), 491–502 17. Donatelle, R. J., Prows, S. L., Champeau, D., & Hudson, D. (2000). Randomised controlled trial using social support and financial incentives for high risk pregnant smokers: significant other supporter (SOS) program. Tobacco Control, 9(Suppl 3), iii67–iii69 18. Heil, S. H., Higgins, S. T., Bernstein, I. M., Solomon, L. J., Rogers, R. E., Thomas, C. S., . Lynch, M. E. (2008). Effects of voucher-based incentives on abstinence from cigarette smoking and fetal growth among pregnant women. Addiction, 103(6), 1009–1018 19. Higgins, S. T., Washio, Y., Lopez, A. A., Heil, S. H., Solomon, L. J., Lynch, M. E., . Bernstein, I. M. (2014). Examining two different schedules of financial incentives for smoking cessation among pregnant women. Preventive Medicine, 68, 51–57. 20. Ondersma, S. J., Svikis, D. S., Lam, P. K., Connors-Burge, V. S., Ledgerwood, D. M., & Hopper, J. A. (2012). A randomized trial of computer-delivered brief intervention and low-intensity contingency management for smoking during pregnancy. Nicotine & Tobacco Research, 14(3), 351–360. 21. Tappin, D. M., Bauld, L., Purves, D., Boyd, K., Sinclair, L., MacAskill, S., . Coleman, T. (2015). Financial incentives for smoking cessation in pregnancy: Randomised controlled trial. BMJ, 350, h134. 22. Tuten, M., Fitzsimons, H., Chisolm, M. S., Nuzzo, P. A., & Jones, H. E. (2012). Contingent incentives reduce cigarette smoking among pregnant, methadone-maintained women: Results of an initial feasibility and efficacy randomized clinical trial. Addiction, 107(10), 1868–1877. |

**S7d: Unique publications inlcuded in smoking reviews**

| **Smoking unique publications** |
| --- |
| 1. Abroms LC, Johnson PR, Heminger CL, et al. Quit4baby: results from a pilot test of a mobile smoking cessation program for pregnant women. JMIR Mhealth Uhealth. 2015;3(1):e10 |
| 1. Abroms, L. C., Johnson, P. R., Leavitt, L. E., Cleary, S. D., Bushar, J., Brandon, T. H., & Chiang, S. C. (2017). A randomized trial of text messaging for smoking cessation in pregnant women. American Journal of Preventive Medicine, 53(6), 781–790. doi:10.1016/j.amepre.2017.08.002 |
| 1. Albrecht S, Cassidy B, Salamie D, Reynolds M. What’s happening. A smoking cessation intervention for pregnant adolescents: implications for nurse practitioners. Journal of American Academy of Nurse Practitioners 199911(4):155–9 |
| 1. Albrecht S, Cornelius M, Braxter B, Reynolds M, Stone C, Cassidy B. An assessment of nicotine dependence among pregnant adolescents. Journal of Substance Abuse Treatment 199916(4):337–44 |
| 1. Albrecht S, Stone CA, Payne L, Reynolds MD. A preliminary study of the use of peer support in smoking cessation programs for pregnant adolescents. Journal of the American Academy of Nurse Practitioners 199810:119–25 |
| 1. Albrecht SA, Caruthers D, Patrick T, Reynolds M, Salamie D, Higgins LW, et al. A randomised controlled trial of a smoking cessation intervention for pregnant adolescents. Nursing Research 200655(6):402–10 |
| 1. Albrecht SA, Caruthers D. Characteristics of inner-city pregnant Smoking teenagers. Journal of Obstetric Gynecologic and Neonatal Nursing 200231:462–9 |
| 1. Albrecht SA, Higgins LW, Lebow H. Knowledge about the deletrious effects of smoking and its relationship to smoking cessation among pregnant adolescents. Adolescence 200035 (140):709–1 |
| 1. Albrecht SA, Higgins LW, Stone C. Factors relating to pregnant adolescents’ decisions to complete a smoking cessation intervention. Journal of Pediatric Nursing 199914 (5):322–8 |
| 1. Albrecht SA, Patrick T, Kim Y, Caruthers D. A randomised controlled trial of a smoking cessation intervention for pregnant adolescents. Society for Research on Nicotine and Tobacco 9th Annual Meeting 2003 February 19-23 New Orleans, Louisiana. 2003:91 |
| 1. Altman D., Ashby D., Birks J., Borenstein M., Campbell M.,Felix Cinciripini P. M., McClure J. B., Wetter D. W., Perry J., Blalock J. A., Cinciripini L. G. et al. An evaluation of videotaped vignettes for smoking cessation and relapse prevention during pregnancy: the very important pregnant smokers (VIPS) program. Tob Control 2000 9: III61–3 |
| 1. Aranda Regules JM, Mateos Vilchez P, Gonzalez Villalba A, Sanchez F, de Dios Luna del Castillo J. Validity of smoking measurements during pregnancy: specificity, sensitivity and cut-off points. Revista Espanola de Salud Publica 200882 (5):535–45 |
| 1. Aveyard P, Lawrence T, Cheng KK, Griffin C, Croghan?E, Johnson C. A randomized controlled trial of smoking cessation for pregnant women to test the effect of a transtheoretical model-based intervention on movement in stage and interaction with baseline stage. British Journal of Health Psychology 200611:263–78 |
| 1. Aveyard P, Lawrence T, Croghan E, Evans O, Cheng KK. Is advice to stop smoking from a midwife stressful for pregnant women who smoke? Data from a randomized controlled trial. Preventive Medicine 200440:575–82 |
| 1. Aveyard P, Lawrence T, Evans O, Cheng KK. The influence of in-pregnancy smoking cessation programmes on partner quitting and women’s social support mobilization: a randomized controlled trial. BMC Public Health 20055:80. Aveyard P, West R. Managing smoking cessation. BMJ 2007335:37–41 |
| 1. Bailey JW, Loeb BK, Waage G. A randomized trial of smoking intervention during pregnancy. Proceedings of the American Public Health Association 111th Annual Meeting 1983 Nov 15 Dallas, Texas, USA. 1983:58 |
| 1. Baric L, MacArthur C, Sherwood M. A study of health education aspects of smoking in pregnancy. International Journal of Health Education 197619(2 Suppl):1–17 |
| 1. Baric L, MacArthur C. Health norms in pregnancy. British Journal of Preventive and Social Medicine 197731:30–8 |
| 1. Bauman KE, Koch GG, Dent CW, Bryan ES. The influence of observing carbon monoxide level on cigarette smoking by public prenatal patients. American Journal of Public Health 198373:1089–91 |
| 1. Belizan JM, Villar J, Victora C, Farnot U, Langer A, Barros F. Impact of health education during pregnancy on behavior and utilization of health resources. American Journal of Obstetrics and Gynecology 1995173:894–9 |
| 1. Benjamin-Garner R, Stotts A. Impact of smoking exposure change on infant birth weight among a cohort of women in a Prenatal Smoking Cessation Study. Nicotine Tob Res. 2013;15(3):685–692 |
| 1. Berg CJ, Park ER, Chang Y, Rigotti NA. Is concern about post-cessation weight gain a barrier to smoking cessation among pregnant women?. Nicotine & Tobacco Research 200810(7):1159–63 |
| 1. Berlin I, Grang e G, Jacob N, et al. Nicotine patches in pregnant smokers: randomised, placebo controlled, multicentre trial of efficacy. BMJ. 2014;348:g1622 |
| 1. Bernstein IM, Mongeon JA, Badger GJ, Solomon L, Heil SH, Higgins ST. Maternal smoking and its association with birthweight. Obstetrics & Gynecology 2005106(5 Pt 1): 986–91 |
| 1. Blake S, El-Mohandes A, Schwartz D, El-Khorazaty N, Gantz M, Joseph J, et al. Promoting smoking cessation during pregnancy and preventing postpartum relapse [abstract]. Pediatric Academic Societies Annual Meeting 2005 May 14-17 Washington DC, USA. 2005:Abstract no: 3074 |
| 1. Blake S, Joseph J, Schwartz D, El-Khorazaty N, Gantz M, El-Mohandes A, et al. Preventing prenatal and postpartum environmental tobacco smoke (ETS) exposure [abstract]. Pediatric Academic Societies Annual Meeting 2005 May 14-17 Washington DC, USA. 2005:Abstract no: 2353 |
| 1. Blake SM, Murray KD, El-Khorazaty MN, Gantz MG, Kiely M, Best D, et al. Environmental tobacco smoke avoidance among pregnant African-American nonsmokers. American Journal of Preventive Medicine 200936(3): 225–34 |
| 1. Blalock JA, Fouladi RT, Wetter DW, Cinciripini PM. Depression in pregnant women seeking smoking cessation treatment. Addictive Behaviours 200530(6):1195–208 |
| 1. Blalock JA, Robinson JD, Wetter DW, Cinciripini PM. Relationship of DSM-IV-Based depressive disorders to smoking cessation and smoking reduction in pregnant women. American Journal on Addictions 200615(4): 268–77 |
| 1. Bonollo DP, Zapka JG, Stoddard AM, Ma Y, Pbert L, Ockene JK. Treating nicotine dependence during pregnancy and postpartum: understanding clinician knowledge and performance. Patient Education and Counselling 200248: 265–74 |
| 1. Bradizza, C. M., Stasiewicz, P. R., Zhuo, Y., Ruszczyk, M., Maisto, S. A., Lucke, J. F., . Giarratano, P. (2017). Smoking cessation for pregnant smokers: Development and pilot test of an emotion regulation treatment supplement to standard smoking cessation for negative affect smokers. Nicotine & Tobacco Research, 19(5), 578–584 |
| 1. Brandon, T. H., Simmons, V. N., Meade, C. D., et al. (2012). Self-help booklets for preventing postpartum smoking relapse: A randomized trial. American Journal of Public Health, 102(11), 2109–2115 |
| 1. Bryce, A., Butler, C., Gnich, W., Sheehy, C., & Tappin, D. CATCH: development of a home-based midwifery intervention to support young pregnant smokers to quit. Midwifery. 2009 25(5): 473-482. PMid:18280015 http://dx.doi.org/10.1016/j.midw.2007.10.006 |
| 1. Bullock L, Everett KD, Mullen PD, Geden E, Longo DR, Madsen R. Baby BEEP: A randomized controlled trial of nurses’ individualized social support for poor rural pregnant smokers. Maternal and Child Health Journal 200913(3): 395–406 |
| 1. Bullock LF, Everett KD, Mullen PD. Baby beep: a randomized clinical trial of smoking cessation for low- income rural pregnant women using nurse-delivered social support. Annals of Behavioral Medicine 200835:S99 |
| 1. Bullock LF, Hornblow AR, Duff GB, Wells JE. Telephone support for pregnant women: outcome in late pregnancy. New Zealand Medical Journal 1995108:476–8 |
| 1. Burling TA, Bigelow GE, Robinson JC, Mead AM. Smoking during pregnancy: reduction via objective assessment and directive advice. Behavior Therapy 199122:31–40 |
| 1. Byrd JC, Meade CD. Smoking cessation among pregnant women in an urban setting. Wisconsin Medical Journal 199392:609–12 |
| 1. Calderón SH, Gilbert P, Jackson R, Kohn MA, Gerbert B. Cueing prenatal providers: effects on discussions of intimate partner violence. American Journal of Preventive Medicine 200834(2):134-7 |
| 1. Campbell E, Walsh RA, Sanson-Fisher, Burrows S, Stojanovski E. A group randomised trial of two methods for disseminating a smoking cessation programme to public antenatal clinics: effects on patient outcomes. Tobacco Control 200615(2):97–102 |
| 1. Campion , P. , Owen , L. , McNeill , A. , & McGuire , C. ( 1994 ). Evaluation of a mass media campaign on smoking and pregnancy . Addiction , 89 , 1245 – 1254 . doi:10.1111/j.1360- 0443.1994.tb03303.x |
| 1. Carroll, K. M., Chang, G., Behr, H., Clinton, B., & Kosten, T. R. (1995). Improving treatment outcome in pregnant, methadone-maintained women: Results from a randomized clinical trial. The American Journal on Addictions, 4(1), 56-59. Doi: 10.1111/j.1521-0391.1995.tb00259.x |
| 1. Chang, G., Carroll, K. M., Behr, H. M., & Kosten, T. R. (1992). Improving treatment outcome in pregnant opiate-dependent women. Journal of Substance Abuse Treatment, 9(4), 327-330 |
| 1. Cinciripini PM, Blalock JA, Minnix JA, Robinson JD, Brown VL, Lam C, et al. Effects of an intensive depression- focused intervention for smoking cessation in pregnancy. Journal of Consulting & Clinical Psychology 201078(1): 44–54. |
| 1. Cinciripini PM, McClure JB, Wetter DW, Perry J, Blalock JA, Cinciripini LG, et al. An evaluation of videotaped vignettes for smoking cessation and relapse prevention during pregnancy: The Very Important Pregnant Smokers (VIPS) Program. Tobacco Control 20009(3):iii61–iii63 |
| 1. Cook C, Ward S, Myers S, Spinnato J. A prospective, randomized evaluation of intensified therapy for smoking reduction in pregnancy. American Journal of Obstetrics and Gynecology 1995172:290 |
| 1. Cooke M, Mattick R, Campbell E. The influence of individual and organisational factors on the reported smoking intervention practices of staff in 20 antenatal clinics. Drug and Alcohol Review 199817(2):175–85 |
| 1. Cooke M, Mattick RP, Campbell E. A description of the adoption of the ’Fresh Start’ smoking cessation program by antenatal clinic managers. Australian Journal of Advanced Nursing 200018(1):13–21 |
| 1. Cooke M, Mattick RP, Campbell E. The dissemination?of a smoking cessation program to 23 antenatal clinics:?the predictors of initial program adoption by managers. Australian and New Zealand Journal of Public Health 1999 23(1):99–103 |
| 1. Cooke M, Mattick RP, Walsh RA. Differential uptake of a smoking cessation programme disseminated to doctors and midwives in antenatal in antenatal clinics. Addiction 2001 96(3):495–505 |
| 1. Cooke M, Mattick RP, Walsh RA. Implementation of the ’Fresh Start’ smoking cessation programme to 23 antenatal clinics: a randomized controlled trial investigating two methods of dissemination. Drug and Alcohol Review 2001 20:19–28 |
| 1. Cooper S, Foster K, Naughton F, et al. Pilot study to evaluate a tailored text message intervention for pregnant smokers (MiQuit): study protocol for a randomised controlled trial. Trials. 2015;16:29. 29 |
| 1. Cope G, Nayyar P, Holder R, Gibbons J, Brunce R. A simple near patient test for nicotine and its metabolites in urine to assess smoking habit. Clinical Chimica Acta 1996 256:135–49.?Cope GF. Smoking status and pregnancy: point of care cotinine test. BMJ 2009339:b5652 |
| 1. Cope GF, Nayyar P, Holder R. Feedback from a point- of-care test for nicotine intake to reduce smoking during pregnancy. Annals of Clinical Biochemistry 200340(Pt 6): 674–9 |
| 1. Cope GF, Nayyar P, Holder R. Measurement of nicotine intake in pregnant women - associations to changes in blood cell count. Nicotine & Tobacco Research 20013(2):119–22 |
| 1. Crittenden, KS, Manfredi C, Cho YI, Dolecek TA. Smoking cessation processes in low-SES women: the impact of time- varying pregnancy status, healthcare messages, stress and health concerns. Addictive Behaviors 200732:1347–66 |
| 1. Curry SJ, McBride C, Grothus L, Lando H, Pirie P. Motivation for smoking cessation among pregnant women. Psychology of Addictive Behaviors 200115(2):126–32 |
| 1. de Vries , H. , Bakker , M. , Mullen , P. D. , & van Breukelen , G. ( 2006 ). The effects of smoking cessation counseling by midwives on Dutch pregnant women and their partners . Patient Education and Counseling , 63 , 177 – 188 . doi:10.1016/j.pec.2005.10.002 |
| 1. Donatelle RJ, Hudson D, Dobie S, Goodall A, Hunsberger M, Oswald K. Incentives in smoking cessation: status of the field and implications for research and practice with pregnant smokers. Nicotine and Tobacco Research 20046 (S2):S163–S179 |
| 1. Donatelle RJ, Hudson D. Using 5 A’s and incentives to promote prenatal smoking cessation. National Conference of Tobacco or Health 2002 November 19-21 San Francisco, California, USA. 2002 |
| 1. Donatelle RJ, Prows SL, Champeau D, Hudson D. Randomised controlled trial using social support and financial incentives for high risk pregnant smokers: Significant Other Supporter (SOS) program. Tobacco Control 20009 Suppl 3:iii67–iii69 |
| 1. Donovan J. Randomised controlled trial of anti-smoking advice in pregnancy. British Journal of Preventive and Social Medicine 197731(1):6–12 |
| 1. Donovan JW, Burgess PL, Hossack CM, Yudkin GD. Routine advice against smoking in pregnancy. Journal of the Royal College of General Practitioners 197525(153):264–8 |
| 1. Donovan JW. Randomised controlled trial of anti-smoking advice in pregnancy. Journal of Epidemiology and Community Health 199650(3):232–6 |
| 1. Dornelas EA, Magnavita J, Beazoglou T, Fischer EH, Oncken C, Lando H, et al. Efficacy and cost-effectiveness of a clinic-based counseling intervention tested in an ethnically diverse sample of pregnant smokers. Patient Education and Counseling 200664(1-3):342–9 |
| 1. Dunkley J. Training midwives to help pregnant women stop smoking. Nursing Times 199793(5):64–6 |
| 1. Eades SJ, Sanson-Fisher RW, Wenitong M, Panaretto?K, D’Este C, Gilligan C, et al. An intensive smoking intervention for pregnant Aboriginal and Torres Strait Islander women: a randomised controlled trial. Medical Journal of Australia 2012197(1):42–6 |
| 1. Edwards, M. J., Geiser, T., Chafin, C., Weatherby, N. L., & Smith, C. S.M.A.R.T. mothers are resisting tobacco: Prenatal smoking cessation in WIC mothers. Journal of Allied Heatlh. 2009 38(3): 170-176. Available from: MEDLINE database PMid:19753429 |
| 1. Elk, R., Mangus, L., Rhoades, H., Andres, R., & Grabowski, J. (1998). Cessation of cocaine use during pregnancy: effects of contingency management interventions on maintaining abstinence and complying with prenatal care. Addictive Behaviors, 23(1), 57-64 |
| 1. Elk, R., Schmitz, J., Spiga, R., Rhoades, H., Andres, R., & Grabowski, J. (1995). Behavioral treatment of cocaine-dependent pregnant women and TB-exposed patients. Addictive Behaviors, 20(4), 533-542 |
| 1. El-Khorazaty MN, Johnson AA, Kiely M, El-Mohandes AA, Subramanian S, Laryea HA, et al. Recruitment and retention of low-income minority women in a behavioral intervention to reduce smoking, depression, and intimate partner violence during pregnancy. BMC Public Health 20077:233. [PUBMED: 17822526] |
| 1. El-Mohandes AA, El-Khorazaty MN, Kiely M, Gantz MG. Smoking cessation and relapse among pregnant African-American smokers in Washington, DC. Maternal & Child Health Journal 201115 Suppl 1:S96–S105. El-Mohandes AA, Kiely M, Blake SM, Gantz MG, El- Khorazaty MN. An intervention to reduce environmental tobacco smoke exposure improves pregnancy outcomes. Pediatrics 2010125(4):721–8 |
| 1. El-Mohandes AA, Kiely M, Joseph JG, Subramanian S, Johnson AA, Blake SM, et al. An intervention to improve postpartum outcomes in African-American mothers: a randomized controlled trial. Obstetrics & Gynecology 2008 112(3):611–20 |
| 1. El-Mohandes AA, Windsor R, Tan S, Perry DC, Gantz MG, Kiely M. A randomized clinical trial of trans-dermal nicotine replacement in pregnant African-American smokers. Maternal and Child Health Journal. 2012 17(5):897–906.10.1007/s10995-012-1069-9 |
| 1. El-Mohandes AAE, for the NIH-DC initiative to reduce infant mortality. An integrated psycho-behavioral intervention during pregnancy has significant effects in reducing risks during the post-partum period in African- American women. Pediatric Academic Societies Annual Meeting 2005 May 14-17 Washington DC, USA. 2005: Abstract no: 39 |
| 1. El-Mohandes AAE, Kiely M, Gantz MG, El-Khorazaty?N. A multiple risk factor behavioral intervention reduces environmental tobacco smoke exposure. Pediatric Academic Societies Annual Meeting 2007 May 5-8 Toronto, Canada 2007 |
| 1. El-Mohandes AAE. A psycho-behavioral intervention?on African American pregnant women with a history of intimate partner violence (IPV) improves birth weight distribution of their newborns [abstract]. Pediatric Academic Societies Annual Meeting 2006 April 29-May 2 San Francisco, CA, USA. 2006 |
| 1. El-Mohandes AAE. An integrated behavioral intervention reduces rates of moderate and extreme prematurity in African American (AA) mothers with a history of smoking during pregnancy [abstract]. Pediatric Academic Societies Annual Meeting 2006 April 29-May 2 San Francisco, CA, USA. 2006 |
| 1. El-Mohandes AEE, Kiely M, Gantz MG, El-Khorazaty N. Very preterm birth is reduced in women receiving an integrated behavioural intervention: A randomized controlled trial. Maternal Child Health Journal 201115: 19–28 |
| 1. El-Mohandes, A. A., El-Khorazaty, M. N., Kiely, M., & Gantz, M. G. (2011). Smoking cessation and relapse among pregnant African-American smokers in Washington, DC. Maternal and Child Health Journal, 15(Suppl 1), S96–S105 |
| 1. England LJ, Kendrick JS, Wilson HG, Merritt RK, Gargiullo PM, Zahniser SC. Effects of smoking reduction during pregnancy on the birth weight of term infants. American Journal of Epidemiology 2001154:694–701 |
| 1. Ershoff D. H., Quinn V. P., Patricia D. M. Relapse prevention among women who stop smoking early in pregnancy: a randomized clinical trial of a self-help intervention. Am J Prev Med 1995 11: 178–84 |
| 1. Ershoff DH, Lairson DR, Mullen PD, Quinn VP. Pregnancy and medical cost outcomes of a self-help prenatal smoking cessation program in an HMO. Public Health Reports 1990 105(4):340–7 |
| 1. Ershoff DH, Quinn VP, Boyd NR, Stern J, Gregory M, Wirtschafter D. The Kaiser Permanente prenatal smoking cessation trial. When more isn’t better, what is enough?. American Journal of Preventive Medicine 199917(3):161–8 |
| 1. Ershoff DH, Quinn VP, Boyd NR, Stern J, Gregory M, Wirtschafter D. The Kaiser Permanente prenatal smoking cessation trial: when more isn’t better, what is enough?. Tobacco Control 20009(Suppl 3):iii60 |
| 1. Ershoff DH, Quinn VP, Mullen PD. A randomized trial of a serialized self-help smoking cessation program for pregnant women in an HMO. American Journal of Public Health 198979(2):182–7 |
| 1. Ershoff DH, Solomon LJ, Dolan-Mullen P. Predictors of intentions to stop smoking early in prenatal care. Tobacco Control 20009(3):41 |
| 1. Ewigman B, Crane J, Frigoletto F, LeFevre M, Bain R, McNellis D. Effect of prenatal ultrasound screening on perinatal outcome. New England Journal of Medicine 1993 329:821–9 |
| 1. Ferreira-Borges, C. Effectiveness of a brief counseling and behavioral intervention for smoking cessation in pregnant women. Preventive Medicine. 2005 41(1): 295-302. PMid:15917025 http://dx.doi.org/10.1016/j.ypmed.2004.11.013 |
| 1. Fox NL, Sexton M, Hebel JR, Thompson B. The reliability of self-reports of smoking and alcohol consumption by pregnant women. Addictive Behaviors 198914(2):187–95 |
| 1. Fox NL, Sexton MJ, Hebel JR. Alcohol consumption among pregnant smokers: effects of a smoking cessation intervention program. American Journal of Public Health 198777:211–3 |
| 1. Gadomski, A., Adams, L., Tallman, N., Krupa, N., & Jenkins, P. (2011). Effectiveness of a combined prenatal and postpartum smoking cessation program. Maternal and Child Health Journal, 15(2), 188–197 |
| 1. Gielen AC, Windsor R, Faden RR, O’Campo P, Repke J, Davis M. Evaluation of a smoking cessation intervention for pregnant women in an urban prenatal clinic. Health Education Research 199712(2):247–54 |
| 1. Gilbert P. The health in pregnancy (HIP) study. ClinicalTrials.gov (http://clinicaltrials.gov/) (accessed 20 February 2008) 2008 |
| 1. Gilligan C, Sanson-Fisher R, Eades S, Wenitong M, Panaretto K, D’Este C. Assessing the accuracy of self- reported smoking status and impact of passive smoke exposure among pregnant Aboriginal and Torres Strait Islander women using cotinine biochemical validation. Drug and Alcohol Review 201029:35–40 |
| 1. Gilligan C, Sanson-Fisher RW, D-Este C, Eades S, Wenitong M. Knowledge and attitudes regarding smoking during pregnancy among Aboriginal and Torres Strait Islander women. Medical Journal of Australia 2009190(10): 557–61 |
| 1. Gilligan C. A pilot randomised controlled trial to test the effectiveness of an intervention to help Aboriginal and Torres Strait Islander women to quit smoking during pregnancy: study design and preliminary results [thesis]. Newcastle, Australia: University of Newcastle, 2008 |
| 1. Glover M, Kira A, Walker N, Bauld L. Using incentives to encourage smoking abstinence among pregnant indigenous women? A feasibility study. Maternal and Child Health Journal 2014.19(6):1393–9 |
| 1. Graham AV, Reeb KG, Kitson GC, Zyzanski SJ, Frank SH. A clinical trial to reduce the rate of low birth weight in an inner-city black population. Family Medicine 199224: 439–46 |
| 1. Groff J, Stotts A, Velasquez M, Benjamin-Garner R, Green C, Mastrobattista J. Ultrasound and motivational enhancement for prenatal smoking cessation. Annual Meeting of the Society for Behavioural Medicine 2005 April 13-16 Boston, MA. 2005 |
| 1. Groff JY. Ultrasound and motivational enhancement for prenatal smoking cessation. ClinicalTrials.gov (http:// clinicaltrials.gov/) (accessed March 2006) |
| 1. Haddow JE, Polomak JE, Sepulveda D. Smoking cessation during routine public prenatal care.American Journal of Public Health 199585(10):1451–2 |
| 1. Haddow JE, Wald NJ, Palomaki GE, Kloza EM, Knight GJ. Cotinine-assisted intervention in pregnancy to reduce smoking and low birthweight delivery. British Journal of Obstetrics and Gynaecology 199198(9):859–65 |
| 1. Hajek P., West R., Lee A., Foulds J., Owen L., Eiser J. R. Randomized controlled trial of a midwife-delivered brief smoking cessation intervention in pregnancy. Addiction 2001 96: 485–94 |
| 1. Hamilton BH. Estimating treatment effects in randomized clinical trials with non-compliance: the impact of maternal smoking on birthweight. Health Economics 200110(5): 399–410 |
| 1. Harris M, Reynolds B. A pilot study of home-based smoking cessation programs for rural, Appalachian, pregnant smokers. Journal of Obstetric, Gynecologic, and Neonatal Nursing 2015 [EPub ahead of print] |
| 1. Hartmann K, Thorp J, Pahel-Short L, Koch M. A randomized controlled trial of smoking cessation intervention in pregnancy. American Journal of Obstetrics and Gynecology 1995172:287 |
| 1. Hartmann KE, Koch MA, Pahel-Short L, Thorp JM.?A randomized controlled trial of smoking cessation intervention in pregnancy in an academic clinic. Obstetrics & Gynecology 199687:621–6 |
| 1. Haug K, Fugelli P, Aaro LE, Foss OP. Is smoking intervention in general practice more successful among pregnant than non-pregnant women?. Family Practice 1994 11:111–6 |
| 1. Haug K, Fugelli P, Aaro LE. Recruitment and participation of General Practitioners in a multipractice study of smoking cessation. Scandinavian Journal of Primary Health Care 199210(3):206–10 |
| 1. Haug NA, DiClemente C, Svikis DS. Motivational?enhancement therapy for nicotine dependence in methadone-maintained pregnant women. Psychology of Addictive Behaviours 200418(3):289–92 |
| 1. Hebel JR, Sexton M, Nowicki P. The effect of antismoking intervention during pregnancy: an assessment of interactions with maternal characteristics. American Journal of Epidemiology 198512 |
| 1. Hegaard H, Hjaergaard H, Moller L, Wachmann H, Ottesen B. Multimodel intervention raises smoking cessation rate during pregnancy. Acta Obstetricia et Gynecologica Scandinavica 200382:813–9 |
| 1. Hegaard HK, Kjaergaard H, Moller LF, Wachmann H, Ottesen B. Determination of a saliva cotinine cut-off to distinguish pregnant smokers from pregnanct non-smokers. Acta Obstetricia et Gynecologica 200786:401–6 |
| 1. Hegaard HK, Kjaergaard H, Moller LF, Wachmann H, Ottesen B. Long-term nicotine replacement therapy. British Journal of Midwifery 200412(4):214–20 |
| 1. Heil SH, Higgins ST, Bernstein IM, Solomon LJ, Rogers RE, Thomas CS, et al. Effects of voucher-based incentives on abstinence from cigarette smoking and fetal growth among pregnant women. Addiction 2008103(6):1009–18 |
| 1. Heil SH, Higgins ST, Solomon LJ, Lynch ME, McHale L, Dumeer A, et al. Voucher-based incentives for abstinence from cigarette smoking in pregnant and postpartum women. Society for Research on Nicotine and Tobacco 13th Annual Meeting 2007 Feb 21-24 Austin, Texas. 2007:25, Abstract no: PA6-1 |
| 1. Heil SH, Higgins ST. Characterizing nicotine withdrawal and craving in pregnant cigarette smokers. 66th Annual Scientific Meeting of the College on Problems of Drug Dependence 2004 June 12-17 San Juan, Puerto Rico. 2004 |
| 1. Heil SH, Tidey JW, Holmes HW, Badger GJ, Higgins ST. A contingent payment model of smoking cessation: effects on abstinence and withdrawal. Nicotine and Tobacco Research 20035(2):205–13 |
| 1. Hennrikus D, Pirie P, Hellerstedt W, Lando HA, Steele?J, Dunn C. Increasing support for smoking cessation during pregnancy and postpartum: results of a randomized controlled pilot study. Preventive Medicine 201050(3): 134–7 |
| 1. Herbec A, Brown J, Tombor I, Michie S, West R. Pilot randomized controlled trial of an internet-based smoking cessation intervention for pregnant smokers (’MumsQuit’). Drug & Alcohol Dependence 2014.140:130–6 |
| 1. Hiett A, Brazus S, Hedberg J, Brown H. Smoking cessation program effectiveness during pregnancy. American Journal of Obstetrics and Gynecology 2000182(1 Pt 2):S150 |
| 1. Higgins ST, Bernstein IM, Washio Y, Heil SH, Badger GJ, Skelly JM, et al. Effects of smoking cessation with voucher- based contingency management on birth outcomes. Addiction 2010105:2023–30 |
| 1. Higgins ST, Heil SH, Badger GJ, Mongeon JA, Solomon LJ, McHale L, et al. Biochemical verification of smoking status in pregnant and recently postpartum women. Experimental and Clinical Psychopharmacology 200715(1):58–66 |
| 1. Higgins ST, Heil SH, Dumeer AM, Thomas CS, Solomon LJ, Bernstein IM. Smoking status in the initial weeks of quitting as a predictor of smoking-cessation outcomes in pregnant women. Drug and Alcohol Dependence 200685: 138–41 |
| 1. Higgins ST, Heil SH, Solomon LJ, Bernstein IM, Lussier JP, Abel RL, et al. A pilot study on voucher-based incentives to promote abstinence from cigarette smoking during pregnancy and postpartum. Nicotine and Tobacco Research 20046(6):1015–20 |
| 1. Higgins ST, Washio Y, Lopez AA, Heil SH, Solomon LJ, Lynch ME, et al. Examining two different schedules of financial incentives for smoking cessation among pregnant women. Preventive Medicine 2014 Vol 68:51–7 |
| 1. Higgins TM, Higgins ST, Heil SH, Badger GJ, Skelly JM, Bernstein IM, et al. Effects of cigarette smoking cessation on breastfeeding duration. Nicotine & Tobacco Research 201012(5):483–8 |
| 1. Hjalmarson AIM, Svanberg B, Hahn L. Stopping smoking in pregnancy: effect of a self-help manual in a controlled trial. British Journal of Obstetrics and Gynaecology 199198: 260–4.?Svanberg B. Smoking during pregnancy: possibilities of prevention in antenatal care. International Journal of Technology Assessment in Health Care 19928(Suppl 1): 96–100 |
| 1. Hjermann I, Velve Byre K, Holme I, Leren P. Effect of diet and smoking intervention on the incidence of coronary heart disease: report from the Oslo Study Group or a randomized trial in healthy men. Lancet. 1981;12: 1303–10 |
| 1. Hughes E, Lamont D, Beecroft M, Wilson D. Randomized trial of a “stage-of-change” orientated smoking cessation intervention in infertile and pregnant women. Fertility and Sterility 200074(3):498–503 |
| 1. Hughes EG, Beecroft ML, Lamont D, Rice S, Wilson D, Freebury M, et al. A randomised controlled trial of a “State- of Change” smoking cessation intervention for subfertile and pregnant patients. Fertility and Sterility 199972(3 Suppl 1):S61–S62 |
| 1. Ierfino, D., Mantzari, E., Hirst, J., Jones, T., Aveyard, P., & Marteau, T. M. (2015). Financial incentives for smoking cessation in pregnancy: a single-arm intervention study assessing cessation and gaming. Addiction, 110(4), 680-688. doi: 10.1111/add.12817 |
| 1. Jaakkola, N., Zahlsen, K., & Jaakkola, J. K. Effects of a population-based smoking cessation programme on smoking in pregnancy. European Journal of Public Health. 2001 11(4): 446-449. PMid:11766488 http://dx.doi.org/10.1093/eurpub/11.4.446 |
| 1. Jones, H. E., Haug, N., Silverman, K., Stitzer, M., & Svikis, D. (2000). Improving treatment outcomes for pregnant drug-dependent women using low-magnitude voucher incentives Addictive Behaviors, 25(2), 263-267 |
| 1. Jones, H. E., Haug, N., Silverman, K., Stitzer, M., & Svikis, D. (2001). The effectiveness of incentives in enhancing treatment attendance and drug abstinence in methadone-maintained pregnant women. Drug and Alcohol Dependence, 61(3), 297-306 |
| 1. Jones, H. E., Svikis, D., & Tran, G. (2002). Patient compliance and maternal/infant outcomes in pregnant drug-using women. Substance Use & Misuse, 37(11), 1411-1422 |
| 1. Jones, H. E., Svikis, D., Rosado, J., Tuten, M., & Kulstad, J. L. (2004). What if they do not want treatment?: Lessons learned from intervention studies of non-treatment seeking, drug-using pregnant women. American Journal on Addictions, 13(4), 342-357 |
| 1. Joseph J, for NIH-DC initiative to reduce infant mortality. Randomized trial to reduce 4 behaviors linked to adverse pregnancy outcomes among 1048 inner-city African American women [abstract]. Pediatric Academic Societies Annual Meeting 2005 May 14-17 Washington DC, USA. 2005:Abstract no: 1701 |
| 1. Joseph JG, El-Mohandes AA, Kiely M, El-Khorazaty MN, Gantz MG, Johnson AA, et al. Reducing psychosocial and behavioral pregnancy risk factors: results of a randomized clinical trial among high-risk pregnant African American women. American Journal of Public Health 200999(6): 1053–61 |
| 1. Kataray, G., Kublay, G., & Emiroglu, O. N. Effect of motivational interviewing on smoking cessation in pregnant women. Journal of Advanced Nursing. 2010 66(6): 1328-1337. http://dx.doi.org/10.1111/j.1365-2648.2010.05267.x |
| 1. Katz, KS, Blake SM, Milligan RA, Sharps PW, White?DB, Rodan MF, et al. The design, implementation and acceptability of an integrated intervention to address multiple behavioural and psychosocial risk factors among pregnant African American women. BMC Pregnancy and Childbirth 20088(22):doi:10.1186/1471–2393-8-22 |
| 1. Kemp L, Harris E, McMahon C, Matthey S, Vimpani G, Anderson T, Schmied V, Aslam H, Zapart S. Child and family outcomes of a long-term nurse home visitation programme: a randomised controlled trial. Archives of Disease in Childhood 2011.96(6):533–540 |
| 1. Kendrick JS, Metzger RW, Sexton M, Spierto FW, Floyd RL, Gargiullo PM, et al. Integrating smoking cessation into routine public prenatal care: the Smoking Cessation in Pregnancy project. American Journal of Public Health 1995 85:217–22 |
| 1. Ker, M., Leischow, S., Markowitz, I. B., & Merikle, E. (1996). Involuntary smoking cessation: a treatment option in chemical dependency programs for women and children. Journal of Psychoactive Drugs, 28(1), 47-60. doi: 10.1080/02791072.1996.10471714 |
| 1. Kiely M, El-Khorazaty MN, El-Mohandes AAE. Depression and smoking during pregnancy impact the efficacy of an integral behavioral intervention to resolve risks. Pediatric Academic Societies Annual Meeting 2007 May 5-8 Toronto, Canada 2007 |
| 1. Lando HA, Valanis BG, Lichtenstein E, Curry SJ, McBride CM, Pirie PL, et al. Promoting smoking abstinence in pregnant and postpartum patients: a comparison of 2 approaches. American Journal of Managed Care 20017: 685–93 |
| 1. Lawrence T, Aveyard P, Cheng KK, Griffin C, Johnson C, Croghan E. Does stage-based smoking advice in pregnancy result in long-term quitters? 18-month post-partum follow up of a randomised controlled trial. Society for Research on Nicotine and Tobacco 11th Annual Meeting 2005 March 20-23 Prague, Czech Republic. 2005 |
| 1. Lawrence T, Aveyard P, Cheng KK, Griffin C, Johnson C, Croghan E. Does stage-based smoking cessation advice in pregnancy result in long term quitters? 18-month postpartum follow-up of a randomized controlled trial. Addiction 2005100:107–16 |
| 1. Lawrence T, Aveyard P, Croghan E. What happens to women’s self-reported cigarette consumption and urinary cotinine levels in pregnancy?. Addiction 200398:1315–20 |
| 1. Lawrence T, Aveyard P, Evans O, Cheng KK. A cluster randomised controlled trial of smoking cessation in pregnant women comparing interventions based on the transtheoretical (stages of change) model to standard care. Tobacco Control 200312:168–77 |
| 1. Lee M, Miller SM, Wen KY, Hui SA, Roussi P, Hernandez E. Cognitive-behavioral intervention to promote smoking cessation for pregnant and postpartum inner city women. Journal of Behavioral Medicine 2015.38(6):923–43 |
| 1. LeFevre ML, Ewigman B, Evans JK. Is smoking an indication for prenatal ultrasonography? RADIUS Study Group. Archives of Family Medicine 19954:120–3 |
| 1. Lilley J, Forster DP. A randomised controlled trial of individual counselling of smokers in pregnancy. Public Health 1986100:309–15 |
| 1. Lillington L, Chlebowski R, Ruvalcaba M, Novak D, Royce J. Evaluation of a smoking cessation program for pregnant minority smokers. Cancer Practice 19953(3):157–63 |
| 1. Linares Scott TJ, Heil SH, Higgins ST, Badger GJ, Bernstein IM. Depressive symptoms predict smoking status among pregnant women. Addictive Behaviours 200934:705–8 |
| 1. Loeb BK, Waage G, Bailey J. Smoking intervention in pregnancy. Proceedings of the Fifth World Conference on Smoking and Health 1983 July Winnipeg, Canada. Ottawa: Canadian Council on Smoking and Health, 1983: 389–95 |
| 1. Loukopoulou A, Vardavas C, Tzatzarakis M, Farmakides G, Rossolymos C, Chrelias C, et al. Urinary nicotine and cotinine as a method for assessing the effectiveness of an intervention to aid smoking cessation during pregnancy. Chest 2012.142(4 Suppl 1):1081A |
| 1. Lowe J. B., Balanda K. P., Clare G. Evaluation of antenatal smoking cessation programs for pregnant women. Aust NZ J Public Health 1998 22: 55–9 |
| 1. Lowe J. B., Windsor R., Balanda K. P., Woodby L. Smoking relapse prevention methods for pregnant women: a formative evaluation. Am J Health Promot 1997 11: 244–6 |
| 1. Lowe JB, Balanda KP, Stanton WR, Del Mar C, O’Connor V. Dissemination of an efficacious antenatal smoking cessation program in public hospitals in Australia: a randomised controlled trial. Health Education & Behavior 200229(5):608–19 |
| 1. Lussier JP, Heil SH, Mongeon JA, Badger GJ, Higgins ST. A meta-analysis of voucher-based reinforcement therapy for substance use disorders. Addiction 2006101:192–203 |
| 1. Ma Y, Goins KV, Pbert L, Ockene JK. Predictors of smoking cessation in pregnancy and maintenance postpartum in low- income women. Maternal and Child Health Journal 20059 (4):393–402 |
| 1. MacArthur C, Knox EG, Lancashire RJ. Effects at age nine of maternal smoking in pregnancy: experimental and observational findings. BJOG. 2001;108(1):67–73 |
| 1. Malchodi CS, Oncken C, Dornelas EA, Caramanica L, Gregonis E. The effects of peer counselling on smoking cessation and reduction. Obstetrics & Gynecology 2003101 (3):504–10 |
| 1. Malchodi, J. B., Filho, P. V., Peterson, G. O., & Chatkin, J. M. Quantitative effects of tobacco smoking exposure on the maternal-fetal circulation. Biomed Central. 2011 11(24): 1-6 |
| 1. Manfredi C, Cho YI, Warnecke R, Saunders S, Sullivan M. Dissemination strategies to improve implementation of the PHS smoking cessation guideline in MCH public health clinics: experimental evaluation results and contextual factors. Health Education Research 201126(2):348–60 |
| 1. Manfredi C, Crittenden KS, Cho YI, Gao S. Long-term effects (up to 18 months) of a smoking cessation program among women smokers in public health clinics. Preventive Medicine 200438:10–9 |
| 1. Manfredi C, Crittenden KS, Warnecke R, Engler J, Cho YI, Shaligram C. Evaluation of a motivational smoking cessation intervention for women in public health clinics. Preventive Medicine 199928:51–6 |
| 1. Mauriello L, Dyment S, Prochaska J, Gagliardi A, Weingrad-Smith J. Acceptability and feasibility of a multiple-behaviour, computer-tailored intervention for underserved pregnant women. Journal of Midwifery and Women’s Health 2011.56:75–80 |
| 1. Mayer JP, Todd R, Hawkins B. A randomised evaluation of smoking cessation interventions for pregnant women at a WIC clinic. American Journal of Public Health 199080: 76–7 |
| 1. McBride C. M., Baucom D. H., Peterson B. L., Pollak K. I., Palmer C., Westman E. et al. Prenatal and postpartum smoking abstinence a partner-assisted approach. Am J Prev Med 2004 27: 232–8 |
| 1. McBride C. M., Curry S. J., Lando H. A., Pirie P. L., Grothaus L. C., Nelson J. C. Prevention of relapse in women who quit smoking during pregnancy. Am J Public Health 1999 89: 706–11 |
| 1. McBride CM, Curry SJ, Grothaus LC, Nelson JC. Partner smoking status and pregnant smokers’ perception of support for and likelihood of smoking cessation. Health Psychology 199817:63–9 |
| 1. McGowan, A., Hamilton, S., Barnett, D., Nsofor, M., Proudfoot, J., & Tappin, D. 'Breathe': the stop smoking service for pregnant women in Glasgow. Midwifery. 2010 26(3): 1-13. PMid:18692285 http://dx.doi.org/10.1016/j.midw.2008.05.005 |
| 1. McIntyre-Kingsolver K, Lichtenstein E, Mermelstein RJ. Spouse training in a multicomponent smoking-cessation program. Behav Ther. 1986;17: 67–74 |
| 1. McLeod D, Benn C, Pullon S, Viccars A, White S, Cookson T, et al. The midwife’s role in facilitating smoking behaviour change during pregnancy. Midwifery 200319(4):285–97 |
| 1. McLeod D, Pullon S, Benn C, Cookson T, Dowell A, Viccars A, et al. Can support and education for smoking cessation and reduction be provided effectively by midwives within primary maternity care?. Midwifery 200420:37–50 |
| 1. Mejdoubi J, van den Heijkant SCCM, van Leerdam FJM, Crone M, Crijnen A, HiraSing RA. Effects of nurse home visitation on cigarette smoking pregnancy outcomes: a randomized controlled trial. Midwifery 2014.30:688–95 |
| 1. Messimer SR, Henry RC, Hickner JM. A comparison of two antismoking interventions among pregnant women in eleven primary care practices. Journal of Family Practice 198928(3):283–8 |
| 1. Moore LO, Campbell R, Whelan A, Mills N, Lupton?P, Misselbrook E, et al. Self help smoking cessation in pregnancy: cluster randomised controlled trial. BMJ 2002 325:1383–6 |
| 1. Moore ML, Meis PJ, Ernest JM, Wells HB, Zaccaro DJ, Terrell T. A randomized trial of nurse intervention to reduce preterm and low birth weight births. Obstetrics & Gynecology 199891:656–61 |
| 1. Moore ML, Zaccaro DJ. Cigarette smoking, low birth weight, and preterm births in low-income African-American women. Journal of Perinatology 20003:176–80 |
| 1. Morasco B. J., Dornelas E. A., Fischer E. H., Oncken C., Lando H. A. Spontaneous smoking cessation during pregnancy among ethnic minority women: a preliminary investigation. Addict Behav 2006 31: 203–10 |
| 1. Mullen, P. D., Quinn, V. P., & Ershoff, D. H. (1990). Maintenance of nonsmoking postpartum by women who stopped smoking during pregnancy. American Journal of Public Health, 80(8), 992–994 |
| 1. Naughton F, Jamison J, Sutton S. Attitudes towards SMS text message smoking cessation support: a qualitative study of pregnant smokers. Health Educ Res. 2013;28(5):911–922. doi: 10.1093/her/cyt057 |
| 1. Naughton F, Prevost AT, Gilbert H, Sutton S. Randomized controlled trial evaluation of a tailored leaflet and SMS text message self-help intervention for pregnant smokers (MiQuit). Nicotine and Tobacco Research 201214(5): 569–77 |
| 1. Naughton, F., Cooper, S., Foster, K., Emery, J., Leonardi-Bee, J., Sutton, S., … Leighton, M. (2017). Large multi-centre pilot randomized controlled trial testing a low-cost, tailored, self-help smoking cessation text message intervention for pregnant smokers (MiQuit). Addiction, 112(7), 1238–1249. doi:10.1093/ntr/ntr254 |
| 1. Nowicki P, Gintzig L, Hebel JR, Lathem R, Miller V, Sexton M. Effective smoking intervention during pregnancy. Birth 198411:217–24 |
| 1. Nyborg KF, Nevid JS. Couples who smoke: a comparison of couples training versus individual training for smoking cessation. Behav Ther. 1986;17:620–5. |
| 1. O’Connor, A. M., Davies, B. L., Dulberg, C. S., et al. (1992). Effectiveness of a pregnancy smoking cessation program. Journal of Obstetric, Gynecologic, and Neonatal Nursing, 21(5), 385–392 |
| 1. Øien, T., Storro, O., Jenssen, J. A., Johnsen, R. The impact of a minimal smoking cessation intervention for pregnant women and their partners on a perinatal smoking behavior in primary health care: A real-life controlled study. BMC Public Health. 2008 8: 325-335. PMid:18808705 http://dx.doi.org/10.1186/1471-2458-8-325 |
| 1. Olds D, Robinson J, O’Brien R, Luckey D, Pettit L, Henderson C, et al. Home visiting by paraprofessionals and by nurses: a randomized, controlled trial. Pediatrics 2002 110(3):486–96 |
| 1. Olds DL, Henderson CR, Chamberlin R, Tatelbaum R. Preventing child abuse and neglect: a randomized trial of nurse home visitation. Pediatrics 198678:65–78 |
| 1. Olds DL, Henderson CR, Tatelbaum R, Chamberlin R. Improving the delivery of prenatal care and outcomes of pregnancy: a randomized trial of nurse home visitation. Pediatrics 198677:16–28 |
| 1. Olds DL, Henderson CR, Tatelbaum R. Prevention of intellectual impairment in children of women who smoke cigarettes during pregnancy. Pediatrics 199493:228–33 |
| 1. Oncken C, Dornelas E, Greene J, et al. Nicotine gum for pregnant smokers: a randomized controlled trial. Obstet Gynecol. 2008;112(4):859–867 |
| 1. Ondersma S, Svikis DS, Beatty JR, Lockhart N. A randomized clinical trial of a computer delivered brief intervention for post-partum drug, alcohol, and tobacco use: three-month outcomes. Proceedings of the 73rd Annual Scientific Meeting of the College on Problems of Drug Dependence 2011 June 18-23, Hollywood, Florida. 2011 Vol. http://www.cpdd.vcu.edu/Pages/Meetings/ Meetings ?PDFs/2011Programbook.pdf. Accessed 22/8/ 2013:134 , Abstract no: 534.?Ondersma SJ. Computer-assisted Intervention for smoking during pregnancy (HPP). ClinicalTrials.gov 2009 |
| 1. Ondersma SJ, Chase SK, Svikis DS, Schuster CR. Computer-based brief motivational intervention for perinatal drug use. Journal of Substance Abuse Treatment 200528:305–12 |
| 1. Ondersma SJ, Svikis DS, Lam PK, Connors-Burge VS, Ledgerwood DM, Hopper JA. A randomized trial of computer-delivered brief intervention and low-intensity contingency management for smoking during pregnancy. Nicotine & Tobacco Research 2012 Vol. 14, issue 3: 351–6 |
| 1. Oude Wesselink SF, Lingsma HF, Reulings PG, Wentzel HR, Erasmus V, Robben PB, et al. Does government supervision improve stop-smoking counseling in midwifery practices? Nicotine & Tobacco Research 2015 Vol 17, issue 5:572–9 |
| 1. Panaretto KS, Mitchell MR, Anderson L, Gilligan C, Buettner P, Larkins SL, et al. Tobacco use and measuring nicotine dependence among urban Indigenous pregnant women. Medical Journal of Australia 2009191(10):554–7 |
| 1. Panjari M, Bell R, Astbury J, Bishop S, Dalais F, Rice G. Women who spontaneously quit smoking in early pregnancy. Australian New Zealand Journal of Obstetrics and Gynaecology 199737(3):271 |
| 1. Panjari M, Bell R, Bishop S, Astbury J, Rice G, Doery?J. A randomized controlled trial of a smoking cessation intervention during pregnancy. Australian and New Zealand Journal of Obstetrics and Gynaecology 199939(3):312–7 |
| 1. Park ER, Quinn VP, Chang Y, Regan S, Loudin B, Cummins S, et al. Recruiting pregnant smokers into a clinical trial: using a network-model managed care organization versus community-based practices. Preventive Medicine 200744: 223–9 |
| 1. Parker DR, Roberts MB, Windsor RA, Lasater TM. Telephone-based smoking cessation interventions effective in high-risk, underserved pregnant women. Joint Conference of SRNT and SRNT-Europe 2009 April 27-30 Dublin, Ireland. 2099 |
| 1. Parker DR, Windsor RA, Roberts MB, Hecht J,?Hardy NV, Strolla LO, et al. Feasibility, cost, and cost- effectiveness of a telephone-based motivational intervention for underserved pregnant smokers. Nicotine and Tobacco Research 20079(10):1043–51 |
| 1. Patten CA, Hughes CA, Lopez KN, Thomas JL, Brockman TA, Smith CM, et al. Web-based intervention for adolescent nonsmokers to help parents stop smoking: a pilot feasibility study. Addict Behav. 2012;37:85–91 |
| 1. Patten CA, Windsor RA, Renner CC, Enoch C, Hochreiter A, Nevak C, et al. Feasibility of a tobacco cessation intervention for pregnant Alaska Native women. Nicotine & Tobacco Research 200912(2):79–87 |
| 1. Patten CA. Tobacco cessation intervention during pregnancy among Alaska Native women. Journal of Cancer Education 201227(Supp 1):S86–90.?Patten CA. Tobacco cessation treatment for pregnant Alaska natives. ClinicalTrials.gov (http://clinicaltrials.gov/) (accessed 21 June 2007) |
| 1. Pbert L., Ockene J. K., Zapka J.,Ma Y., Goins K. V., Oncken C. et al. A community health center smoking-cessation intervention for pregnant and postpartum women. Am J Prev Med 2004 26: 377–85 |
| 1. Peden, A. R., Rayens, M. K., Hall, L. A., et al. (2008). Nicotine addiction in pregnancy: Preliminary efficacy of a mental health intervention. Addictive Disorders and Their Treatment, 7(4), 179–189 |
| 1. Peden, A. R., Rayens, M. K., Hall, L. A., Hahn, E., Riker, C., Ashford, K., et al. Nicotine addiction in pregnancy: Preliminary efficacy of a mental health intervention. Addictive Disorders & Their Treatment. 2008 7(4): 179-189. http://dx.doi.org/10.1097/ADT.0b013e3181484768 |
| 1. Petersen, L., Handel, J., Kotch, J., Podedworny, T., & Rosen, A. (1992). Smoking reduction during pregnancy by a program of self-help and clinical support. Obstetrics and Gynecology, 79(6), 924–930 |
| 1. Peterson L, Rosen A, Podedworny T, Kotch J, Handel J. Smoking reduction during pregnancy by a program of self- help and clinical support. Obstetrics & Gynecology 199279: 924–30 |
| 1. Polanska K, Hanke W, Sobala W, Lowe JB. Efficacy and effectiveness of the smoking cessation program for pregnant women. International Journal of Occupational Medicine and Environmental Health 200417(3):369–77 |
| 1. Polanska K, Hanke W, Sobala W. Smoking relapse one year after delivery among women who quit smoking during pregnancy. International Journal of Occupational Medicine and Environmental Health 200518(2):159–65 |
| 1. Polanska K, Hanke W, Sobala. Characteristic of the smoking habit among pregnant women on the base of the test “Why am I a smoker?” [Charakterystyka nagogu palenia papierosow wsrod kobiet ciezarnych na podstawie testu Dlaczego pale?]. Przeglad Lekarski 200562(10):1095–8 |
| 1. Pollak KI, Lyna P, Bilheimer A, Farrell D, Gao X, Swamy GK, et al. A pilot study testing SMS text delivered scheduled gradual reduction to pregnant smokers. Nicotine & Tobacco Research 2013.15(10):1773–6 |
| 1. Pollak, K. I., Oncken, C. A., Lipkus, I. M., et al. (2007). Nicotine replacement and behavioral therapy for smoking cessation in pregnancy. American Journal of Preventive Medicine, 33(4), 297–305 |
| 1. Prapavessis H, De Jesus S, Harper T, Cramp A, Fitzgeorge L, Mottola MF, et al. The effects of acute exercise on tobacco cravings and withdrawal symptoms in temporary abstinent pregnant smokers. Addictive Behaviors 2014.39(3):703–8 |
| 1. Price JH, Snyder FF, Roberts SM, Losh DP, Desmond SM, Krol RA. Comparison of three antismoking interventions among pregnant women in an urban setting: a randomized trial. Psychological Reports 199168:595–604 |
| 1. Pullon S, McCleod D, Benn C, Viccars A, White S, Cookson T, et al. Smoking cessation in New Zealand: education and resources for use by midwives for women who smoke during pregnancy. Health Promotion International 200318(4):315–24 |
| 1. Quinn VP, Mullen PD, Ershoff DH. Women who stop smoking spontaneously prior to prenatal care and predictors of relapse before delivery. Addictive Behaviour 199116(1- 2):29–40 |
| 1. RA, Redman S, Byrne JM, Melmeth A, Brinsmead MW. Process measures in an antenatal smoking cessation trial: another part of the picture. Health Education Research 200015(4):469–83 |
| 1. Radley, A., Ballard, P., Eadie, D., MacAskill, S., Donnelly, L., & Tappin, D. (2013). Give it up for baby: Outcomes and factors influencing uptake of a pilot smoking cessation incentive scheme for pregnant women. BMC public health, 13(1), 343. doi: 10.1186/1471-2458-13-343 |
| 1. Ram A, Tuten M, Chisolm MS. Cigarette smoking reduction in pregnant women with opioid use disorder. J Addict Med. 2016;10(1):53–59 |
| 1. Reading AE, Cox DN. The effects of ultrasound on maternal anxiety. Journal of Behavioral Medicine 19825(2): 237–47 |
| 1. Reading AE, Sledmere CM, Cox DNB, Campbell S. Health beliefs and health care behaviour in pregnancy. Psychological Medicine 198212:379–83 |
| 1. Reitzel, L. R., Vidrine, J. I., Businelle, M. S., et al. (2010). Preventing postpartum smoking relapse among diverse lowincome women: A randomized clinical trial. Nicotine & Tobacco Research, 12(4), 326–335 |
| 1. Rigotti N, Park E, Regan S, Chang Y, Perry K, Loudin B, et al. Efficacy of proactive telephone counseling for pregnant smokers: a randomized trial. Society for Research on Nicotine and Tobacco 12th Annual Meeting 2006 February 15-18, Orlando, Florida, USA 2006:22 |
| 1. Rigotti N, Park E, Regan S, Chang Y, Perry K, Loudin B, et al. The efficacy of telephone couseling for pregnant smokers: a randomized controlled trial [abstract]. 13th World Conference on Tobacco or Health 2006 July 12-15 Washington DC, USA. 2006 |
| 1. Rigotti N, Park ER, Regan S, Chang Y, Perry K, Loudin B, et al. Efficacy of telephone counseling for pregnant smokers. Obstetrics & Gynecology 2006108(1):83–92 |
| 1. Rigotti NA, Park ER, Chang Y, Regan S. Smoking cessation medication use among pregnant and postpartum smokers. Obstetrics & Gynecology 2008111(2 Pt 1):348–55 |
| 1. Robling M, Bekkers MJ, Bell K, Butler CC, CanningsJohn R, Channon S, et al. Effectiveness of a nurse-led intensive home-visitation programme for first-time teenage mothers (Building Blocks): a pragmatic randomised controlled trial. Lancet 2016.387(10014):146–55 |
| 1. Ruger J. P.,Weinstein M. C., Hammond S. K., KearneyM. H., Emmons K. M. Cost-effectiveness of motivational interviewing for smoking cessation and relapse prevention among low-income pregnant women: a randomized controlled trial. Value Health 2008 11: 191–8 |
| 1. Schottenfeld, R. S., Moore, B., & Pantalon, M. V. (2011). Contingency management with community reinforcement approach or twelve-step facilitation drug counseling for cocaine dependent pregnant women or women with young children. Drug and Alcohol Dependence, 118(1), 48-55 |
| 1. Secker-Walker R. H., Solomon L. J., Flynn B. S., Skelly J. M. Smoking relapse prevention counseling during prenatal and early postnatal care. AmJ Prev Med 1995 11: 86–93 |
| 1. Secker-Walker R. H., Solomon L. J., Flynn B. S., Skelly J. M., Mead P. B. Smoking relapse prevention during pregnancy. AmJ Prev Med 1998 15: 25–31 |
| 1. Secker-Walker RH, Mead PB, Goodwin GD, Lepage SS, Skelly JM, Flynn BS, et al. Individualised smoking cessation counseling during prenatal and early postnatal care. American Journal of Obstetrics and Gynecology 1994 71:1347–55 |
| 1. Secker-Walker RH, Solomon LJ, Flynn BS, LePage SS, Crammond JE, Worden JK, et al. Training obstetric and family practice residents to give smoking cessation advice during prenatal care. American Journal of Obstetrics and Gynecology 1992166:1356–63 |
| 1. Secker-Walker RH, Solomon LJ, Flynn BS, Skelly JM, Mead PB. Reducing smoking during pregnancy and postpartum: physician’s advice supported by individual counseling. Preventive Medicine 199827:422–30 |
| 1. Secker-Walker RH, Solomon LJ, Geller BM, Flynn BS, Worden JK, Skelly JM, et al. Modeling smoking cessation: exploring the use of a videotape to help pregnant women quit smoking. Women & Health 199725:23–35 |
| 1. Secker-Walker RH, Vacek PM, Flynn BS, Mead PB. Estimated gains in birth weight associated with reductions in smoking during pregnancy. Journal of Reproductive Medicine 199843(11):967–74 |
| 1. Secker-Walker RH, Vacek PM, Flynn BS, Mead PB. Smoking in pregnancy, exhaled carbon monoxide, and birth weight. Obstetrics and Gynecology 199789:648–53 |
| 1. Sexton M, Hebel JR. A clinical trial of change in maternal smoking and its effect on birth weight. JAMA 1984251: 911–5 |
| 1. Sexton M, Nowicki P, Hebel JR. Verification of smoking status by thiocyanate in unrefrigerated, mailed saliva samples. Preventive Medicine 198615(1):28–34 |
| 1. Silverman, K., Svikis, D., Robles, E., Stitzer, M. L., & Bigelow, G. E. (2001). A reinforcement-based therapeutic workplace for the treatment of drug abuse: Sixmonth abstinence outcomes. Experimental and Clinical Psychopharmacology, 9(1), 14-23 |
| 1. Solomon LJ, Higgins ST, Heil SH, Badger GJ, Thomas CS, Bernstein IM. Predictors of postpartum relapse to smoking. Drug and Alcohol Dependence 200790:224–7 |
| 1. Solomon LJ, Secker-Walker RH, Flynn BS, Skelly JM, Capeless EL. Proactive telephone peer support to help pregnant women stop smoking. Tobacco Control 20009 Suppl 3:iii72–iii74 |
| 1. Solomon LJ, Secker-Walker RH, Skelly JM, Flynn BS. Stages of change in smoking during pregnancy in low risk women. Journal of Behavioral Medicine 199619:333–4 |
| 1. Spierto FW, Hannon WH, Kendrick JS, Bernert JT, Pirkle J, Gargiullo PM. Urinary cotinine levels in women enrolled in a smoking cessation study during and after pregnancy. Journal of Smoking-Related Disease 19945:65–76 |
| 1. Stanton WR, Lowe JB, Moffatt J, DelMar CB. Randomised control trial of a smoking cessation intervention directed at men whose partners are pregnant. Preventive Medicine 200438:6–9 |
| 1. Stotts AL, DeLaune KA, Schmitz JM, Grabowski J. Impact of a motivational intervention on mechanisms of change in low-income pregnant women. Addictive Behaviors 200429 (8):1649–57 |
| 1. Stotts AL, Groff JY, Velasquez MM, Benjamin-Garner R, Green C, Carbonari JP, et al. Ultrasound feedback and motivational interviewing targeting smoking cessation in the second and third trimesters of pregnancy. Nicotine & Tobacco Research 200911(8):961–8 |
| 1. Stotts AL, Schmitz JM, Shipley SL, Delaune KA, Grabowski J. Impact of a motivational interviewing intervention on mechanisms of change in low-income pregnant smokers (POS4-32) [abstract]. Society for Research on Nicotine and Tobacco 9th Annual Meeting 2003 February 19-22 New Orleans, Louisiana. 2003:89 |
| 1. Stotts S, DiClemente CC, Dolan-Mullen P. One-to-One: a motivational intervention for resistant pregnant smokers. Addictive Behaviors 200227:275–92 |
| 1. Strecher VJ, Bishop KR, Bernhardt J, Thorp JM, Cheuvrout B, Potts P. Quits for keeps: tailored smoking cessation guides for pregnancy and beyond. Tobacco Control 20009 (Suppl 3):iii78–iii79 |
| 1. Subramanian S, Katz KS, Rodam M, Gantz MG, El- Khorazaty NM, Johnson A, et al. An integrated randomized intervention to reduce behavioural and psychosocial risks: Pregnancy and neonatal outcomes. Maternal Child Health Journal 201216:545–54 |
| 1. Suplee, P. D. (2005). The importance of providing smoking relapse counseling during the postpartum hospitalization. Journal of Obstetrics and Gynecology in Neonatal Nursing, 34, 703–712 |
| 1. Svikis, D. S., Lee, J. H., Haug, N. A., & Stitzer, M. L. (1997). Attendance incentives for outpatient treatment: Effects in methadone and nonmethadone-maintained pregnant drug dependent women. Drug and Alcohol Dependence, 48 33-41 |
| 1. Svikis, D. S., Silverman, K., Haug, N. A., Stitzer, M., & Keyser-Marcus, L. (2007). Behavioral strategies to improve treatment participation and retention by pregnant drug-dependent women. Substance Use & Misuse, 42(10), 1527-1535 |
| 1. Tan S, Courtney LP, El-Mohandes AAE, Gantz MG, Blake SM, Thornberry J, et al. Relationships between self- reported smoking, household environmental tobacco smoke exposure and depressive symptoms in a pregnant minority population. Maternal Child Health Journal 201115:S65- S74 |
| 1. Tappin D, Bauld L, Purves D, Boyd K, Sinclair L, MacAskill S, et al. Financial incentives for smoking cessation in pregnancy: randomised controlled trial. BMJ (Clinical Research Ed.) 2015.350:h134 |
| 1. Tappin DM, Lumsden MA, Gilmour WH, Crawford F, McIntyre D, Stone DH, et al. Randomised controlled trial of home based motivational interviewing by midwives to help pregnant smokers quit or cut down. BMJ 2005331: 373–7. |
| 1. Tappin DM, Lumsden MA, McIntyre D, McKay C, Gilmour WH, Webber R, et al. A pilot study to establish a randomized trial methodology to test the efficacy of a behavioural intervention. Health Education Research 2000 15(4):491–502 |
| 1. Tappin DM, Lumsden MA, McKay C, McIntyre D, Gilmour H, Webber R, et al. The effect of home-based motivational interviewing on the smoking behaviour of pregnant women: a pilot randomised controlled efficacy study. Ambulatory Child Health 20006(Suppl 1):34–5 |
| 1. Thornton L, Gogan C, McKenna P. The rotunda stop smoking programme [abstract]. Irish Journal of Medical Science 1998167(Suppl 9):28 |
| 1. Thornton L. Smoking and pregnancy: feasibility and effectiveness of a smoking intervention programme among pregnant women [thesis]. Dublin: Dept of Public Health, 1997 |
| 1. Tsoh JY, Kohn MA, Gerbert B. Promoting smoking cessation in pregnancy with Video Doctor plus provider cueing: a randomized trial. Acta Obstetricia et Gynecologica Scandinavica 201089(4):515–23 |
| 1. Tuten M, Fitzsimons H, Chisolm MS, Nuzzo PA, Jones HE. Contingent incentives reduce cigarette smoking among pregnant, methadone-maintained women: results of an initial feasibility and efficacy randomized clinical trial. Addiction 2012107(10):1868–77 |
| 1. Tuten, M., Svikis, D. S., Keyser-Marcus, L., O’Grady, K. E., & Jones, H. E. (2012b). Lessons learned from a randomized trial of fixed and escalating contingency management schedules in opioid-dependent pregnant women. The American Journal of Drug and Alcohol Abuse, 38(4), 286-292 |
| 1. Ussher M, Lewis S, Aveyard P, et al. The London Exercise and Pregnant smokers (LEAP) trial: a randomised controlled trial of physical activity for smoking cessation in pregnancy with an economic evaluation. Health Technol Assess. 2015;19(84):vii–xxiv, 1 |
| 1. Ussher M, Lewis S, Aveyard P, Manyonda I, West R, Lewis B, et al. Physical activity for smoking cessation in pregnancy: randomised controlled trial. BMJ (Clinical Research Ed.) 2015.350:h2145 |
| 1. Valanis, B., Lichtenstein, E., Mullooly, J. P., et al. (2001). Maternal smoking cessation and relapse prevention during health care visits. American Journal of Preventive Medicine, 20(1), 1–8 |
| 1. Valbo A, Eide T. Smoking cessation in pregnancy: the effect of hypnosis in a randomized study. Addictive Behaviors 199621:29–35 |
| 1. Valbo A, Nylander G. Smoking cessation in pregnancy: intervention among heavy smokers. Acta Obstetricia et Gynecologica Scandinavica 199473:215–9 |
| 1. Valbo A., Schioldborg P. Smoking cessation in pregnancy: the effect of self-help manuals. J Matern Fetal Invest 1994 4: 167–70 |
| 1. van Mierlo T, Fournier R, Jean-Charles A, Hovington J, Ethier I, Selby P. I’ll txt u if i have a problem: how the Société? Canadienne du cancer in Quebec applied behavior-change theory, data mining and agile software development to help young adults quit smoking. PLoS One. 2014;9(3):e91832. 30 |
| 1. Victora CG, Langer A, Barros F, Belizan J, Farnot U, Villar J, et al. The Latin American Multicenter Trial on psychosocial support during pregnancy: methodology and baseline comparability. Controlled Clinical Trials 199415: 379–94 |
| 1. Vilches P. Consumption of tobacco in pregnant women: Proposal of a psychological intervention model in the public health system of Andalucia. [Consumo de tabaco en mujeres gestantes: propuesta de un modelo de intervención psicológica en el sistema sanitario público de Andalucía]. [Doctoral Thesis]. Univesidad di Malaga, 2009 |
| 1. Villar J, Farnot U, Barros F, Victora C, Langer A, Belizan JM. A randomized trial of psychosocial support during high-risk pregnancies. New England Journal of Medicine 1992327:1266–71 |
| 1. Wall, M. A., Severson, H. H., Andrews, J. A., Lichtenstein, E., & Zoref, L. (1995). Pediatric office-based smoking intervention: Impact on maternal smoking and relapse. Pediatrics, 96(4 Pt 1), 622–628 |
| 1. Walsh RA, Melmeth A, Byrne JM, Brinsmead MW, Redman S. A smoking cessation program at a public antenatal clinic. American Journal of Public Health 199787: 1201–4 |
| 1. Washio Y, Higgins ST, Heil SH, Badger GJ, Skelly J, Bernstein IM, et al. Examining maternal weight gain during contingency-management treatment for smoking cessation among pregnant women. Drug and Alcohol Dependence 2011114(1):73–6 |
| 1. Wilkinson SA, McIntyre HD. Evaluation of the ’healthy start to pregnancy’ early antenatal health promotion workshop: a randomized controlled trial. BMC Pregnancy and Childbirth 2012.0.590972222222222 |
| 1. Windsor R, Woodby L, Miller T, Hardin M. Effectiveness of Smoking Cessation and Reduction in Pregnancy Treatment (SCRIPT) methods in Medicaid-supported prenatal care: Trial III. Health Education & Behavior 201138(4):412–22 |
| 1. Windsor RA, Contreras L, Artz L, Lowe JB. Smoking cessation and pregnancy intervention trial: preliminary mid-trial results. Progress in Clinical and Biological Research 1990339:107–17 |
| 1. Windsor RA, Li CQ, Boyd NR, Hartmann KE. The use of significant reduction rates to evaluate health education methods for pregnant smokers: a new harm reduction behavioural indicator. Health Education and Behavior 1999 26:648–61 |
| 1. Windsor RA, Lowe JB, Perkins LL, Smith-Yoder D, Artz L, Crawford M, et al. Health education for pregnant smokers: its behavioral impact and cost benefit. American Journal of Public Health 199383:201–6 |
| 1. Windsor RA, Spanos D, Samuelsson C, Bartlett EE, Manzella B, Reese Y, et al. The effectiveness of smoking cessation methods for smokers in public health maternity clinics: a randomized trial. American Journal of Public Health 198575:1389–92 |
| 1. Windsor RA, Warner KE, Cutter GR. A cost-effectiveness analysis of self-help smoking cessation methods for pregnant women. Public Health Reports 1988103(1):83–8 |
| 1. Windsor RA. The efficacy and cost-effectiveness of smoking cessation methods for pregnant women. Southern Medical Journal 19863.31527777777778 |
| 1. Wisborg, K., Henriksen, T. B., Jespersen, L. B., & Secher, N. J. (2000). Nicotine patches for pregnant smokers: A randomized controlled study. Obstetrics and Gynecology, 96(6), 967–971 |
| 1. Wood DA, Kotseva K, Connolly S, Jennings C, Mead A, Jones J, Holden A, De Bacquer D, Collier T, De Backer G, Faergeman O, EUROACTION Study Group. Nurse-coordinated multidisciplinary, family based cardiovascular disease prevention programme (EUROACTION) for patients with coronary heart disease and asymptomatic individuals at high risk of cardiovascular disease: a paired, cluster-randomised controlled trial. Lancet. 2008;371:199–2012 |
| 1. Yoon JH, Higgins ST, Heil SH, Sugarbaker RJ, Thomas CS, Badger GJ. Delay discounting predicts postpartum relapse to cigarette smoking among pregnant women. Experimental and Clinical Psychopharmacology 200715(2):176–86 |
| 1. Zapka J, Goins KV, Pbert L, Ockene JK. Translating efficacy research into effectiveness studies in practice: lessons from research to promote smoking cessation in community health centers. Health Promotion Practice 20045(3):245–55 |
| 1. Zapka JG, Pbert L, Stoddard AM, Ockene JK, Goins KV, Bonollo D. Smoking cessation counseling with pregnant and postpartum women: a survey of community health center providers. American Journal of Public Health 2000 90:78–84 |

**S7e: Included studies in diet and/or physical activity reviews**

| **Review** | **References of included behavioural interventions delivered in pregnancy** |
| --- | --- |
| Bain  *et al.* 2015[21] | 1. Asbee SM, Jenkins TR, Butler JR, White J, Elliot M, Rutledge A. Preventing excessive weight gain during pregnancy through dietary and lifestyle counseling: a randomized controlled trial. Obstetrics and Gynecology 2009;113(2 Pt 1):305–12. 2. Dodd JM, Turnbull D, McPhee AJ, Deussen AR, Grivell RM, Yelland LN, et al. Antenatal lifestyle advice for women who are overweight or obese: LIMIT randomised trial. BMJ 2014;348:g1285. 3. El Beltagy N, El Deen SS, Mohamed R. Does physical activity and diet control reduce the risk of developing gestational diabetes mellitus in Egypt? A randomized controlled trial. Journal of Perinatal Medicine 2013;41 (Suppl 1):1176. 4. Harrison CL, Lombard CB, Strauss BJ, Teede HJ. Optimizing healthy gestational weight gain in women at high risk of gestational diabetes: a randomized controlled trial. Obesity 2013;21(5):904–9. 5. Hui A, Back L, Ludwig S, Gardiner P, Sevenhuysen G, Dean H, et al. Lifestyle intervention on diet and exercise reduced excessive gestational weight gain in pregnant women under a randomised controlled trial. BJOG: An International Journal of Obstetrics & Gynaecology 2012;119 (1):70–7. 6. Korpi-Hyovalti EA, Laaksonen DE, Schwab US, Vanhapiha TH, Vihla KR, Heinonen ST, et al. Feasibility of a lifestyle intervention in early pregnancy to prevent deterioration of glucose tolerance. BMC Public Health 2011;11:179. 7. Luoto R, Kinnunen TI, Aittasalo M, Kolu P, Raitanen J, Ojala K, et al. Primary prevention of gestational diabetes mellitus and large-for-gestational-age newborns by lifestyle counseling: a cluster-randomized controlled trial. PLoS Medicine 2011;8(5):1–11. 8. Petrella E, Facchinetti F, Bertarini V, Pignatti L, Neri I, Battistini NC. Occurrence of pregnancy complications in women with BMI > 25 submitted to a healthy lifestyle and eating habits program. American Journal of Obstetrics and Gynecology 2013;208(1 Suppl):S33–4. 9. Phelan S, Phipps MG, Abrams B, Darroch F, Schaffner A, Wing RR. Randomized trial of a behavioral intervention to prevent excessive gestational weight gain: the Fit for Delivery Study. American Journal of Clinical Nutrition 2011;93(4):772–9. 10. Polley BA, Wing RR, Sims CJ. Randomized controlled trial to prevent excessive weight gain in pregnant women. International Journal of Obesity & Related Metabolic Disorders: Journal of the International Association for the Study of Obesity 2002;26(11):1494–502. 11. Poston L, Briley AL, Barr S, Bell R, Croker H, Coxon K, et al. Developing a complex intervention for diet and activity behaviour change in obese pregnant women (the UPBEAT trial); assessment of behavioural change and process evaluation in a pilot randomised controlled trial. BMC Pregnancy and Childbirth 2013;13(1):148. 12. Rauh K, Gabriel E, Kerschbaum E, Schuster T, von Kries R, Amann-Gassner U, et al. Safety and efficacy of a lifestyle intervention for pregnant women to prevent excessive maternal weight gain: a cluster-randomized controlled trial. BMC Pregnancy and Childbirth 2013;13(1):151. 13. Vinter C, Jensen D, Ovesen P, Beck-Nielsen H, Lamont R, Jorgensen J. Postpartum weight retention and breastfeeding among obese women from the LiP (Lifestyle in Pregnancy) Study. Acta Obstetricia et Gynecologica Scandinavica 2012; 91(Suppl 159):141–2. |
| Brown  *et al.* 2012[22] | 1. Polley BA, Wing RR, Sims CJ (2002) Randomized controlled trial to prevent excessive weight gain in pregnant women. Int J Obes Relat Metab Disord 26(11): 1494–1502. 2. Asbee SM, Jenkins TR, Butler JR, White J, Elliot M, et al. (2009) Preventing excessive weight gain during pregnancy through dietary and lifestyle counseling: A randomized controlled trial. Obstet Gynecol 113(2, Part 1): 305. 3. Huang T, Yeh C, Tsai Y (2009) A diet and physical activity intervention for preventing weight retention among taiwanese childbearing women: A randomised controlled trial. Midwifery 27: 257–264. 4. Phelan S, Phipps MG, Abrams B, Darroch F, Schaffner A, et al. (2011) Randomized trial of a behavioral intervention to prevent excessive gestational weight gain: The fit for delivery study. Am J Clin Nutr. 2011 Apr;93(4): 772–9. 5. Wolff S, Legarth J, Vangsgaard K, Toubro S, Astrup A (2008) A randomized trial of the effects of dietary counseling on gestational weight gain and glucose metabolism in obese pregnant women. Int J Obes 32(3): 495–501. |
| Flynn  *et al.* 2016[23] | 1. Dodd JM, Turnbull D, McPhee AJ, et al. Antenatal lifestyle advice for women who are overweight or obese: LIMIT randomised trial. BMJ. 2014;348:g1285. 2. Hawkins M, Hosker M, Marcus BH, et al. A pregnancy lifestyle intervention to prevent gestational diabetes risk factors in overweight Hispanic women: a feasibility randomized controlled trial. Diabet Med. 2015;32:108–115. 3. Harrison CL, Lombard CB, Strauss BJ, et al. Optimizing healthy gestational weight gain in women at high risk of gestational diabetes: a randomized controlled trial. Obesity (Silver Spring, Md.). 2013;21:904–909. 4. Vesco KK, Karanja N, King JC, et al. Efficacy of a group-based dietary intervention for limiting gestational weight gain among obese women: a randomized trial. Obesity (Silver Spring, Md.). 2014;22:1989–1996. 5. Petrella E, Malavolti M, Bertarini V, et al. Gestational weight gain in overweight and obese women enrolled in a healthy lifestyle and eating habits program. J Matern Fetal Neonatal Med. 2014;27:1348–1352. 6. Poston L, Briley AL, Barr S, et al. Developing a complex intervention for diet and activity behaviour change in obese pregnant women (the UPBEAT trial); assess- ment of behavioural change and process evaluation in a pilot randomised con- trolled trial. BMC Preg Childbirth. 2013;13:148. 7. Bogaerts AF, Devlieger R, Nuyts E, et al. Effects of lifestyle intervention in obese pregnant women on gestational weight gain and mental health: a randomized controlled trial. Int J Obes. (Lond.). 2013;37:814–821. 8. Guelinckx I, Devlieger R, Mullie P, et al. Effect of lifestyle intervention on dietary habits, physical activity, and gestational weight gain in obese pregnant women: a randomized controlled trial. Am J Clin Nutr. 2010;91:373–380. 9. Renault KM, Norgaard K, Nilas L, et al. The Treatment of Obese Pregnant Women (TOP) study: a randomized controlled trial of the effect of physical activity inter- vention assessed by pedometer with or without dietary intervention in obese pregnant women. Am J Obstet Gynecol. 2014;210:134.e131–e139. 10. Vinter CA, Jensen DM, Ovesen P, et al. The LiP (Lifestyle in Pregnancy) study: a randomized controlled trial of lifestyle intervention in 360 obese pregnant women. Diabetes Care. 2011;34:2502–2507. 11. Wolff S, Legarth J, Vangsgaard K, et al. A randomized trial of the effects of dietary counseling on gestational weight gain and glucose metabolism in obese pregnant women. Int J Obes. (Lond.). 2008;32:495–501. 12. Quinlivan JA, Lam LT, Fisher J. A randomised trial of a four-step multidisciplinary approach to the antenatal care of obese pregnant women. Aust N Z J Obstet Gynaecol. 2011;51:141–146. 13. Thornton YS, Smarkola C, Kopacz SM, et al. Perinatal outcomes in nutritionally monitored obese pregnant women: a randomized clinical trial. J Natl Med Assoc. 2009;101:569–577 |
| Gardner  *et al.* 2011[24] | 1. *Claesson IM, Sydsjö G, Brynhildsen J, Cedergren M, Jeppsson A, Nyström F, Sydsjö A, Josefsson A. Weight gain restriction for obese pregnant women: a case-control intervention study. Br J Obstet Gynaecol 2007; 115: 44–50. 2. *Hui AL, Ludwig SM, Gardiner P, Sevenhuysen G, Murray R, Morris M, Shen GX. Community-based exercise and dietary intervention during pregnancy: a pilot study. Can J Diabetes 2006; 30: 169–175. 3. *Polley BA, Wing RR, Cims CJ. Randomized controlled trial to prevent excessive weight gain in pregnant women. Int J Obes (Lond) 2002; 26: 1494–1502. 4. *Gray-Donald K, Robinson E, Collier A, David K, Renaud L, Rodrigues S. Intervening to reduce weight gain in pregnancy and gestational diabetes mellitus. CMAJ 2000; 163: 1247–1251. 5. *Kinnunen TI, Pasanen M, Aittasalo M, Fogelholm M, Hilakivi-Clarke L, Weiderpass E, Luoto R. Preventing excessive weight gain during pregnancy: a controlled trial in primary health care. Eur J Clin Nutr 2007; 61: 884–892. 6. *Wolff S, Legarth J, Vangsgaard K, Toubro S, Astrup A. A randomized trial of the effects of dietary counselling on gestational weight gain and glucose metabolism in obese pregnant women. Int J Obes (Lond) 2008; 32: 495–501. 7. *Asbee SM, Jenkins TR, Butler JR, White J, Elliot M, Rutledge A. Preventing excessive gestational weight during pregnancy through dietary and lifestyle counselling. Obstet Gynecol 2009; 113: 305–311. 8. *Shirazian T, Monteith S, Friedman F, Rebarber A. Lifestyle modification program decreases pregnancy weight gain in obese women. Am J Perinat 2010; 27: 411–414 9. *Guelinckx I, Devlieger R, Mullie P, Vansant G. Effect of lifestyle intervention on dietary habits, physical activity, and gestational weight gain in obese pregnant women. Am J Clin Nut 2010; 91: 373–380. 10. *Olson CM, Strawderman MS, Reed RG. Efficacy of an intervention to prevent excessive gestational weight gain. Am J Obstet Gynecol 2004; 191: 530–536.   **Note: References 3 and 9 were included in meta-analysis twice** |
| Webb-Girard *et al.* 2011[25] | 1. Abel R, Rajaratnam J, Kalaimani A, Kirubakaran S. Can iron status be improved in each of the three trimesters? A community-based study. European Journal of Clinical Nutrition 2000; 54:490–493. Nutrition education and counseling during pregnancy 201 © 2012 Blackwell Publishing Ltd Paediatric and Perinatal Epidemiology, 2012, 26 (Suppl. 1), 191–204 2. Adhikari K, Liabsuetrakul T, Pradhan N. Effect of education and pill count on hemoglobin status during prenatal care in Nepalese women: a randomized controlled trial. The Journal of Obstetrics and Gynaecology Research 2009; 35:459–466. 3. Ahrari M, Houser RF, Yassin S, Mogheez M, Hussaini Y. A positive deviance-based antenatal nutrition project improves birth-weight in upper Egypt. Journal of Health, Population, and Nutrition 2006; 24:498–507. 23 4. Anderson AS, Campbell DM, Shepherd R. The influence of dietary advice on nutrient intake during pregnancy. The British Journal of Nutrition 1995; 73:163–177. 5. Asbee SM, Jenkins TR, Butler JR, White J, Elliot M, Rutledge A. Preventing excessive weight gain during pregnancy through dietary and lifestyle counseling: a randomized controlled trial. Obstetrics and Gynecology 2009; 113:305–312. 6. Bechtel-Blackwell DA. Computer-assisted self-interview and nutrition education in pregnant teens. Clinical Nursing Research 2002; 11:450–462. 7. Belizan JM, Barros F, Langer A, Farnot U, Victora C, Villar J. Impact of health education during pregnancy on behavior and utilization of health resources. Latin American Network for Perinatal and Reproductive Research. American Journal of Obstetrics and Gynecology 1995; 173:894–899. 8. Berry K, Wiehl DG. An experiment in diet education during pregnancy. The Milbank Memorial Fund Quarterly 1952; 30:119–151. 9. Briley C, Flanagan NL, Lewis N. In-home prenatal nutrition intervention increased dietary iron intakes and reduced low birthweight in low-income African-American women. Journal of the American Dietetic Association 2002; 102:984–987. 10. Daelhousen BB, Guthrie HA. A self-instruction nutrition program for pregnant women. Journal of the American Dietetic Association 1982; 81:407–412. 11. el-Shabrawy Ali M. Effects of nutrition education on the dietary patterns of two socio-economic groups of pregnant females in Dakahlia Governorate (Egypt). The Journal of the Egyptian Medical Association 1971; 54:829–840. 12. Ershoff DH, Aaronson NK, Danaher BG, Wasserman FW. Behavioral, health, and cost outcomes of an HMO-based prenatal health education program. Public Health Reports 1983; 98:536–547 13. Gadallah M, Rady M, Salem B, Aly EM, Anwer W. The effect of nutritional intervention program on the prevalence of anemia among pregnant women in rural areas of Belbis district-Sharkia Governorate-Egypt. The Journal of the Egyptian Public Health Association 2002; 77:261–273. 14. Garg A, Kashyap S. Effect of counseling on nutritional status during pregnancy. Indian Journal of Pediatrics 2006; 73:687–692. 15. Graham AV, Frank SH, Zyzanski SJ, Kitson GC, Reeb KG. A clinical trial to reduce the rate of low birth weight in an inner-city black population. Family Medicine 1992; 24:439–446. 16. Hankin ME, Symonds EM. Body weight, diet and pre-eclamptic toxaemia of pregnancy. The Australian & New Zealand Journal of Obstetrics & Gynaecology 1962; 4:156–160. 17. Hunt IF, Jacob M, Ostegard NJ, Masri G, Clark VA, Coulson AH. Effect of nutrition education on the nutritional status of low-income pregnant women of Mexican descent. The American Journal of Clinical Nutrition 1976; 29:675–684 18. Hunt DJ, Stoecker BJ, Hermann JR, Kopel BL, Williams GS, Claypool PL. Effects of nutrition education programs on anthropometric measurements and pregnancy outcomes of adolescents. Journal of the American Dietetic Association 2002; 102:S100–S102. 19. Kafatos AG, Vlachonikolis IG, Codrington CA. Nutrition during pregnancy: the effects of an educational intervention program in Greece. The American Journal of Clinical Nutrition 1989; 50:970–979. 20. Kinnunen TI, Pasanen M, Aittasalo M, Fogelholm M, Hilakivi-Clarke L, Weiderpass E, et al. Preventing excessive weight gain during pregnancy – a controlled trial in primary health care. European Journal of Clinical Nutrition 2007; 61:884–891. 21. Laitinen K, Poussa T, Isolauri E. Probiotics and dietary counselling contribute to glucose regulation during and after pregnancy: a randomised controlled trial. The British Journal of Nutrition 2009; 101:1679–1687. 22. Long VA, Martin T, Janson-Sand C. The great beginnings program: impact of a nutrition curriculum on nutrition knowledge, diet quality, and birth outcomes in pregnant and parenting teens. Journal of the American Dietetic Association 2002; 102:S86–S89. 23. McLaughlin FJ, Altemeier WA, Christensen MJ, Sherrod KB, Dietrich MS, Stern DT. Randomized trial of comprehensive prenatal-care for low-income women – effect on infant birth-weight. Pediatrics 1992; 89:128–132. 24. Ndiaye M, Siekmans K, Haddad S, Receveur O. Impact of a positive deviance approach to improve the effectiveness of an iron-supplementation program to control nutritional anemia among rural Senegalese pregnant women. Food and Nutrition Bulletin 2009; 30:128–136. 25. Olds DL, Henderson CR, Tatelbaum R, Chamberlin R. Improving the delivery of prenatal-care and outcomes of pregnancy – a randomized trial of nurse home visitation. Pediatrics 1986; 77:16–28. 26. Piirainen T, Isolauri E, Lagstrom H, Laitinen K. Impact of dietary counselling on nutrient intake during pregnancy: a prospective cohort study. The British Journal of Nutrition 2006; 96:1095–1104. 27. Polley B, Wing R, Sims C. Randomized controlled trial to prevent excessive weight gain in pregnant women. International Journal of Obesity 2002; 26:1494–1502. 28. Sachdeva R, Mann SK. Impact of nutrition education and medical supervision on pregnancy outcome. Indian Pediatrics 1993; 30:1309–1314. 29. Sachdeva R, Mann SK. Impact of nutrition counselling and supplements on the mineral nutriture of rural pregnant women and their neonates. Indian Pediatrics 1994; 31:643–649. 30. Senanayake HM, Premaratne SP, Palihawadana T, Wijeratne S. Simple educational intervention will improve the efficacy of routine antenatal iron supplementation. The Journal of Obstetrics and Gynaecology Research 2010; 36:646–650. 31. Smoke J, Grace MC. Effectiveness of prenatal care and education for pregnant adolescents: nurse-midwifery intervention and team approach. Journal of Nurse-Midwifery 1988; 33:178–184 32. Sultemeier A. An innovative approach to teaching prenatal nutrition. Journal of Community Health Nursing 1988; 5:247–254. 33. Sun JD, Shao YF, Zhang PL, Li DZ, Gu LY, Guo QN. Evaluation of prenatal nutrition counseling: maternal nutrition status and infant birthweight. Biomedical and Environmental Sciences 1990; 3:458–465. 32 34. Widga AC, Lewis NM. Defined, in-home, prenatal nutrition intervention for low-income women. Journal of the American Dietetic Association 1999; 99:1058–1062; quiz 1063–1054, 1175. |
| Mohd Yusof  *et al.* 2014[26] | 1. Moses RG et al (2009) Can a low glycaemic index diet reduce the need for insulin in gestational diabetes? Diabetes Care, 32, 996. 2. Grant SM et al (2011) Effect of a low glycaemic diet on blood glucose in women with gestational hyperglycaemia. Diabetes Res Clin Prac, 91, 15-22. 3. Louie JCY et al (2011) A randomized controlled trial investigating the effects of a low glycaemic index diet on pregnancy outcomes in gestational diabetes mellitus. Diabetes Care, 34, 2341-6. |
| Muktabhant  *et al.* 2015[27] | 1. Althuizen E, Van Poppel MN, Seidell JC, Van der Wijden C, Van Mechelen W. Design of the new life(style) study: a randomised controlled trial to optimise maternal weight development during pregnancy. *BMC Public Health* 2006;6:168. 2. Althuizen E, van der Wijden C, van Mechelen W, SeidellJ, van Poppel M. The effect of a counselling intervention on weight changes during and after pregnancy: a randomised trial. *BJOG : an International Journal of Obstetrics and Gynaecology*2013;120(1):92–9. 3. Broekhuizen K, Althuizen E, van Poppel MNM, Donker M, van Mechelen W. From theory to practice: intervention fidelity in a randomized controlled trial aiming to optimize weight development during pregnancy. *Health Promotion Practice* 2012;13(6):816–25. 4. Angel MD, De Haene J, Perez M, Hernandez G, Castaneda D, King JC. Dietary patterns associated with gestational weight gain and fat mass gain in overweight and obese pregnant women. *FASEB Journal* 2011;25:783.15. 5. Asbee SM, Jenkins TR, Butler JR, White J, Elliot M, Rutledge A. Dietary counseling prevents excessive weight gain during pregnancy, a randomized controlled trial. *Obstetrics & Gynecology* 2008;11(4 Suppl):6S. 6. Asbee SM, Jenkins TR, ButlerJR, White J, Elliot M, Rutledge A. Preventing excessive weight gain during pregnancy through dietary and lifestyle counseling: a randomized controlled trial. *Obstetrics & Gynecology* 2009;113 (2 Pt 1):305–12. 7. Barakat R. Effect of physical exercise program during pregnancy on excessive weight gain and its consequences. [ClinicalTrials.gov](https://www.dropbox.com/referrer_cleansing_redirect?hmac=5WLySbT4Hb7szRpbJ9U75Y6Z7NFaQ7F3XH0jwK6iJbM%3D&url=http%3A%2F%2FClinicalTrials.gov)[(http://clinicaltrials.gov/)](https://www.dropbox.com/referrer_cleansing_redirect?hmac=An6ki23nhchhSzR2Oqm6oQ%2FUvkNbI3ult6gA2vOtHTM%3D&url=http%3A%2F%2F(http%3A%2F%2Fclinicaltrials.gov%2F)) [accessed 21 May 2013] 2011. 8. Barakat R, Pelaez M, Montejo R, Luaces M, Zakynthinaki M. Exercise during pregnancy improves maternal health perception: a randomized controlled trial. *American Journal of Obstetrics and Gynecology* 2011;204(5):402.e1–402.e7. 9. Bisson M, Almeras N, Dufresne S, Rheaume C, Bujold E, Robitailee J, et al. Exercise improving fitness in obese women during pregnancy: a difference for the mother and child?. Pediatric Academic Societies and Asian Society for Pediatric Research Joint Meeting; 2014 May 3-6;Vancouver, Canada. 2014:Abstract no: 2946.618. 10. Bogaerts A. Effect of psycho-education on gestational weight gain and anxiety/depression in obese pregnant women. [ClinicalTrials.gov](https://www.dropbox.com/referrer_cleansing_redirect?hmac=5WLySbT4Hb7szRpbJ9U75Y6Z7NFaQ7F3XH0jwK6iJbM%3D&url=http%3A%2F%2FClinicalTrials.gov)[(http://clinicaltrials.gov/)](https://www.dropbox.com/referrer_cleansing_redirect?hmac=An6ki23nhchhSzR2Oqm6oQ%2FUvkNbI3ult6gA2vOtHTM%3D&url=http%3A%2F%2F(http%3A%2F%2Fclinicaltrials.gov%2F)) [accessed 21 May 2013] 2011. 11. Bogaerts A, Devlieger R, Nuyts E, Witters I, Gyselaers W, Guelinckx I. Psycho-education reduces gestational weight gain in obese pregnant women: Randomized controlled trial [abstract]. *Obesity Facts* 2012;5:53. 12. Byrne NM, Groves AM, McIntyre HD, Callaway LK. Changes in resting and walking energy expenditure and walking speed during pregnancy in obese women. *American Journal of Clinical Nutrition* 2011;94(3):819–30. 13. Callaway L. A randomized controlled trial using exercise to reduce gestational diabetes and other adverse maternal and neonatal outcomes in obese pregnant women - the pilot study. Australian Clinical Trials Registry [(www.actr.org.au](https://www.dropbox.com/referrer_cleansing_redirect?hmac=%2FqNONmwvRt3gD2xb%2FfUTsY06vStnRty%2B7t0qN24qbVU%3D&url=http%3A%2F%2F(www.actr.org.au)/)[accessed 31 October 2010]. 14. Callaway L, McIntyre D, Colditz P, Byrne N, Foxcroft K, O’Connor B. Exercise in obese pregnant women: a randomized study to assess feasibility. *Hypertension in Pregnancy* 2008;27(4):549. 15. Callaway LK, Colditz PB, Byrne NM, Lingwood BE, Rowlands IJ, Foxcroft K, et al. Prevention of gestational diabetes. Feasibility issues for an exercise intervention in obese pregnant women. *Diabetes Care* 2010;33(7):1457–9. 16. Foxcroft KF, Rowlands IJ, Byrne NM, McIntyre HD, Callaway LK, for the BAMBINO group. Exercise in obese pregnant women: the role of social factors, lifestyle and pregnancy symptoms. *BMC Pregnancy and Childbirth* 2011;11:4. 17. Clapp III JF. Maternal carbohydrate intake and pregnancy outcome. *Proceedings of the Nutrition Society* 2002; 61(1):45–50. 18. Clapp IJF. Diet, exercise, and feto-placental growth. *Archives of Gynecology and Obstetrics* 1997;260:101–8. 19. Clapp JF 3rd, Kim H, Burciu B, Schmidt S, Petry K, Lopez B. Continuing regular exercise during pregnancy: effect of exercise volume on fetoplacental growth. *American Journal of Obstetrics and Gynecology* 2002;186(1):142–7. 20. Cordero Y, Mottola MF, Vargas J, Blanco M, Barakat R. Exercise is associated with a reduction in gestational diabetes mellitus. Medicine and Science in Sports and Exercise 2014;Oct 20 [Epub ahead of print]. 21. De Oliveria Melo AS, Silva JLP, Tavares JS, Barros VO, Leite DFB, Amorim MMR. Effect of a physical exercise program during pregnancy on uteroplacental and fetal blood flow and fetal growth: A randomized controlled trial. *Obstetrics & Gynecology* 2012;120(2 Pt 1):302–10. 22. Melo A. Exercise and pregnancy: randomised clinical trial. Current Controlled Trials [(www.controlled-trials.com/)](https://www.dropbox.com/referrer_cleansing_redirect?hmac=YPGjIV707QXeIYSj6BVOrrnFGx%2Fopsn7NqhYIe5SKkk%3D&url=http%3A%2F%2F(www.controlled-trials.com%2F))[accessed 31 October 2010]. 23. Di Carlo C, Iannotti G, Sparice S, Chiacchio MP, Greco E, Tommaselli GA, et al. The role of a personalized dietary intervention in managing gestational weight gain: a prospective, controlled study in a low-risk antenatal population. *Archives of Gynecology and Obstetrics* 2014;289:765–70. 24. Cramp CS, Moran LJ, Deussen AR, Yelland LN, Dodd JM. Evaluation of printed nutrition education material in overweight and obese women during pregnancy-findings from the limit randomised trial. *Journal of Paediatrics and Child Health* 2013;49 Suppl 2:118. 25. Dodd J. Limiting weight gain in overweight and obese women during pregnancy to improve health outcomes: a randomised trial. Australian Clinical Trials Registry [(www.actr.org.au/)](https://www.dropbox.com/referrer_cleansing_redirect?hmac=miAR0ld8inTp7LjP8pLhJ7pc2fHa%2BXGY1ECDo96DT3M%3D&url=http%3A%2F%2F(www.actr.org.au%2F)) (accessed 31 October 2010). 26. Dodd J. Obesity in pregnancy-the limit randomised trial. *Journal of Paediatrics and Child Health* 2013;49 Suppl 2:4. 27. Dodd JM. Dietary and lifestyle advice for pregnant women who Are overweight or obese: the LIMIT randomized trial. *Annals of Nutrition & Metabolism* 2014;64(3-4):197–202. 28. Dodd JM, Cramp C, Sui Z, Yelland LN, Deussen AR, Grivell RM, et al. The effects of antenatal dietary and lifestyle advice for women who are overweight or obese onmaternal diet and physical activity: the LIMIT randomised trial. *BMC Medicine* 2014;12(1):161. 29. Dodd JM, McPhee AJ, Turnbull D, Yelland LN, Deussen 30. AR, Grivell RM, et al. The effects of antenatal dietary and lifestyle advice for women who are overweight or obese on neonatal health outcomes: the LIMIT randomised trial. *BMC Medicine* 2014;12(1):163. 31. Dodd JM, Turnbull D, McPhee AJ, Deussen AR, Grivell RM, Yelland LN, et al. Antenatal lifestyle advice for women who are overweight or obese: LIMIT randomised trial.*BMJ (Clinical Research Ed.)*2014;348:g1285. 32. Dodd JM, Turnbull DA, McPhee AJ, Wittert G, Crowther CA, Robinson JS. Limiting weight gain in overweight and obese women during pregnancy to improve health outcomes: the LIMIT randomised controlled trial. *BMC Pregnancy and Childbirth* 2011;11:79. 33. Eames AJ, Grivell RM, Dodd JM, Deussen A. The effect of limited gestational weight gain in overweight and obese women on maternal and infant outcomes. *Journal of Paediatrics and Child Health* 2013;49 Suppl 2:19. 34. Grivell R, Yelland L, Earl RA, Staehr CJ, Dodd J. The effect of antenatal dietary and lifestyle advice on fetal body composition in women who are overweight or obese: findings from the LIMIT randomised trial. *Ultrasound in Obstetrics and Gynecology* 2013;42(Suppl 1):10. 35. Grivell R, Yelland L, Staehr CJ, Earl RA, Dodd J. The effect of antenatal dietary and lifestyle advice on fetal growth in women who are overweight or obese: findings from the LIMIT randomised trial. *Ultrasound in Obstetrics and Gynecology* 2013;42(Suppl 1):83. 36. Kannieappan LM, Deussen AR, Moran LJ, Grivell RM, Yelland LN, Dodd JM. The effect of antenatal dietary advice on maternal body composition in women who are overweight or obese - findings from the limit randomised trial. *Journal of Paediatrics and Child Health* 37. 2013;49 Suppl2:94. 38. Newman AK, Deussen AR, Moran LJ, Grivell RM, Yelland LN, Turnbull D, et al. The effect of antenatal dietary and lifestyle advice on maternal psychological health in women who are overweight or obese-findings from the limit randomised trial. *Journal of Paediatrics and Child Health* 2013;49 Suppl 2:119. 39. Sui Z, Yelland LN, Turnbull D, Dodd JM. Walking to limit gestational weight gain and keep fit during pregnancy - findings from the walk randomised trial. *Journal of Paediatrics and Child Health* 2013;49 Suppl 2:120. 40. Ehrlich SF, Hedderson MM, Feng J, Crites Y, Quesenberry CP, Ferrara A. Lifestyle intervention improves postpartum fasting glucose levels in women with gestational diabetes. *Diabetes* 2014; 63 (Suppl 1):A95. 41. Ferrara A. Diet, exercise and breastfeeding intervention program for women with gestational diabetes (DEBI Trial). [ClinicalTrials.gov](https://www.dropbox.com/referrer_cleansing_redirect?hmac=5WLySbT4Hb7szRpbJ9U75Y6Z7NFaQ7F3XH0jwK6iJbM%3D&url=http%3A%2F%2FClinicalTrials.gov)[(www.clinicaltrials.gov)](https://www.dropbox.com/referrer_cleansing_redirect?hmac=LJcCh%2BVT3tZ%2BaqSHfqJfboFykUDsduloFI4FX0k9Jcc%3D&url=http%3A%2F%2F(www.clinicaltrials.gov)) [accessed 31 October 2010]. 42. Ferrara A, Hedderson MM, Albright CL, Ehrlich SF, Quesenberry CP, Peng T, et al. A pregnancy and postpartum lifestyle intervention in women with gestational diabetes mellitus reduces diabetes risk factors: a feasibility randomized control trial. *Diabetes Care* 2011;34(7):1519–25. 43. Guelinckx I, Devlieger R, Mullie P, Vansant G. Effect of lifestyle intervention on dietary habits, physical activity, and gestational weight gain in obese pregnant women: a randomized controlled trial. *American Journal of Clinical Nutrition* 2010;91(2):373–80. 44. Bo K, Haakstad LAH. Is pelvic floor muscle training effective when taught in a general fitness class in pregnancy? A randomised controlled trial. *Physiotherapy* 2011;97(3):190–5. 45. Haakstad L. Effect of regular exercise in prevention of excessive weight gain in pregnancy. [ClinicalTrials.gov](https://www.dropbox.com/referrer_cleansing_redirect?hmac=5WLySbT4Hb7szRpbJ9U75Y6Z7NFaQ7F3XH0jwK6iJbM%3D&url=http%3A%2F%2FClinicalTrials.gov) [(www.clinicaltrials.gov)](https://www.dropbox.com/referrer_cleansing_redirect?hmac=LJcCh%2BVT3tZ%2BaqSHfqJfboFykUDsduloFI4FX0k9Jcc%3D&url=http%3A%2F%2F(www.clinicaltrials.gov)) [accessed 31 October 2010]. 46. Haakstad L, Bo K. Effect of supervised aerobic dance exercise in prevention of excessive weight gain in pregnancy: a single blind randomized controlled trial. *International Journal of Gynecology & Obstetrics* 2009;107(Suppl 2):S198. 47. Haakstad L, Bo K. Exercise during pregnancy-Does it impact offspring birth weight parameters?. *Journal of Science and Medicine in Sport* 2012;15(Suppl 1):S340. 48. Haakstad LA, Bo K. Exercise in pregnant women and birth weight: a randomized controlled trial. *BMC Pregnancy and Childbirth* 49. 2011;11:66. 50. Haakstad LAH, Bo K. Effect of regular exercise on prevention of excessive weight gain in pregnancy: A randomised controlled trial. 51. *European Journal of Contraception and Reproductive Health Care* 2011;16(2):116–25. 52. Harrison CL, Lombard CB, Gibson-Helm M, Deeks A, Teede HJ. Limiting excess weight gain in high-risk pregnancies: A randomized controlled trial. *Endocrine Reviews* 2011; 32(3 Meeting Abstracts):P1–466. 53. Harrison CL, Lombard CB, Strauss BJ, Teede HJ. Optimizing healthy gestational weight gain in women at high risk of gestational diabetes: a randomized controlled trial. *Obesity* 2013;21(5):904–9. 54. Harrison CL, Teede HJ, Lombard CB. How effective is self-weighing in the setting of a lifestyle intervention to reduce gestational weight gain and postpartum weight retention?. *Australian & New Zealand Journal of Obstetrics & Gynaecology* 2014;54:382–5. 55. Lombard C, Harrison C, Teede H. A randomized controlled trial investigating self-weighing and the prevention of excess weight gain in early pregnancy. *Endocrine Reviews* 2011;32(3 Meeting Abstracts):P2–768. 56. Teede HJ, Harrison CL, Gibson-Helm M, Lombard CB. Improving physical activity in high-risk pregnancies: A randomized controlled trial. *Endocrine Reviews* 2011;32(3Meeting Abstracts):P1–467. 57. Hawkins M, Hosker M, Marcus BH, Rosal MC, Braun B, Stanek EJ, et al. A pregnancy lifestyle intervention to prevent gestational diabetes risk factors in overweight Hispanic women: a feasibility randomized controlled trial. *Diabetic Medicine* 2014;32:108–15. 58. Huang TT, Yeh CY, Tsai YC. A diet and physical activity intervention for preventing weight retention among Taiwanese childbearing women: a randomised controlled trial. *Midwifery* 2011;27(2):257–64. 59. Hui AL, Ludwig SM, Gardiner P, Sevenhuysen G, Murray R, Morris M, et al. Community-based exercise and dietary intervention during pregnancy: a pilot study. *Canadian Journal of Diabetes* 2006; 30(2):169–75. 60. Hui A, Back L, Ludwig S, Gardiner P, Sevenhuysen G, Dean H, et al. Lifestyle intervention on diet and exercise reduced excessive gestational weight gain in pregnant women undera randomised controlled trial. *BJOG: an International Journal of Obstetrics & Gynaecology* 2012;119(1):70–7. 61. Hui AL, Ludwig S, Gardiner P, Sevenhuysen G, Dean HJ, Sellers E, et al. Effects of lifestyle intervention on dietary intake, physical activity level, and gestational weight gain in pregnant women with different pre-pregnancy Body Mass Index in a randomized control trial. *BMC Pregnancy and Childbirth* 2014;14(1):331. 62. Jackson RA, Stotland NE, Caughey AB, Gerbert B. Improving diet and exercise in pregnancy with Video Doctor counseling: a randomized trial. *Patient Education and Counseling* 2011;83(2):203–9. 63. Jeffries K, Shub A, Walker SP, Hiscock R, Permezel M. Reducing excessive weight gain in pregnancy: a randomised controlled trial. 64. *Medical Journal of Australia* 2009;191(8):429–33. 65. Shub A. Diet and exercise in pregnancy. Australian Clinical Trials Registry [(www.actr.org.au/)](https://www.dropbox.com/referrer_cleansing_redirect?hmac=miAR0ld8inTp7LjP8pLhJ7pc2fHa%2BXGY1ECDo96DT3M%3D&url=http%3A%2F%2F(www.actr.org.au%2F)) [accessed 21 June 2007]. 66. Kieffer EC, Welmerink DB, Sinco BR, Welch KB, Rees Clayton EM, Schumann CY, et al. Dietary outcomes in a Spanish-language randomized controlled diabetes prevention trial with pregnant Latinas. *American Journal of Public Health* 2014;104(3):526–33. 67. Kong KL, Campbell G, Foster C, Peterson D, Lanningham-Foster L. A pilot walking program promotes moderate-intensity physical activity during pregnancy. *Medicine & Science in Sports & Exercise* 2014;46(3):462–72. 68. Korpi-Hyovalti E, Schwab U, Laaksonen DE, Linjama H, Heinonen S, Niskanen L. Effect of intensive counselling on and increasing physical activity during pregnancy: A feasibility study. *Diabetes* 2012;61 Suppl 1:A344. 69. Umpierrez G. Lifestyle intervention to limit excessive weight gain during pregnancy in minority women. [ClinicalTrials.gov](https://www.dropbox.com/referrer_cleansing_redirect?hmac=5WLySbT4Hb7szRpbJ9U75Y6Z7NFaQ7F3XH0jwK6iJbM%3D&url=http%3A%2F%2FClinicalTrials.gov) (accessed 21 May 2013). NCT01084941 2010. 70. Moses RG, Barker M, Winter M, Petocz P, Brand-Miller JC. Can a low-glycemic index diet reduce the need for insulin in gestational diabetes mellitus? A randomized trial. *Diabetes Care* 2009;32(6):996–1000. 71. Brand-Miller J. A pregnancy intervention to reduce postprandial glucose excursions in the primary prevention of paediatric obesity. Current Controlled Trials [(www.controlled-trials.com/)](https://www.dropbox.com/referrer_cleansing_redirect?hmac=YPGjIV707QXeIYSj6BVOrrnFGx%2Fopsn7NqhYIe5SKkk%3D&url=http%3A%2F%2F(www.controlled-trials.com%2F)) [accessed 31 October 2010]. 72. Moses RG, Casey S, Cleary J, Milosavljevic M, Quinn E, Tapsell L, et al. Effect of low glycaemic index dietary advice in normal pregnancy: The PREGGIO study. *Obesity Research and Clinical Practice* 2013;7:e34–5. 73. Moses RG, Casey SA, Quinn EG, Cleary JM, Tapsell LC, Milosavljevic M, et al. Pregnancy and Glycemic Index Outcomes study: effects of low glycemic index compared with conventional dietary advice on selected pregnancy outcomes. *American Journal of Clinical Nutrition* 2014;99(3):517–23. 74. Mujsindi W, Habash D, Childs G. Impact of nutrition education on gestational weight gain in obese pregnant women. *American Journal of Obstetrics and Gynecology* 2014;210(1 Suppl):S188. 75. Murtezani A, Pacarada M, Ibraimi Z, Nevzati A, Abazi N. The impact of exercise during pregnancy on neonatal outcomes: a randomised controlled trial. *Journal of Sports Medicine and Physical Fitness* 2014;54(6):802–8. 76. Nascimento KLK, Surita SLN, Parpinelli FGS, Kasawara MAP. Type of delivery and neonatal outcome in overweight and obese pregnant women with excessive weight gain. *Journal of Maternal-Fetal and Neonatal Medicine* 2012;25(S2):73–4. 77. Nascimento SL, Surita FG, Parpinelli MA, Siani S, Pintoe Silva JL. The effect of an antenatal physical exercise programme on maternal/perinatal outcomes and quality of life in overweight and obese pregnant women: a randomised clinical trial. *BJOG: an International Journal of Obstetrics & Gynaecology* 2011;118(12):1455–63. 78. Surita F. Physical exercise influence among overweightand obese pregnant women. [ClinicalTrials.gov](https://www.dropbox.com/referrer_cleansing_redirect?hmac=5WLySbT4Hb7szRpbJ9U75Y6Z7NFaQ7F3XH0jwK6iJbM%3D&url=http%3A%2F%2FClinicalTrials.gov) (http://[clinicaltrials.gov/)](https://www.dropbox.com/referrer_cleansing_redirect?hmac=10ucmo0GUjXKZslchdxEyyW85phL%2FEV6I2qBkesMoEo%3D&url=http%3A%2F%2Fclinicaltrials.gov%2F)) [accessed 21 May 2013] 2010. 79. Oostdam N, Bosmans J, Wouters MGAJ, Eekhoff EMW, van Mechelen W, van Poppel MNM. Cost-effectiveness of an exercise program during pregnancy to prevent gestational diabetes: Results of an economic evaluation alongside a randomised controlled trial. *BMC Pregnancy and Childbirth* 2012;12:64. 80. Oostdam N, Van Poppel MN, Eekhoff EM, Wouters MG, Van Mechelen W. Design of FitFor2 study: the effects of an exercise program on insulin sensitivity and plasma glucose levels in pregnant women at high risk for gestational diabetes. *BMC Pregnancy and Childbirth* 2009;9:1. 81. Oostdam N, Van Poppel MNM, Wouters MGAJ, Eekhoff EMW, Bekedam DJ, Kuchenbecker WKH, et al. No effect of the FitFor2 exercise programme on blood glucose, insulin sensitivity, and birthweight in pregnant women who were overweight and at risk for gestational diabetes: Results of a randomised controlled trial. *BJOG: an International Journal of Obstetrics and Gynaecology* 2012;119(9):1098–107. 82. Van Poppel M, Oostdam N, Wouters M, Eekhoff M, Van Mechelen W. FitFor2: Effects of an exercise training program on the incidence of gestational diabetes. *Journal of Science and Medicine in Sport* 2012;15 Suppl 1:S342–S343. 83. Facchinetti F. Pregnancy complications in women with BMI > 25kg/m2 enrolled in a healthy lifestule and eating habits program. [ClinicalTrials.gov](https://www.dropbox.com/referrer_cleansing_redirect?hmac=5WLySbT4Hb7szRpbJ9U75Y6Z7NFaQ7F3XH0jwK6iJbM%3D&url=http%3A%2F%2FClinicalTrials.gov) (accessed 21 May 2013). NCT01783210 2013. 84. Petrella E, Facchinetti F, Bertarini V, Pignatti L, Neri I, Battistini NC. Occurrence of pregnancy complications in women with BMI > 25 submitted to a healthy lifestyle and eating habits program. *American Journal of Obstetrics and Gynecology* 2013;208(1 Suppl):S33–4. 85. Petrella E, Malavolti M, Bertarini V, Pignatti L, Neri I, Battistini NC, et al. Gestational weight gain in overweight and obese women enrolled in a healthy lifestyle and eating habits program. *Journal of Maternal-Fetal and Neonatal Medicine* 2014;27(13):1348–52. 86. Petrov Fieril K, Glantz A, Fagevik Olsen M. The efficacy of moderate-to-vigorous resistance exercise during pregnancy: A randomized controlled trial. Acta Obstetricia et Gynecologica Scandinavica 2014 Oct 7 [Epub ahead of print]. 87. Phelan S, Phipps MG, Abrams B, Darroch F, Grantham K, Schaffner A, et al. Does behavioral intervention in pregnancy reduce postpartum weight retention? Twelve-month outcomes of the Fit for Delivery randomized trial. *American Journal of Clinical Nutrition* 2014;99(2):302–11. 88. Phelan S, Phipps MG, Abrams B, Darroch F, Schaffner A, Wing RR. Factors associated with success in the “fit for delivery” intervention to reduce excessive gestational weight gain. *Obesity* 2011;19(Suppl 1):S95. 89. Phelan S, Phipps MG, Abrams B, Darroch F, Schaffner A, Wing RR. Randomized trial of a behavioral intervention to prevent excessive gestational weight gain: the Fit for Delivery Study. *American Journal of Clinical Nutrition* 2011;93(4):772–9. 90. Pinzon DC, Zamora K, Martinez JH, Florez-Lopez ME, de Plata AC, Mosquera M, et al. Type of delivery and 91. Ramirez-Velez R. Aerobic exercise in pregnant Latina women: Effect on metabolic and body composition outcomes. A randomized clinical trial. *FASEB Journal* 2014;28(1 Suppl 1):886.6. 92. Ramirez-Velez R. Combined aerobic and resistance exercise on metabolic and body composition outcomes in primigravid Latina women. *Obesity Reviews* 2014;15(Suppl2):201–2. 93. Ramirez-Velez R, Aguilar de Plata AC, Escudero MM, Echeverry I, Ortega JG, Salazar B, et al. Influence of regular aerobic exercise on endothelium-dependent vasodilation and cardiorespiratory fitness in pregnant women. *Journal of Obstetrics and Gynaecology Research* 2011;37(11):1601–8. 94. Pollak KI, Alexander SC, Bennett G, Lyna P, Coffman CJ, Bilheimer A, et al. Weight-related SMS texts promoting appropriate pregnancy weight gain: A pilot study. *Patient Education and Counselling* 2014;97:256–60. 95. Polley BA, Wing RR, Sims CJ. Randomized controlled trial to prevent excessive weight gain in pregnant women. *International Journal of Obesity & Related Metabolic Disorders: Journal of the International Association for the Study of Obesity* 2002;26(11):1494–502. 96. Briley AL, Barr S, Badger S, Bell R, Croker H, Godfrey KM, et al. A complex intervention to improve pregnancy outcome in obese women; the UPBEAT randomised controlled trial. *BMC Pregnancy and Childbirth* 2014;14(1):74. 97. Hayes L, Bell R, Robson S, Poston L. Association between physical activity in obese pregnant women and pregnancy outcomes: The UPBEAT Pilot Study. *Annals of Nutrition & Metabolism* 2014;64(3-4):239–46. 98. Maitland RA, Barr S, Briley A, Seed P, Poston L. Incidence of gestational diabetes in an obese population using the International Association of Diabetes and Pregnancy Study Groups (IADPSG) criteria in the UK Pregnancies Better Eating and Activity Trial (UPBEAT) pilot study. *Diabetic Medicine* 2012;29(Suppl 1):152. 99. Poston L. Improving pregnancy outcome in obese women: a feasibility study. Current Controlled Trials (http://[controlled-trials.com/)](https://www.dropbox.com/referrer_cleansing_redirect?hmac=rHNe51lhWn%2F0B9DgogBPbs6dQ545SGQx1e6PiCym0mM%3D&url=http%3A%2F%2Fcontrolled-trials.com%2F)) (accessed 31 October 2010). 100. Poston L, Briley AL, Barr S, Bell R, Croker H, Coxon K, et al. Developing a complex intervention for diet andactivity behaviour change in obese pregnant women (the UPBEAT trial); assessment of behavioural change and process evaluation in a pilot randomised controlled trial. *BMC Pregnancy and Childbirth* 2013;13(1):148. 101. Schneeberger C, Flynn A, Barr S, Seed PT, Inskip HM, Poston L. Maternal diet patterns and glycaemic load in obese pregnant women taking part in a pilot trial of a lifestyle intervention (the upbeat trial). Diabetes 2014; Vol.63. 102. Price B, Amini B, Kappeler K. Exercise in pregnancy: Effect on fitness and obstetric outcomes - a randomized trial. *Medicine & Science in Sports & Exercise* 2012;44(12):2263–9. 103. Quinlivan JA, Lam LT, Fisher J. A randomised trial of a four-step multidisciplinary approach to the antenatal care of obese pregnant women. *Australian and New Zealand Journal of Obstetrics and Gynaecology* 2011;51:141–6. 104. Rae A, Bond D, Evans S, North F, Roberman B, Walters B. A randomised controlled trial of dietary energy restriction in the management of obese women with gestational diabetes. *Australian & New Zealand Journal of Obstetrics & Gynaecology* 2000;40 (4):416–22. 105. Rauh K, Gabriel E, Kerschbaum E, Schuster T, von Kries R, Amann-Gassner U, et al. Safety and efficacy of a lifestyle intervention for pregnant women to prevent excessive maternal weight gain: a cluster-randomized controlled trial. *BMC Pregnancy and Childbirth* 2013;13(1):151. 106. Renault KM, Norgaard K, Nilas L, Carlsen EM, Cortes D, Pryds O, et al. The Treatment of Obese Pregnant Women (TOP) study: A randomized controlled trial of the effect of physical activity intervention assessed by pedometer with or without dietary intervention in obese pregnant women. *American Journal of Obstetrics and Gynecology* 2014;210(2):134.e1–9. 107. Pawlak DB. Glycemic load and infant birth weight in pregnant overweight/obese women. [ClinicalTrials.gov](https://www.dropbox.com/referrer_cleansing_redirect?hmac=5WLySbT4Hb7szRpbJ9U75Y6Z7NFaQ7F3XH0jwK6iJbM%3D&url=http%3A%2F%2FClinicalTrials.gov) [(http://clinicaltrials.gov/)](https://www.dropbox.com/referrer_cleansing_redirect?hmac=An6ki23nhchhSzR2Oqm6oQ%2FUvkNbI3ult6gA2vOtHTM%3D&url=http%3A%2F%2F(http%3A%2F%2Fclinicaltrials.gov%2F)) (accessed 31 October 2010). 108. Rhodes ET, Pawlak DB, Takoudes TC, Ebbeling CB, Feldman HA, Lovesky MM, et al. Effects of a low-glycemic load diet in overweight and obese pregnant women: a pilot randomized controlled trial. *American Journal of Clinical Nutrition* 2010;92(6):1306–15. 109. Donnelly J, Horan M, Walsh J, McGowan C, Byrne J, Molloy EJ, et al. Impact of a low GI diet on neonatal body composition (ROLO Kids). Pediatric Academic Societies Annual Meeting; 2013 May 4-7; Washington DC, USA. 2013. 110. Donnelly JM, Walsh JM, Byrne J, Molloy E, McAuliffe FM. Altered neonatal anthropometric measurements following maternal low GI diet in pregnancy (ROLO study). *Acta Obstetricia et Gynecologica Scandinavica* 2013;92(s160):13. 111. Horan M, McGowan C, Donnelly J, Gibney E, McAuliffe F. Maternal diet and weight at 3 months partum following a pregnancy intervention with a low glycaemic index diet:Results from the ROLO randomised control trial. *Archives of Disease in Childhood. Fetal and Neonatal Edition* 2014;99 (Suppl 1):A129-A130, Abstract no: PMM.20. 112. Horan MK, McGowan CA, Donnelly J, Gibney E, McAuliffe FM. The association of maternal characteristics and macronutrient intake in pregnancy with neonatal body composition. *Archives of Disease in Childhood. Fetal and Neonatal Edition* 2014;99(Suppl 1):A11. 113. Horan MK, McGowan CA, Doyle O, McAuliffe FM. Well-being in pregnancy: An examination of the effect of socioeconomic, dietary and lifestyle factors including impact of a low glycaemic index dietary intervention. *European Journal of Clinical Nutrition* 2014;68(1):19–24. 114. Horan MK, McGowan CA, Gibney ER, Donnelly JM, McAuliffe FM. Maternal diet and weight at 3 months postpartum following a pregnancy intervention with a low glycaemic index diet: results from the ROLO randomised control trial. *Nutrients* 2014;6(7):2946–55. 115. Horan MK, McGowan CA, Gibney ER, Donnelly JM, McAuliffe FM. Maternal low glycaemic index diet, fat intake and postprandial glucose influences neonatal adiposity - secondary analysis from the ROLO study. *Nutrition Journal* 2014;13(1):78. 116. Mahony R, Byrne J, Curran S, O’Herlihy C, McAuliffe F. A pilot study of the feasibility of a randomised trial of low glycaemic diet versus normal diet from early pregnancy in euglycaemic women. *Archives of Disease in Childhood. Fetal and Neonatal Edition* 2008;93(Suppl 1):Fa38. 117. McAuliffe F. A randomised controlled trial of low glycaemic index carbohydrate diet versus no dietary intervention in the prevention of recurrence of foetal macrosomia. CurrentControlled Trials [(www.controlled-trials.com/)](https://www.dropbox.com/referrer_cleansing_redirect?hmac=YPGjIV707QXeIYSj6BVOrrnFGx%2Fopsn7NqhYIe5SKkk%3D&url=http%3A%2F%2F(www.controlled-trials.com%2F)) (accessed 12.05.2010). 118. McGowan CA, Walsh JM, Byrne J, Curran S, McAuliffe FM. The influence of a low glycemic index dietary intervention on maternal dietary intake, glycemic index and gestational weight gain during pregnancy: a randomized controlled trial. *Nutrition Journal* 2013;12(1):140. 119. Walsh J, Mahony R, Foley M, Mc Auliffe F. A randomised control trial of low glycaemic index carbohydrate diet versus no dietary intervention in the prevention of recurrence of macrosomia. *BMC Pregnancy and Childbirth*2010;10:16. 120. Walsh J, Mahony R, Foley M, McAuliffe F. ROLO study: a randomized control trial of low glycemic index diet to prevent macrosomia in euglycemic women. *American Journal of Obstetrics and Gynecology* 2012;206,Suppl 1):S4. 121. Walsh J, McGowan C, Byrne J, Foley M, Mahony R, McAuliffe F. The influence of a low glycaemic index dietary intervention on maternal glycaemic index, dietary intake and gestational weight gain. *American Journal of Obstetrics and Gynecology* 2013;208(1 Suppl):S33. 122. Walsh JM, Mahony RM, Canty G, Foley ME, McAuliffe FM. Identification of those most likely to benefit from a low-glycaemic index dietary intervention in pregnancy. *British Journal of Nutrition* 2014;112:583–9. 123. Walsh JM, McGowan CA, Mahony R, Foley ME, McAuliffe FM. Low glycaemic index diet in pregnancy to prevent macrosomia (ROLO study): randomised control trial. *BMJ* 2012;345:e5605. 124. Nilsson K. Weight gain during pregnancy - a randomized controlled trial of intervention to prevent excessive gestational weight gain. [ClinicalTrials.gov](https://www.dropbox.com/referrer_cleansing_redirect?hmac=5WLySbT4Hb7szRpbJ9U75Y6Z7NFaQ7F3XH0jwK6iJbM%3D&url=http%3A%2F%2FClinicalTrials.gov) (http://[clinicaltrials.gov/)](https://www.dropbox.com/referrer_cleansing_redirect?hmac=10ucmo0GUjXKZslchdxEyyW85phL%2FEV6I2qBkesMoEo%3D&url=http%3A%2F%2Fclinicaltrials.gov%2F)) [accessed 5 September 2014] 2009. 125. Ronnberg A, Ostlund I, Fadl H, Gottvall T, Nilsson K. Intervention during pregnancy to reduce excessive gestational weight gain-a randomised controlled trial. BJOG:an International Journal of Obstetrics and Gynaecology 2014 Nov 4 [Epub ahead of print]. 126. Ruchat SM, Davenport MH, Giroux I, Hillier M, Batada A, Sopper MM, et al. Nutrition and exercise reduce excessive weight gain in normal-weight pregnant women. *Medicine and Science in Sports and Exercise* 2012;44(8):1419–26. 127. Ruiz R, Perales M, Pelaez M, Lopez C, Lucia A, Barakat R. Supervised exercise-based intervention to prevent excessive gestational weight gain: a randomized controlled trial. *Mayo Clinic Proceedings* 2013;88(12):1388–97. 128. Santaella MP. The role of a supervised physical exercise program as an alternative on the control of maternal estational weight gain. [ClinicalTrials.gov](https://www.dropbox.com/referrer_cleansing_redirect?hmac=5WLySbT4Hb7szRpbJ9U75Y6Z7NFaQ7F3XH0jwK6iJbM%3D&url=http%3A%2F%2FClinicalTrials.gov) (accessed 21 May20 13). NCT01790347 2013. 129. Santos IA, Stein R, Fuchs SC, Duncan BB, Ribeiro JP, Kroeff LR, et al. Aerobic exercise and submaximal functional capacity in overweight pregnant women: a randomized trial. *Obstetrics & Gynecology* 2005;106(2):243–9. 130. Morkved S. Effects of regular exercise during pregnancy.[ClinicalTrials.gov](https://www.dropbox.com/referrer_cleansing_redirect?hmac=5WLySbT4Hb7szRpbJ9U75Y6Z7NFaQ7F3XH0jwK6iJbM%3D&url=http%3A%2F%2FClinicalTrials.gov)[(www.clinicaltrials.gov)](https://www.dropbox.com/referrer_cleansing_redirect?hmac=LJcCh%2BVT3tZ%2BaqSHfqJfboFykUDsduloFI4FX0k9Jcc%3D&url=http%3A%2F%2F(www.clinicaltrials.gov)) (accessed 31 October 2010). 131. Stafne SN, Salvesen KÅ, Romundstad PR, Eggebø TM, Carlsen SM, Mørkved S. Regular exercise during pregnancy to prevent gestational diabetes: a randomized controlled trial. *Obstetrics & Gynecology* 2012;119(1):29–36. 132. Szmeja MA, Grivell RM, Deussen AR, Dodd JM. Evaluation of information provision to women who are overweight or obese during pregnancy. *Journal of Paediatrics and Child Health* 2011;47(Suppl 1):78. 133. Thornton YS, Smarkola C, Kopacz SM, Ishoof SB. Perinatal outcomes in nutritionally monitored obese pregnant women: a randomized clinical trial. *Journal of the National Medical Association* 2009;101:569–77. 134. Vesco K, Leo M, Gillman M, King J, McEvoy C, Karanjaa N, et al. Impact of a weight management intervention on pregnancy outcomes among obese women: The Healthy Moms Trial. *American Journal of Obstetrics and Gynecology* 2013;208(1 Suppl 1):S352. 135. Vesco KK, Karanja N, King JC, Gillman MW, Leo C, Perrin N, et al. Efficacy of a group-based dietary intervention for limiting gestational weight gain among obese women: A randomized trial. *Obesity (Silver Spring, Md.)*2014;2(9):1989–96. 136. Vesco KK, Karanja N, King JC, Gillman MW, Perrin N, McEvoy C, et al. Healthy Moms, a randomized trial to promote and evaluate weight maintenance among obese pregnant women: study design and rationale. *Contemporary Clinical Trials* 2012;33(4):777–85. 137. Tanvig M. Lifestyle in pregnancy and offspring (LiPO). [ClinicalTrials.gov](https://www.dropbox.com/referrer_cleansing_redirect?hmac=5WLySbT4Hb7szRpbJ9U75Y6Z7NFaQ7F3XH0jwK6iJbM%3D&url=http%3A%2F%2FClinicalTrials.gov)[(http://clinicaltrials.gov/)](https://www.dropbox.com/referrer_cleansing_redirect?hmac=An6ki23nhchhSzR2Oqm6oQ%2FUvkNbI3ult6gA2vOtHTM%3D&url=http%3A%2F%2F(http%3A%2F%2Fclinicaltrials.gov%2F)) [accessed 6 February 2014] 2013. 138. Tanvig M. Lifestyle in pregnancy and offspring - comparison between children born to obese women and children born to normal weight women (LiPO). [ClinicalTrials.gov](https://www.dropbox.com/referrer_cleansing_redirect?hmac=5WLySbT4Hb7szRpbJ9U75Y6Z7NFaQ7F3XH0jwK6iJbM%3D&url=http%3A%2F%2FClinicalTrials.gov) (http:// [clinicaltrials.gov/)](https://www.dropbox.com/referrer_cleansing_redirect?hmac=10ucmo0GUjXKZslchdxEyyW85phL%2FEV6I2qBkesMoEo%3D&url=http%3A%2F%2Fclinicaltrials.gov%2F)) [accessed 6 February 2014] 2013. 139. Tanvig M. Offspring body size and metabolic profile - Effects of lifestyle intervention in obese pregnant women. *Danish Medical Journal* 2014;61(7):B4893. 140. Tanvig M, Vinter CA, Jorgensen JS, Wehberg S, Ovesen PG, Beck-Nielsen H, et al. Effects of lifestyle intervention in pregnancy and anthropometrics at birth on offspring metabolic profile at 2.8 years - results from the Lifestyle in Pregnancy and Offspring (LiPO) study. *Journal of Clinical Endocrinology and Metabolism* 2015;100(1):175–83. 141. Vinter A. Lifestyle and pregnancy: the clinical effect of lifestyle intervention during pregnancy in obese women. [ClinicalTrials.gov](https://www.dropbox.com/referrer_cleansing_redirect?hmac=5WLySbT4Hb7szRpbJ9U75Y6Z7NFaQ7F3XH0jwK6iJbM%3D&url=http%3A%2F%2FClinicalTrials.gov)[(www.clinicaltrials.gov)](https://www.dropbox.com/referrer_cleansing_redirect?hmac=LJcCh%2BVT3tZ%2BaqSHfqJfboFykUDsduloFI4FX0k9Jcc%3D&url=http%3A%2F%2F(www.clinicaltrials.gov)) [accessed 31 October 2010]. 142. Vinter C, Jensen D, Ovesen P, Beck-Nielsen H, Lamont R, Jorgensen J. Postpartum weight retention and breastfeeding among obese women from the LiP (Lifestyle in Pregnancy) Study. *Acta Obstetricia et Gynecologica Scandinavica* 2012;91(Suppl 159):141–2. 143. Vinter CA, Jensen DM, Ovesen P, Beck-Nielsen H, Jorgensen JS. The LiP (Lifestyle in Pregnancy) study: a randomized controlled trial of lifestyle intervention in 360 obese pregnant women. *Diabetes Care* 2011;34(12):2502–7. 144. Vinter CA, Jensen DM, Ovesen P, Beck-Nielsen H, Tanvig M, Lamont RF, et al. Postpartum weight retention and breastfeeding among obese women from the randomized controlled Lifestyle in Pregnancy (LiP) trial. *Acta Obstetricia et Gynecologica Scandinavica* 2014;93:794–801. 145. Vitolo MR. Impact of a nutritional intervention program for weight control during pregnancy. [ClinicalTrials.gov](https://www.dropbox.com/referrer_cleansing_redirect?hmac=5WLySbT4Hb7szRpbJ9U75Y6Z7NFaQ7F3XH0jwK6iJbM%3D&url=http%3A%2F%2FClinicalTrials.gov) [(www.clinicaltrials.gov)](https://www.dropbox.com/referrer_cleansing_redirect?hmac=LJcCh%2BVT3tZ%2BaqSHfqJfboFykUDsduloFI4FX0k9Jcc%3D&url=http%3A%2F%2F(www.clinicaltrials.gov)) [accessed 31 October 2010]. 146. Vitolo MR, Fraga Bueno MS, Mendes Gama C. Impact of a dietary counseling program on the gain weight speed of pregnant women attended in a primary care service. *Revista Brasileira de Ginecologia e Obstetricia* 2011;33(1):13–9. 147. Wilkinson SA, McIntyre HD. Evaluation of the ’healthy start to pregnancy’ early antenatal health promotion workshop: a randomized controlled trial. *BMC Pregnancy and Childbirth* 2012;12:131. 148. Wolff S, Legarth J, Vangsgaard K, Toubro S, Astrup A. A randomized trial of the effects of dietary counseling on gestational weight gain and glucose metabolism in obese pregnant women. *International Journal of Obesity* 2008;32(3):495–501   **Note: There are more references than studies included as several papers are reported for some studies** |
| Nasciment  *et al.* 2012[28] | 1. Stafne SN, Salvesen KA, Romundstad PR, et al. Does regular exercise influence lumbopelvic pain? A randomized controlled trial. Acta Obstet Gynecol Scand 2012; 91:552–559. 2. Kluge J, Hall D, Louw Q, et al. Specific exercises to treat pregnancy-related low back pain in a South African population. Int J Gynecol Obstet 2011; 113:187–191. 3. Robledo-Colonia AF, Sandoval-Restrepo N, Mosquera-Valderrama YF, et al. Aerobic exercise training during pregnancy reduces depressive symptoms in nulliparous women: a randomized clinical trial. J Physiother 2012; 58:9–15. 4. Songøygard KM, Stafne SN, Evensen KA, et al. Does exercise during pregnancy prevent postnatal depression? A randomized controlled trial. Acta Obstet Gynecol Scand 2012; 91:62–67. 5. Hui A, Back L, Ludwig S, et al. Lifestyle intervention on diet and exercise reduced excessive gestational weight gain in pregnant women under a randomised controlled trial. BJOG 2012; 119:70–77. 6. Nascimento SL, Surita FG, Parpinelli MA, et al. The effect of an antenatal physical exercise programme on maternal/perinatal outcomes and quality of life in overweight and obese pregnant women: a randomized clinical trial. BJOG 2011; 118:1455–1463. 7. Haakstad LA, Bø K. Effect of regular exercise on prevention of excessive weight gain in pregnancy: a randomised controlled trial. Eur J Contracept Reprod Healthcare 2011; 16:116–125. 8. Phelan S, Phipps MG, Abrams B, et al. Randomized trial of a behavioral intervention to prevent excessive gestational weight gain: the Fit for Delivery Study. Am J Clin Nutr 2011; 93:772–779. 9. Stafne SN, Salvesen KA˚ , Romundstad PR, et al. Regular exercise during pregnancy to prevent gestational diabetes a randomized controlled trial. Obstet Gynecol 2012; 119:29–36. 10. Barakat R, Cordero Y, Coteron J, et al. Exercise during pregnancy improves maternal glucose screen at 24–28 weeks: a randomised controlled trial. Br J Sports Med 2012; 46:656–661. 11. de Barros MC, Lopes MA, Francisco RP, et al. Resistance exercise and glycemic control in women with gestational diabetes mellitus. Am J Obstet Gynecol 2010; 203:556.e1–e6. 12. Ko PC, Liang CC, Chang SD, et al. A randomized controlled trial of antenatal pelvic floor exercises to prevent and treat urinary incontinence. Int Urogynecol J 2011; 22:17–22. 13. Mason L, Roe B, Wong H, et al. The role of antenatal pelvic floor muscle exercises in prevention of postpartum stress incontinence: a randomised controlled trial. J Clin Nurs 2010; 19:2777–2786. 14. Bø K, Haakstad LA. Is pelvic floor muscle training effective when taught in a general fitness class in pregnancy? A randomised controlled trial. Physiotherapy 2011; 97:190–195. 15. Ramı´rez-Ve´lez R, Aguilar de Plata AC, Escudero MM, et al. Influence of regular aerobic exercise on endothelium-dependent vasodilation and cardiorespiratory fitness in pregnant women. J Obstet Gynaecol Res 2011; 37:1601– 1608. 16. Barakat R, Pelaez M, Montejo R, et al. Exercise during pregnancy improves maternal health perception: a randomized controlled trial. Am J Obstet Gynecol 2011; 204:402.e1–7. 17. Vallim AL, Osis MJ, Cecatti JG, et al. Water exercises and quality of life during pregnancy. Reprod Health 2011; 8:14. 18. Montoya Arizabaleta AV, Orozco Buitrago L, Aguilar de Plata AC, et al. Aerobic exercise during pregnancy improves health-related quality of life: a randomised trial. J Physiother 2010; 56:253–258. 19. Haakstad LA, Bø K. Exercise in pregnant women and birth weight: a randomized controlled trial. BMC Pregnancy Childbirth 2011; 11:66. |
| O’Brien  *et al.* 2014[29] | 1. Nagle C, Skouteris H, Morris h, Nankervis A, Rasmussen B, Mayall P et al. Primary prevention of gestational diabetes for women who are overweight and obese: a randomised controlled trial. BMC Pregnancy Childbirth 2013; 13: 65. 2. Jackson RA, Stotland NE, Caughey AB, Gerbert B. Improving diet and exercise in pregnancy with Video Doctor counseling: a randomized trial. Patient Educ Couns 2011; 83: 203–209. 3. ClinicalTrials.gov. Electronically-mediated weight interventions for pregnant and postpartum women. National Library of Medicine: Bethesda, MD, USA. 2013. Identifier NCT01331564. Available from http://clinicaltrials.gov/ct2/show/record/ NCT01331564. 4. ClinicalTrials.gov. Personalised management of body weight during pregnancy. National Library of Medicine: Bethesda, MD, USA. 2012. Identifier NCT01610752. Available from <http://www.clinicaltrials.gov/ct2/show/NCT01610752>. 5. ClinicalTrials.gov The MOTHER (Mobile Technologies to Help Enhancing Regular Physical Activity) trial for pregnant women. National Library of Medicine: Bethesda, MD, USA. 2011. Identifier NCT01461707. Available from http://www. clinicaltrials.gov/ct2/show/record/NCT01461707 6. van Zutphen M, Milder IE, Bemelmans WJ. Usage of an online healthy lifestyle program by pregnant women attending midwifery practices in Amsterdam. Prev Med 2008; 46: 552–557. 7. Bot M, Milder IE, Bemelmans WJ. Nationwide implementation of Hello World: a Dutch email-based health promotion program for pregnant women. J Med Internet Res 2009; 11: e24. |
| Lau *et al.* 2017[30] | 1. Albright CL, Steffen AD, Wilkens LR et al. Effectiveness of a 12-month randomized clinical trial to increase physical activity in multiethnic postpartum women: results from Hawaii’s Na Mikimiki Project. Prev Med 2014; 69: 214–223. 2. Bertz F, Brekke HK, Ellegård L, Rasmussen KM, Wennergren M, Winkvist A. Diet and exercise weight-loss trial in lactating overweight and obese women. Am J Clin Nutr 2012; 96: 698–705 3. Bertz F, Winkvist A, Brekke HK. Sustainable weight loss among overweight and obese lactating women is achieved with an energy-reduced diet in line with dietary recommendations: results from the LEVA randomized controlled trial. J Acad Nutr Diet 2015; 115: 78–8 4. Choi J, Lee J, Vittinghoff E, Fukuoka Y. mHealth Physical Activity Intervention: a randomized pilot study in physically inactive pregnant women. Matern Child Health J 2016; 20: 1091–1101. 5. Colleran HL, Lovelady CA. Use of mypyramid menu planner for moms in a weight-loss intervention during lactation. J Acad Nutr Diet 2012; 112: 553–558 6. Fjeldsoe BS, Miller YD, Marshall AL. MobileMums: a randomized controlled trial of an SMS-based physical activity intervention. Ann Behav Med 2010; 39: 101–111. 7. Harrison CL, Lombard CB, Strauss BJ, Teede HJ. Optimizing healthy gestational weight gain in women at high risk of gestational diabetes: a randomized controlled trial. Obesity 2013; 21: 904–909. 8. Harrison CL, Lombard CB, Teede HJ. Limiting postpartum weight retention through early antenatal intervention: the HeLPher randomised controlled trial. Int J Behav Nutr Phys Act 2014; 11: 1–8. 9. Herring SJ, Cruice JF, Bennett GG, Davey A, Foster GD. Using technology to promote postpartum weight loss in urban, lowincome mothers: a pilot randomized controlled trial. J Nutr Educ Behav 2014; 46: 610–615. 10. Herring SJ, Cruice JF, Bennett GG, Rose MZ, Davey A, Foster GD. Preventing excessive gestational weight gain among African American women: a randomized clinical trial. Obesity 2016; 24: 30–36. 11. Hui AL, Ludwig SM, Gardiner P et al. Community-based exercise and dietary intervention during pregnancy: a pilot study. Can J Diabetes 2006; 30: 169–175. 12. Jackson RA, Stotland NE, Caughey AB, Gerbert B. Improving diet and exercise in pregnancy with video doctor counseling: a randomized trial. Patient Educ Couns 2011; 83: 203–209. 13. Phelan S, Phipps MG, Abrams B, Darroch F, Schaffner A, Wing RR. Randomized trial of a behavioral intervention to prevent excessive gestational weight gain: the Fit for delivery study. Am J Clin Nutr 2011; 93: 772–779. 14. Phelan S, Phipps MG, Abrams B et al. Does behavioural intervention in pregnancy reduce postpartum weight retention? Twelve-month outcomes of the fit for delivery randomized trial. Am J Clin Nutr 2014; 99: 302–311. 15. Pollak KI, Alexander SC, Bennett G et al. Weight-related SMS texts promoting appropriate pregnancy weight gain: a pilot study. Patient Educ Couns 2014; 97: 256–260 16. Poston L, Bell R, Croker H et al. Effect of a behavioural intervention in obese pregnant women (the UPBEAT study): a multicentre, randomised controlled trial. Lancet Diabetes Endocrinol 2015; 3: 767–777. 17. Smith KM. The Blossom Project Online: use of a behaviorallybased website to promote physical activity and prevent excessive gestational weight gain in previously sedentary pregnant women. Graduate Theses and Dissertations 2014: 14051 |
| Shepherd *et al* 2017[31] | 1. Asbee SM, Jenkins TR, Butler JR, White J, Elliot M, Rutledge A. Dietary counseling prevents excessive weight gain during pregnancy, a randomized controlled trial. Obstetrics and Gynecology 2008;111(4 Suppl):6S.? 2. Asbee SM, Jenkins TR, Butler JR, White J, Elliot M, Rutledge A. Preventing excessive weight gain during pregnancy through dietary and lifestyle counseling: a randomized controlled trial. Obstetrics and Gynecology 2009;113(2 Pt 1):305–12.?NCT00792480. Does dietary and lifestyle counseling prevent excessive weight gain during pregnancy? A randomized controlled trial (WIP). https://clinicaltrials.gov/ ct2/show/NCT00792480 (first received 14 November 2008). 3. Bruno R, Petrella E, Bertarini V, Pedrielli G, Neri I, Facchinetti F. Adherence to a lifestyle programme in overweight/obese pregnant women and effect on gestational diabetes mellitus: a randomized controlled trial. Maternal and Child Nutrition 2016 Sep 19 [Epub ahead of print]. [DOI: 10.1111/mcn.12333?NCT01783210. Pregnancy complications in women with BMI > 25kg/m2 enrolled in a healthy lifestyle and eating habits program. clinicaltrials.gov/ct2/show/NCT01783210 (first received 28 January 2013). 4. Dodd 2014 Limiting weight gain in overweight and obese women during pregnancy to improve health outcomes: a randomised trial. anzctr.org.au/Trial/ Registration/TrialReview.aspx?id=81642 (first received 12 October 2006).? 5. Cramp CS, Moran LJ, Deussen AR, Yelland LN, Dodd JM. Evaluation of printed nutrition education material in overweight and obese women during pregnancy-findings from the LIMIT randomised trial. Journal of Paediatrics and Child Health 2013;49 Suppl 2:118.? 6. Dodd J. Obesity in pregnancy-the LIMIT randomised trial. Journal of Paediatrics and Child Health 2013;49 Suppl 2:4. Dodd JM. Dietary and lifestyle advice for pregnant women who are overweight or obese: the LIMIT randomized trial. Annals of Nutrition and Metabolism 2014;64(3-4):197–202. 7. Dodd JM, Cramp C, Sui Z, Yelland LN, Deussen AR, Grivell RM, et al. The effects of antenatal dietary and lifestyle advice for women who are overweight or obese on maternal diet and physical activity: the LIMIT randomised trial. BMC Medicine 2014;12(1):161.? 8. Dodd JM, Deussen AR, Mohamad I, Rifas-Shiman SL, Yelland LN, Louise J, et al. The effect of antenatal lifestyle advice for women who are overweight or obese on secondary measures of neonatal body composition: the LIMIT randomised trial. BJOG: an international journal of obstetrics and gynaecology 2016;123(2):244–53. 9. Dodd JM, Kannieappan LM, Grivell RM, Deussen AR, Moran LJ, Yelland LN, et al. Effects of an antenatal dietary intervention on maternal anthropometric measures in pregnant women with obesity. Obesity 2015;23(8): 1555–62. 10. Dodd JM, McPhee AJ, Turnbull D, Yelland LN, Deussen AR, Grivell RM, et al. The effects of antenatal dietary and lifestyle advice for women who are overweight or obese on neonatal health outcomes: the LIMIT randomised trial. BMC Medicine 2014;12(1):163. 11. Dodd JM, Newman A, Moran LJ, Deussen AR, Grivell RM, Yelland LN, et al. The effect of antenatal dietary and lifestyle advice for women who are overweight or obese on emotional well-being: the LIMIT randomized trial. Acta Obstetricia et Gynecologica Scandinavica 2016;95(3): 309–18. 12. Dodd JM, O’Brien CM, Grivell RM. Modifying diet and physical activity to support pregnant women who are overweight or obese. Current Opinion in Clinical Nutrition and Metabolic Care 2015;18(3):318–23. 13. Dodd JM, Turnbull D, McPhee AJ, Deussen AR, Grivell RM, Yelland LN, et al. Antenatal lifestyle advice for women who are overweight or obese: LIMIT randomised trial. BMJ 2014;348:g1285. 14. Dodd JM, Turnbull D, McPhee AJ, Deussen AR, Grivell RM, Yelland LN, et al. Dietary and physical activity interventions for women who are overweight or obese during pregnancy - the findings of the LIMIT randomized trial. The Power of Programming 2014: International Conference on Developmental Origins of Adiposity and Long-Term Health; 2014 March 13-15; Munich, women on maternal and infant outcomes. Journal of Paediatrics and Child Health 2013;49 Suppl 2:19.? 15. Grivell R, Yelland L, Earl RA, Staehr CJ, Dodd J. The effect of antenatal dietary and lifestyle advice on fetal body composition in women who are overweight or obese: findings from the LIMIT randomised trial. Ultrasound in Obstetrics and Gynecology 2013;42(Suppl 1):10. 16. Grivell R, Yelland L, Staehr CJ, Earl RA, Dodd J. The effect of antenatal dietary and lifestyle advice on fetal growth?in women who are overweight or obese: findings from?the LIMIT randomised trial. Ultrasound in Obstetrics and Gynecology 2013;42(Suppl 1):83. 17. Grivell RM, Yelland L, Staehr C, Earl RA, Dodd JM. The effect of antenatal dietary and lifestyle advice on fetal body composition in women who are overweight or obese: Findings from the LIMIT randomised trial. The Power of Programming 2014: International Conference on Developmental Origins of Adiposity and Long-Term Health; 2014 March 13-15; Munich, Germany. 2014: 56–7. 18. Grivell RM, Yelland LN, Deussen A, Crowther CA, Dodd JM. Antenatal dietary and lifestyle advice for women who are overweight or obese and the effect on fetal growth and adiposity: the LIMIT randomised trial. BJOG: an international journal of obstetrics & gynaecology 2016;123 (2):233–43. 19. Kannieappan LM, Deussen AR, Moran LJ, Grivell RM, Yelland LN, Dodd JM. The effect of antenatal dietary advice on maternal body composition in women who are overweight or obese - findings from the LIMIT randomised trial. Journal of Paediatrics and Child Health 2013;49 Suppl 2:94. 20. Newman AK, Deussen AR, Moran LJ, Grivell RM, Yelland LN, Turnbull D, et al. The effect of antenatal dietary and lifestyle advice on maternal psychological health in women who are overweight or obese-findings from the LIMIT randomised trial. Journal of Paediatrics and Child Health 2013;49 Suppl 2:119. 21. Sui Z, Yelland LN, Turnbull D, Dodd JM. Walking to limit gestational weight gain and keep fit during pregnancy - findings from the walk randomised trial. Journal of Paediatrics and Child Health 2013;49 Suppl 2:120.? 22. Szmeja MA, Cramp C, Grivell RM, Deussen AR, Yelland LN, Dodd JM. Use of a DVD to provide dietary and lifestyle information to pregnant women who are overweight or obese: a nested randomised trial. BMC Pregnancy and Childbirth 2014;14(1):409.? 23. Szmeja MA, Grivell RM, Deussen AR, Dodd JM. Evaluation of information provision to women who are overweight or obese during pregnancy. Journal of Paediatrics Germany. 2014:22–3. 24. Dodd JM, Turnbull DA, McPhee AJ, Wittert G, Crowther CA, Robinson JS. Limiting weight gain in overweight and obese women during pregnancy to improve health outcomes: the LIMIT randomised controlled trial. BMC Pregnancy and Childbirth 2011;11:79. 25. Eames AJ, Grivell RM, Dodd JM, Deussen A. The effect of limited gestational weight gain in overweight and obese 26. El Beltagy N, El Deen SS, Mohamed R. Does physical activity and diet control reduce the risk of developing gestational diabetes mellitus in Egypt? A randomized controlled trial. Journal of Perinatal Medicine 2013;41 (Suppl 1):1176. 27. Harrison 2013 ACTRN12608000233325. Healthier lifestyles: preventing gestational diabetes in high risk pregnancies: a research and education project. anzctr.org.au/Trial/Registration/ TrialReview.aspx?ACTRN=12608000233325 (first received 5 May 2008).? 28. Harrison CL, Lombard CB, Gibson-Helm M, Deeks?A, Teede HJ. Limiting excess weight gain in high-risk pregnancies: A randomized controlled trial. Endocrine Reviews 2011;32(3 Meeting Abstracts):P1–466.? 29. Harrison CL, Lombard CB, Strauss BJ, Teede HJ. Optimizing healthy gestational weight gain in women at high risk of gestational diabetes: a randomized controlled trial. Obesity 2013;21(5):904–9.? 30. Harrison CL, Lombard CB, Teede HJ. Limiting postpartum weight retention through early antenatal intervention: the HeLP-her randomised controlled trial. International Journal of Behavioral Nutrition and Physical Activity 2014;11(1): 134.? 31. Harrison CL, Teede HJ, Lombard CB. How effective is self- weighing in the setting of a lifestyle intervention to reduce gestational weight gain and postpartum weight retention??. Australian and New Zealand Journal of Obstetrics and Gynaecology 2014;54:382–5.? 32. Lombard C, Harrison C, Teede H. A randomized controlled trial investigating self-weighing and the prevention of excess weight gain in early pregnancy. Endocrine Reviews 2011;32 (3 Meeting Abstracts):P2–768.? 33. Teede HJ, Harrison CL, Gibson-Helm M, Lombard CB. Improving physical activity in high-risk pregnancies: A randomized controlled trial. Endocrine Reviews 2011;32(3 Meeting Abstracts):P1–467. 34. Hawkins M, Hosker M, Marcus BH, Rosal MC, Braun B, Stanek EJ, et al. A pregnancy lifestyle intervention?to prevent gestational diabetes risk factors in overweight Hispanic women: a feasibility randomized controlled trial. Diabetic Medicine 2014;32:108–15.?NCT01141582. A lifestyle intervention to prevent gestational diabetes. clinicaltrials.gov/archive/ NCT01141582/2010 06 09 (first received 2 August 2008). 35. Herring SJ, Cruice JF, Bennett GG, Rose MZ, Davey A, Foster GD. Preventing excessive gestational weight gain among African American women: a randomized clinical trial. Obesity 2016;24(1):30–6.?NCT01530776. Healthy4Baby: Preventing postpartum weight retention among low-income, black women. clinicaltrials.gov/ct2/show/NCT01530776 (first received 23 January 2012). 36. Hoirisch-Clapauch 2016 {published and unpublished data} Hoirisch-Clapauch S, Sant’Anna MCW, Moreira ECC, Frankel PP, Valle MP, D’Ippolito MM. A protocol combining daily walking and a low glycemic index diet increases the rate of take-home babies in women with consecutive first-trimester miscarriages. BJOG: an international journal of obstetrics and gynaecology 2016;123 (Suppl 2):74–5. 37. Hui A, Back L, Ludwig S, Gardiner P, Sevenhuysen G, Dean H, et al. Exercise and dietary intervention increases physical activity, promotes healthy diet and reduces excessive gestational weight gain in pregnant women: A randomized controlled trial in Urban community. Diabetes 2011;60: A351.? 38. Hui A, Back L, Ludwig S, Gardiner P, Sevenhuysen G, Dean H, et al. Lifestyle intervention on diet and exercise reduced excessive gestational weight gain in pregnant women under a randomised controlled trial. BJOG: an international journal of obstetrics and gynaecology 2012;119 (1):70–7.? 39. Hui AL, Ludwig S, Gardiner P, Sevenhuysen G, Dean H, Sellers E, et al. Exercise and dietary intervention during pregnancy results in reduced excessive gestational weight gain. Diabetes 2010;59(Suppl 1):A509.? 40. Hui AL, Ludwig SM, Gardiner P, Sevenhuysen G, Murray R, Morris M, et al. Community-based exercise and dietary intervention during pregnancy: a pilot study. Canadian Journal of Diabetes 2006;30(2):169–75. 41. Hui AL, Back L, Reid A, Sevenhuysen G, Ludwig S, Dean H, et al. Effects of physical activity and dietary intakes on weight gain of pregnant women with normal and above normal prepregnancy weight. Canadian Journal of Diabetes 2012;36(5 Suppl 1):S8.? 42. Hui AL, Ludwig S, Gardiner P, Sevenhuysen G, Dean HJ, Sellers E, et al. Effects of lifestyle intervention on dietary intake, physical activity level, and gestational weight gain in pregnant women with different pre-pregnancy Body Mass Index in a randomized control trial. BMC Pregnancy and Childbirth 2014;14(1):331.?NCT00486629. Impact of diet and exercise activity on pregnancy outcomes (IDEA). clinicaltrials.gov/ct2/show/ NCT00486629 (first received 12 June 2007). 43. Jing 2015 ChiCTR-IPR-15005809. The effect of a personalized intervention on weight gain and physical activity among pregnant women in China. chictr.org.cn/showprojen.aspx? proj=10240 (first received 12 January 2015).? 44. Jing W, Huang Y, Liu X, Luo B, Yang Y, Liao S. The effect of a personalized intervention on weight gain and physical activity among pregnant women in China. International Journal of Gynaecology and Obstetrics 2015;129(2):138–41. 45. Koivusalo 2016 {published data only}?Grotenfelt NE, Wasenius NS, Rono K, Laivuori H, Stach-Lempinen B, Orho-Melander M, et al. Interaction between rs10830963 polymorphism in mtnr1b and lifestyle intervention on occurrence of gestational diabetes. Diabetologia 2016;59(8):1655–8.? 46. Koivusalo SB, Rono K, Klemetti MM, Roine RP, Lindstrom J, Erkkola M, et al. Gestational diabetes mellitus can be prevented by lifestyle intervention: The Finnish gestational diabetes prevention study (RADIEL): A randomized controlled trial. Diabetes Care 2016;39:24–30. NCT01698385. Prevention of gestational diabetes through lifestyle modification (RADIEL) - a randomized controlled multi-centre intervention study. clinicaltrials.gov/ct2/show/ NCT01698385 (first received 11 September 2012). 47. Rono K, Stach-Lempinen B, Klemetti MM, Kaaja RJ, Poyhonen-Alho M, Eriksson JG, et al. Prevention of gestational diabetes through lifestyle intervention: study design and methods of a Finnish randomized controlled multicenter trial (RADIEL). BMC Pregnancy and Childbirth 2014;14:70. 48. Valkama A, Koivusalo S, Lindstrom J, Meinila J, Kautiainen H, Stach-Lempinen B, et al. The effect of dietary counselling on diet in pregnant women at risk for gestational diabetes. Annals of Nutrition and Metabolism 2015;67 (Suppl 1):138. 49. Valkama A, Koivusalo S, Lindstrom J, Meinila J, Kautiainen H, Stach-Lempinen B, et al. The effect of dietary counselling on food intakes in pregnant women at risk for gestational diabetes: a secondary analysis of a randomised controlled trial RADIEL. European Journal of Clinical Nutrition 2016;70(8):912–7. 50. Korpi-Hyovalti EA, Laaksonen DE, Schwab US, Vanhapiha TH, Vihla KR, Heinonen ST, et al. Feasibility of a lifestyle intervention in early pregnancy to prevent deterioration of glucose tolerance. BMC Public Health 2011;11:179.?NCT01130012. Prevention of gestational diabetes. clinicaltrials.gov/ct2/show/NCT01130012 (first received 11 May 2010). 51. Aittasalo M, Raitanen J, Kinnunen TI, Ojala K, Kolu P, Luoto R. Is intensive counseling in maternity care feasible and effective in promoting physical activity among women at risk for gestational diabetes? Secondary analysis of a cluster randomized NELLI study in Finland. International Journal of Behavioral Nutrition and Physical Activity 2012;9: 104.?ISRCTN33885819. Primary prevention of gestational diabetes among women at risk: a cluster-randomized controlled trial. isrctn.com/ISRCTN33885819 (first received 12 September 2007).? 52. Kinnunen TI, Puhkala J, Raitanen J, Ahonen S, Aittasalo M, Virtanen SM, et al. Effects of dietary counselling on food habits and dietary intake of Finnish pregnant women at increased risk for gestational diabetes - a secondary analysis of a cluster-randomized controlled trial. Maternal and Child Nutrition 2014;10(2):184–97.? 53. Kolu P, Raitanen J, Luoto R. Physical activity and health- related quality of life during pregnancy: a secondary analysis of a cluster-randomised trial. Maternal and Child Health Journal 2014;18(9):2098–105.? 54. Kolu P, Raitanen J, Rissanen P, Luoto R. Cost-effectiveness of lifestyle counselling as primary prevention of gestational diabetes mellitus: findings from a cluster-randomised trial. PLOS One 2013;8(2):e56392. 55. Kolu P, Raitanen J, Rissanen P, Luoto R. Health care costs associated with gestational diabetes mellitus among high-risk women - results from a randomised trial. BMC Pregnancy and Childbirth 2012;12:71.? 56. Leppanen M, Aittasalo M, Raitanen J, Kinnunen TI, Kujala UM, Luoto R. Physical activity during pregnancy: predictors of change, perceived support and barriers among women at increased risk of gestational diabetes. Maternal and Child Health Journal 2014;18(9):2158–66.? 57. Luoto R, Kinnunen TI, Aittasalo M, Kolu P, Raitanen J, Ojala K, et al. Primary prevention of gestational diabetes mellitus and large-for-gestational-age newborns by lifestyle counseling: a cluster-randomized controlled trial. PLOS Medicine 2011;8(5):1–11.? 58. Luoto RM, Kinnunen TI, Aittasalo M, Ojala K, Mansikkamaki K, Poropainen E, et al. Prevention of gestational diabetes: design of a cluster-randomized controlled trial and one-year follow-up. BMC Pregnancy and Childbirth 2010;10:39.? 59. Puhkala J, Luoto R, Ahotupa M, Raitanen J, Vasankari T. Postpartum weight retention is associated with elevated ratio of oxidized LDL lipids to HDL-cholesterol. Lipids 2013;48(12):1227–35. 60. Petrella E, Facchinetti F, Bertarini V, Pignatti L, Neri I, Battistini NC. Occurrence of pregnancy complications in women with BMI > 25 submitted to a healthy lifestyle and eating habits program. American Journal of Obstetrics and Gynecology 2013;208(1 Suppl):S33–4.? 61. Petrella E, Malavolti M, Bertarini V, Pignatti L, Neri I, Battistini NC, et al. Gestational weight gain in overweight and obese women enrolled in a healthy lifestyle and eating habits program. Journal of Maternal-Fetal and Neonatal Medicine 2014;27(13):1348–52. 62. Hagobian TA, Phelan S, Gorin AA, Phipps MG, Abrams B, Wing RR. Effects of maternal lifestyle intervention during pregnancy on untreated partner weight: results from fit for delivery study. Obesity 2016;24(1):23–5.?NCT01117961. Promoting healthy weight gain during pregnancy. clinicaltrials.gov/ct2/show/NCT01117961 (first received 15 April 2010).? 63. Phelan S, Phipps MG, Abrams B, Darroch F, Grantham?K, Schaffner A, et al. Does behavioral intervention in pregnancy reduce postpartum weight retention? Twelve- month outcomes of the Fit for Delivery randomized trial. American Journal of Clinical Nutrition 2014;99(2):302–12. 64. Phelan S, Phipps MG, Abrams B, Darroch F, Schaffner A, Wing RR. Factors associated with success in the ”fit for delivery“ intervention to reduce excessive gestational weight gain. Obesity 2011;19(Suppl 1):S95.? 65. Phelan S, Phipps MG, Abrams B, Darroch F, Schaffner A, Wing RR. Randomized trial of a behavioral intervention to prevent excessive gestational weight gain: the Fit for Delivery Study. American Journal of Clinical Nutrition 2011;93(4):772–9.?Phelan S, Phipps MG, Abrams B, Darroch F, Schafner A, Wing RR. Randomized trial of a behavioral intervention to prevent excessive gestational weight gain: The fit for delivery study. Obesity 2010;18(Suppl 2):S68. 66. Polley BA, Wing RR, Sims CJ. Randomized controlled trial to prevent excessive weight gain in pregnant women. International Journal of Obesity and Related Metabolic Disorders 2002;26(11):1494–502. 67. Hayes L, Bell R, Robson S, Poston L. Association between physical activity in obese pregnant women and pregnancy outcomes: The UPBEAT pilot study. Annals of Nutrition and Metabolism 2014;64(3-4):239–46.? 68. Hayes L, Mcparlin C, Kinnunen TI, Poston L, Robson SC, Bell R. Change in level of physical activity during pregnancy in obese women: findings from the UPBEAT pilot trial. BMC Pregnancy and Childbirth 2015;15:52.? 69. Maitland RA, Barr S, Briley A, Seed P, Poston L. Incidence of gestational diabetes in an obese population using the International Association of Diabetes and Pregnancy Study Groups (IADPSG) criteria in the UK Pregnancies Better Eating and Activity Trial (UPBEAT) pilot study. Diabetic Medicine 2012;29(Suppl 1):152.? 70. Poston L, Briley AL, Barr S, Bell R, Croker H, Coxon?K, et al. Developing a complex intervention for diet and activity behaviour change in obese pregnant women (the UPBEAT trial); assessment of behavioural change and process evaluation in a pilot randomised controlled trial. BMC Pregnancy and Childbirth 2013;13(1):148.? 71. Poston L, Holmes B, Kinnunen T, Croker H, Bell R, Sanders T, et al. A complex intervention to improve outcome in obese pregnancies; the upbeat study. Archives of Disease in Childhood: Fetal and Neonatal Edition 2011;96 (Suppl 1):Fa97.? 72. Schneeberger C, Flynn A, Barr S, Seed PT, Inskip HM, Poston L. Maternal diet patterns and glycaemic load in obese pregnant women taking part in a pilot trial of a lifestyle intervention (the upbeat trial). Diabetes 2014; Vol. 63:A342. 73. Briley A, Seed P, Singh C, Patel N, Poston L. Gestational weight gain in obese pregnant women, the impact of?a lifestyle intervention and implications for guidelines (UPBEAT trial). BJOG: an international journal of obstetrics and gynaecology 2016;123(Suppl 1):55–6.? 74. Briley A, Seed PT, Singh C, Patel N, Poston L. Gestational weight gain, BMI and pregnancy outcomes in obese pregnant women in the UPBEAT behavioural intervention RCT. Reproductive Sciences 2016;23(1 Suppl 1):277A. 75. Briley AL, Barr S, Badger S, Bell R, Croker H, Godfrey KM, et al. A complex intervention to improve pregnancy outcome in obese women; the UPBEAT randomised controlled trial. BMC Pregnancy and Childbirth 2014;14 (1):74. 76. Briley AL, Barr S, Badger S, Bell R, Croker H, Godfrey KM, et al. Erratum: A complex intervention to improve pregnancy outcome in obese women; the UPBEAT randomised controlled trial. BMC Pregnancy and Childbirth 2015;15:111. 77. Hayes L, Bell R, Robson S, Poston L. UPBEAT study: Association between physical activity in obese pregnant women and health of the offspring. The Power of Programming 2014: International Conference on Developmental Origins of Adiposity and Long-Term Health; 2014 March 13-15; Munich, Germany. 2014:48. ISRCTN89971375. UK Pregnancies Better Eating and Activity Trial. isrctn.com/ISRCTN89971375 (first received 23 July 2008). 78. Patel N, Godfrey KM, Pasupathy D, Levin J, Flynn AC, Hayes L, et al. Infant adiposity following a randomised controlled trial of a behavioural intervention in obese pregnancy. International Journal of Obesity 2017 Mar 21 [Epub ahead of print]. [DOI: 10.1038/ijo.2017.44 79. Patel NR, Pasupathy D, Flynn AC, Hayes L, Levin JG, Singh C, et al. The UPBEAT behavioural intervention in obese pregnant women - maternal and infant follow-up 6 months postpartum. Reproductive Sciences 2016;23(Suppl 1):71A. 80. Poston L. The UPBEAT study: A lifestyle intervention?in obese pregnant women. The Power of Programming 2014: International Conference on Developmental Origins of Adiposity and Long-Term Health; 2014 March 13-15; Munich, Germany. 2014:22.? 81. Poston L, Bell R, Croker H, Flynn AC, Godfrey KM, Goff L, et al. Effect of a behavioural intervention in obese pregnant women (the UPBEAT study): a multicentre, randomised controlled trial. Lancet. Diabetes and Endocrinology 2015;3(10):767–77.? 82. Seed PT, Briley A, Singh C, Patel N, Poston L. A novel method for devising optimal gestational weight gain in obese pregnant women. Reproductive Sciences 2016; Vol. 23, issue 1 Suppl 1:276A.? 83. Vieira MC, Pasupathy D, Patel NR, White SL, Briley A, Seed PT, et al. Factors associated with uncomplicated pregnancy in obese women from the UPBEAT trial. Reproductive Sciences 2016;23(Suppl 1):109A.?White L, Pasupathy D, Vieira MC, Briley AL, Seed P, Lawlor DA, et al. Prediction of gestational diabetes (GDM) in obese women. Reproductive Sciences 2016;23(Suppl 1): 124A. 84. Rauh 2013 DRKS00003801. Feasibility of a lifestyle intervention?in pregnancy to optimize maternal weight development. drks-neu.uniklinik-freiburg.de/drks web/navigate.do? navigationId=trial.HTML&TRIAL ID=DRKS00003801 (first received 24 April 2012).? 85. Rauh K, Gabriel E, Kerschbaum E, Schuster T, von Kries R, Amann-Gassner U, et al. Safety and efficacy of a lifestyle intervention for pregnant women to prevent excessive maternal weight gain: a cluster-randomized controlled trial. BMC Pregnancy and Childbirth 2013;13(1):151. 86. Rauh K, Gunther J, Kunath J, Stecher L, Hauner H. Lifestyle intervention to prevent excessive maternal weight gain: mother and infant follow-up at 12 months postpartum. BMC Pregnancy and Childbirth 2015;15:265. 87. Hillesund ER, Bere E, Sagedal LR, Vistad I, Overby NC. Effect of a diet intervention during pregnancy on dietary behavior in the randomized controlled Norwegian Fit for Delivery study. Journal of Developmental Origins of Health and Disease 2016;7(5):538–47.?NCT01001689. Fit for delivery: a study of the effect of exercise sessions and nutritional counselling on pregnancy outcome (FFF). clinicaltrials.gov/ct2/show/NCT01001689 (first received 26 October 2009).? 88. Sagedal LR. Who are we missing? Examining non- participants in ”Fit for Delivery“, a randomized, controlled trial of a lifestyle intervention in pregnancy. Pregnancy Hypertension 2014;4(3):237.? 89. Sagedal LR, Henriksen T, Overby NC, Lohne-Seiler?H, Torstveit MK, Bere E, et al. The problem of non- participation: Who declined to participate in ”Fit for Delivery“, a randomized, controlled trial of a lifestyle intervention in pregnancy?. The Power of Programming 2014: International Conference on Developmental Origins of Adiposity and Long-Term Health; 2014 March 13-15; Munich, Germany. 2014:63.? 90. Sagedal LR, Overby N, Lohne-Seiler H, Bere E, Torstveit M, Henriksen T, et al. Study protocol: Fit for Delivery - can a lifestyle intervention in pregnancy result in measurable health benefits for mothers and newborns? A randomized controlled trial. BMC Public Health 2013;13:132.? 91. Sagedal LR, Overby NC, Bere E, Torstveit MK, Lohne- Seiler H, Smastuen M, et al. Lifestyle intervention to limit gestational weight gain: the Norwegian Fit for Delivery randomised controlled trial. BJOG: an international journal of obstetrics and gynaecology 2017;124(1):97–109.? 92. Sagedal LR, Sanda B, Overby NC, Bere E, Torstveit MK, Lohne-Seiler H, et al. The effect of prenatal lifestyle intervention on weight retention 12 months postpartum: results of the Norwegian Fit for Delivery randomised controlled trial. BJOG: an international journal of obstetrics and gynaecology 2017;124(1):111–21. 93. Vinter 2011 NCT00530439. Lifestyle and pregnancy: the clinical effect of lifestyle intervention during pregnancy in obese women (LiP). clinicaltrials.gov/ct2/show/NCT00530439 (first received 13 September 2007).?NCT01918319. Lifestyle in pregnancy and offspring (LiPO). https://clinicaltrials.gov/ct2/show/NCT01918319 (first received 2 August 2013).?NCT01918423. Lifestyle in pregnancy and offspring?- comparison between children born to obese women?and children born to normal weight women (LiPO). clinicaltrials.gov/ct2/show/NCT01918423 (first received 2 August 2013).? 94. Tanvig M. Offspring body size and metabolic profile - Effects of lifestyle intervention in obese pregnant women. Danish Medical Journal 2014;61(7):B4893. 95. Tanvig M, Vinte CA, Jorgensen JS, Wehberg S, Ovesen PG, Lamont RF, et al. Anthropometrics and body composition by dual energy X-ray in children of obese women: a follow-up of a randomized controlled trial (the Lifestyle in Pregnancy and Offspring [LiPO] study). PLOS One 2014;9 (2):e89590. 96. Tanvig M, Vinter CA, Jorgensen JS, Wehberg S, Ovesen PG, Beck-Nielsen H, et al. Effects of lifestyle intervention in pregnancy and anthropometrics at birth on offspring metabolic profile at 2.8 years - results from the Lifestyle in Pregnancy and Offspring (LiPO) study. Journal of Clinical Endocrinology and Metabolism 2015;100(1):175–83.? 97. Vinter C, Jensen D, Ovesen P, Beck-Nielsen H, Lamont R, Jorgensen J. Postpartum weight retention and breastfeeding among obese women from the LiP (Lifestyle in Pregnancy) Study. Acta Obstetricia et Gynecologica Scandinavica 2012; 91(Suppl 159):141–2. 98. Vinter CA, Jensen DM, Ovesen P, Beck-Nielsen H, Jorgensen JS. The LiP (Lifestyle in Pregnancy) study: a randomized controlled trial of lifestyle intervention in 360 obese pregnant women. Diabetes Care 2011;34(12): 2502–7. 99. Vinter CA, Jensen DM, Ovesen P, Beck-Nielsen H, Tanvig M, Lamont RF, et al. Postpartum weight retention and breastfeeding among obese women from the randomized controlled Lifestyle in Pregnancy (LiP) trial. Acta Obstetricia et Gynecologica Scandinavica 2014;93:794–801. 100. Vinter CA, Jensen DM, Ovesen PG, Beck-Nielsen H, Jorgensen JS. Lifestyle and pregnancy (LIP) study: The clinical effect of lifestyle intervention during pregnancy in obese women. Diabetes 2011;60:A348–9. 101. Vinter CA, Jørgensen JS, Ovesen P, Beck-Nielsen H, Skytthe A, Jensen DM. Metabolic effects of lifestyle intervention in obese pregnant women. results from the randomized controlled trial ’lifestyle in pregnancy’ (LiP). Diabetic Medicine 2014;31(11):1323–30. 102. Wang S, Ma JM, Yang HX. Lifestyle intervention for gestational diabetes mellitus prevention: A cluster- randomized controlled study. Chronic Diseases and Translational Medicine 2015;1(3):169–74. |
| Sherifali *et al* 2017[32] | 1. Pollak KI, Alexander SC, Bennett G, Lyna P, Coffman CJ, Bilheimer A, et al. Weight-related SMS texts promoting appropriate pregnancy weight gain: a pilot study. Patient Educ Couns 2014 Nov;97(2):256-260 [FREE Full text] [doi: 10.1016/j.pec.2014.07.030] [Medline: 25153313] 2. Smith K, Lanningham-Foster L, Welch A, Campbell C. Web-based behavioral intervention increases maternal exercise but does not prevent excessive gestational weight gain in previously sedentary women. J Phys Act Health 2016 Jun;13(6):587-593. [doi: 10.1123/jpah.2015-0219] [Medline: 26594820] 3. Herring SJ, Cruice JF, Bennett GG, Rose MZ, Davey A, Foster GD. Preventing excessive gestational weight gain among African American women: a randomized clinical trial. Obesity (Silver Spring) 2016 Jan;24(1):30-36 [FREE Full text] [doi: 10.1002/oby.21240] [Medline: 26592857] 4. Pérez-Ferre N, Galindo M, Fernández MD, Velasco V, Runkle I, de la Cruz MJ, et al. The outcomes of gestational diabetes mellitus after a telecare approach are not inferior to traditional outpatient clinic visits. Int J Endocrinol 2010;2010:386941 [FREE Full text] [doi: 10.1155/2010/386941] [Medline: 20628517] 5. Carral F, Ayala Mdel C, Fernández JJ, González C, Piñero A, García G, et al. Web-based telemedicine system is useful for monitoring glucose control in pregnant women with diabetes. Diabetes Technol Ther 2015 May;17(5):349-354. [doi: 10.1089/dia.2014.0223] [Medline: 25629547] 6. Soltani H, Duxbury AM, Arden MA, Dearden A, Furness PJ, Garland C. Maternal obesity management using mobile technology: a feasibility study to evaluate a text messaging based complex intervention during pregnancy. J Obes 2015;2015:814830 [FREE Full text] [doi: 10.1155/2015/814830] [Medline: 25960889] |
| Tieu *et al* 2017[33] | 1. Clapp JF. Influence of endurance exercise and diet on human placental development and fetal growth. Placenta 2006;27(6-7):527–34 2. Clapp JF III. Diet, exercise, and feto-placental growth. Archives of Gynecology and Obstetrics 1997;260:101–8 3. Clapp JF III. Effect of dietary carbohydrate on the glucose and insulin response to mixed caloric intake and exercise in both nonpregnant and pregnant women. Diabetes Care 1998;21(Suppl 2):B107–B112. Clapp JF III. Maternal carbohydrate intake and pregnancy outcome. Proceedings of the Nutrition Society 2002;61: 45–50 4. Fraser RB. High fibre diets in pregnancy. Nutrition in Pregnancy. Proceedings of 10th Study Group of the Royal College of Obstetricians and Gynaecologists; 1982 September. London: RCOG, 1983:269–80 5. Fraser RB, Ford FA, Milner RDG. A controlled trial of a high dietary fibre intake in pregnancy - effects on plasma glucose and insulin levels. Diabetologia 1983;25:238–41 6. Hoppu U, Isolauri E, Koskinen P, Laitinen K. Diet and blood lipids in 1-4 year-old children. Nutrition Metabolism and Cardiovascular Diseases 2013;23(10):980–6 7. Hoppu U, Isolauri E, Laakso P, Matomaki J, Laitinen K. Probiotics and dietary counselling targeting maternal dietary fat intake modifies breast milk fatty acids and cytokines. European Journal of Nutrition 2012;51(2):211–9 8. Laitinen K, Ilmonen J, Isolauri E. Dietary counselling and probiotic intervention during pregnancy modify postpartum adiposity. Annals of Nutrition and Metabolism 2011;58(Suppl 3):87 9. Laitinen K, Poussa T, Isolauri E, Nutrition, Allergy, Mucosal Immunology and Intestinal Microbiota Group. Probiotics and dietary counselling contribute to glucose regulation during and after pregnancy: a randomised controlled trial. British Journal of Nutrition 2009;101(11): 1679–87 10. Luoto R, Laitinen K, Nermes M, Isolauri E. Impact of maternal probiotic-supplemented dietary counseling during pregnancy on colostrum adiponectin concentration: A prospective, randomized, placebo-controlled study. Early Human Development 2012;88(6):339–44 11. Luoto R, Laitinen K, Nermes M, Isolauri E. Impact of maternal probiotic-supplemented dietary counselling on pregnancy outcome and prenatal and postnatal growth: a double-blind, placebo-controlled study. British Journal of Nutrition 2010;103(12):1792–9 12. Luoto R, Nermes M, Laitinen K, Isolauri E. Impact of maternal probiotic-supplemented dietary counselling on pregnancy outcome and prenatal and postnatal growth: a double-blind, placebo-controlled study. Pediatric Academic Societies Annual Meeting; 2009 May 2-5; Baltimore, USA. 2009 13. Niinivirta K, Isolauri E, Laakso P, Linderborg K, Laitinen K. Dietary counseling to improve fat quality during pregnancy alters maternal fat intake and infant essential fatty acid status. Journal of Nutrition 2011;141(7):1281–5 14. Niinivirta K, Laakso P, Linderborg K, Poussa T, Isolauri E, Laitinen K. Maternal dietary counseling during pregnancy and infant fatty acid profiles. International Journal of Food Sciences and Nutrition 2014;65(3):268–72 15. Piirainen T, Isolauri E, Lagstrom H, Laitinen K. Impact of dietary counselling on nutrient intake during pregnancy: a prospective cohort study. British Journal of Nutrition 2006; 96(6):1095–104 16. Vahamiko S, Isolauri E, Laitinen K. Weight status and dietary intake determine serum leptin concentrations in pregnant and lactating women and their infants. British Journal of Nutrition 2013;110(6):1098–106 17. Markovic 2016 ACTRN12610000681055. A randomized, two-arm parallel dietary intervention study to compare the effects of consuming a low glycemic diet or wholegrain high fibre diet on infant birth weight and body composition, complications related to Gestational Diabetes Mellitus (GDM) and progression to GDM diagnosis in women at high-risk of GDM. anzctr.org.au/Trial/Registration/ TrialReview.aspx?id=335632 Date first received: 18 August 2010 18. Kizirian N, Garnett S, Markovic T, Ross G, Louie J, Muirhead R, et al. Effects of a low-glycaemic index diet during pregnancy on offspring body composition: a pilot study. Obesity Research and Clinical Practice 2013;7(Suppl 2):e103 19. Kizirian N, Garnett S, Markovic T, Ross G, Muirhead R, Brodie S, et al. Maternal diet and infant body composition in women at risk of gestational diabetes mellitus. Obesity Research and Clinical Practice 2014;8(Suppl 1):55 20. Markovic TP, Muirhead R, Overs S, Kizirian N, Louie J, Sweeting A, et al. Predictors of birthweight in women at high risk of gestational diabetes mellitus. Obesity Research and Clinical Practice 2013;7(2):e3–4 21. Markovic TP, Muirhead R, Overs S, Ross GP, Louie JC, Kizirian N, et al. Randomized controlled trial investigating the effects of a low-glycemic index diet on pregnancy outcomes in women at high risk of gestational diabetes mellitus: the GI Baby 3 Study. Diabetes Care 2016;39(1): 31–8 22. Moses RG, Luebcke M, Davis WS, Coleman KJ, Tapsell LC, Petocz P, et al. Effect of a low-glycemic-index diet during pregnancy on obstetric outcomes. American Journal of Clinical Nutrition 2006;84(4):807–12 23. Moses RG, Luebke M, Petocz P, Brand-Miller JC. Maternal diet and infant size 2 y after the completion of a study of a low-glycemic-index diet in pregnancy. American Journal of Clinical Nutrition 2007;86(6):1806 24. Goletzke J, Buyken AE, Louie JC, Moses RG, BrandMiller JC. Dietary micronutrient intake during pregnancy is a function of carbohydrate quality. American Journal of Clinical Nutrition 2015;102(3):626–32 25. Moses RG, Casey S, Cleary J, Milosavljevic M, Quinn E, Tapsell L, et al. Effect of low glycaemic index dietary advice in normal pregnancy: The PREGGIO study. Obesity Research and Clinical Practice 2013;7:e34–5 26. Moses RG, Casey SA, Quinn EG, Cleary JM, Tapsell LC, Milosavljevic M, et al. Pregnancy and Glycemic Index Outcomes study: effects of low glycemic index compared with conventional dietary advice on selected pregnancy outcomes. American Journal of Clinical Nutrition 2014;99 (3):517–23 27. Quinlivan J. A randomised trial of a multidisciplinary teamcare approach involving obstetric, dietary and clinical psychological input in obese pregnant women to reduce the incidence of gestational diabetes. https:// www.anzctr.org.au/Trial/Registration/TrialReview.aspx?id= 821 (accessed 2 November 2015) 28. Quinlivan JA, Lam LT, Fisher J. A randomised trial of a four-step multidisciplinary approach to the antenatal care of obese pregnant women. Australian and New Zealand Journal of Obstetrics and Gynaecology 2011;51(2):141–6 29. Thornton YS, Smarkola C, Kopacz SM, Ishoof SB. Perinatal outcomes in nutritionally monitored obese pregnant women: a randomized clinical trial. Journal of the National Medical Association 2009;101(6):569–77 30. Vitolo MR, Fraga Bueno MS, Mendes Gama C. Impact of a dietary counseling program on the gain weight speed of pregnant women attended in a primary care service. Revista Brasileira de Ginecologia e Obstetricia 2011;33(1):13–9 31. Donnelly J, Horan M, Walsh J, McGowan C, Byrne J, Molloy EJ, et al. Impact of a low GI diet on neonatal body composition (ROLO Kids). Pediatric Academic Societies Annual Meeting; 2013 May 4-7; Washington DC, USA. 2013 32. Donnelly JM, Lindsay KL, Walsh JM, Horan M, Molloy EJ, McAuliffe FM. Fetal metabolic influences of neonatal anthropometry and adiposity. BMC Pediatrics 2015;15(1): 175 33. Donnelly JM, Walsh JM, Byrne J, Molloy E, McAuliffe FM. Altered neonatal anthropometric measurements following maternal low GI diet in pregnancy (ROLO study). Acta Obstetricia et Gynecologica Scandinavica 2013;92(s160):13 34. Donnelly JM, Walsh JM, Byrne J, Molloy EJ, McAuliffe FM. Impact of maternal diet on neonatal anthropometry: A randomized controlled trial. Pediatric Obesity 2015;10(1): 52–6 35. Donnelly JMT, Lindsay KL, Walsh JM, Horan M, Molloy EJ, McAuliffe F. Impact of maternal and fetal inflammatory markers on neonatal and infant adiposity. Archives of Disease in Childhood 2015;100:A133–4 36. Horan M, Donnelly J, Gibney E, McGowan C, McAuliffe F. The association of maternal characteristics and macronutrient intake in pregnancy with neonatal body composition. The Power of Programming 2014: International Conference on Developmental Origins of Adiposity and Long-Term Health; 2014 March 13-15; Munich, Germany. 2014 37. Horan M, McGowan C, Donnelly J, Gibney E, McAuliffe F. Maternal diet and weight at 3 months partum following a pregnancy intervention with a low glycaemic index diet: Results from the ROLO randomised control trial. Archives of Disease in Childhood. Fetal and Neonatal Edition 2014;99 (Suppl 1):A129-A130, Abstract no: PMM.20 38. Horan MK, McGowan CA, Donnelly J, Gibney E, McAuliffe FM. The association of maternal characteristics and macronutrient intake in pregnancy with neonatal body composition. Archives of Disease in Childhood. Fetal and Neonatal Edition 2014;99(Suppl 1):A11 39. Horan MK, McGowan CA, Doyle O, McAuliffe FM. Well-being in pregnancy: An examination of the effect of socioeconomic, dietary and lifestyle factors including impact of a low glycaemic index dietary intervention. European Journal of Clinical Nutrition 2014;68(1):19–24 40. Horan MK, McGowan CA, Gibney ER, Donnelly JM, McAuliffe FM. Maternal diet and weight at 3 months postpartum following a pregnancy intervention with a low glycaemic index diet: results from the ROLO randomised control trial. Nutrients 2014;6(7):2946–55 41. Horan MK, McGowan CA, Gibney ER, Donnelly JM, McAuliffe FM. Maternal low glycaemic index diet, fat intake and postprandial glucose influences neonatal adiposity - secondary analysis from the ROLO study. Nutrition Journal 2014;13(1):78. ISRCTN54392969. A randomised controlled trial of low glycaemic index carbohydrate diet versus no dietary intervention in the prevention of recurrence of foetal macrosomia. controlled-trials.com/ISRCTN54392969 Date first received: 10 August 2009 42. Mahony R, Byrne J, Curran S, O’Herlihy C, McAuliffe F. A pilot study of the feasibility of a randomised trial of low glycaemic diet versus normal diet from early pregnancy in euglycaemic women. Archives of Disease in Childhood. Fetal and Neonatal Edition 2008;93(Suppl 1):Fa38 43. McAuliffe F. Maternal nutrition and fetal health. The Power of Programming 2014: International Conference on Developmental Origins of Adiposity and Long-Term Health; 2014 March 13-15; Munich, Germany. 2014 44. McGowan CA, Walsh JM, Byrne J, Curran S, McAuliffe FM. The influence of a low glycemic index dietary intervention on maternal dietary intake, glycemic index and gestational weight gain during pregnancy: a randomized controlled trial. Nutrition Journal 2013;12(1):140 45. Walsh J, Mahony R, Foley M, Mc Auliffe F. A randomised control trial of low glycaemic index carbohydrate diet versus no dietary intervention in the prevention of recurrence of macrosomia. BMC Pregnancy and Childbirth 2010;10:16 46. Walsh J, Mahony R, Foley M, McAuliffe F. ROLO study: a randomized control trial of low glycemic index diet to prevent macrosomia in euglycemic women. American Journal of Obstetrics and Gynecology 2012;206(Suppl 1):S4 47. Walsh J, McGowan C, Byrne J, Foley M, Mahony R, McAuliffe F. The influence of a low glycaemic index dietary intervention on maternal glycaemic index, dietary intake and gestational weight gain. American Journal of Obstetrics and Gynecology 2013;208(1 Suppl):S33 48. Walsh JM, Mahony RM, Canty G, Foley ME, McAuliffe FM. Identification of those most likely to benefit from a low-glycaemic index dietary intervention in pregnancy. British Journal of Nutrition 2014;112:583–9 49. Walsh JM, Mahony RM, Culliton M, Foley ME, McAuliffe FM. Impact of a low glycemic index diet in pregnancy on markers of maternal and fetal metabolism and inflammation. Reproductive Sciences 2014;21(11):1378–81 50. Walsh JM, McAuliffe FM. Impact of maternal nutrition on pregnancy outcome - Does it matter what pregnant women eat? Best Practice and Research. Clinical Obstetrics and Gynaecology 2015;29(1):63–78 51. Walsh JM, McGowan CA, Mahony R, Foley ME, McAuliffe FM. Low glycaemic index diet in pregnancy to prevent macrosomia (ROLO study): randomised control trial. BMJ 2012;345:e5605. 52. Wolff S, Legarth J, Vangsgaard K, Toubro S, Astrup A. A randomized trial of the effects of dietary counseling on gestational weight gain and glucose metabolism in obese pregnant women. International Journal of Obesity 2008;32 (3):495–501 |
| Currie *et al* 2013[34] | 1. Callaway LK, Colditz PB, Byrne NM, Lingwood BE, Rowlands IJ, et al. (2010) Prevention of gestational diabetes feasibility issues for an exercise intervention in obese pregnant women. Diabetes Care 33: 1457–1459. 2. Gaston A, Prapavessis H (2009) Maternal-fetal disease information as a source of exercise motivation during pregnancy. Health Psychology 28: 726. 3. Haakstad LA, Bø K (2011) Effect of regular exercise on prevention of excessive weight gain in pregnancy: A randomised controlled trial. The European Journal of Contraception and Reproductive Health Care 16: 116–125. 4. Ong M, Guelfi K, Hunter T, Wallman K, Fournier P, et al. (2009) Supervised home-based exercise may attenuate the decline of glucose tolerance in obese pregnant women. Diabetes Metab 35: 418–421. 5. Oostdam N, van Poppel M, Wouters M, Eekhoff E, Bekedam D, et al. (2012) No effect of the FitFor2 exercise programme on blood glucose, insulin sensitivity,and birthweight in pregnant women who were overweight and at risk for gestational diabetes: Results of a randomised controlled trial. BJOG: An International Journal of Obstetrics & Gynaecology: 1098–1107. 6. Rankin J (2002) Effects of antenatal exercise on psychological well-being.pregnancy and birth outcome. 7. Yeo S (2009) Adherence to walking or stretching, and risk of preeclampsia in sedentary pregnant women. Res Nurs Health 32: 379–390. 8. Chasan-Taber L, Silveira M, Marcus BH, Braun B, Stanek E, et al. (2011) Feasibility and efficacy of a physical activity intervention among pregnant women: The behaviors affecting baby and you (BABY) study. Journal of physical activity & health 8: S228. 9. Guelinckx I, Devlieger R, Mullie P, Vansant G (2010) Effect of lifestyle intervention on dietary habits, physical activity, and gestational weight gain in obese pregnant women: A randomized controlled trial. Am J Clin Nutr 91: 373–380. 10. Shen GX (2006) Community-based exercise and dietary intervention during pregnancy: A pilot study. Canadian Journal of diabetes 30: 169–175. 11. Huang T, Yeh C, Tsai Y (2011) A diet and physical activity intervention for preventing weight retention among Taiwanese childbearing women: A randomised controlled trial. Midwifery 27: 257–264. 12. Jackson RA, Stotland NE, Caughey AB, Gerbert B (2011) Improving diet and exercise in pregnancy with video doctor counseling: A randomized trial. Patient Educ Couns 83: 203–209. 13. Luoto R, Kinnunen TI, Aittasalo M, Kolu P, Raitanen J, et al. (2011) Primary prevention of gestational diabetes mellitus and large-for-gestational-age newborns by lifestyle counseling: A cluster-randomized controlled trial. PLoS medicine 8: e1001036. 14. Polley B, Wing R, Sims C (2002) Randomized controlled trial to prevent excessive weight gain in pregnant women. Int J Obes 26: 1494–1502. |
| Chan et al 2019[35] | 1. Kinnunen, T.I.; Pasanen, M.; Aittasalo, M.; Fogelholm, M.; Hilakivi-Clarke, L.; Weiderpass, E.; Luoto, R. Preventing excessive weight gain during pregnancy—A controlled trial in primary health care. Eur. J. Clin. Nutr. 2007, 61, 884–891. 2. Ronnberg, A.K.; Ostlund, I.; Fadl, H.; Gottvall, T.; Nilsson, K. Intervention during pregnancy to reduce excessive gestational weight gain—A randomised controlled trial. BJOG 2015, 122, 537–544. 3. Miquelutti, M.A.; Cecatti, J.G.; Makuch, M.Y. Evaluation of a birth preparation program on lumbopelvic pain, urinary incontinence, anxiety and exercise: A randomized controlled trial. BMC Pregnancy Childbirth 2013, 13, 154. 4. Da Silva, S.G.; Hallal, P.C.; Domingues, M.R.; Bertoldi, A.D.; Silveira, M.F.D.; Bassani, D.; da Silva, I.C.M.; da Silva, B.G.C.; Coll, C.V.N.; Evenson, K. A randomized controlled trial of exercise during pregnancy on maternal and neonatal outcomes: Results from the PAMELA study. Int. J. Behav. Nutr. Phys. Act. 2017, 14, 175. 5. Hui, A.; Back, L.; Ludwig, S.; Gardiner, P.; Sevenhuysen, G.; Dean, H.; Sellers, E.; McGavock, J.; Morris, M.; Bruce, S.; et al. Lifestyle intervention on diet and exercise reduced excessive gestational weight gain in pregnant women under a randomised controlled trial. BJOG 2012, 119, 70–77 6. Arizabaleta, A.V.M.; Buitrago, L.O.; de Plata, A.C.A.; Escudero, M.M.; Ramirez-Velez, R. Aerobic exercise during pregnancy improves health-related quality of life: A randomised trial. J. Physiother. 2010, 56, 253–258. 7. Robledo-Colonia, A.F.; Sandoval-Restrepo, N.; Mosquera-Valderrama, Y.F.; Escobar-Hurtado, C.; Ramírez-Vélez, R. Aerobic exercise training during pregnancy reduces depressive symptoms in nulliparous women: A randomised trial. J. Physiother. 2012, 58, 9–15. 8. Marquez-Sterling, S.; Perry, A.C.; Kaplan, T.A.; Halberstein, R.A.; Signorile, J.F. Physical and psychological changes with vigorous exercise in sedentary primigravidae. Med. Sci. Sports Exerc. 2000, 32, 58–62. 9. Garshasbi, A.; Faghih Zadeh, S. The effect of exercise on the intensity of low back pain in pregnant women. Int. J. Gynaecol. Obstet. 2005, 88, 271–275. 10. Ghodsi, Z.; Asltoghiri, M. Effects of aerobic exercise training on maternal and neonatal outcome: A randomized controlled trial on pregnant women in Iran. J. Pak. Med. Assoc. 2014, 64, 1053–1056 11. Gau, M.L.; Chang, C.Y.; Tian, S.H.; Lin, K.C. Effects of birth ball exercise on pain and self-efficacy during childbirth: A randomised controlled trial in Taiwan. Midwifery 2011, 27, e293–e300 12. Kluge, J.; Hall, D.; Louw, Q.; Theron, G.; Grové, D. Specific exercises to treat pregnancy-related low back pain in a South African population. Int. J. Gynaecol. Obstet. 2011, 113, 187–191 13. Suputtitada, A.; Wacharapreechanont, T.; Chaisayan, P. Effect of the “sitting pelvic tilt exercise” during the third trimester in primigravidas on back pain. J. Med. Assoc. Thail. 2002, 85 (Suppl. 1), S170–S179. 14. Huang, T.T.; Yeh, C.Y.; Tsai, Y.C. A diet and physical activity intervention for preventing weight retention among Taiwanese childbearing women: A randomised controlled trial. Midwifery 2011, 27, 257–264 15. Ozdemir, S.; Bebis, H.; Ortabag, T.; Acikel, C. Evaluation of the efficacy of an exercise program for pregnant women with low back and pelvic pain: A prospective randomized controlled trial. J. Adv. Nurs. 2015, 71, 1926–1939 16. Stafne, S.N.; Salvesen, K.A.; Romundstad, P.R.; Stuge, B.; Mørkved, S. Does regular exercise during pregnancy influence lumbopelvic pain? A randomized controlled trial. Acta Obstet. Gynecol. Scand. 2012, 91, 552–559 17. Songoygard, K.M.; Stafne, S.N.; Evensen, K.A.; Salvesen, K.A.; Vik, T.; Morkved, S. Does exercise during pregnancy prevent postnatal depression? A randomized controlled trial. Acta Obstet. Gynecol. Scand. 2012, 91, 62–67 18. Gustafsson, M.K.; Stafne, S.N.; Romundstad, P.R.; Mørkved, S.; Salvesen, K.; Helvik, A.S. The effects of an exercise program during pregnancy on health-related quality of life in pregnant women: A Norwegian randomised controlled trial. BJOG 2016, 123, 1152–1160. 19. Eggen, M.H.; Stuge, B.; Mowinckel, P.; Jensen, K.S.; Hagen, K.B. Can supervised group exercises including ergonomic advice reduce the prevalence and severity of low back pain and pelvic girdle pain in pregnancy? A randomized controlled trial. Phys. Ther. 2012, 92, 781–790 20. Sagedal, L.R.; Øverby, N.C.; Bere, E.; Torstveit, M.K.; Lohne-Seiler, H.; Småstuen, M.; Hillesund, E.R.; Henriksen, T.; Vistad, I. Lifestyle intervention to limit gestational weight gain: The Norwegian Fit for Delivery randomised controlled trial. BJOG 2017, 124, 97–109. 21. Haakstad, L.A.; Vistad, I.; Sagedal, L.R.; Lohne-Seiler, H.; Torstveit, M.K. How does a lifestyle intervention during pregnancy influence perceived barriers to leisure-time physical activity? The Norwegian fit for delivery study, a randomized controlled trial. BMC Pregnancy Childbirth 2018, 18, 127 22. Haakstad, L.A.; Bø, K. Effect of regular exercise on prevention of excessive weight gain in pregnancy: A randomised controlled trial. Eur. J. Contracept. Reprod. Health Care 2011, 16, 116–125 23. Haakstad, L.A.; Bø, K. Effect of a regular exercise program on pelvic girdle and low back pain in previously inactive pregnant women: A randomized controlled trial. J. Rehabil. Med. 2015, 47, 229–234 24. Haakstad, L.A.; Torset, B.; Bø, K. What is the effect of regular group exercise on maternal psychological outcomes and common pregnancy complaints? An assessor blinded RCT. Midwifery 2016, 32, 81–86 25. Perales, M.; Refoyo, I.; Coteron, J.; Bacchi, M.; Barakat, R. Exercise during pregnancy attenuates prenatal depression: A randomized controlled trial. Eval. Health Prof. 2015, 38, 59–72 26. Hui, A.; Ludwig, S.; Gardiner, P.; Sevenhuysen, G.; Murray, R.; Morris, M.; Shen, G.X. Community-based exercise and dietary intervention during pregnancy: A pilot study. Can. J. Diabetes 2006, 30, 1–7 27. A¸scı, O.; Rathfisch, G. Effect of lifestyle interventions of pregnant women on their dietary habits, lifestyle behaviors, and weight gain: A randomized controlled trial. J. Health Popul. Nutr. 2016, 35, 7 28. Ruiz, J.R.; Perales, M.; Pelaez, M.; Lopez, C.; Lucia, A.; Barakat, R. Supervised exercise-based intervention to prevent excessive gestational weight gain: A randomized controlled trial. Mayo Clin. Proc. 2013, 88, 1388–1397 29. Kokic, I.S.; Ivanisevic, M.; Uremovic, M.; Kokic, T.; Pisot, R.; Simunic, B. Effect of therapeutic exercises on pregnancy-related low back pain and pelvic girdle pain: Secondary analysis of a randomized controlled trial. J. Rehabil. Med. 2017, 49, 251–257 |
| Flannery et al 2019[36] | 1. Callaway LK, Colditz PB, Byrne NM, Lingwood BE, Rowlands IJ, Foxcroft K, et al. Prevention of gestational diabetes feasibility issues for an exercise intervention in obese pregnant women. Diabetes Care. 2010;33(7):1457–9. 2. Hawkins M, Hosker M, Marcus BH, Rosal MC, Braun B, Stanek EJ 3rd, et al. A pregnancy lifestyle intervention to prevent gestational diabetes risk factors in overweight Hispanic women: a feasibility randomized controlled trial. Diabet Med. 2015;32(1):108–15 3. Kong KL, Campbell CG, Foster RC, Peterson AD, Lanningham-Foster L. A pilot walking program promotes moderate-intensity physical activity during pregnancy. Med Sci Sports Exerc. 2014;46(3):462–71 4. Guelinckx I, Devlieger R, Mullie P, Vansant G. Effect of lifestyle intervention on dietary habits, physical activity, and gestational weight gain in obese pregnant women: a randomized controlled trial. Am J Clin Nutr. 2009;91(2):373–80 5. Koivusalo SB, Rono K, Klemetti MM, Roine RP, Lindstrom J, Erkkola M, et al. Gestational diabetes mellitus can be prevented by lifestyle intervention: the Finnish gestational diabetes prevention study (RADIEL): a randomized controlled trial. Diabetes Care. 2016;39(1):24–30 6. Nascimento S, Surita F, Parpinelli M, Siani S, Pinto e Silva J. The effect of an antenatal physical exercise programme on maternal/perinatal outcomes and quality of life in overweight and obese pregnant women: a randomised clinical trial. BJOG. 2011;118(12):1455–63 7. Oostdam N, Van Poppel M, Wouters M, Eekhoff E, Bekedam D, Kuchenbecker W, et al. No effect of the FitFor2 exercise programme on blood glucose, insulin sensitivity, and birthweight in pregnant women who were overweight and at risk for gestational diabetes: Results of a randomised controlled trial. BJOG: an International Journal of Obstetrics and Gynaecology 2012;119(9):1098–107. 8. Vinter CA, Jensen DM, Ovesen P, Beck-Nielsen H, Jorgensen JS. The LiP (lifestyle in pregnancy) study: a randomized controlled trial of lifestyle intervention in 360 obese pregnant women. Diabetes Care. 2011;34(12):2502–7 9. Ong M, Guelfi K, Hunter T, Wallman K, Fournier P, Newnham J. Supervised home-based exercise may attenuate the decline of glucose tolerance in obese pregnant women. Diabetes Metab. 2009;35(5):418–21 10. Santos IA, Stein R, Fuchs SC, Duncan BB, Ribeiro JP, Kroeff LR, et al. Aerobic exercise and submaximal functional capacity in overweight pregnant women: a randomized trial. Obstet Gynecol. 2005;106(2):243–9 11. Kennelly MA, Ainscough K, Lindsay KL, O'Sullivan E, Gibney ER, McCarthy M, et al. Pregnancy exercise and nutrition with smartphone application support: a randomized controlled trial. Obstet Gynecol. 2018;131(5):818–26 12. Van Horn L, Peaceman A, Kwasny M, Vincent E, Fought A, Josefson J, et al. Dietary approaches to stop hypertension diet and activity to limit gestational weight: maternal offspring Metabolics family intervention trial, a technology enhanced randomized trial. Am J Prev Med. 2018;55(5):603–14 13. Dodd JM, Cramp C, Sui Z, Yelland LN, Deussen AR, Grivell RM, et al. The effects of antenatal dietary and lifestyle advice for women who are overweight or obese on maternal diet and physical activity: the LIMIT randomised trial. BMC Med. 2014;12(1):161 14. Poston L, Bell R, Croker H, Flynn AC, Godfrey KM, Goff L, et al. Effect of a behavioural intervention in obese pregnant women (the UPBEAT study): a multicentre, randomised controlled trial. Lancet Diab Endocrinol. 2015;3(10):767–77 15. Renault KM, Nørgaard K, Nilas L, Carlsen EM, Cortes D, Pryds O, et al. The Treatment of Obese Pregnant Women (TOP) study: a randomized controlled trial of the effect of physical activity intervention assessed by pedometer with or without dietary intervention in obese pregnant women. Am J Obstet Gynecol. 2014;210(2):134. e1–9. 16. Bruno R, Petrella E, Bertarini V, Pedrielli G, Neri I, Facchinetti F. Adherence to a lifestyle programme in overweight/obese pregnant women and effect on gestational diabetes mellitus: a randomized controlled trial. Maternal & Child Nutrition. 2017;13:e12333. https://doi.org/10.1111/mcn.12333 17. Seneviratne S, Jiang Y, Derraik J, McCowan L, Parry G, Biggs J, et al. Effects of antenatal exercise in overweight and obese pregnant women on maternal and perinatal outcomes: a randomised controlled trial. BJOG. 2016; 123(4):588–97. 18. Garnaes KK, Morkved S, Salvesen O, Moholdt T. Exercise training and weight gain in obese pregnant women: a randomized controlled trial (ETIP Trial) [with consumer summary]. PLoS Med. 2016;13(7):e1002079 19. Szmeja MA, Cramp C, Grivell RM, Deussen AR, Yelland LN, Dodd JM. Use of a DVD to provide dietary and lifestyle information to pregnant women who are overweight or obese: a nested randomised trial. BMC Pregnancy and Childbirth 2014;14(1):409 |

**S7f: Unique publications inlcuded in diet and/or physical activity reviews**

| **Diet and/or physical activity unique publications** |
| --- |
| 1. Althuizen E, Van Poppel MN, Seidell JC, Van der Wijden C, Van Mechelen W. Design of the new life(style) study: a randomised controlled trial to optimise maternal weight development during pregnancy. *BMC Public Health* 2006;6:168. |
| 1. Angel MD, De Haene J, Perez M, Hernandez G, Castaneda D, King JC. Dietary patterns associated with gestational weight gain and fat mass gain in overweight and obese pregnant women. *FASEB Journal* 2011;25:783.15. |
| 1. Szmeja MA, Grivell RM, Deussen AR, Dodd JM. Evaluation of information provision to women who are overweight or obese during pregnancy. *Journal of Paediatrics and Child Health* 2011;47(Suppl 1):78. |
| 1. A¸scı, O.; Rathfisch, G. Effect of lifestyle interventions of pregnant women on their dietary habits, lifestyle behaviors, and weight gain: A randomized controlled trial. J. Health Popul. Nutr. 2016, 35, 7 |
| 1. Abel R, Rajaratnam J, Kalaimani A, Kirubakaran S. Can iron status be improved in each of the three trimesters? A community-based study. European Journal of Clinical Nutrition 2000; 54:490–493. Nutrition education and counseling during pregnancy 201 © 2012 Blackwell Publishing Ltd Paediatric and Perinatal Epidemiology, 2012, 26 (Suppl. 1), 191–204 |
| 1. Adhikari K, Liabsuetrakul T, Pradhan N. Effect of education and pill count on hemoglobin status during prenatal care in Nepalese women: a randomized controlled trial. The Journal of Obstetrics and Gynaecology Research 2009; 35:459–466 |
| 1. Ahrari M, Houser RF, Yassin S, Mogheez M, Hussaini Y. A positive deviance-based antenatal nutrition project improves birth-weight in upper Egypt. Journal of Health, Population, and Nutrition 2006; 24:498–507. 23 |
| 1. Aittasalo M, Raitanen J, Kinnunen TI, Ojala K, Kolu P, Luoto R. Is intensive counseling in maternity care feasible and effective in promoting physical activity among women at risk for gestational diabetes? Secondary analysis of a cluster randomized NELLI study in Finland. International Journal of Behavioral Nutrition and Physical Activity 2012;9: 104.?ISRCTN33885819. Primary prevention of gestational diabetes among women at risk: a cluster-randomized controlled trial. isrctn.com/ISRCTN33885819 (first received 12 September 2007).? |
| 1. Albright CL, Steffen AD, Wilkens LR et al. Effectiveness of a 12-month randomized clinical trial to increase physical activity in multiethnic postpartum women: results from Hawaii’s Na Mikimiki Project. Prev Med 2014; 69: 214–223. |
| 1. Althuizen E, van der Wijden C, van Mechelen W, SeidellJ, van Poppel M. The effect of a counselling intervention on weight changes during and after pregnancy: a randomised trial. *BJOG : an International Journal of Obstetrics and Gynaecology*2013;120(1):92–9. |
| 1. Anderson AS, Campbell DM, Shepherd R. The influence of dietary advice on nutrient intake during pregnancy. The British Journal of Nutrition 1995; 73:163–177 |
| 1. Asbee SM, Jenkins TR, Butler JR, White J, Elliot M, Rutledge A. Dietary counseling prevents excessive weight gain during pregnancy, a randomized controlled trial. *Obstetrics & Gynecology* 2008;11(4 Suppl):6S. |
| 1. Asbee SM, Jenkins TR, ButlerJR, White J, Elliot M, Rutledge A. Preventing excessive weight gain during pregnancy through dietary and lifestyle counseling: a randomized controlled trial. *Obstetrics & Gynecology* 2009;113 (2 Pt 1):305–12. |
| 1. Barakat R, Pelaez M, Montejo R, Luaces M, Zakynthinaki M. Exercise during pregnancy improves maternal health perception: a randomized controlled trial. *American Journal of Obstetrics and Gynecology* 2011;204(5):402.e1–402.e7. |
| 1. Barakat R, Cordero Y, Coteron J, et al. Exercise during pregnancy improves maternal glucose screen at 24–28 weeks: a randomised controlled trial. Br J Sports Med 2012; 46:656–661. |
| 1. Bechtel-Blackwell DA. Computer-assisted self-interview and nutrition education in pregnant teens. Clinical Nursing Research 2002; 11:450–462 |
| 1. Belizan JM, Barros F, Langer A, Farnot U, Victora C, Villar J. Impact of health education during pregnancy on behavior and utilization of health resources. Latin American Network for Perinatal and Reproductive Research. American Journal of Obstetrics and Gynecology 1995; 173:894–899 |
| 1. Berry K, Wiehl DG. An experiment in diet education during pregnancy. The Milbank Memorial Fund Quarterly 1952; 30:119–151 |
| 1. Bertz F, Brekke HK, Ellegård L, Rasmussen KM, Wennergren M, Winkvist A. Diet and exercise weight-loss trial in lactating overweight and obese women. Am J Clin Nutr 2012; 96: 698–705 |
| 1. Bertz F, Winkvist A, Brekke HK. Sustainable weight loss among overweight and obese lactating women is achieved with an energy-reduced diet in line with dietary recommendations: results from the LEVA randomized controlled trial. J Acad Nutr Diet 2015; 115: 78–8 |
| 1. Bisson M, Almeras N, Dufresne S, Rheaume C, Bujold E, Robitailee J, et al. Exercise improving fitness in obese women during pregnancy: a difference for the mother and child?. Pediatric Academic Societies and Asian Society for Pediatric Research Joint Meeting; 2014 May 3-6;Vancouver, Canada. 2014:Abstract no: 2946.618. |
| 1. Bo K, Haakstad LAH. Is pelvic floor muscle training effective when taught in a general fitness class in pregnancy? A randomised controlled trial. *Physiotherapy* 2011;97(3):190–5. |
| 1. Bø K, Haakstad LA. Is pelvic floor muscle training effective when taught in a general fitness class in pregnancy? A randomised controlled trial. Physiotherapy 2011; 97:190–195. |
| 1. Bogaerts A, Devlieger R, Nuyts E, Witters I, Gyselaers W, Guelinckx I. Psycho-education reduces gestational weight gain in obese pregnant women: Randomized controlled trial [abstract]. *Obesity Facts* 2012;5:53. |
| 1. Bogaerts A. Effect of psycho-education on gestational weight gain and anxiety/depression in obese pregnant women. ClinicalTrials.gov(http://clinicaltrials.gov/) [accessed 21 May 2013] 2011. |
| 1. Bogaerts AF, Devlieger R, Nuyts E, et al. Effects of lifestyle intervention in obese pregnant women on gestational weight gain and mental health: a randomized controlled trial. Int J Obes. (Lond.). 2013;37:814–821 |
| 1. Bot M, Milder IE, Bemelmans WJ. Nationwide implementation of Hello World: a Dutch email-based health promotion program for pregnant women. J Med Internet Res 2009; 11: e24 |
| 1. Brand-Miller J. A pregnancy intervention to reduce postprandial glucose excursions in the primary prevention of paediatric obesity. Current Controlled Trials (www.controlled-trials.com/) [accessed 31 October 2010]. |
| 1. Briley AL, Barr S, Badger S, Bell R, Croker H, Godfrey KM, et al. A complex intervention to improve pregnancy outcome in obese women; the UPBEAT randomised controlled trial. *BMC Pregnancy and Childbirth* 2014;14(1):74. |
| 1. Briley A, Seed P, Singh C, Patel N, Poston L. Gestational weight gain in obese pregnant women, the impact of?a lifestyle intervention and implications for guidelines (UPBEAT trial). BJOG: an international journal of obstetrics and gynaecology 2016;123(Suppl 1):55–6.? |
| 1. Briley A, Seed PT, Singh C, Patel N, Poston L. Gestational weight gain, BMI and pregnancy outcomes in obese pregnant women in the UPBEAT behavioural intervention RCT. Reproductive Sciences 2016;23(1 Suppl 1):277A. |
| 1. Briley C, Flanagan NL, Lewis N. In-home prenatal nutrition intervention increased dietary iron intakes and reduced low birthweight in low-income African-American women. Journal of the American Dietetic Association 2002; 102:984–987 |
| 1. Broekhuizen K, Althuizen E, van Poppel MNM, Donker M, van Mechelen W. From theory to practice: intervention fidelity in a randomized controlled trial aiming to optimize weight development during pregnancy. *Health Promotion Practice* 2012;13(6):816–25. |
| 1. Bruno R, Petrella E, Bertarini V, Pedrielli G, Neri I, Facchinetti F. Adherence to a lifestyle programme in overweight/obese pregnant women and effect on gestational diabetes mellitus: a randomized controlled trial. Maternal and Child Nutrition 2016 Sep 19 [Epub ahead of print]. [DOI: 10.1111/mcn.12333?NCT01783210. Pregnancy complications in women with BMI > 25kg/m2 enrolled in a healthy lifestyle and eating habits program. clinicaltrials.gov/ct2/show/NCT01783210 (first received 28 January 2013). |
| 1. Byrne NM, Groves AM, McIntyre HD, Callaway LK. Changes in resting and walking energy expenditure and walking speed during pregnancy in obese women. *American Journal of Clinical Nutrition* 2011;94(3):819–30. |
| 1. Callaway L, McIntyre D, Colditz P, Byrne N, Foxcroft K, O’Connor B. Exercise in obese pregnant women: a randomized study to assess feasibility. *Hypertension in Pregnancy* 2008;27(4):549. |
| 1. Callaway L. A randomized controlled trial using exercise to reduce gestational diabetes and other adverse maternal and neonatal outcomes in obese pregnant women - the pilot study. Australian Clinical Trials Registry (www.actr.org.au/)[accessed 31 October 2010]. |
| 1. Callaway LK, Colditz PB, Byrne NM, Lingwood BE, Rowlands IJ, Foxcroft K, et al. Prevention of gestational diabetes. Feasibility issues for an exercise intervention in obese pregnant women. *Diabetes Care* 2010;33(7):1457–9. |
| 1. Carral F, Ayala Mdel C, Fernández JJ, González C, Piñero A, García G, et al. Web-based telemedicine system is useful for monitoring glucose control in pregnant women with diabetes. Diabetes Technol Ther 2015 May;17(5):349-354. [doi: 10.1089/dia.2014.0223] [Medline: 25629547] |
| 1. Chasan-Taber L, Silveira M, Marcus BH, Braun B, Stanek E, et al. (2011) Feasibility and efficacy of a physical activity intervention among pregnant women: The behaviors affecting baby and you (BABY) study. Journal of physical activity & health 8: S228. |
| 1. Choi J, Lee J, Vittinghoff E, Fukuoka Y. mHealth Physical Activity Intervention: a randomized pilot study in physically inactive pregnant women. Matern Child Health J 2016; 20: 1091–1101. |
| 1. Claesson IM, Sydsjö G, Brynhildsen J, Cedergren M, Jeppsson A, Nyström F, Sydsjö A, Josefsson A. Weight gain restriction for obese pregnant women: a case-control intervention study. Br J Obstet Gynaecol 2007; 115: 44–50. |
| 1. Clapp III JF. Maternal carbohydrate intake and pregnancy outcome. *Proceedings of the Nutrition Society* 2002; 61(1):45–50. |
| 1. Clapp IJF. Diet, exercise, and feto-placental growth. *Archives of Gynecology and Obstetrics* 1997;260:101–8. |
| 1. Clapp JF 3rd, Kim H, Burciu B, Schmidt S, Petry K, Lopez B. Continuing regular exercise during pregnancy: effect of exercise volume on fetoplacental growth. *American Journal of Obstetrics and Gynecology* 2002;186(1):142–7. |
| 1. Clapp JF III. Diet, exercise, and feto-placental growth. Archives of Gynecology and Obstetrics 1997;260:101–8 |
| 1. Clapp JF III. Effect of dietary carbohydrate on the glucose and insulin response to mixed caloric intake and exercise in both nonpregnant and pregnant women. Diabetes Care 1998;21(Suppl 2):B107–B112. Clapp JF III. Maternal carbohydrate intake and pregnancy outcome. Proceedings of the Nutrition Society 2002;61: 45–50 |
| 1. Clapp JF. Influence of endurance exercise and diet on human placental development and fetal growth. Placenta 2006;27(6-7):527–34 |
| 1. Colleran HL, Lovelady CA. Use of mypyramid menu planner for moms in a weight-loss intervention during lactation. J Acad Nutr Diet 2012; 112: 553–558 |
| 1. Cordero Y, Mottola MF, Vargas J, Blanco M, Barakat R. Exercise is associated with a reduction in gestational diabetes mellitus. Medicine and Science in Sports and Exercise 2014;Oct 20 [Epub ahead of print]. |
| 1. Cramp CS, Moran LJ, Deussen AR, Yelland LN, Dodd JM. Evaluation of printed nutrition education material in overweight and obese women during pregnancy-findings from the limit randomised trial. *Journal of Paediatrics and Child Health* 2013;49 Suppl 2:118. |
| 1. Da Silva, S.G.; Hallal, P.C.; Domingues, M.R.; Bertoldi, A.D.; Silveira, M.F.D.; Bassani, D.; da Silva, I.C.M.; da Silva, B.G.C.; Coll, C.V.N.; Evenson, K. A randomized controlled trial of exercise during pregnancy on maternal and neonatal outcomes: Results from the PAMELA study. Int. J. Behav. Nutr. Phys. Act. 2017, 14, 175. |
| 1. Daelhousen BB, Guthrie HA. A self-instruction nutrition program for pregnant women. Journal of the American Dietetic Association 1982; 81:407–412 |
| 1. De Oliveria Melo AS, Silva JLP, Tavares JS, Barros VO, Leite DFB, Amorim MMR. Effect of a physical exercise program during pregnancy on uteroplacental and fetal blood flow and fetal growth: A randomized controlled trial. *Obstetrics & Gynecology* 2012;120(2 Pt 1):302–10. |
| 1. de Barros MC, Lopes MA, Francisco RP, et al. Resistance exercise and glycemic control in women with gestational diabetes mellitus. Am J Obstet Gynecol 2010; 203:556.e1–e6. |
| 1. Di Carlo C, Iannotti G, Sparice S, Chiacchio MP, Greco E, Tommaselli GA, et al. The role of a personalized dietary intervention in managing gestational weight gain: a prospective, controlled study in a low-risk antenatal population. *Archives of Gynecology and Obstetrics* 2014;289:765–70. |
| 1. Dodd J. Obesity in pregnancy-the limit randomised trial. *Journal of Paediatrics and Child Health* 2013;49 Suppl 2:4. |
| 1. Dodd JM, Cramp C, Sui Z, Yelland LN, Deussen AR, Grivell RM, et al. The effects of antenatal dietary and lifestyle advice for women who are overweight or obese onmaternal diet and physical activity: the LIMIT randomised trial. *BMC Medicine* 2014;12(1):161. |
| 1. Dodd JM, McPhee AJ, Turnbull D, Yelland LN, Deussen AR, Grivell RM, et al. The effects of antenatal dietary and lifestyle advice for women who are overweight or obese on neonatal health outcomes: the LIMIT randomised trial. BMC Medicine 2014;12(1):163. |
| 1. Dodd JM, Turnbull D, McPhee AJ, Deussen AR, Grivell RM, Yelland LN, et al. Antenatal lifestyle advice for women who are overweight or obese: LIMIT randomised trial.*BMJ (Clinical Research Ed.)*2014;348:g1285. |
| 1. Dodd JM, Turnbull DA, McPhee AJ, Wittert G, Crowther CA, Robinson JS. Limiting weight gain in overweight and obese women during pregnancy to improve health outcomes: the LIMIT randomised controlled trial. *BMC Pregnancy and Childbirth* 2011;11:79. |
| 1. Dodd JM. Dietary and lifestyle advice for pregnant women who Are overweight or obese: the LIMIT randomized trial. *Annals of Nutrition & Metabolism* 2014;64(3-4):197–202. |
| 1. Dodd JM, Deussen AR, Mohamad I, Rifas-Shiman SL, Yelland LN, Louise J, et al. The effect of antenatal lifestyle advice for women who are overweight or obese on secondary measures of neonatal body composition: the LIMIT randomised trial. BJOG: an international journal of obstetrics and gynaecology 2016;123(2):244–53. |
| 1. Dodd JM, Kannieappan LM, Grivell RM, Deussen AR, Moran LJ, Yelland LN, et al. Effects of an antenatal dietary intervention on maternal anthropometric measures in pregnant women with obesity. Obesity 2015;23(8): 1555–62. |
| 1. Dodd JM, Newman A, Moran LJ, Deussen AR, Grivell RM, Yelland LN, et al. The effect of antenatal dietary and lifestyle advice for women who are overweight or obese on emotional well-being: the LIMIT randomized trial. Acta Obstetricia et Gynecologica Scandinavica 2016;95(3): 309–18. |
| 1. Dodd JM, O’Brien CM, Grivell RM. Modifying diet and physical activity to support pregnant women who are overweight or obese. Current Opinion in Clinical Nutrition and Metabolic Care 2015;18(3):318–23. |
| 1. Dodd JM, Turnbull D, McPhee AJ, Deussen AR, Grivell RM, Yelland LN, et al. Antenatal lifestyle advice for women who are overweight or obese: LIMIT randomised trial. BMJ 2014;348:g1285. |
| 1. Donnelly J, Horan M, Walsh J, McGowan C, Byrne J, Molloy EJ, et al. Impact of a low GI diet on neonatal body composition (ROLO Kids). Pediatric Academic Societies Annual Meeting; 2013 May 4-7; Washington DC, USA. 2013. |
| 1. Donnelly JM, Walsh JM, Byrne J, Molloy E, McAuliffe FM. Altered neonatal anthropometric measurements following maternal low GI diet in pregnancy (ROLO study). *Acta Obstetricia et Gynecologica Scandinavica* 2013;92(s160):13. |
| 1. Donnelly JM, Lindsay KL, Walsh JM, Horan M, Molloy EJ, McAuliffe FM. Fetal metabolic influences of neonatal anthropometry and adiposity. BMC Pediatrics 2015;15(1): 175 |
| 1. Donnelly JMT, Lindsay KL, Walsh JM, Horan M, Molloy EJ, McAuliffe F. Impact of maternal and fetal inflammatory markers on neonatal and infant adiposity. Archives of Disease in Childhood 2015;100:A133–4 |
| 1. Eames AJ, Grivell RM, Dodd JM, Deussen A. The effect of limited gestational weight gain in overweight and obese women on maternal and infant outcomes. *Journal of Paediatrics and Child Health* 2013;49 Suppl 2:19. |
| 1. Eggen, M.H.; Stuge, B.; Mowinckel, P.; Jensen, K.S.; Hagen, K.B. Can supervised group exercises including ergonomic advice reduce the prevalence and severity of low back pain and pelvic girdle pain in pregnancy? A randomized controlled trial. Phys. Ther. 2012, 92, 781–790 |
| 1. Ehrlich SF, Hedderson MM, Feng J, Crites Y, Quesenberry CP, Ferrara A. Lifestyle intervention improves postpartum fasting glucose levels in women with gestational diabetes. *Diabetes* 2014; 63 (Suppl 1):A95. |
| 1. El Beltagy N, El Deen SS, Mohamed R. Does physical activity and diet control reduce the risk of developing gestational diabetes mellitus in Egypt? A randomized controlled trial. Journal of Perinatal Medicine 2013;41 (Suppl 1):1176 |
| 1. el-Shabrawy Ali M. Effects of nutrition education on the dietary patterns of two socio-economic groups of pregnant females in Dakahlia Governorate (Egypt). The Journal of the Egyptian Medical Association 1971; 54:829–840 |
| 1. Ershoff DH, Aaronson NK, Danaher BG, Wasserman FW. Behavioral, health, and cost outcomes of an HMO-based prenatal health education program. Public Health Reports 1983; 98:536–547 |
| 1. Facchinetti F. Pregnancy complications in women with BMI > 25kg/m2 enrolled in a healthy lifestule and eating habits program. ClinicalTrials.gov (accessed 21 May 2013). NCT01783210 2013. |
| 1. Ferrara A, Hedderson MM, Albright CL, Ehrlich SF, Quesenberry CP, Peng T, et al. A pregnancy and postpartum lifestyle intervention in women with gestational diabetes mellitus reduces diabetes risk factors: a feasibility randomized control trial. *Diabetes Care* 2011;34(7):1519–25. |
| 1. Ferrara A. Diet, exercise and breastfeeding intervention program for women with gestational diabetes (DEBI Trial). ClinicalTrials.gov(www.clinicaltrials.gov) [accessed 31 October 2010]. |
| 1. Fjeldsoe BS, Miller YD, Marshall AL. MobileMums: a randomized controlled trial of an SMS-based physical activity intervention. Ann Behav Med 2010; 39: 101–111. |
| 1. Foxcroft KF, Rowlands IJ, Byrne NM, McIntyre HD, Callaway LK, for the BAMBINO group. Exercise in obese pregnant women: the role of social factors, lifestyle and pregnancy symptoms. *BMC Pregnancy and Childbirth* 2011;11:4. |
| 1. Fraser RB, Ford FA, Milner RDG. A controlled trial of a high dietary fibre intake in pregnancy - effects on plasma glucose and insulin levels. Diabetologia 1983;25:238–41 |
| 1. Fraser RB. High fibre diets in pregnancy. Nutrition in Pregnancy. Proceedings of 10th Study Group of the Royal College of Obstetricians and Gynaecologists; 1982 September. London: RCOG, 1983:269–80 |
| 1. Gadallah M, Rady M, Salem B, Aly EM, Anwer W. The effect of nutritional intervention program on the prevalence of anemia among pregnant women in rural areas of Belbis district-Sharkia Governorate-Egypt. The Journal of the Egyptian Public Health Association 2002; 77:261–273 |
| 1. Garg A, Kashyap S. Effect of counseling on nutritional status during pregnancy. Indian Journal of Pediatrics 2006; 73:687–692 |
| 1. Garnaes KK, Morkved S, Salvesen O, Moholdt T. Exercise training and weight gain in obese pregnant women: a randomized controlled trial (ETIP Trial) [with consumer summary]. PLoS Med. 2016;13(7):e1002079 |
| 1. Garshasbi, A.; Faghih Zadeh, S. The effect of exercise on the intensity of low back pain in pregnant women. Int. J. Gynaecol. Obstet. 2005, 88, 271–275. |
| 1. Gaston A, Prapavessis H (2009) Maternal-fetal disease information as a source of exercise motivation during pregnancy. Health Psychology 28: 726. |
| 1. Gau, M.L.; Chang, C.Y.; Tian, S.H.; Lin, K.C. Effects of birth ball exercise on pain and self-efficacy during childbirth: A randomised controlled trial in Taiwan. Midwifery 2011, 27, e293–e300 |
| 1. Ghodsi, Z.; Asltoghiri, M. Effects of aerobic exercise training on maternal and neonatal outcome: A randomized controlled trial on pregnant women in Iran. J. Pak. Med. Assoc. 2014, 64, 1053–1056 |
| 1. Goletzke J, Buyken AE, Louie JC, Moses RG, BrandMiller JC. Dietary micronutrient intake during pregnancy is a function of carbohydrate quality. American Journal of Clinical Nutrition 2015;102(3):626–32 |
| 1. Graham AV, Frank SH, Zyzanski SJ, Kitson GC, Reeb KG. A clinical trial to reduce the rate of low birth weight in an inner-city black population. Family Medicine 1992; 24:439–446. |
| 1. Grant SM et al (2011) Effect of a low glycaemic diet on blood glucose in women with gestational hyperglycaemia. Diabetes Res Clin Prac, 91, 15-22. |
| 1. Gray-Donald K, Robinson E, Collier A, David K, Renaud L, Rodrigues S. Intervening to reduce weight gain in pregnancy and gestational diabetes mellitus. CMAJ 2000; 163: 1247–1251. |
| 1. Grivell R, Yelland L, Earl RA, Staehr CJ, Dodd J. The effect of antenatal dietary and lifestyle advice on fetal body composition in women who are overweight or obese: findings from the LIMIT randomised trial. *Ultrasound in Obstetrics and Gynecology* 2013;42(Suppl 1):10. |
| 1. Grivell R, Yelland L, Staehr CJ, Earl RA, Dodd J. The effect of antenatal dietary and lifestyle advice on fetal growth in women who are overweight or obese: findings from the LIMIT randomised trial. *Ultrasound in Obstetrics and Gynecology* 2013;42(Suppl 1):83. |
| 1. Grivell RM, Yelland LN, Deussen A, Crowther CA, Dodd JM. Antenatal dietary and lifestyle advice for women who are overweight or obese and the effect on fetal growth and adiposity: the LIMIT randomised trial. BJOG: an international journal of obstetrics & gynaecology 2016;123 (2):233–43. |
| 1. Grotenfelt NE, Wasenius NS, Rono K, Laivuori H, Stach-Lempinen B, Orho-Melander M, et al. Interaction between rs10830963 polymorphism in mtnr1b and lifestyle intervention on occurrence of gestational diabetes. Diabetologia 2016;59(8):1655–8.? |
| 1. Guelinckx I, Devlieger R, Mullie P, Vansant G. Effect of lifestyle intervention on dietary habits, physical activity, and gestational weight gain in obese pregnant women: a randomized controlled trial. *American Journal of Clinical Nutrition* 2010;91(2):373–80. |
| 1. Gustafsson, M.K.; Stafne, S.N.; Romundstad, P.R.; Mørkved, S.; Salvesen, K.; Helvik, A.S. The effects of an exercise program during pregnancy on health-related quality of life in pregnant women: A Norwegian randomised controlled trial. BJOG 2016, 123, 1152–1160. |
| 1. Haakstad L, Bo K. Effect of supervised aerobic dance exercise in prevention of excessive weight gain in pregnancy: a single blind randomized controlled trial. *International Journal of Gynecology & Obstetrics* 2009;107(Suppl 2):S198. |
| 1. Haakstad L, Bo K. Exercise during pregnancy-Does it impact offspring birth weight parameters?. *Journal of Science and Medicine in Sport* 2012;15(Suppl 1):S340. |
| 1. Haakstad LA, Bo K. Exercise in pregnant women and birth weight: a randomized controlled trial. *BMC Pregnancy and Childbirth 2011;11:66.* |
| 1. Haakstad LAH, Bo K. Effect of regular exercise on prevention of excessive weight gain in pregnancy: A randomised controlled trial. European Journal of Contraception and Reproductive Health Care 2011;16(2):116–25. |
| 1. Haakstad, L.A.; Torset, B.; Bø, K. What is the effect of regular group exercise on maternal psychological outcomes and common pregnancy complaints? An assessor blinded RCT. Midwifery 2016, 32, 81–86 |
| 1. Haakstad, L.A.; Vistad, I.; Sagedal, L.R.; Lohne-Seiler, H.; Torstveit, M.K. How does a lifestyle intervention during pregnancy influence perceived barriers to leisure-time physical activity? The Norwegian fit for delivery study, a randomized controlled trial. BMC Pregnancy Childbirth 2018, 18, 127 |
| 1. Hagobian TA, Phelan S, Gorin AA, Phipps MG, Abrams B, Wing RR. Effects of maternal lifestyle intervention during pregnancy on untreated partner weight: results from fit for delivery study. Obesity 2016;24(1):23–5.?NCT01117961. Promoting healthy weight gain during pregnancy. clinicaltrials.gov/ct2/show/NCT01117961 (first received 15 April 2010).? |
| 1. Hankin ME, Symonds EM. Body weight, diet and pre-eclamptic toxaemia of pregnancy. The Australian & New Zealand Journal of Obstetrics & Gynaecology 1962; 4:156–160 |
| 1. Harrison CL, Lombard CB, Gibson-Helm M, Deeks A, Teede HJ. Limiting excess weight gain in high-risk pregnancies: A randomized controlled trial. *Endocrine Reviews* 2011; 32(3 Meeting Abstracts):P1–466. |
| 1. Harrison CL, Lombard CB, Strauss BJ, Teede HJ. Optimizing healthy gestational weight gain in women at high risk of gestational diabetes: a randomized controlled trial. *Obesity* 2013;21(5):904–9. |
| 1. Harrison CL, Teede HJ, Lombard CB. How effective is self-weighing in the setting of a lifestyle intervention to reduce gestational weight gain and postpartum weight retention?. *Australian & New Zealand Journal of Obstetrics & Gynaecology* 2014;54:382–5. |
| 1. Harrison CL, Lombard CB, Teede HJ. Limiting postpartum weight retention through early antenatal intervention: the HeLPher randomised controlled trial. Int J Behav Nutr Phys Act 2014; 11: 1–8. |
| 1. Hawkins M, Hosker M, Marcus BH, Rosal MC, Braun B, Stanek EJ, et al. A pregnancy lifestyle intervention to prevent gestational diabetes risk factors in overweight Hispanic women: a feasibility randomized controlled trial. *Diabetic Medicine* 2014;32:108–15. |
| 1. Hayes L, Bell R, Robson S, Poston L. Association between physical activity in obese pregnant women and pregnancy outcomes: The UPBEAT Pilot Study. *Annals of Nutrition & Metabolism* 2014;64(3-4):239–46. |
| 1. Hayes L, Bell R, Robson S, Poston L. UPBEAT study: Association between physical activity in obese pregnant women and health of the offspring. The Power of Programming 2014: International Conference on Developmental Origins of Adiposity and Long-Term Health; 2014 March 13-15; Munich, Germany. 2014:48. ISRCTN89971375. UK Pregnancies Better Eating and Activity Trial. isrctn.com/ISRCTN89971375 (first received 23 July 2008). |
| 1. Hayes L, Mcparlin C, Kinnunen TI, Poston L, Robson SC, Bell R. Change in level of physical activity during pregnancy in obese women: findings from the UPBEAT pilot trial. BMC Pregnancy and Childbirth 2015;15:52.? |
| 1. Herring SJ, Cruice JF, Bennett GG, Davey A, Foster GD. Using technology to promote postpartum weight loss in urban, lowincome mothers: a pilot randomized controlled trial. J Nutr Educ Behav 2014; 46: 610–615. |
| 1. Herring SJ, Cruice JF, Bennett GG, Rose MZ, Davey A, Foster GD. Preventing excessive gestational weight gain among African American women: a randomized clinical trial. Obesity 2016; 24: 30–36. |
| 1. Hillesund ER, Bere E, Sagedal LR, Vistad I, Overby NC. Effect of a diet intervention during pregnancy on dietary behavior in the randomized controlled Norwegian Fit for Delivery study. Journal of Developmental Origins of Health and Disease 2016;7(5):538–47.?NCT01001689. Fit for delivery: a study of the effect of exercise sessions and nutritional counselling on pregnancy outcome (FFF). clinicaltrials.gov/ct2/show/NCT01001689 (first received 26 October 2009).? |
| 1. Hoirisch-Clapauch S, Sant’Anna MCW, Moreira ECC, Frankel PP, Valle MP, D’Ippolito MM. A protocol combining daily walking and a low glycemic index diet increases the rate of take-home babies in women with consecutive first-trimester miscarriages. BJOG: an international journal of obstetrics and gynaecology 2016;123 (Suppl 2):74–5. |
| 1. Hoppu U, Isolauri E, Koskinen P, Laitinen K. Diet and blood lipids in 1-4 year-old children. Nutrition Metabolism and Cardiovascular Diseases 2013;23(10):980–6 |
| 1. Hoppu U, Isolauri E, Laakso P, Matomaki J, Laitinen K. Probiotics and dietary counselling targeting maternal dietary fat intake modifies breast milk fatty acids and cytokines. European Journal of Nutrition 2012;51(2):211–9 |
| 1. Horan M, McGowan C, Donnelly J, Gibney E, McAuliffe F. Maternal diet and weight at 3 months partum following a pregnancy intervention with a low glycaemic index diet:Results from the ROLO randomised control trial. *Archives of Disease in Childhood. Fetal and Neonatal Edition* 2014;99 (Suppl 1):A129-A130, Abstract no: PMM.20. |
| 1. Horan MK, McGowan CA, Donnelly J, Gibney E, McAuliffe FM. The association of maternal characteristics and macronutrient intake in pregnancy with neonatal body composition. *Archives of Disease in Childhood. Fetal and Neonatal Edition* 2014;99(Suppl 1):A11. |
| 1. Horan MK, McGowan CA, Doyle O, McAuliffe FM. Well-being in pregnancy: An examination of the effect of socioeconomic, dietary and lifestyle factors including impact of a low glycaemic index dietary intervention. *European Journal of Clinical Nutrition* 2014;68(1):19–24. |
| 1. Horan MK, McGowan CA, Gibney ER, Donnelly JM, McAuliffe FM. Maternal diet and weight at 3 months postpartum following a pregnancy intervention with a low glycaemic index diet: results from the ROLO randomised control trial. *Nutrients* 2014;6(7):2946–55. |
| 1. Horan MK, McGowan CA, Gibney ER, Donnelly JM, McAuliffe FM. Maternal low glycaemic index diet, fat intake and postprandial glucose influences neonatal adiposity - secondary analysis from the ROLO study. *Nutrition Journal* 2014;13(1):78. |
| 1. Horan MK, McGowan CA, Doyle O, McAuliffe FM. Well-being in pregnancy: An examination of the effect of socioeconomic, dietary and lifestyle factors including impact of a low glycaemic index dietary intervention. European Journal of Clinical Nutrition 2014;68(1):19–24 |
| 1. Huang TT, Yeh CY, Tsai YC. A diet and physical activity intervention for preventing weight retention among Taiwanese childbearing women: a randomised controlled trial. *Midwifery* 2011;27(2):257–64. |
| 1. Hui A, Back L, Ludwig S, Gardiner P, Sevenhuysen G, Dean H, et al. Lifestyle intervention on diet and exercise reduced excessive gestational weight gain in pregnant women undera randomised controlled trial. *BJOG: an International Journal of Obstetrics & Gynaecology* 2012;119(1):70–7. |
| 1. Hui AL, Ludwig S, Gardiner P, Sevenhuysen G, Dean HJ, Sellers E, et al. Effects of lifestyle intervention on dietary intake, physical activity level, and gestational weight gain in pregnant women with different pre-pregnancy Body Mass Index in a randomized control trial. *BMC Pregnancy and Childbirth* 2014;14(1):331. |
| 1. Hui AL, Ludwig SM, Gardiner P, Sevenhuysen G, Murray R, Morris M, et al. Community-based exercise and dietary intervention during pregnancy: a pilot study. *Canadian Journal of Diabetes* 2006; 30(2):169–75. |
| 1. Hui A, Back L, Ludwig S, Gardiner P, Sevenhuysen G, Dean H, et al. Exercise and dietary intervention increases physical activity, promotes healthy diet and reduces excessive gestational weight gain in pregnant women: A randomized controlled trial in Urban community. Diabetes 2011;60: A351.? |
| 1. Hui AL, Back L, Reid A, Sevenhuysen G, Ludwig S, Dean H, et al. Effects of physical activity and dietary intakes on weight gain of pregnant women with normal and above normal prepregnancy weight. Canadian Journal of Diabetes 2012;36(5 Suppl 1):S8.? |
| 1. Hui AL, Ludwig S, Gardiner P, Sevenhuysen G, Dean H, Sellers E, et al. Exercise and dietary intervention during pregnancy results in reduced excessive gestational weight gain. Diabetes 2010;59(Suppl 1):A509.? |
| 1. Hunt DJ, Stoecker BJ, Hermann JR, Kopel BL, Williams GS, Claypool PL. Effects of nutrition education programs on anthropometric measurements and pregnancy outcomes of adolescents. Journal of the American Dietetic Association 2002; 102:S100–S102 |
| 1. Hunt IF, Jacob M, Ostegard NJ, Masri G, Clark VA, Coulson AH. Effect of nutrition education on the nutritional status of low-income pregnant women of Mexican descent. The American Journal of Clinical Nutrition 1976; 29:675–684 |
| 1. Jackson RA, Stotland NE, Caughey AB, Gerbert B. Improving diet and exercise in pregnancy with Video Doctor counseling: a randomized trial. *Patient Education and Counseling* 2011;83(2):203–9. |
| 1. Jeffries K, Shub A, Walker SP, Hiscock R, Permezel M. Reducing excessive weight gain in pregnancy: a randomised controlled trial. Medical Journal of Australia 2009;191(8):429–33. |
| 1. Jing W, Huang Y, Liu X, Luo B, Yang Y, Liao S. The effect of a personalized intervention on weight gain and physical activity among pregnant women in China. International Journal of Gynaecology and Obstetrics 2015;129(2):138–41. |
| 1. Kafatos AG, Vlachonikolis IG, Codrington CA. Nutrition during pregnancy: the effects of an educational intervention program in Greece. The American Journal of Clinical Nutrition 1989; 50:970–979 |
| 1. Kannieappan LM, Deussen AR, Moran LJ, Grivell RM, Yelland LN, Dodd JM. The effect of antenatal dietary advice on maternal body composition in women who are overweight or obese - findings from the limit randomised trial. *Journal of Paediatrics 2013;49 Suppl2:94.and Child Health* |
| 1. Kennelly MA, Ainscough K, Lindsay KL, O'Sullivan E, Gibney ER, McCarthy M, et al. Pregnancy exercise and nutrition with smartphone application support: a randomized controlled trial. Obstet Gynecol. 2018;131(5):818–26 |
| 1. Kieffer EC, Welmerink DB, Sinco BR, Welch KB, Rees Clayton EM, Schumann CY, et al. Dietary outcomes in a Spanish-language randomized controlled diabetes prevention trial with pregnant Latinas. *American Journal of Public Health* 2014;104(3):526–33. |
| 1. Kinnunen TI, Pasanen M, Aittasalo M, Fogelholm M, Hilakivi-Clarke L, Weiderpass E, Luoto R. Preventing excessive weight gain during pregnancy: a controlled trial in primary health care. Eur J Clin Nutr 2007; 61: 884–892. |
| 1. Kinnunen TI, Puhkala J, Raitanen J, Ahonen S, Aittasalo M, Virtanen SM, et al. Effects of dietary counselling on food habits and dietary intake of Finnish pregnant women at increased risk for gestational diabetes - a secondary analysis of a cluster-randomized controlled trial. Maternal and Child Nutrition 2014;10(2):184–97.? |
| 1. Kizirian N, Garnett S, Markovic T, Ross G, Louie J, Muirhead R, et al. Effects of a low-glycaemic index diet during pregnancy on offspring body composition: a pilot study. Obesity Research and Clinical Practice 2013;7(Suppl 2):e103 |
| 1. Kizirian N, Garnett S, Markovic T, Ross G, Muirhead R, Brodie S, et al. Maternal diet and infant body composition in women at risk of gestational diabetes mellitus. Obesity Research and Clinical Practice 2014;8(Suppl 1):55 |
| 1. Kluge J, Hall D, Louw Q, et al. Specific exercises to treat pregnancy-related low back pain in a South African population. Int J Gynecol Obstet 2011; 113:187–191. |
| 1. Ko PC, Liang CC, Chang SD, et al. A randomized controlled trial of antenatal pelvic floor exercises to prevent and treat urinary incontinence. Int Urogynecol J 2011; 22:17–22. |
| 1. Koivusalo SB, Rono K, Klemetti MM, Roine RP, Lindstrom J, Erkkola M, et al. Gestational diabetes mellitus can be prevented by lifestyle intervention: The Finnish gestational diabetes prevention study (RADIEL): A randomized controlled trial. Diabetes Care 2016;39:24–30. NCT01698385. Prevention of gestational diabetes through lifestyle modification (RADIEL) - a randomized controlled multi-centre intervention study. clinicaltrials.gov/ct2/show/ NCT01698385 (first received 11 September 2012). |
| 1. Kokic, I.S.; Ivanisevic, M.; Uremovic, M.; Kokic, T.; Pisot, R.; Simunic, B. Effect of therapeutic exercises on pregnancy-related low back pain and pelvic girdle pain: Secondary analysis of a randomized controlled trial. J. Rehabil. Med. 2017, 49, 251–257 |
| 1. Kolu P, Raitanen J, Luoto R. Physical activity and health- related quality of life during pregnancy: a secondary analysis of a cluster-randomised trial. Maternal and Child Health Journal 2014;18(9):2098–105.? |
| 1. Kolu P, Raitanen J, Rissanen P, Luoto R. Cost-effectiveness of lifestyle counselling as primary prevention of gestational diabetes mellitus: findings from a cluster-randomised trial. PLOS One 2013;8(2):e56392. |
| 1. Kolu P, Raitanen J, Rissanen P, Luoto R. Health care costs associated with gestational diabetes mellitus among high-risk women - results from a randomised trial. BMC Pregnancy and Childbirth 2012;12:71.? |
| 1. Kong KL, Campbell G, Foster C, Peterson D, Lanningham-Foster L. A pilot walking program promotes moderate-intensity physical activity during pregnancy. *Medicine & Science in Sports & Exercise* 2014;46(3):462–72. |
| 1. Korpi-Hyovalti E, Schwab U, Laaksonen DE, Linjama H, Heinonen S, Niskanen L. Effect of intensive counselling on and increasing physical activity during pregnancy: A feasibility study. *Diabetes* 2012;61 Suppl 1:A344. |
| 1. Korpi-Hyovalti EA, Laaksonen DE, Schwab US, Vanhapiha TH, Vihla KR, Heinonen ST, et al. Feasibility of a lifestyle intervention in early pregnancy to prevent deterioration of glucose tolerance. BMC Public Health 2011;11:179 |
| 1. Laitinen K, Poussa T, Isolauri E, Nutrition, Allergy, Mucosal Immunology and Intestinal Microbiota Group. Probiotics and dietary counselling contribute to glucose regulation during and after pregnancy: a randomised controlled trial. British Journal of Nutrition 2009;101(11): 1679–87 |
| 1. Laitinen K, Poussa T, Isolauri E. Probiotics and dietary counselling contribute to glucose regulation during and after pregnancy: a randomised controlled trial. The British Journal of Nutrition 2009; 101:1679–1687 |
| 1. Leppanen M, Aittasalo M, Raitanen J, Kinnunen TI, Kujala UM, Luoto R. Physical activity during pregnancy: predictors of change, perceived support and barriers among women at increased risk of gestational diabetes. Maternal and Child Health Journal 2014;18(9):2158–66.? |
| 1. Lombard C, Harrison C, Teede H. A randomized controlled trial investigating self-weighing and the prevention of excess weight gain in early pregnancy. *Endocrine Reviews* 2011;32(3 Meeting Abstracts):P2–768. |
| 1. Long VA, Martin T, Janson-Sand C. The great beginnings program: impact of a nutrition curriculum on nutrition knowledge, diet quality, and birth outcomes in pregnant and parenting teens. Journal of the American Dietetic Association 2002; 102:S86–S89 |
| 1. Louie JCY et al (2011) A randomized controlled trial investigating the effects of a low glycaemic index diet on pregnancy outcomes in gestational diabetes mellitus. Diabetes Care, 34, 2341-6. |
| 1. Luoto R, Kinnunen TI, Aittasalo M, Kolu P, Raitanen J, Ojala K, et al. Primary prevention of gestational diabetes mellitus and large-for-gestational-age newborns by lifestyle counseling: a cluster-randomized controlled trial. PLoS Medicine 2011;8(5):1–11 |
| 1. Luoto R, Laitinen K, Nermes M, Isolauri E. Impact of maternal probiotic-supplemented dietary counseling during pregnancy on colostrum adiponectin concentration: A prospective, randomized, placebo-controlled study. Early Human Development 2012;88(6):339–44 |
| 1. Luoto R, Laitinen K, Nermes M, Isolauri E. Impact of maternal probiotic-supplemented dietary counselling on pregnancy outcome and prenatal and postnatal growth: a double-blind, placebo-controlled study. British Journal of Nutrition 2010;103(12):1792–9 |
| 1. Luoto RM, Kinnunen TI, Aittasalo M, Ojala K, Mansikkamaki K, Poropainen E, et al. Prevention of gestational diabetes: design of a cluster-randomized controlled trial and one-year follow-up. BMC Pregnancy and Childbirth 2010;10:39. |
| 1. Mahony R, Byrne J, Curran S, O’Herlihy C, McAuliffe F. A pilot study of the feasibility of a randomised trial of low glycaemic diet versus normal diet from early pregnancy in euglycaemic women. *Archives of Disease in Childhood. Fetal and Neonatal Edition* 2008;93(Suppl 1):Fa38. |
| 1. Mahony R, Byrne J, Curran S, O’Herlihy C, McAuliffe F. A pilot study of the feasibility of a randomised trial of low glycaemic diet versus normal diet from early pregnancy in euglycaemic women. Archives of Disease in Childhood. Fetal and Neonatal Edition 2008;93(Suppl 1):Fa38 |
| 1. Maitland RA, Barr S, Briley A, Seed P, Poston L. Incidence of gestational diabetes in an obese population using the International Association of Diabetes and Pregnancy Study Groups (IADPSG) criteria in the UK Pregnancies Better Eating and Activity Trial (UPBEAT) pilot study. *Diabetic Medicine* 2012;29(Suppl 1):152. |
| 1. Markovic 2016. A randomized, two-arm parallel dietary intervention study to compare the effects of consuming a low glycemic diet or wholegrain high fibre diet on infant birth weight and body composition, complications related to Gestational Diabetes Mellitus (GDM) and progression to GDM diagnosis in women at high-risk of GDM. anzctr.org.au/Trial/Registration/ TrialReview.aspx?id=335632 Date first received: 18 August 2010 |
| 1. Markovic TP, Muirhead R, Overs S, Kizirian N, Louie J, Sweeting A, et al. Predictors of birthweight in women at high risk of gestational diabetes mellitus. Obesity Research and Clinical Practice 2013;7(2):e3–4 |
| 1. Markovic TP, Muirhead R, Overs S, Ross GP, Louie JC, Kizirian N, et al. Randomized controlled trial investigating the effects of a low-glycemic index diet on pregnancy outcomes in women at high risk of gestational diabetes mellitus: the GI Baby 3 Study. Diabetes Care 2016;39(1): 31–8 |
| 1. Marquez-Sterling, S.; Perry, A.C.; Kaplan, T.A.; Halberstein, R.A.; Signorile, J.F. Physical and psychological changes with vigorous exercise in sedentary primigravidae. Med. Sci. Sports Exerc. 2000, 32, 58–62. [ |
| 1. Mason L, Roe B, Wong H, et al. The role of antenatal pelvic floor muscle exercises in prevention of postpartum stress incontinence: a randomised controlled trial. J Clin Nurs 2010; 19:2777–2786. |
| 1. McAuliffe F. A randomised controlled trial of low glycaemic index carbohydrate diet versus no dietary intervention in the prevention of recurrence of foetal macrosomia. CurrentControlled Trials (www.controlled-trials.com/) (accessed 12.05.2010). |
| 1. McAuliffe F. Maternal nutrition and fetal health. The Power of Programming 2014: International Conference on Developmental Origins of Adiposity and Long-Term Health; 2014 March 13-15; Munich, Germany. 2014 |
| 1. McGowan CA, Walsh JM, Byrne J, Curran S, McAuliffe FM. The influence of a low glycemic index dietary intervention on maternal dietary intake, glycemic index and gestational weight gain during pregnancy: a randomized controlled trial. *Nutrition Journal* 2013;12(1):140. |
| 1. McLaughlin FJ, Altemeier WA, Christensen MJ, Sherrod KB, Dietrich MS, Stern DT. Randomized trial of comprehensive prenatal-care for low-income women – effect on infant birth-weight. Pediatrics 1992; 89:128–132 |
| 1. Melo A. Exercise and pregnancy: randomised clinical trial. Current Controlled Trials (www.controlled-trials.com/)[accessed 31 October 2010]. |
| 1. Miquelutti, M.A.; Cecatti, J.G.; Makuch, M.Y. Evaluation of a birth preparation program on lumbopelvic pain, urinary incontinence, anxiety and exercise: A randomized controlled trial. BMC Pregnancy Childbirth 2013, 13, 154. |
| 1. Montoya Arizabaleta AV, Orozco Buitrago L, Aguilar de Plata AC, et al. Aerobic exercise during pregnancy improves health-related quality of life: a randomised trial. J Physiother 2010; 56:253–258. |
| 1. Morkved S. Effects of regular exercise during pregnancy.ClinicalTrials.gov(www.clinicaltrials.gov) (accessed 31 October 2010). |
| 1. Moses RG, Barker M, Winter M, Petocz P, Brand-Miller JC. Can a low-glycemic index diet reduce the need for insulin in gestational diabetes mellitus? A randomized trial. *Diabetes Care* 2009;32(6):996–1000. |
| 1. Moses RG, Casey S, Cleary J, Milosavljevic M, Quinn E, Tapsell L, et al. Effect of low glycaemic index dietary advice in normal pregnancy: The PREGGIO study. *Obesity Research and Clinical Practice* 2013;7:e34–5. |
| 1. Moses RG, Casey SA, Quinn EG, Cleary JM, Tapsell LC, Milosavljevic M, et al. Pregnancy and Glycemic Index Outcomes study: effects of low glycemic index compared with conventional dietary advice on selected pregnancy outcomes. *American Journal of Clinical Nutrition* 2014;99(3):517–23. |
| 1. Moses RG, Casey SA, Quinn EG, Cleary JM, Tapsell LC, Milosavljevic M, et al. Pregnancy and Glycemic Index Outcomes study: effects of low glycemic index compared with conventional dietary advice on selected pregnancy outcomes. American Journal of Clinical Nutrition 2014;99 (3):517–23 |
| 1. Moses RG, Luebke M, Petocz P, Brand-Miller JC. Maternal diet and infant size 2 y after the completion of a study of a low-glycemic-index diet in pregnancy. American Journal of Clinical Nutrition 2007;86(6):1806 |
| 1. Mujsindi W, Habash D, Childs G. Impact of nutrition education on gestational weight gain in obese pregnant women. *American Journal of Obstetrics and Gynecology* 2014;210(1 Suppl):S188. |
| 1. Murtezani A, Pacarada M, Ibraimi Z, Nevzati A, Abazi N. The impact of exercise during pregnancy on neonatal outcomes: a randomised controlled trial. *Journal of Sports Medicine and Physical Fitness* 2014;54(6):802–8. |
| 1. Nagle C, Skouteris H, Morris h, Nankervis A, Rasmussen B, Mayall P et al. Primary prevention of gestational diabetes for women who are overweight and obese: a randomised controlled trial. BMC Pregnancy Childbirth 2013; 13: 65. |
| 1. Nascimento KLK, Surita SLN, Parpinelli FGS, Kasawara MAP. Type of delivery and neonatal outcome in overweight and obese pregnant women with excessive weight gain. *Journal of Maternal-Fetal and Neonatal Medicine* 2012;25(S2):73–4. |
| 1. Nascimento SL, Surita FG, Parpinelli MA, Siani S, Pintoe Silva JL. The effect of an antenatal physical exercise programme on maternal/perinatal outcomes and quality of life in overweight and obese pregnant women: a randomised clinical trial. *BJOG: an International Journal of Obstetrics & Gynaecology* 2011;118(12):1455–63. |
| 1. Ndiaye M, Siekmans K, Haddad S, Receveur O. Impact of a positive deviance approach to improve the effectiveness of an iron-supplementation program to control nutritional anemia among rural Senegalese pregnant women. Food and Nutrition Bulletin 2009; 30:128–136 |
| 1. Newman AK, Deussen AR, Moran LJ, Grivell RM, Yelland LN, Turnbull D, et al. The effect of antenatal dietary and lifestyle advice on maternal psychological health in women who are overweight or obese-findings from the limit randomised trial. *Journal of Paediatrics and Child Health* 2013;49 Suppl 2:119. |
| 1. Niinivirta K, Isolauri E, Laakso P, Linderborg K, Laitinen K. Dietary counseling to improve fat quality during pregnancy alters maternal fat intake and infant essential fatty acid status. Journal of Nutrition 2011;141(7):1281–5 |
| 1. Niinivirta K, Laakso P, Linderborg K, Poussa T, Isolauri E, Laitinen K. Maternal dietary counseling during pregnancy and infant fatty acid profiles. International Journal of Food Sciences and Nutrition 2014;65(3):268–72 |
| 1. Nilsson K. Weight gain during pregnancy - a randomized controlled trial of intervention to prevent excessive gestational weight gain. ClinicalTrials.gov (http://clinicaltrials.gov/) [accessed 5 September 2014] 2009. |
| 1. Olds DL, Henderson CR, Tatelbaum R, Chamberlin R. Improving the delivery of prenatal-care and outcomes of pregnancy – a randomized trial of nurse home visitation. Pediatrics 1986; 77:16–28 |
| 1. Olson CM, Strawderman MS, Reed RG. Efficacy of an intervention to prevent excessive gestational weight gain. Am J Obstet Gynecol 2004; 191: 530–536. |
| 1. Ong M, Guelfi K, Hunter T, Wallman K, Fournier P, et al. (2009) Supervised home-based exercise may attenuate the decline of glucose tolerance in obese pregnant women. Diabetes Metab 35: 418–421. |
| 1. Oostdam N, Bosmans J, Wouters MGAJ, Eekhoff EMW, van Mechelen W, van Poppel MNM. Cost-effectiveness of an exercise program during pregnancy to prevent gestational diabetes: Results of an economic evaluation alongside a randomised controlled trial. *BMC Pregnancy and Childbirth* 2012;12:64. |
| 1. Oostdam N, Van Poppel MN, Eekhoff EM, Wouters MG, Van Mechelen W. Design of FitFor2 study: the effects of an exercise program on insulin sensitivity and plasma glucose levels in pregnant women at high risk for gestational diabetes. *BMC Pregnancy and Childbirth* 2009;9:1. |
| 1. Oostdam N, Van Poppel MNM, Wouters MGAJ, Eekhoff EMW, Bekedam DJ, Kuchenbecker WKH, et al. No effect of the FitFor2 exercise programme on blood glucose, insulin sensitivity, and birthweight in pregnant women who were overweight and at risk for gestational diabetes: Results of a randomised controlled trial. *BJOG: an International Journal of Obstetrics and Gynaecology* 2012;119(9):1098–107. |
| 1. Ozdemir, S.; Bebis, H.; Ortabag, T.; Acikel, C. Evaluation of the efficacy of an exercise program for pregnant women with low back and pelvic pain: A prospective randomized controlled trial. J. Adv. Nurs. 2015, 71, 1926–1939 |
| 1. Patel N, Godfrey KM, Pasupathy D, Levin J, Flynn AC, Hayes L, et al. Infant adiposity following a randomised controlled trial of a behavioural intervention in obese pregnancy. International Journal of Obesity 2017 Mar 21 [Epub ahead of print]. [DOI: 10.1038/ijo.2017.44 |
| 1. Patel NR, Pasupathy D, Flynn AC, Hayes L, Levin JG, Singh C, et al. The UPBEAT behavioural intervention in obese pregnant women - maternal and infant follow-up 6 months postpartum. Reproductive Sciences 2016;23(Suppl 1):71A. |
| 1. Pawlak DB. Glycemic load and infant birth weight in pregnant overweight/obese women. ClinicalTrials.gov (http://clinicaltrials.gov/) (accessed 31 October 2010). |
| 1. Perales, M.; Refoyo, I.; Coteron, J.; Bacchi, M.; Barakat, R. Exercise during pregnancy attenuates prenatal depression: A randomized controlled trial. Eval. Health Prof. 2015, 38, 59–72 |
| 1. Pérez-Ferre N, Galindo M, Fernández MD, Velasco V, Runkle I, de la Cruz MJ, et al. The outcomes of gestational diabetes mellitus after a telecare approach are not inferior to traditional outpatient clinic visits. Int J Endocrinol 2010;2010:386941 [FREE Full text] [doi: 10.1155/2010/386941] [Medline: 20628517] |
| 1. Petrella E, Facchinetti F, Bertarini V, Pignatti L, Neri I, Battistini NC. Occurrence of pregnancy complications in women with BMI > 25 submitted to a healthy lifestyle and eating habits program. *American Journal of Obstetrics and Gynecology* 2013;208(1 Suppl):S33–4. |
| 1. Petrella E, Malavolti M, Bertarini V, Pignatti L, Neri I, Battistini NC, et al. Gestational weight gain in overweight and obese women enrolled in a healthy lifestyle and eating habits program. *Journal of Maternal-Fetal and Neonatal Medicine* 2014;27(13):1348–52. |
| 1. Petrella E, Facchinetti F, Bertarini V, Pignatti L, Neri I, Battistini NC. Occurrence of pregnancy complications in women with BMI > 25 submitted to a healthy lifestyle and eating habits program. American Journal of Obstetrics and Gynecology 2013;208(1 Suppl):S33–4.? |
| 1. Petrella E, Malavolti M, Bertarini V, Pignatti L, Neri I, Battistini NC, et al. Gestational weight gain in overweight and obese women enrolled in a healthy lifestyle and eating habits program. Journal of Maternal-Fetal and Neonatal Medicine 2014;27(13):1348–52. |
| 1. Petrov Fieril K, Glantz A, Fagevik Olsen M. The efficacy of moderate-to-vigorous resistance exercise during pregnancy: A randomized controlled trial. Acta Obstetricia et Gynecologica Scandinavica 2014 Oct 7 [Epub ahead of print]. |
| 1. Phelan S, Phipps MG, Abrams B, Darroch F, Grantham K, Schaffner A, et al. Does behavioral intervention in pregnancy reduce postpartum weight retention? Twelve-month outcomes of the Fit for Delivery randomized trial. *American Journal of Clinical Nutrition* 2014;99(2):302–11. |
| 1. Phelan S, Phipps MG, Abrams B, Darroch F, Schaffner A, Wing RR. Factors associated with success in the “fit for delivery” intervention to reduce excessive gestational weight gain. *Obesity* 2011;19(Suppl 1):S95. |
| 1. Phelan S, Phipps MG, Abrams B, Darroch F, Schaffner A, Wing RR. Randomized trial of a behavioral intervention to prevent excessive gestational weight gain: the Fit for Delivery Study. *American Journal of Clinical Nutrition* 2011;93(4):772–9. |
| 1. Piirainen T, Isolauri E, Lagstrom H, Laitinen K. Impact of dietary counselling on nutrient intake during pregnancy: a prospective cohort study. The British Journal of Nutrition 2006; 96:1095–1104 |
| 1. Pinzon DC, Zamora K, Martinez JH, Florez-Lopez ME, de Plata AC, Mosquera M, et al. Type of delivery and |
| 1. Pollak KI, Alexander SC, Bennett G, Lyna P, Coffman CJ, Bilheimer A, et al. Weight-related SMS texts promoting appropriate pregnancy weight gain: A pilot study. *Patient Education and Counselling* 2014;97:256–60. |
| 1. Polley BA, Wing RR, Sims CJ. Randomized controlled trial to prevent excessive weight gain in pregnant women. *International Journal of Obesity & Related Metabolic Disorders: Journal of the International Association for the Study of Obesity* 2002;26(11):1494–502. |
| 1. Poston L, Briley AL, Barr S, Bell R, Croker H, Coxon K, et al. Developing a complex intervention for diet andactivity behaviour change in obese pregnant women (the UPBEAT trial); assessment of behavioural change and process evaluation in a pilot randomised controlled trial. *BMC Pregnancy and Childbirth* 2013;13(1):148. |
| 1. Poston L, Bell R, Croker H et al. Effect of a behavioural intervention in obese pregnant women (the UPBEAT study): a multicentre, randomised controlled trial. Lancet Diabetes Endocrinol 2015; 3: 767–777. |
| 1. Poston L, Holmes B, Kinnunen T, Croker H, Bell R, Sanders T, et al. A complex intervention to improve outcome in obese pregnancies; the upbeat study. Archives of Disease in Childhood: Fetal and Neonatal Edition 2011;96 (Suppl 1):Fa97.? |
| 1. Price B, Amini B, Kappeler K. Exercise in pregnancy: Effect on fitness and obstetric outcomes - a randomized trial. *Medicine & Science in Sports & Exercise* 2012;44(12):2263–9. |
| 1. Puhkala J, Luoto R, Ahotupa M, Raitanen J, Vasankari T. Postpartum weight retention is associated with elevated ratio of oxidized LDL lipids to HDL-cholesterol. Lipids 2013;48(12):1227–35. |
| 1. Quinlivan JA, Lam LT, Fisher J. A randomised trial of a four-step multidisciplinary approach to the antenatal care of obese pregnant women. *Australian and New Zealand Journal of Obstetrics and Gynaecology* 2011;51:141–6. |
| 1. Quinlivan J. A randomised trial of a multidisciplinary teamcare approach involving obstetric, dietary and clinical psychological input in obese pregnant women to reduce the incidence of gestational diabetes. https:// www.anzctr.org.au/Trial/Registration/TrialReview.aspx?id= 821 (accessed 2 November 2015) |
| 1. Rae A, Bond D, Evans S, North F, Roberman B, Walters B. A randomised controlled trial of dietary energy restriction in the management of obese women with gestational diabetes. *Australian & New Zealand Journal of Obstetrics & Gynaecology* 2000;40 (4):416–22. |
| 1. Ramirez-Velez R, Aguilar de Plata AC, Escudero MM, Echeverry I, Ortega JG, Salazar B, et al. Influence of regular aerobic exercise on endothelium-dependent vasodilation and cardiorespiratory fitness in pregnant women. *Journal of Obstetrics and Gynaecology Research* 2011;37(11):1601–8. |
| 1. Ramirez-Velez R. Aerobic exercise in pregnant Latina women: Effect on metabolic and body composition outcomes. A randomized clinical trial. *FASEB Journal* 2014;28(1 Suppl 1):886.6. |
| 1. Ramirez-Velez R. Combined aerobic and resistance exercise on metabolic and body composition outcomes in primigravid Latina women. *Obesity Reviews* 2014;15(Suppl2):201–2. |
| 1. Ramırez-Velez R, Aguilar de Plata AC, Escudero MM, et al. Influence of regular aerobic exercise on endothelium-dependent vasodilation and cardiorespiratory fitness in pregnant women. J Obstet Gynaecol Res 2011; 37:1601– 1608. |
| 1. Rankin J (2002) Effects of antenatal exercise on psychological well-being.pregnancy and birth outcome. |
| 1. Rauh K, Gabriel E, Kerschbaum E, Schuster T, von Kries R, Amann-Gassner U, et al. Safety and efficacy of a lifestyle intervention for pregnant women to prevent excessive maternal weight gain: a cluster-randomized controlled trial. *BMC Pregnancy and Childbirth* 2013;13(1):151. |
| 1. Rauh K, Gunther J, Kunath J, Stecher L, Hauner H. Lifestyle intervention to prevent excessive maternal weight gain: mother and infant follow-up at 12 months postpartum. BMC Pregnancy and Childbirth 2015;15:265. |
| 1. Renault KM, Norgaard K, Nilas L, Carlsen EM, Cortes D, Pryds O, et al. The Treatment of Obese Pregnant Women (TOP) study: A randomized controlled trial of the effect of physical activity intervention assessed by pedometer with or without dietary intervention in obese pregnant women. *American Journal of Obstetrics and Gynecology* 2014;210(2):134.e1–9. |
| 1. Rhodes ET, Pawlak DB, Takoudes TC, Ebbeling CB, Feldman HA, Lovesky MM, et al. Effects of a low-glycemic load diet in overweight and obese pregnant women: a pilot randomized controlled trial. *American Journal of Clinical Nutrition* 2010;92(6):1306–15. |
| 1. Robledo-Colonia AF, Sandoval-Restrepo N, Mosquera-Valderrama YF, et al. Aerobic exercise training during pregnancy reduces depressive symptoms in nulliparous women: a randomized clinical trial. J Physiother 2012; 58:9–15. |
| 1. Ronnberg A, Ostlund I, Fadl H, Gottvall T, Nilsson K. Intervention during pregnancy to reduce excessive gestational weight gain-a randomised controlled trial. BJOG:an International Journal of Obstetrics and Gynaecology 2014 Nov 4 [Epub ahead of print]. |
| 1. Rono K, Stach-Lempinen B, Klemetti MM, Kaaja RJ, Poyhonen-Alho M, Eriksson JG, et al. Prevention of gestational diabetes through lifestyle intervention: study design and methods of a Finnish randomized controlled multicenter trial (RADIEL). BMC Pregnancy and Childbirth 2014;14:70. |
| 1. Ruchat SM, Davenport MH, Giroux I, Hillier M, Batada A, Sopper MM, et al. Nutrition and exercise reduce excessive weight gain in normal-weight pregnant women. *Medicine and Science in Sports and Exercise* 2012;44(8):1419–26. |
| 1. Ruiz R, Perales M, Pelaez M, Lopez C, Lucia A, Barakat R. Supervised exercise-based intervention to prevent excessive gestational weight gain: a randomized controlled trial. *Mayo Clinic Proceedings* 2013;88(12):1388–97. |
| 1. Sachdeva R, Mann SK. Impact of nutrition counselling and supplements on the mineral nutriture of rural pregnant women and their neonates. Indian Pediatrics 1994; 31:643–649 |
| 1. Sachdeva R, Mann SK. Impact of nutrition education and medical supervision on pregnancy outcome. Indian Pediatrics 1993; 30:1309–1314 |
| 1. Sagedal LR, Henriksen T, Overby NC, Lohne-Seiler?H, Torstveit MK, Bere E, et al. The problem of non- participation: Who declined to participate in ”Fit for Delivery“, a randomized, controlled trial of a lifestyle intervention in pregnancy?. The Power of Programming 2014: International Conference on Developmental Origins of Adiposity and Long-Term Health; 2014 March 13-15; Munich, Germany. 2014:63.? |
| 1. Sagedal LR, Overby N, Lohne-Seiler H, Bere E, Torstveit M, Henriksen T, et al. Study protocol: Fit for Delivery - can a lifestyle intervention in pregnancy result in measurable health benefits for mothers and newborns? A randomized controlled trial. BMC Public Health 2013;13:132.? |
| 1. Sagedal LR, Overby NC, Bere E, Torstveit MK, Lohne- Seiler H, Smastuen M, et al. Lifestyle intervention to limit gestational weight gain: the Norwegian Fit for Delivery randomised controlled trial. BJOG: an international journal of obstetrics and gynaecology 2017;124(1):97–109.? |
| 1. Sagedal LR, Sanda B, Overby NC, Bere E, Torstveit MK, Lohne-Seiler H, et al. The effect of prenatal lifestyle intervention on weight retention 12 months postpartum: results of the Norwegian Fit for Delivery randomised controlled trial. BJOG: an international journal of obstetrics and gynaecology 2017;124(1):111–21. |
| 1. Sagedal LR. Who are we missing? Examining non- participants in ”Fit for Delivery“, a randomized, controlled trial of a lifestyle intervention in pregnancy. Pregnancy Hypertension 2014;4(3):237.? |
| 1. Santaella MP. The role of a supervised physical exercise program as an alternative on the control of maternal estational weight gain. ClinicalTrials.gov (accessed 21 May20 13). NCT01790347 2013. |
| 1. Santos IA, Stein R, Fuchs SC, Duncan BB, Ribeiro JP, Kroeff LR, et al. Aerobic exercise and submaximal functional capacity in overweight pregnant women: a randomized trial. *Obstetrics & Gynecology* 2005;106(2):243–9. |
| 1. Schneeberger C, Flynn A, Barr S, Seed PT, Inskip HM, Poston L. Maternal diet patterns and glycaemic load in obese pregnant women taking part in a pilot trial of a lifestyle intervention (the upbeat trial). Diabetes 2014; Vol.63. |
| 1. Seed PT, Briley A, Singh C, Patel N, Poston L. A novel method for devising optimal gestational weight gain in obese pregnant women. Reproductive Sciences 2016; Vol. 23, issue 1 Suppl 1:276A.? |
| 1. Senanayake HM, Premaratne SP, Palihawadana T, Wijeratne S. Simple educational intervention will improve the efficacy of routine antenatal iron supplementation. The Journal of Obstetrics and Gynaecology Research 2010; 36:646–650 |
| 1. Seneviratne S, Jiang Y, Derraik J, McCowan L, Parry G, Biggs J, et al. Effects of antenatal exercise in overweight and obese pregnant women on maternal and perinatal outcomes: a randomised controlled trial. BJOG. 2016; 123(4):588–97. |
| 1. Shen GX (2006) Community-based exercise and dietary intervention during pregnancy: A pilot study. Canadian Journal of diabetes 30: 169–175. |
| 1. Shirazian T, Monteith S, Friedman F, Rebarber A. Lifestyle modification program decreases pregnancy weight gain in obese women. Am J Perinat 2010; 27: 411–414 |
| 1. Shub A. Diet and exercise in pregnancy. Australian Clinical Trials Registry (www.actr.org.au/) [accessed 21 June 2007]. |
| 1. Smith K, Lanningham-Foster L, Welch A, Campbell C. Web-based behavioral intervention increases maternal exercise but does not prevent excessive gestational weight gain in previously sedentary women. J Phys Act Health 2016 Jun;13(6):587-593. [doi: 10.1123/jpah.2015-0219] [Medline: 26594820] |
| 1. Smith KM. The Blossom Project Online: use of a behaviorallybased website to promote physical activity and prevent excessive gestational weight gain in previously sedentary pregnant women. Graduate Theses and Dissertations 2014: 14051 |
| 1. Smoke J, Grace MC. Effectiveness of prenatal care and education for pregnant adolescents: nurse-midwifery intervention and team approach. Journal of Nurse-Midwifery 1988; 33:178–184 |
| 1. Soltani H, Duxbury AM, Arden MA, Dearden A, Furness PJ, Garland C. Maternal obesity management using mobile technology: a feasibility study to evaluate a text messaging based complex intervention during pregnancy. J Obes 2015;2015:814830 [FREE Full text] [doi: 10.1155/2015/814830] [Medline: 25960889] |
| 1. Songøygard KM, Stafne SN, Evensen KA, et al. Does exercise during pregnancy prevent postnatal depression? A randomized controlled trial. Acta Obstet Gynecol Scand 2012; 91:62–67. |
| 1. Songoygard, K.M.; Stafne, S.N.; Evensen, K.A.; Salvesen, K.A.; Vik, T.; Morkved, S. Does exercise during pregnancy prevent postnatal depression? A randomized controlled trial. Acta Obstet. Gynecol. Scand. 2012, 91, 62–67 |
| 1. *S*tafne SN, Salvesen KÅ, Romundstad PR, Eggebø TM, Carlsen SM, Mørkved S. Regular exercise during pregnancy to prevent gestational diabetes: a randomized controlled trial. *Obstetrics & Gynecology* 2012;119(1):29–36. |
| 1. Stafne SN, Salvesen KA, Romundstad PR, et al. Does regular exercise influence lumbopelvic pain? A randomized controlled trial. Acta Obstet Gynecol Scand 2012; 91:552–559. |
| 1. Sui Z, Yelland LN, Turnbull D, Dodd JM. Walking to limit gestational weight gain and keep fit during pregnancy - findings from the walk randomised trial. *Journal of Paediatrics and Child Health* 2013;49 Suppl 2:120. |
| 1. Sultemeier A. An innovative approach to teaching prenatal nutrition. Journal of Community Health Nursing 1988; 5:247–254 |
| 1. Sun JD, Shao YF, Zhang PL, Li DZ, Gu LY, Guo QN. Evaluation of prenatal nutrition counseling: maternal nutrition status and infant birthweight. Biomedical and Environmental Sciences 1990; 3:458–465. 32 |
| 1. Suputtitada, A.; Wacharapreechanont, T.; Chaisayan, P. Effect of the “sitting pelvic tilt exercise” during the third trimester in primigravidas on back pain. J. Med. Assoc. Thail. 2002, 85 (Suppl. 1), S170–S179. |
| 1. Surita F. Physical exercise influence among overweightand obese pregnant women. ClinicalTrials.gov (http://clinicaltrials.gov/) [accessed 21 May 2013] 2010. |
| 1. Szmeja MA, Cramp C, Grivell RM, Deussen AR, Yelland LN, Dodd JM. Use of a DVD to provide dietary and lifestyle information to pregnant women who are overweight or obese: a nested randomised trial. BMC Pregnancy and Childbirth 2014;14(1):409.? |
| 1. Tanvig M, Vinter CA, Jorgensen JS, Wehberg S, Ovesen PG, Beck-Nielsen H, et al. Effects of lifestyle intervention in pregnancy and anthropometrics at birth on offspring metabolic profile at 2.8 years - results from the Lifestyle in Pregnancy and Offspring (LiPO) study. *Journal of Clinical Endocrinology and Metabolism* 2015;100(1):175–83. |
| 1. Tanvig M. Offspring body size and metabolic profile - Effects of lifestyle intervention in obese pregnant women. *Danish Medical Journal* 2014;61(7):B4893. |
| 1. Tanvig M, Vinte CA, Jorgensen JS, Wehberg S, Ovesen PG, Lamont RF, et al. Anthropometrics and body composition by dual energy X-ray in children of obese women: a follow-up of a randomized controlled trial (the Lifestyle in Pregnancy and Offspring [LiPO] study). PLOS One 2014;9 (2):e89590. |
| 1. Teede HJ, Harrison CL, Gibson-Helm M, Lombard CB. Improving physical activity in high-risk pregnancies: A randomized controlled trial. *Endocrine Reviews* 2011;32(3Meeting Abstracts):P1–467. |
| 1. Thornton YS, Smarkola C, Kopacz SM, Ishoof SB. Perinatal outcomes in nutritionally monitored obese pregnant women: a randomized clinical trial. *Journal of the National Medical Association* 2009;101:569–77. |
| 1. Umpierrez G. Lifestyle intervention to limit excessive weight gain during pregnancy in minority women. ClinicalTrials.gov (accessed 21 May 2013). NCT01084941 2010. |
| 1. Vahamiko S, Isolauri E, Laitinen K. Weight status and dietary intake determine serum leptin concentrations in pregnant and lactating women and their infants. British Journal of Nutrition 2013;110(6):1098–106 |
| 1. Valkama A, Koivusalo S, Lindstrom J, Meinila J, Kautiainen H, Stach-Lempinen B, et al. The effect of dietary counselling on diet in pregnant women at risk for gestational diabetes. Annals of Nutrition and Metabolism 2015;67 (Suppl 1):138. |
| 1. Valkama A, Koivusalo S, Lindstrom J, Meinila J, Kautiainen H, Stach-Lempinen B, et al. The effect of dietary counselling on food intakes in pregnant women at risk for gestational diabetes: a secondary analysis of a randomised controlled trial RADIEL. European Journal of Clinical Nutrition 2016;70(8):912–7. |
| 1. Vallim AL, Osis MJ, Cecatti JG, et al. Water exercises and quality of life during pregnancy. Reprod Health 2011; 8:14. |
| 1. Van Poppel M, Oostdam N, Wouters M, Eekhoff M, Van Mechelen W. FitFor2: Effects of an exercise training program on the incidence of gestational diabetes. *Journal of Science and Medicine in Sport* 2012;15 Suppl 1:S342–S343. |
| 1. Van Horn L, Peaceman A, Kwasny M, Vincent E, Fought A, Josefson J, et al. Dietary approaches to stop hypertension diet and activity to limit gestational weight: maternal offspring Metabolics family intervention trial, a technology enhanced randomized trial. Am J Prev Med. 2018;55(5):603–14 |
| 1. van Zutphen M, Milder IE, Bemelmans WJ. Usage of an online healthy lifestyle program by pregnant women attending midwifery practices in Amsterdam. Prev Med 2008; 46: 552–557. |
| 1. Vesco K, Leo M, Gillman M, King J, McEvoy C, Karanjaa N, et al. Impact of a weight management intervention on pregnancy outcomes among obese women: The Healthy Moms Trial. *American Journal of Obstetrics and Gynecology* 2013;208(1 Suppl 1):S352. |
| 1. Vesco KK, Karanja N, King JC, Gillman MW, Leo C, Perrin N, et al. Efficacy of a group-based dietary intervention for limiting gestational weight gain among obese women: A randomized trial. *Obesity (Silver Spring, Md.)*2014;2(9):1989–96. |
| 1. Vesco KK, Karanja N, King JC, Gillman MW, Perrin N, McEvoy C, et al. Healthy Moms, a randomized trial to promote and evaluate weight maintenance among obese pregnant women: study design and rationale. *Contemporary Clinical Trials* 2012;33(4):777–85. |
| 1. Vieira MC, Pasupathy D, Patel NR, White SL, Briley A, Seed PT, et al. Factors associated with uncomplicated pregnancy in obese women from the UPBEAT trial. Reproductive Sciences 2016;23(Suppl 1):109A.?White L, Pasupathy D, Vieira MC, Briley AL, Seed P, Lawlor DA, et al. Prediction of gestational diabetes (GDM) in obese women. Reproductive Sciences 2016;23(Suppl 1): 124A. |
| 1. Vinter C, Jensen D, Ovesen P, Beck-Nielsen H, Lamont R, Jorgensen J. Postpartum weight retention and breastfeeding among obese women from the LiP (Lifestyle in Pregnancy) Study. *Acta Obstetricia et Gynecologica Scandinavica* 2012;91(Suppl 159):141–2. |
| 1. Vinter CA, Jensen DM, Ovesen P, Beck-Nielsen H, Tanvig M, Lamont RF, et al. Postpartum weight retention and breastfeeding among obese women from the randomized controlled Lifestyle in Pregnancy (LiP) trial. *Acta Obstetricia et Gynecologica Scandinavica* 2014;93:794–801. |
| 1. Vinter CA, Jensen DM, Ovesen P, Beck-Nielsen H, Jorgensen JS. The LiP (Lifestyle in Pregnancy) study: a randomized controlled trial of lifestyle intervention in 360 obese pregnant women. *Diabetes Care* 2011;34(12):2502–7. |
| 1. Vinter CA, Jensen DM, Ovesen PG, Beck-Nielsen H, Jorgensen JS. Lifestyle and pregnancy (LIP) study: The clinical effect of lifestyle intervention during pregnancy in obese women. Diabetes 2011;60:A348–9. |
| 1. Vinter CA, Jørgensen JS, Ovesen P, Beck-Nielsen H, Skytthe A, Jensen DM. Metabolic effects of lifestyle intervention in obese pregnant women. results from the randomized controlled trial ’lifestyle in pregnancy’ (LiP). Diabetic Medicine 2014;31(11):1323–30. |
| 1. Vitolo MR, Fraga Bueno MS, Mendes Gama C. Impact of a dietary counseling program on the gain weight speed of pregnant women attended in a primary care service. *Revista Brasileira de Ginecologia e Obstetricia* 2011;33(1):13–9. |
| 1. Walsh J, Mahony R, Foley M, Mc Auliffe F. A randomised control trial of low glycaemic index carbohydrate diet versus no dietary intervention in the prevention of recurrence of macrosomia. *BMC Pregnancy and Childbirth*2010;10:16. |
| 1. Walsh J, Mahony R, Foley M, McAuliffe F. ROLO study: a randomized control trial of low glycemic index diet to prevent macrosomia in euglycemic women. *American Journal of Obstetrics and Gynecology* 2012;206,Suppl 1):S4. |
| 1. Walsh J, McGowan C, Byrne J, Foley M, Mahony R, McAuliffe F. The influence of a low glycaemic index dietary intervention on maternal glycaemic index, dietary intake and gestational weight gain. *American Journal of Obstetrics and Gynecology* 2013;208(1 Suppl):S33. |
| 1. Walsh JM, Mahony RM, Canty G, Foley ME, McAuliffe FM. Identification of those most likely to benefit from a low-glycaemic index dietary intervention in pregnancy. *British Journal of Nutrition* 2014;112:583–9. |
| 1. Walsh JM, McGowan CA, Mahony R, Foley ME, McAuliffe FM. Low glycaemic index diet in pregnancy to prevent macrosomia (ROLO study): randomised control trial. *BMJ* 2012;345:e5605. |
| 1. Walsh JM, Mahony RM, Culliton M, Foley ME, McAuliffe FM. Impact of a low glycemic index diet in pregnancy on markers of maternal and fetal metabolism and inflammation. Reproductive Sciences 2014;21(11):1378–81 |
| 1. Walsh JM, McAuliffe FM. Impact of maternal nutrition on pregnancy outcome - Does it matter what pregnant women eat? Best Practice and Research. Clinical Obstetrics and Gynaecology 2015;29(1):63–78 |
| 1. Wang S, Ma JM, Yang HX. Lifestyle intervention for gestational diabetes mellitus prevention: A cluster- randomized controlled study. Chronic Diseases and Translational Medicine 2015;1(3):169–74. |
| 1. Widga AC, Lewis NM. Defined, in-home, prenatal nutrition intervention for low-income women. Journal of the American Dietetic Association 1999; 99:1058–1062; quiz 1063–1054, 1175 |
| 1. Wilkinson SA, McIntyre HD. Evaluation of the ’healthy start to pregnancy’ early antenatal health promotion workshop: a randomized controlled trial. *BMC Pregnancy and Childbirth* 2012;12:131. |
| 1. Wolff S, Legarth J, Vangsgaard K, Toubro S, Astrup A. A randomized trial of the effects of dietary counseling on gestational weight gain and glucose metabolism in obese pregnant women. *International Journal of Obesity* 2008;32(3):495–501 |
| 1. Yeo S (2009) Adherence to walking or stretching, and risk of preeclampsia in sedentary pregnant women. Res Nurs Health 32: 379–390. |

**S7 References:**

1. Gilinsky A, Swanson V, Power K. Interventions delivered during antenatal care to reduce alcohol consumption during pregnancy: A systematic review. Addiction Research & Theory. 2011;19(3):235-50. doi: 10.3109/16066359.2010.507894.

2. Gebara CF, Bhona FM, Ronzani TM, Lourenco LM, Noto AR. Brief intervention and decrease of alcohol consumption among women: a systematic review. Substance abuse treatment, prevention, and policy. 2013;8:31. Epub 2013/09/11. doi: 10.1186/1747-597x-8-31. PubMed PMID: 24016074; PubMed Central PMCID: PMCPMC3847063.

3. Stade BC, Bailey C, Dzendoletas D, Sgro M, Dowswell T, Bennett D. Psychological and/or educational interventions for reducing alcohol consumption in pregnant women and women planning pregnancy. The Cochrane database of systematic reviews. 2009;(2):Cd004228. Epub 2009/04/17. doi: 10.1002/14651858.CD004228.pub2. PubMed PMID: 19370597; PubMed Central PMCID: PMCPMC4164939.

4. Lui S, Terplan M, Smith EJ. Psychosocial interventions for women enrolled in alcohol treatment during pregnancy. The Cochrane database of systematic reviews. 2008;(3):Cd006753. Epub 2008/07/23. doi: 10.1002/14651858.CD006753.pub2. PubMed PMID: 18646166.

5. Agboola S, McNeill A, Coleman T, Leonardi Bee J. A systematic review of the effectiveness of smoking relapse prevention interventions for abstinent smokers. Addiction (Abingdon, England). 2010;105(8):1362-80. Epub 2010/07/27. doi: 10.1111/j.1360-0443.2010.02996.x. PubMed PMID: 20653619.

6. Chamberlain C, O'Mara-Eves A, Oliver S, Caird JR, Perlen SM, Eades SJ, et al. Psychosocial interventions for supporting women to stop smoking in pregnancy. The Cochrane database of systematic reviews. 2013;(10):Cd001055. Epub 2013/10/25. doi: 10.1002/14651858.CD001055.pub4. PubMed PMID: 24154953; PubMed Central PMCID: PMCPMC4022453.

7. Filion KB, Abenhaim HA, Mottillo S, Joseph L, Gervais A, O'Loughlin J, et al. The effect of smoking cessation counselling in pregnant women: a meta-analysis of randomised controlled trials. BJOG : an international journal of obstetrics and gynaecology. 2011;118(12):1422-8. Epub 2011/09/02. doi: 10.1111/j.1471-0528.2011.03065.x. PubMed PMID: 21880109.

8. Hemsing N, Greaves L, O'Leary R, Chan K, Okoli C. Partner support for smoking cessation during pregnancy: a systematic review. Nicotine & tobacco research : official journal of the Society for Research on Nicotine and Tobacco. 2012;14(7):767-76. Epub 2011/12/20. doi: 10.1093/ntr/ntr278. PubMed PMID: 22180588.

9. Hettema JE, Hendricks PS. Motivational interviewing for smoking cessation: a meta-analytic review. Journal of consulting and clinical psychology. 2010;78(6):868-84. Epub 2010/12/01. doi: 10.1037/a0021498. PubMed PMID: 21114344.

10. Kintz T, Pryor C, Shemami H, Kridli SA-O. Nursing interventions to promote smoking cessation during pregnancy: An integrative review Journal of Nursing Education and Practice. 2014;4(9).

11. Naughton F, Prevost AT, Sutton S. Self-help smoking cessation interventions in pregnancy: a systematic review and meta-analysis. Addiction (Abingdon, England). 2008;103(4):566-79. Epub 2008/03/15. doi: 10.1111/j.1360-0443.2008.02140.x. PubMed PMID: 18339103.

12. Su A, Buttenheim AM. Maintenance of smoking cessation in the postpartum period: which interventions work best in the long-term? Maternal and child health journal. 2014;18(3):714-28. Epub 2013/07/03. doi: 10.1007/s10995-013-1298-6. PubMed PMID: 23812798.

13. Washio Y, Cassey H. Systematic Review of Interventions for Racial/Ethnic-Minority Pregnant Smokers. Journal of smoking cessation. 2016;11(1):12-27. Epub 2016/03/01. doi: 10.1017/jsc.2014.12. PubMed PMID: 26925170; PubMed Central PMCID: PMCPMC4764131.

14. Chamberlain C, O'Mara-Eves A, Porter J, Coleman T, Perlen SM, Thomas J, et al. Psychosocial interventions for supporting women to stop smoking in pregnancy. The Cochrane database of systematic reviews. 2017;2:Cd001055. Epub 2017/02/15. doi: 10.1002/14651858.CD001055.pub5. PubMed PMID: 28196405; PubMed Central PMCID: PMCPMC6472671.

15. Griffiths SE, Parsons J, Naughton F, Fulton EA, Tombor I, Brown KE. Are digital interventions for smoking cessation in pregnancy effective? A systematic review and meta-analysis. Health psychology review. 2018;12(4):333-56. Epub 2018/06/19. doi: 10.1080/17437199.2018.1488602. PubMed PMID: 29912621.

16. Hand D, Ellis J, Carr M, Abatemarco D, Ledgerwood D. Contingency Management Interventions for Tobacco and Other Substance Use Disorders in Pregnancy. Psychology of Addictive Behaviors. 2017;31. doi: 10.1037/adb0000291.

17. Heminger CL, Schindler-Ruwisch JM, Abroms LC. Smoking cessation support for pregnant women: role of mobile technology. Substance abuse and rehabilitation. 2016;7:15-26. Epub 2016/04/26. doi: 10.2147/sar.S84239. PubMed PMID: 27110146; PubMed Central PMCID: PMCPMC4835136.

18. Veisani Y, Jenabi E, Delpisheh A, Khazaei S. Effect of prenatal smoking cessation interventions on birth weight: meta-analysis. The journal of maternal-fetal & neonatal medicine : the official journal of the European Association of Perinatal Medicine, the Federation of Asia and Oceania Perinatal Societies, the International Society of Perinatal Obstet. 2019;32(2):332-8. Epub 2017/09/12. doi: 10.1080/14767058.2017.1378335. PubMed PMID: 28889768.

19. Hubbard G, Gorely T, Ozakinci G, Polson R, Forbat L. A systematic review and narrative summary of family-based smoking cessation interventions to help adults quit smoking. BMC family practice. 2016;17:73. Epub 2016/06/28. doi: 10.1186/s12875-016-0457-4. PubMed PMID: 27342987; PubMed Central PMCID: PMCPMC4921023.

20. Wilson SM, Newins AR, Medenblik AM, Kimbrel NA, Dedert EA, Hicks TA, et al. Contingency Management Versus Psychotherapy for Prenatal Smoking Cessation: A Meta-Analysis of Randomized Controlled Trials. Women's health issues : official publication of the Jacobs Institute of Women's Health. 2018;28(6):514-23. Epub 2018/08/01. doi: 10.1016/j.whi.2018.05.002. PubMed PMID: 30061033; PubMed Central PMCID: PMCPMC6215492.

21. Bain E, Crane M, Tieu J, Han S, Crowther CA, Middleton P. Diet and exercise interventions for preventing gestational diabetes mellitus. The Cochrane database of systematic reviews. 2015;(4):Cd010443. Epub 2015/04/13. doi: 10.1002/14651858.CD010443.pub2. PubMed PMID: 25864059.

22. Brown MJ, Sinclair M, Liddle D, Hill AJ, Madden E, Stockdale J. A systematic review investigating healthy lifestyle interventions incorporating goal setting strategies for preventing excess gestational weight gain. PloS one. 2012;7(7):e39503. Epub 2012/07/14. doi: 10.1371/journal.pone.0039503. PubMed PMID: 22792178; PubMed Central PMCID: PMCPMC3390339.

23. Flynn A, Dalrymple K, Barr S, Poston L, Goff L, Rogozińska E, et al. Dietary interventions in overweight and obese pregnant women: A systematic review of the content, delivery, and outcomes of randomized controlled trials. Nutrition Reviews. 2016;74:312-28. doi: 10.1093/nutrit/nuw005.

24. Gardner B, Wardle J, Poston L, Croker H. Changing diet and physical activity to reduce gestational weight gain: a meta-analysis. Obesity reviews : an official journal of the International Association for the Study of Obesity. 2011;12(7):e602-20. Epub 2011/04/28. doi: 10.1111/j.1467-789X.2011.00884.x. PubMed PMID: 21521451.

25. Girard AW, Olude O. Nutrition education and counselling provided during pregnancy: effects on maternal, neonatal and child health outcomes. Paediatric and perinatal epidemiology. 2012;26 Suppl 1:191-204. Epub 2012/07/07. doi: 10.1111/j.1365-3016.2012.01278.x. PubMed PMID: 22742611.

26. Mohd Yusof BN, Firouzi S, Mohd Shariff Z, Mustafa N, Mohamed Ismail NA, Kamaruddin NA. Weighing the evidence of low glycemic index dietary intervention for the management of gestational diabetes mellitus: an Asian perspective. International journal of food sciences and nutrition. 2014;65(2):144-50. Epub 2014/02/13. doi: 10.3109/09637486.2013.845652. PubMed PMID: 24517860.

27. Muktabhant B, Lawrie TA, Lumbiganon P, Laopaiboon M. Diet or exercise, or both, for preventing excessive weight gain in pregnancy. The Cochrane database of systematic reviews. 2015;(6):Cd007145. Epub 2015/06/13. doi: 10.1002/14651858.CD007145.pub3. PubMed PMID: 26068707.

28. Nascimento SL, Surita FG, Cecatti JG. Physical exercise during pregnancy: a systematic review. Current opinion in obstetrics & gynecology. 2012;24(6):387-94. Epub 2012/09/28. doi: 10.1097/GCO.0b013e328359f131. PubMed PMID: 23014142.

29. O'Brien OA, McCarthy M, Gibney ER, McAuliffe FM. Technology-supported dietary and lifestyle interventions in healthy pregnant women: a systematic review. European journal of clinical nutrition. 2014;68(7):760-6. Epub 2014/05/02. doi: 10.1038/ejcn.2014.59. PubMed PMID: 24781682.

30. Lau Y, Klainin-Yobas P, Htun TP, Wong SN, Tan KL, Ho-Lim ST, et al. Electronic-based lifestyle interventions in overweight or obese perinatal women: a systematic review and meta-analysis. Obesity reviews : an official journal of the International Association for the Study of Obesity. 2017;18(9):1071-87. Epub 2017/05/26. doi: 10.1111/obr.12557. PubMed PMID: 28544551.

31. Shepherd E, Gomersall JC, Tieu J, Han S, Crowther CA, Middleton P. Combined diet and exercise interventions for preventing gestational diabetes mellitus. The Cochrane database of systematic reviews. 2017;11:Cd010443. Epub 2017/11/13. doi: 10.1002/14651858.CD010443.pub3. PubMed PMID: 29129039; PubMed Central PMCID: PMCPMC6485974.

32. Sherifali D, Nerenberg KA, Wilson S, Semeniuk K, Ali MU, Redman LM, et al. The Effectiveness of eHealth Technologies on Weight Management in Pregnant and Postpartum Women: Systematic Review and Meta-Analysis. Journal of medical Internet research. 2017;19(10):e337. Epub 2017/10/17. doi: 10.2196/jmir.8006. PubMed PMID: 29030327; PubMed Central PMCID: PMCPMC5660296.

33. Tieu J, Shepherd E, Middleton P, Crowther CA. Dietary advice interventions in pregnancy for preventing gestational diabetes mellitus. The Cochrane database of systematic reviews. 2017;1:Cd006674. Epub 2017/01/04. doi: 10.1002/14651858.CD006674.pub3. PubMed PMID: 28046205; PubMed Central PMCID: PMCPMC6464792.

34. Currie S, Sinclair M, Murphy MH, Madden E, Dunwoody L, Liddle D. Reducing the decline in physical activity during pregnancy: a systematic review of behaviour change interventions. PloS one. 2013;8(6):e66385. Epub 2013/06/27. doi: 10.1371/journal.pone.0066385. PubMed PMID: 23799096; PubMed Central PMCID: PMCPMC3682976.

35. Chan CWH, Au Yeung E, Law BMH. Effectiveness of Physical Activity Interventions on Pregnancy-Related Outcomes among Pregnant Women: A Systematic Review. Int J Environ Res Public Health. 2019;16(10):1840. doi: 10.3390/ijerph16101840. PubMed PMID: 31126153.

36. Flannery C, Fredrix M, Olander EK, McAuliffe FM, Byrne M, Kearney PM. Effectiveness of physical activity interventions for overweight and obesity during pregnancy: a systematic review of the content of behaviour change interventions. International Journal of Behavioral Nutrition and Physical Activity. 2019;16(1):97. doi: 10.1186/s12966-019-0859-5.
