# Supplementary material for: The effectiveness of smoking cessation, alcohol reduction, diet and physical activity interventions in changing behaviours during pregnancy: A systematic review of systematic reviews
Source: PLoS One. 2020 May 29;15(5):e0232774. doi: 10.1371/journal.pone.0232774 (PMC7259673; doi:10.1371/journal.pone.0232774)
Supplement: S8 Table — (DOCX) [file pone.0232774.s008.docx]

**S8 Table: Smoking behaviour summary of evidence from systematic reviews incorporating meta-analysis data**

| **Behaviour outcome** | **Systematic review author, year** | **Number of studies and sample size of pooled data** | **Result** | **Significance** | **Summary of direction of effect** |
| --- | --- | --- | --- | --- | --- |
| Smoking abstinence (or cessation) during pregnancy | Agboola *et al.* 2010 [1] | 2 studies, n=382 | At 6–9 months follow up  OR 1.23 [0.80, 1.88] | Not significant | Increased |
|  | Agboola *et al.* 2010 [1] | 5 studies, n=772 | At end of pregnancy or longest follow-up before delivery  OR 1.18 [0.85, 1.62] | Not significant | Increased |
|  | Chamberlain *et al.* 2013 [2] | 27 studies, n=11,979 | Counselling interventions compared with usual care at late pregnancy  RR 1.44 [1.19, 1.75] | Significant | Increased |
|  | Chamberlain *et al.* 2017 [3] | 30 studies, n= 12432 | Counselling intervention compared with usual care at late pregnancy  RR 1.44 [1.19-1.73] | Significant | Increased |
|  | Chamberlain *et al.* 2013 [2] | 18 studies, n=9250 | Counselling interventions compared with usual care at late pregnancy – biochemically validated.  RR 1.25 [1.03, 1.50] | Significant | Increased |
|  | Chamberlain *et al*. 2017 [3] | 21 studies, n= 9703 | Counselling interventions compared with usual care at late pregnancy - biochemically validated  RR 1.23 [1.04-1.45] | Significant | Increased |
|  | Chamberlain *et al.* 2013 [2] | 16 studies, n=5,247 | Counselling interventions compared with less intensive intervention at late pregnancy  RR 1.35 [1.00, 1.82] | Significant | Increased |
|  | Chamberlain *et al.* 2017 [3] | 18 studies, n= 5657 | Counselling intervention compared with less intensive intervention at late pregnancy  RR 1.25 [1.07,1.47] | Significant | Increased |
|  | Chamberlain *et al.* 2013 [2] | 12 studies, n=2858 | Counselling interventions compared with less intensive intervention at late pregnancy –biochemically validated  RR 1.46 [1.15, 1.85] | Significant | Increased |
|  | Chamberlain *et al.* 2017 [3] | 15 studies, n= 4919 | Counselling intervention compared with less intensive intervention at late pregnancy - biochemically validated  RR 1.31 (1.10, 1.56) | Significant | Increased |
|  | Chamberlain *et al.* 2013 [2] | 4 studies, n=692 | Counselling interventions compared with less intensive intervention at late pregnancy for spontaneous quitters RR 1.05 [0.98, 1.13] | Not significant | Increased |
|  | Chamberlain *et al.* 2017 [3] | 5 studies, n= 904 | Counselling intervention compared with less intensive intervention, late pregnancy for spontaneous quitters  RR 1.06 [0.99, 1.13] | Not significant | Increased |
|  | Chamberlain *et al.* 2017 [3] | 3 studies, n= 337 | Health education compared with usual care at late pregnancy - biochemically validated  RR 1.45 [0.82, 2.58) | Not significant | Increased |
|  | Chamberlain *et al.* 2017 [3] | 4 studies, n= 1282 | Health education compared with less intensive intervention at late pregnancy  RR 1.20 [0.85, 1.70] | Not significant | Increased |
|  | Chamberlain *et al.* 2017 [3] | 3 studies, n= 1082 | Health education compared with less intensive intervention at late pregnancy - biochemically validated only  RR 1.15 [0.70, 1.91] | Not significant | Increased |
|  | Chamberlain *et al.* 2017 [3] | 3 studies, n= 439 | Feedback compared with less intensive intervention at late pregnancy  RR 1.29 [0.75, 2.20] | Not significant | Increased |
|  | Chamberlain *et al.* 2017 [3] | 3 studies, n= 439 | Feedback compared with less intensive intervention at late pregnancy - biochemically validated  RR 1.29 [0.75, 2.20] | Not significant | Increased |
|  | Chamberlain *et al.* 2017 [3] | 2 studies, n= 355 | Incentives compared with usual care at late pregnancy  RR 4.39 [1.89, 10.21] | Significant | Increased |
|  | Chamberlain *et al.* 2017 [3] | 4 studies, n= 212 | Incentives compared with alternative intervention at late pregnancy  RR 2.36 [1.36, 4.09] | Significant | Increased |
|  | Chamberlain *et al.* 2017 [3] | 7 studies, n= 781 | Social support compared with less intensive intervention at late pregnancy  RR 1.21 [0.93, 1.58] | Not significant | Increased |
|  | Chamberlain *et al.* 2017 [3] | 6 studies, n= 601 | Social support compared with less intensive intervention at late pregnancy biochemically validated  RR 1.42 [0.98, 2.07] | Not significant | Increased |
|  | Chamberlain *et al.* 2017 [3] | 2 studies, n = 258 | Maternal health intervention with smoking cessation component counselling compared with usual care at late pregnancy  RR 0.93 [0.69, 1.25] | Not significant | Decreased |
|  | Chamberlain *et al.* 2017 [3] | 3 studies, n= 455 | Maternal health intervention with smoking cessation component social support compared with less intensive intervention at late pregnancy  RR 1.06 [0.68, 1.63] | Not significant | Increased |
|  | Chamberlain *et al.* 2017 [3] | 2 studies, n= 389 | Maternal health intervention with smoking cessation component social support compared with less intensive intervention at late pregnancy biochemically validated  RR 1.13 [0.72, 1.78] | Not significant | Increased |
|  | Chamberlain *et al.* 2017 [3] | 97 studies, n= 26637 | Interventions for smoking cessation in pregnancy compared with control: sub grouped by main intervention strategy (counselling) at late pregnancy, self-reported and biochemically validated  RR 1.35 [1.23, 1,48] | Significant | Increased |
|  | Filion *et al.* 2011 [4] | 7 studies, n=2528 | At 28-37 weeks gestation  OR 1.17 [0.87–1.57] | Not significant | Increased |
|  | Griffiths *et al.* 2018 [5] | 12 studies, n= 2306 | OR 1.44 [1.04, –2.00] | Significant | Increased |
|  | Hettema *et al.* 2010 [6] | 7 studies, n=2079 | At short term, <6 month follow up  d_c_-0.01 [-0.17, 0.15] | Not significant | Increased |
|  | Hettema *et al.* 2010 [6] | 2 studies, n=744 | At long term >6 month follow up  d_c_  0.15 [-0.19, 0.49] | Not significant | Increased |
|  | Naughton *et al. 2*008 [7] | 12 studies, n=4721 | Comparing self-help with usual care,  OR 1.83 [1.23, 2.73] | Significant | Increased |
|  | Naughton *et al. 2*008 | 7 studies, n=1487 | Comparing self-help and more intensive self-help  OR 1.25 [0.81, 1.94] | Significant | Increased |
|  | Veisani *et al*. 2017 [8] | 3 studies, n=974 | RR 2.47 [1.73, 3.20], end of pregnancy | Significant | Increased |
|  | Wilson *et al.* 2018 [9] | 21 studies, n=6,371 | OR 1.55 [1.19, 2.02], late pregnancy | Significant | Increased |
| Smoking abstinence (or cessation) postpartum following intervention delivered during pregnancy | Chamberlain *et al.* 2017 [3] | 11 studies, n= 2926 | Counselling interventions compared with usual care, 0-5 months post-partum  RR 1.59 [1.26-2.01] | Significant | Increased |
|  | Chamberlain *et al.* 2013 [2] | 6 studies, n=1980 | Counselling interventions compared with less intensive intervention, 0-5 months postpartum  RR 1.17 [0.82, 1.66] | Not significant | Increased |
|  | Chamberlain *et al.* 2017 [3] | 2 studies, n= 150 | Incentives compared with usual care, 0-5 months post-partum  RR 1.09 [0.56, 2.13] | Not significant | Increased |
|  | Chamberlain *et al.* 2017 [3] | 3 studies, n= 195 | Incentives compared with alternative intervention, 0-5 months post-partum  RR 1.79 [0.57, 5.61] | Not significant | Increased |
|  | Chamberlain *et al.* 2017 [3] | 2 studies, n= 237 | Health education compared with usual care, 0-5 months post-partum  RR 3.56 [1.31, 9.67] | Significant | Increased |
|  | Chamberlain *et al.* 2017 [3] | 2 studies, n= 472 | Social support compared with less intensive intervention, 0-5 months post-partum  RR 1.34 [0.35, 5.14] | Not significant | Increased |
|  | Chamberlain *et al.* 2017 [3] | 2 studies, n= 389 | Maternal health intervention with smoking cessation component social support compared with less intensive intervention, 0-5 months post-partum  RR 0.89 [0.51, 1.55] | Not significant | Decreased |
|  | Chamberlain *et al.* 2017 [3] | 6 studies, n= 2458 | Counselling interventions compared with usual care, 6-11 months post-partum  RR 1.33 [1.00-1.77] | Not significant | Increased |
|  | Chamberlain *et al.* 2013 [2] | 3 studies, n=1271 | Counselling interventions compared with less intensive intervention, 6-11 months postpartum  RR 1.08 [0.83, 1.40] | Not significant | Increased |
|  | Chamberlain *et al.* 2017 [3] | 4 studies, n= 1661 | Counselling interventions compared with less intensive intervention, 6-11 months post-partum  RR 1.09 [0.91, 1.31] | Not significant | Increased |
|  | Chamberlain *et al.* 2017 [3] | 3 studies, n=195 | Incentives compared with alternative intervention, 6-11 months post-partum  RR 0.93 [0.85, 1.01] | Not significant | Decreased |
|  | Chamberlain *et al.* 2017 [3] | 3 studies, n= 533 | Social support compared with less intensive intervention, 6-11 months post-partum  RR 1.08 [0.81, 1.44]: | Not significant | Increased |
|  | Chamberlain *et al.* 2017 [3] | 2 studies, n= 431 | Counselling interventions compared with usual care, 12-17 months post-partum  RR 2.20 [1.23-3.96] | Significant | Increased |
|  | Chamberlain *et al.* 2013 [2] | 2 studies, n=1188 | Counselling interventions compared with less intensive intervention, 12-17 months postpartum  RR 1.25 [0.71, 2.20] | Not significant | Increased |
|  | Chamberlain *et al.* 2017 [3] | 3 studies, n= 1578 | Counselling interventions compared with less intensive interventions, 12-17 months post-partum  RR 1.11 [0.87, 1.41] | Not significant | Increased |
|  | Chamberlain *et al.* 2013 [2] | 2 studies, n=934 | Counselling interventions compared with usual care, 18 months or more postpartum  RR 1.25 [0.57, 2.73] | Not significant | Increased |
|  | Chamberlain *et al.* 2017 [3] | 3 studies, n= 798 | Counselling interventions compared with usual care, 18 months or more post-partum  RR 0.98 [0.50-1.92] | Not significant | Decreased |
|  | Filion *et al.* 2011 [4] | 8 studies, n=3290 | At 28 weeks gestation to 6 weeks postpartum  OR 1.08 [0.84, 1.40] | Not significant | Increased |
|  | Wilson *et al.* 2018 [9] | 7 studies, n=1714 | OR 1.47 [0.90, 2.39], early postpartum | Not significant | Increased |
|  | Wilson *et al.* 2018 [9] | 8 studies, n=2360 | OR 1.68 [0.95, 2.98], late postpartum | Not significant | Increased |
| Smoking relapse during pregnancy | Chamberlain *et al.* 2013 [2] | 8 studies, n=688 | Counselling interventions compared with usual care at late pregnancy for spontaneous quitters  RR 1.06 [0.93, 1.21] | Not significant | Increased |
| Smoking reduction  (self- reported – any reductions or reduction greater than 50%) during pregnancy | Chamberlain *et al.* 2013 [2] | 2 studies, n=323 | Counselling interventions compared with usual care at late pregnancy - self-reported various definitions  RR 1.61 [1.06, 2.43] | Significant | Increased |
|  | Chamberlain *et al.* 2017 [3] | 5 studies, n= 839 | Counselling interventions compared with usual care at late pregnancy – self reported various definitions  RR 1.66 [1.27, 2.17] | Significant | Increased |
|  | Chamberlain *et al.* 2017 [3] | 2 studies, n= 1235 | Counselling interventions compared with less intensive intervention smoking reduction at late pregnancy – self reported >50%  RR 1.35 [1.07, 1.71] | Significant | Increased |
| Smoking (cigarettes per day) during pregnancy | Chamberlain *et al.* 2013 [2] | 9 studies, n=3368 | Counselling interventions compared with usual care at late pregnancy - cigarettes per day  SMD -0.25 [-0.46, -0.03] | Significant | Decreased |
|  | Chamberlain *et al.* 2017 [3] | 2 studies, n= 397 | Counselling interventions compared with less intensive intervention at late pregnancy - cigarettes per day  SMD -0.11 [-0.30, 0.09] | Not significant | Decreased |
|  | Chamberlain *et al.* 2017 [3] | 2 studies, n= 687 | Health education compared with usual care at late pregnancy - cigarettes per day  SMD -0.55 [-0.94, -0.15] | Significant | Decreased |
|  | Chamberlain *et al.* 2017 [3] | 2 studies, n= 271 | Maternal health intervention with smoking cessation component (social support) compared with usual care at late pregnancy – cigarettes per day  SMD -0.31 [-0.55, -0.07] | Significant | Decreased |
| Smoking reduction  (biochemically validated reduction) during pregnancy (note: increased direction of effect indicates improved smoking reduction) | Chamberlain *et al.* 2013 [2] | 3 studies, n=1311 | Counselling interventions compared with usual care at late pregnancy - biochemically validated  RR 1.11 [0.54, 2.26] | Not significant | Increased |
|  | Chamberlain *et al.* 2017 [3] | 2 studies, n= 1002 | Counselling interventions compared with usual care at late pregnancy – biochemically validated  RR 0.79 [0.49,-1.28] | Not significant | Decreased |
|  | Chamberlain *et al.* 2017 [3] | 2 studies, n= 857 | Counselling interventions compared with less intensive intervention at late pregnancy – biochemically validated  RR 1.35 [0.98, 1.87] | Not significant | Increased |
| Smoking reduction (mean biochemical cotinine; note: decreased direction of effect indicates improved smoking reduction) | Chamberlain *et al.* 2013 [2] | 3 studies, n=1742 | Counselling interventions compared with usual care at late pregnancy  SMD -0.05 [-0.14, 0.05] | Not significant | Decreased |
|  | Chamberlain *et al.* 2017 [3] | 6 studies, n= 1884 | Counselling interventions compared with usual care at late pregnancy  SMD -0.44 [-0.76, -0.12] | Significant | Decreased |
|  | Chamberlain *et al.* 2017 [3] | 2 studies, n= 102 | Incentives compared with usual care at late pregnancy  MD -2.00 [-6.61, 2.60] | Not significant | Decreased |

Abbreviations: OR, odds ratio; RR, risk ratio; SMD, standardised mean difference; dc, combined effect size; MD, mean difference.

Note: Values in brackets represent 95% confidence interval

**S8 References:**

1. Agboola S, McNeill A, Coleman T, Leonardi Bee J. A systematic review of the effectiveness of smoking relapse prevention interventions for abstinent smokers. Addiction (Abingdon, England). 2010;105(8):1362-80.

2. Chamberlain C, O'Mara-Eves A, Oliver S, Caird JR, Perlen SM, Eades SJ, et al. Psychosocial interventions for supporting women to stop smoking in pregnancy. The Cochrane database of systematic reviews. 2013;(10):Cd001055.

3. Chamberlain C, O'Mara-Eves A, Porter J, Coleman T, Perlen SM, Thomas J, et al. Psychosocial interventions for supporting women to stop smoking in pregnancy. The Cochrane database of systematic reviews. 2017;2:Cd001055.

4. Filion KB, Abenhaim HA, Mottillo S, Joseph L, Gervais A, O'Loughlin J, et al. The effect of smoking cessation counselling in pregnant women: a meta-analysis of randomised controlled trials. BJOG : an international journal of obstetrics and gynaecology. 2011;118(12):1422-8.

5. Griffiths SE, Parsons J, Naughton F, Fulton EA, Tombor I, Brown KE. Are digital interventions for smoking cessation in pregnancy effective? A systematic review and meta-analysis. Health psychology review. 2018;12(4):333-56.

6. Hettema JE, Hendricks PS. Motivational interviewing for smoking cessation: a meta-analytic review. Journal of consulting and clinical psychology. 2010;78(6):868-84.

7. Naughton F, Prevost AT, Sutton S. Self-help smoking cessation interventions in pregnancy: a systematic review and meta-analysis. Addiction (Abingdon, England). 2008;103(4):566-79.

8. Veisani Y, Jenabi E, Delpisheh A, Khazaei S. Effect of prenatal smoking cessation interventions on birth weight: meta-analysis. The journal of maternal-fetal & neonatal medicine : the official journal of the European Association of Perinatal Medicine, the Federation of Asia and Oceania Perinatal Societies, the International Society of Perinatal Obstet. 2019;32(2):332-8.

9. Wilson SM, Newins AR, Medenblik AM, Kimbrel NA, Dedert EA, Hicks TA, et al. Contingency Management Versus Psychotherapy for Prenatal Smoking Cessation: A Meta-Analysis of Randomized Controlled Trials. Women's health issues : official publication of the Jacobs Institute of Women's Health. 2018;28(6):514-23.
