# Supplementary material for: The effectiveness of smoking cessation, alcohol reduction, diet and physical activity interventions in changing behaviours during pregnancy: A systematic review of systematic reviews
Source: PLoS One. 2020 May 29;15(5):e0232774. doi: 10.1371/journal.pone.0232774 (PMC7259673; doi:10.1371/journal.pone.0232774)
Supplement: S9 Table — (DOCX) [file pone.0232774.s009.docx]

**S9 Table: Smoking behaviour summary of evidence from systematic reviews reporting narrative synthesis data**

| **Behaviour outcome** | **Systematic review author, year** | **Number of studies and sample size of pooled data** | **Result** | **Summary findings** |
| --- | --- | --- | --- | --- |
| Smoking abstinence (or cessation) during pregnancy | Agboola *et al.* 2010 [1] | 1 study, n=158 | Short term follow up (pregnancy)  No effect on abstinence at short term follow up (OR 0.83; 95% CI 0.43–1.58) | No difference |
|  | Hand *et al.* 2017 [2] | 14 studies, n= 2827 | From the nine randomised controlled trials, seven found that contingency management produced significantly greater nicotine abstinence than control conditions. All five non-randomised controlled trials tended to show that contingency management was effective to increase quit in pregnancy. | Favour intervention |
|  | Hemsing *et al.* 2012 [3] | 4 studies, sample size not reported | Three out of four studies reported no significant effect of the intervention. One RCT reported a significant difference between intervention and control group women in quit attempts (38% vs 23%; p<0.05) and 7-day abstinence (21 vs 12%; p<0.05) | No difference |
|  | Heminger *et al.* 2016 [4] | 7 studies, n=1108 | Five unique short message service programs and two mobile applications were identified and reviewed. Little evidence was identified to support their use. | No difference |
|  | Hubbard *et al* 2016 [5] | 3 studies, n= 5323 | The three studies show no significant differences by group in pregnant women’s reports of abstinence | No difference |
|  | Kintz *et al.,* 2014 [6] | 24 studies, n= 31958 | Seventy percent of the reviewed studies reported either smoking cessation or a reduction in smoking as a result of participating in a smoking cessation program | Favour intervention |
|  | Su *et al*. 2014 [7] | 2 studies, n=431 | Pharmacological interventions  Significant increase in quit rate in one study, no significant difference in the other. | Inconsistent evidence |
|  | Su *et al*. 2014 [7] | 3 studies, n=355 | Incentives  All studies reported significantly increase in quit rates | Favour intervention |
|  | Washio *et al.* 2016 [8] | 9 studies, n=1690 | End of pregnancy  Five studies showed a significant increase in smoking abstinence during pregnancy, four studies reported no significant difference between intervention and control groups (direction of effect was increased for two studies and equal for two studies) | Inconsistent evidence |
| Smoking relapse during pregnancy | Agboola *et al.* 2010 [1] | 3 studies, n=456 | Medium term follow up (pregnancy)  All studies show no effectiveness at reducing relapse in medium term follow up (OR 0.88; 95% CI 0.49–1.58; OR 0.35; 95% CI 0.18–0.70; OR 3.45; 95% CI 0.95–12.62) | No difference |
|  | Agboola *et al.* 2010 [1] | 1 studies, n=125 | Long term follow up (pregnancy)  No significant effect at long term follow up (OR 0.76; 95% CI 0.36–1.57) | No difference |
| Smoking relapse postpartum | Su *et al*. 2014 [7] | 32 studies =24,595 | None of the intervention types were effective at preventing relapse in the longer-term postpartum period | No difference |
| Smoking abstinence | Washio *et al.* 2016 [8] | 4 studies, n=833 | Postpartum, 6 weeks to 6 months  Three studies reported higher postpartum abstinence in intervention groups, and one study reported no difference between groups. | Favour intervention |
| Smoking reduction | Washio *et al.* 2016 [8] | 1 study, n=145 | One study reported a significant reduction in the number of cigarettes per day in the intervention group | Favour intervention |
| Smoking (biochemical measure, cotinine) | Washio *et al.* 2016 [8] | 2 studies, n=1157 | Two studies reported no significant difference in blood cotinine between intervention and control groups. | No difference |

**S9 References:**

1. Agboola S, McNeill A, Coleman T, Leonardi Bee J. A systematic review of the effectiveness of smoking relapse prevention interventions for abstinent smokers. Addiction (Abingdon, England). 2010;105(8):1362-80.

2. Hand D, Ellis J, Carr M, Abatemarco D, Ledgerwood D. Contingency Management Interventions for Tobacco and Other Substance Use Disorders in Pregnancy. Psychology of Addictive Behaviors. 2017;31.

3. Hemsing N, Greaves L, O'Leary R, Chan K, Okoli C. Partner support for smoking cessation during pregnancy: a systematic review. Nicotine & tobacco research : official journal of the Society for Research on Nicotine and Tobacco. 2012;14(7):767-76.

4. Heminger CL, Schindler-Ruwisch JM, Abroms LC. Smoking cessation support for pregnant women: role of mobile technology. Substance abuse and rehabilitation. 2016;7:15-26.

5. Hubbard G, Gorely T, Ozakinci G, Polson R, Forbat L. A systematic review and narrative summary of family-based smoking cessation interventions to help adults quit smoking. BMC family practice. 2016;17:73.

6. Kintz T, Pryor C, Shemami H, Kridli SA-O. Nursing interventions to promote smoking cessation during pregnancy: An integrative review Journal of Nursing Education and Practice. 2014;4(9).

7. Su A, Buttenheim AM. Maintenance of smoking cessation in the postpartum period: which interventions work best in the long-term? Maternal and child health journal. 2014;18(3):714-28.

8. Washio Y, Cassey H. Systematic Review of Interventions for Racial/Ethnic-Minority Pregnant Smokers. Journal of smoking cessation. 2016;11(1):12-27.
