# Supplementary material for: The effectiveness of smoking cessation, alcohol reduction, diet and physical activity interventions in changing behaviours during pregnancy: A systematic review of systematic reviews
Source: PLoS One. 2020 May 29;15(5):e0232774. doi: 10.1371/journal.pone.0232774 (PMC7259673; doi:10.1371/journal.pone.0232774)
Supplement: S10 Table — (DOCX) [file pone.0232774.s010.docx]

**S10 Table: Alcohol behaviour summary of evidence from systematic reviews reporting narrative synthesis data**

| **Behaviour outcome** | **Systematic review author, year** | **Number of studies and sample size of pooled data** | **Result** | **Summary findings** |
| --- | --- | --- | --- | --- |
| Alcohol consumption | Gilinsky *et al.* 2011 [1] | 8 studies, n=3380 | Six studies report no significant difference in alcohol consumption or quantity of alcohol consumed, whereas two studies reported a significant reduction in the intervention group. | No difference |
|  | Gebara *et al.* 2013 [2] | 8 studies, n=3494 | Seven out of eight brief intervention studies reported reduced alcohol consumption in pregnant women in the intervention group compared with controls | Favours intervention |
|  | Stade *et al.* 2009 [3] | 4 studies, n=715 | Two studies reported mean number of drinks to be reduced in intervention groups, and one reported a reduction in mean number of drinking episodes, although none were significant. One study reported reduced alcohol consumption in the third trimester. | No difference |
| Alcohol abstinence | Gilinsky *et al.* 2011 [1] | 6 studies, n=1151 | Four studies reported a significant increase in abstaining from alcohol consumption among intervention participants, and two studies reported no significant difference between intervention and control groups. However, one study reported that there was a significant increase in women abstaining during pregnancy in both intervention and control groups. | Inconsistent evidence |
|  | Stade *et al.* 2009 [3] | 4 studies, n=715 | All studies reported an increase in abstinence among intervention groups, although only significant in two studies. One study reported significantly increased maintenance of abstinence following intervention among women who were already abstaining at baseline. | Inconsistent evidence |

**S10 References:**

1. Gilinsky A, Swanson V, Power K. Interventions delivered during antenatal care to reduce alcohol consumption during pregnancy: A systematic review. Addiction Research & Theory. 2011;19(3):235-50.

2. Gebara CF, Bhona FM, Ronzani TM, Lourenco LM, Noto AR. Brief intervention and decrease of alcohol consumption among women: a systematic review. Substance abuse treatment, prevention, and policy. 2013;8:31.

3. Stade BC, Bailey C, Dzendoletas D, Sgro M, Dowswell T, Bennett D. Psychological and/or educational interventions for reducing alcohol consumption in pregnant women and women planning pregnancy. The Cochrane database of systematic reviews. 2009;(2):Cd004228.
