# Supplementary material for: The effectiveness of smoking cessation, alcohol reduction, diet and physical activity interventions in changing behaviours during pregnancy: A systematic review of systematic reviews
Source: PLoS One. 2020 May 29;15(5):e0232774. doi: 10.1371/journal.pone.0232774 (PMC7259673; doi:10.1371/journal.pone.0232774)
Supplement: S11 Table — (DOCX) [file pone.0232774.s011.docx]

**S11 Table: Diet behaviour summary of evidence from systematic reviews incorporating meta-analysis data**

| **Behaviour outcome** | **Systematic review author, year** | **Number of studies and sample size of pooled data** | **Result** | **Significance** | **Summary of direction of effect** |
| --- | --- | --- | --- | --- | --- |
| Energy intake (kj/day) | Muktabhant *et al.* 2015 [1] | 14 studies, n=4,065 | MD -570.77 [ -894.28, -247.26] | Significant | Decreased |
| Energy (kcal/day) | Lau *et al.* 2017 [2] | 1 study, n= 597 | Antenatal 15 to 18 weeks via software  MD -48.00 [-118.57, 22.57] | Not significant | Decreased |
|  | Lau *et al.* 2017 [2] | 1 study, n= 128 | Antenatal 15 to 18 weeks via self-report  MD -4.00 [-166.06, 158.06] | Not significant | Decreased |
|  | Lau *et al.* 2017 [2] | 1 study, n= 435 | Antenatal 27 to 28 weeks via software  MD 167.00 [-230.86, -103.14] | Significant | Increased |
|  | Lau *et al.* 2017 [2] | 1 study, n= 128 | Antenatal 27 to 28 weeks via self-report  MD -44.00 [-197.91, 109.91] | Not significant | Decreased |
|  | Lau *et al.* 2017 [2] | 2 studies, n= 30 | Postnatal 12-20 weeks via software  MD -305.28 [-585.88, -24.69] | Significant | Decreased |
|  | Lau *et al.* 2017 [2] | 1 study, n= 3,169 | Postnatal 12-20 weeks via self-report  MD -42.00 [-176.91, 92.91] | Not significant | Decreased |
|  | Lau *et al.* 2017 [2] | 1 study, n= 16 | Postnatal 12 months via software.  MD-495.00 [-956.29, -33.71] | Significant | Decreased |
|  | Lau *et al.* 2017 [2] | 1 study, n=128 | Postnatal 12-months via self-report  MD 41.00 [-165.77, 247.77] | Not significant | Increased |
| Energy (%) | Sherifali *et al.*  2017 [3] | 1 study, n= 43 | MD −0.90 [−3.37, 1.57] | Not significant | Decreased |
| Energy intake from carbohydrate (%) | Sherifali *et al.*  2017 [3] | 1 study, n=43 | MD 1.10 [−4.24, 2.04] | Not significant | Increased |
| Energy intake from protein (%) | Sherifali *et al.* 2017 [3] | 1 study, n=43 | MD 1.40 [0.11 to -2.69] | Not significant | Increased |
| Saturated fat (g day-1) | Lau *et al*. 2017 [2] | 1 study, n=1,145 | Antenatal 15– 18 weeks via software.  MD -1.10 [-2.40, 0.20] | Not significant | Decreased |
|  | Lau *et al.*  2017 [2] | 1 study, n= 261 | Antenatal 15– 18 weeks via self-report.  MD -0.10 [-2.39, 2.19] | Not significant | Decreased |
|  | Lau *et al.*  2017 [2] | 1 study, n=946 | Antenatal 27– 28 weeks via software.  MD -4.40 [-5.63, -3.17] | Significant | Decreased |
|  | Lau *et al.*  2017 [2] | 1 study, n= 256 | Antenatal 27– 28 weeks via self-report.  MD -0.90 [-3.08, 1.28] | Not significant | Decreased |
| Low GI diet | Tieu *et al.*  2017 [4] | 1 study, n=197 | RR 5.37 [1.93, 14.89] | Significant | Increased |
| Fibre | Muktabhant *et al.* 2015 [1] | 8 studies, n= 3,466 | RR 1.53 [0.94, 2.1] | Not significant | Increased |
| Fruit/vegetable (servings/day) | Lau *et al.* 2017 [2] | 14 studies, n=3,169 | Antenatal 30–36 weeks via self-report.  MD 0.27 [0.01, 0.53] | Significant | Increased |
| Taking weight reducing diets | Tieu *et al.* 2017 [4] | 1 study, n=248 | RR 0.95 [0.66, 1.38] | Not significant | Decreased |
| Made dietary changes since the start of intervention | Tieu *et al.* 2017 [4] | 1 study, n=420 | RR 1.12 [0.98, 1.29] | Not significant | Decreased |

Abbreviations: MD, mean difference; RR, risk ratio

Note: Values in brackets represent 95% confidence interval

**S11 References:**

1. Muktabhant B, Lawrie TA, Lumbiganon P, Laopaiboon M. Diet or exercise, or both, for preventing excessive weight gain in pregnancy. The Cochrane database of systematic reviews. 2015;(6):Cd007145.

2. Lau Y, Klainin-Yobas P, Htun TP, Wong SN, Tan KL, Ho-Lim ST, et al. Electronic-based lifestyle interventions in overweight or obese perinatal women: a systematic review and meta-analysis. Obesity reviews : an official journal of the International Association for the Study of Obesity. 2017;18(9):1071-87.

3. Sherifali D, Nerenberg KA, Wilson S, Semeniuk K, Ali MU, Redman LM, et al. The Effectiveness of eHealth Technologies on Weight Management in Pregnant and Postpartum Women: Systematic Review and Meta-Analysis. Journal of medical Internet research. 2017;19(10):e337.

4. Tieu J, Shepherd E, Middleton P, Crowther CA. Dietary advice interventions in pregnancy for preventing gestational diabetes mellitus. The Cochrane database of systematic reviews. 2017;1:Cd006674.
