# Supplementary material for: The effectiveness of smoking cessation, alcohol reduction, diet and physical activity interventions in changing behaviours during pregnancy: A systematic review of systematic reviews
Source: PLoS One. 2020 May 29;15(5):e0232774. doi: 10.1371/journal.pone.0232774 (PMC7259673; doi:10.1371/journal.pone.0232774)
Supplement: S12 Table — (DOCX) [file pone.0232774.s012.docx]

**S12 Table: Diet behaviour summary of evidence from systematic reviews reporting narrative synthesis data**

| **Behaviour outcome** | **Systematic review author, year** | **Number of studies and Sample size of pooled data** | **Result** | **Summary findings** |
| --- | --- | --- | --- | --- |
| Energy | Bain *et al*. 2015 [1] | 5 studies, n= 1,698 | Two studies reported lower total energy intake, while one study reported maintenance of energy intake of intervention group by the end of pregnancy. Two studies reported no clear differences. | Favour intervention |
|  | Flynn *et a*l. 2016 [2] | 2 studies, n= 172 | One study reported no difference while another reported significant difference between intervention and control. | Inconsistent evidence |
|  | Webb-Girard *et al*. 2011 [3] | 5 studies, sample size not reported | Of the five studies reporting energy intakes, only one reported a significant positive impact in the intervention | No difference |
| Carbohydrate or sugar intake | Bain *et al.* 2015 [1] | 5 studies, n= 1,509 | Lower carbohydrate or saccharose intake or avoidance of sugar in three studies. No difference in carbohydrate intake in two studies. | Favour intervention |
|  | Flynn *et a*l. 2016 [2] | 2 studies, n= 246 | One study reported decrease in carbohydrate intake. Another study reported decrease in sugar consumption in the intervention group but no difference in complex carbohydrate intake. | Inconsistent evidence |
|  | O'brien, *et al*. 2014 [4] | 1 study, n=321 | Statistically significant decrease on sugary food intake from baseline | Favour intervention |
| Glycaemic index/ glycaemic load | Flynn *et al.*  2016 [2] | 2 studies, n= 246 | One study reported significant reduction in glycaemic load. Another study reported no change in glycaemic index/ glycaemic load | Inconsistent evidence |
| Low glycaemic index diet | Mohd Yusof *et al.* 2014 [5] | 3 studies, n=209 | Two studies reported that average dietary glycaemic index diet was significantly lower in intervention compared to the control group at end of study. One found no difference in dietary glycaemic index. | Favour intervention |
| Protein intake | Bain *et al.* 2015 [1] | 3 studies, n= 808 | One study reported mean protein intake was significantly higher at two months. Two studies reported no clear differences in protein intake. | No difference |
|  | Flynn *et al.* 2016 [2] | 2 studies, n=172 | Two studies reported significant increase in protein intake in the intervention group. | Favour intervention |
|  | Webb-Girard et al. 2011 [3] | 7 studies, sample size not reported | Four RCTs and three QE studies tested the effect of Nutritional Educational Counselling (NEC) provided alone vs. no NEC on macro- and micronutrient intakes. All but one study observed a positive impact of NEC on protein intakes. | Favour intervention |
| Fat intake | Bain *et al.* 2015 [1] | 5 studies, n= 1568 | Three studies reported lower fat intake or saturated fat intake. Two studies reported no clear differences | Favour intervention |
|  | Flynn *et al.*  2016 [2] | 5 studies, n=2570 | Four studies reported reduced fat or saturated fat intake. One study reported no difference. | Favour intervention |
|  | O'brien *et al*. 2014 [4] | 1 study, n= 321 | Statistically significant decrease on solid fat intake from baseline | Favour intervention |
| Fibre intake | Bain *et al*. 2015 [1] | 2 studies, n=1041 | One study reported no clear difference in fibre intake while another study reported significant increase | Inconsistent evidence |
|  | Flynn *et al*. 2016 [2] | 2 studies, n=2270 | One study reported significant increase, while other reported no significantly different to control. | Inconsistent evidence |
|  | O'brien *et al.* 2014 [4] | 1 study, n= 321 | Statistically significant increase on wholegrain intake from baseline | Favour intervention |
|  | O'brien, *et al.*  2014 [4] | 1 study, n=321 | Statistically significant decrease on refined grain intake from baseline | Favour intervention |
| Fried food intake | O'brien *et al.* 2014 [4] | 1 study, n=321 | Statistically significant decrease from baseline | Favour intervention |
| Fast food intake | O'brien *et al*. 201 [4] | 1 study, n=321 | Statistically significant decrease from baseline | Favour intervention |
| Snack consumption | Flynn *et al*. 2016 [2] | 1 study, n=63 | Study reported increase in number snacks per day | Favour intervention |
|  | Webb-Girard et al. 2011 [3] | 1 study, sample size not reported | Study reported significantly increased snack consumption | Favour intervention |
| Fruit and vegetable intake | Flynn *et al*. 2016 [2] | 4 studies, n= 2387 | Three studies reported significant increase in fruit and vegetable. One study reported significant increase in vegetable consumption. | Favour intervention |
|  | Webb-Girard *et al.* 2011 [3] | 1 study, sample size not reported | Significantly greater proportion of women in the intervention group consumed more vegetables | Favour intervention |
|  | O'brien *et al*. 2014 [4] | 1 study, n= 321 | Statistically significant increase from baseline | Favour intervention |
|  | O'brien *et al*. 2014 [4] | 1 study, n=321 | Statistically significant increase from baseline in avocado and nut intake | Favour intervention |
| Meat intake | O'brien *et al*. 2014 [4] | 1 study, n=321 | Statistically significant decrease on high fat meat intake from baseline | Favour intervention |
|  | Flynn *et al.*  2016 [2] | 1 study, n=63 | No difference in red meat intake | No difference |
|  | Webb-Girard *et al*. 2011 [3] | 2 studies, sample size not reported | Positive deviance approaches to Nutritional Educational Counselling in Egypt and Senegal significantly increased consumption of animal source foods | Favour intervention |
| Fish intake | O'brien *et al*. 2014 [4] | 1 study, n=321 | Statistically significant increase from baseline | Favour intervention |
| Vitamins/minerals intake | Bain *et al.* 2015 [1] | 1 study, n= 401 | No clear difference on iron, vitamin D or folate intake | No difference |
|  | Flynn *et al*. 2016 [2] | 1 study, n=122 | Increase in calcium intake. | Favour intervention |
|  | Webb-Girard *et al*. 2011 [3] | 4 studies, sample size not reported | Three studies conducted in developing countries reported significantly higher dietary iron intakes in the groups receiving Nutritional Educational Counselling (NEC). One study failed to observe a significant effect of NEC on iron intakes. | Favour intervention |
| Diet behaviour | Bain *et al*. 2015 [1] | 3 studies, n=822 | Two studies reported significant improvement in eating habits and behaviour. One study reported no clear difference. | Favour intervention |
|  | Brown *et al.* 2012 [6] | 1 study, n=50 | One study reported women receiving the intervention successfully limited their energy intake and followed dietary instructions for the recommended macro- nutrient composition of the diet. | Favour intervention |
|  | Flynn *et al.*  2016 [2] | 3 studies, n=2630 | Three studies reported improvement in eating habits reported as: Healthy Eating Index, consumption of carbonated “fizzy” drinks and juices and fast foods (frozen and fresh); increased consumption of home-cooked meals and water; or eating habits in general. | Favour intervention |
|  | Gardner et al. 2011 [7] | 5 studies, n= 390 | Five showed no effect on change in diet while one trial showed a positive effect. | No difference |
|  | Shepherd *et al* 2017 [8] | 17 studies, n=5996 | Fifteen studies observed some evidence of benefit(s) in favour of the diet and exercise interventions; while two trials observed no evidence of difference between the diet and exercise interventions and control. | Favour intervention |
|  | Webb-Girard *et al.* 2011 [3] | 4 studies, sample size not reported | Enhanced vs. standard Nutritional Educational Counselling (NEC) and any NEC vs. no NEC were each associated with a significantly higher proportions of women reporting positive dietary changes and/or adherence to an ‘appropriate’ or ‘good’ diet. | Favour intervention |

**S12 References:**

1. Bain E, Crane M, Tieu J, Han S, Crowther CA, Middleton P. Diet and exercise interventions for preventing gestational diabetes mellitus. The Cochrane database of systematic reviews. 2015;(4):Cd010443.

2. Flynn A, Dalrymple K, Barr S, Poston L, Goff L, Rogozińska E, et al. Dietary interventions in overweight and obese pregnant women: A systematic review of the content, delivery, and outcomes of randomized controlled trials. Nutrition Reviews. 2016;74:312-28.

3. Girard AW, Olude O. Nutrition education and counselling provided during pregnancy: effects on maternal, neonatal and child health outcomes. Paediatric and perinatal epidemiology. 2012;26 Suppl 1:191-204.

4. O'Brien OA, McCarthy M, Gibney ER, McAuliffe FM. Technology-supported dietary and lifestyle interventions in healthy pregnant women: a systematic review. European journal of clinical nutrition. 2014;68(7):760-6.

5. Mohd Yusof BN, Firouzi S, Mohd Shariff Z, Mustafa N, Mohamed Ismail NA, Kamaruddin NA. Weighing the evidence of low glycemic index dietary intervention for the management of gestational diabetes mellitus: an Asian perspective. International journal of food sciences and nutrition. 2014;65(2):144-50.

6. Brown MJ, Sinclair M, Liddle D, Hill AJ, Madden E, Stockdale J. A systematic review investigating healthy lifestyle interventions incorporating goal setting strategies for preventing excess gestational weight gain. PloS one. 2012;7(7):e39503.

7. Gardner B, Wardle J, Poston L, Croker H. Changing diet and physical activity to reduce gestational weight gain: a meta-analysis. Obesity reviews : an official journal of the International Association for the Study of Obesity. 2011;12(7):e602-20.

8. Shepherd E, Gomersall JC, Tieu J, Han S, Crowther CA, Middleton P. Combined diet and exercise interventions for preventing gestational diabetes mellitus. The Cochrane database of systematic reviews. 2017;11:Cd010443.
