# Supplementary material for: The effectiveness of smoking cessation, alcohol reduction, diet and physical activity interventions in changing behaviours during pregnancy: A systematic review of systematic reviews
Source: PLoS One. 2020 May 29;15(5):e0232774. doi: 10.1371/journal.pone.0232774 (PMC7259673; doi:10.1371/journal.pone.0232774)
Supplement: S13 Table — (DOCX) [file pone.0232774.s013.docx]

**S13 Table: Physical activity behaviour summary of evidence from systematic reviews incorporating meta-analysis data**

| **Explicit behaviour change outcome (definition/measure)** | **Systematic review author, year** | **Number of studies** | **Result** | **Significance** | **Summary of direction of effect** |
| --- | --- | --- | --- | --- | --- |
| Moderate to vigorous physical activity (min/week) | Lau *et al.* 2017 [1] | 1 study, n=24 | Antenatal (24-26 weeks) via objective measure (Sensewear)  MD:82 [-19.73, 183.72] | Not significant | Increased |
|  | Lau *et al.* 2017 [1] | 1 study, n= 559 | Antenatal (27-28 weeks) via subjective measure (self-report)  MD 10.30 [-12.62, 33.22] | Not significant | Increased |
|  | Lau *et al.* 2017 [1] | 1 study, n= 24 | Antenatal (34-36 weeks) via objective measure (Sensewear)  MD 96.00 [-31.99, 223.99] | Not significant | Increased |
|  | Lau *et al.* 2017 [1] | 1 study, n= 45 | Postnatal 6 weeks via subjective measure (self-report)  MD 152.32 [75.85, 228.79] | Significant | Increased |
|  | Lau *et al.* 2017 [1] | 1 study, n=45 | Postnatal 13 weeks via subjective measure (self-report  MD 83.28 [13.33, 153.23] | Significant | Increased |
|  | Lau *et al.* 2017 [1] | 1 study, n= 153 | Postnatal 12 months via subjective measure (self-report)  MD 90.00 [60.05, 119.95] | Significant | Increased |
|  | Lau *et al* .2017 [1] | 1 study, n= 153 | Postnatal 12 months via objective measure (accelerometer)  MD 12.00 [-8.16, 32,16] | Not significant | Increased |
| MET (minutes/week) | Flannery *et al.* 2019 [2] | 8 studies, n=5,181 | SMD 0.39 [0.14, 0.64] | Significant | Increased |
| Steps (steps/day) | Lau *et al* 2017 [1] | 3 studies, n= 264 | Antenatal 12–28 weeks via pedometer  MD 460.22 [-626.39,1546.8] | Not significant | Increased |
|  | Lau et al. 2017 [1] | 3 studies, n= 1479 | Antenatal 24–30 weeks via self-report  MD 24.51[-7.22, 56.23] | Not significant | Increased |
|  | Lau *et al.* 2017 [1] | 2 studies, n= 62 | Antenatal 32–36 week via self-report  MD 340.58 [-454.05, 1,135.21] | Not significant | Increased |
|  | Lau *et al* 2017 [1] | 1 study, n= 202 | Postnatal 6 weeks via pedometer  MD 734.00 [-700.62, 2,168.62] | Not significant | Increased |
|  | Lau *et al*. 2017 [1] | 1 study, n= 31 | Postnatal 12 months via pedometer  MD 822.00 [-1,272.90, 2,916.90] | Not significant | Increased |
|  | Flannery *et al.* 2019 [2] | 3 studies, n=273 | SMD -0.08 [− 1.01, 0.85] | Not significant | Decreased |
| VO_2_ max | Flannery *et al.* 2019 [2] | 2 studies, n=376 | SMD 0.55 [0.34, 0.75] | Significant | Increased |
| Attending gym at 3 months postnatal | Tieu *et al.* 2017 [3] | 1 study, n=440 | RR 0.98 [0.65, 1.49] | Not significant | Decreased |

Abbreviations: MD, mean difference; RR, risk ratio, SMD, standardised mean difference

Note: Values in brackets represent 95% confidence interval

**S13 References:**

1. Lau Y, Klainin-Yobas P, Htun TP, Wong SN, Tan KL, Ho-Lim ST, et al. Electronic-based lifestyle interventions in overweight or obese perinatal women: a systematic review and meta-analysis. Obesity reviews : an official journal of the International Association for the Study of Obesity. 2017;18(9):1071-87.

2. Flannery C, Fredrix M, Olander EK, McAuliffe FM, Byrne M, Kearney PM. Effectiveness of physical activity interventions for overweight and obesity during pregnancy: a systematic review of the content of behaviour change interventions. International Journal of Behavioral Nutrition and Physical Activity. 2019;16(1):97.

3. Tieu J, Shepherd E, Middleton P, Crowther CA. Dietary advice interventions in pregnancy for preventing gestational diabetes mellitus. The Cochrane database of systematic reviews. 2017;1:Cd006674.
