# Supplementary material for: The effectiveness of smoking cessation, alcohol reduction, diet and physical activity interventions in changing behaviours during pregnancy: A systematic review of systematic reviews
Source: PLoS One. 2020 May 29;15(5):e0232774. doi: 10.1371/journal.pone.0232774 (PMC7259673; doi:10.1371/journal.pone.0232774)
Supplement: S14 Table — (DOCX) [file pone.0232774.s014.docx]

**S14 Table: Physical activity behaviour summary of evidence from systematic reviews reporting narrative synthesis data**

| **Explicit behaviour change outcome (definition/measure)** | **Systematic review author, year** | **Number of studies** | **Result** | **Summary findings** |
| --- | --- | --- | --- | --- |
| Physical activity  (mixed measures) | Bain *et al.*  2015 [1] | 8 studies, n= 2406 | Three studies showed an increase in physical activity index, engagement in leisure time physical activity or daily steps. One study showed not decline in MET. Four studies showed no significant changes in physical activity | Inconsistent evidence |
|  | Currie *et al* 2013 [2] | 10 studies, n= 2047 | Physical activity levels increased more in the intervention groups compared to control groups in 5 studies and decreased less than controls in 3 studies. Two studies demonstrated an undesirable effect where physical activity was higher in the control group at follow-up compared with the intervention group. | Favour intervention |
|  | Nascimento *et al*. 2012 [3] | 1 study, n=190 | Increase in physical activity after 2 months of intervention. | Favour intervention |
|  | Sherifali *et al* 2017 [4] | 1 study, n=43 | The study found no significant differences between intervention and control in light or moderate physical activity as determined by the PPAQ at 32 weeks of gestation (moderate: 95% CI −3.5 to −0.3, *P*=.71; light: 95% CI −2.6 to 0.4, *P*=.08). | No difference |
|  | Chan *et al.* 2019 [5] | 9 studies, n=1,596‬ | Nine studies reported the effect of physical activity interventions on physical activity levels exhibited by pregnant women, or their self-efficacy in increasing physical activity levels. The majority (n = 6; 66.7%) of these studies reported a significantly higher level of physical activity among the intervention participants at postintervention (p ≤ 0.027), or a significantly larger increase in this parameter among intervention participants compared to control counterparts (p = 0.0002). | Favour intervention |
| Behaviour change in diet and exercise | Gardner et al. 2011 [6] | 4 studies, n= 315 | Three trials showed no effect on physical activity one revealed a positive effect on physical activity. | No difference |
|  | Shepherd *et al* 2017 [7] | 7 studies, n= 5996 | Five studies observed some evidence of benefit(s) in favour of the diet and exercise interventions; while two trials observed no evidence of differences between the diet and exercise interventions and control. | Favour intervention |

**S14 References**

1. Bain E, Crane M, Tieu J, Han S, Crowther CA, Middleton P. Diet and exercise interventions for preventing gestational diabetes mellitus. The Cochrane database of systematic reviews. 2015;(4):Cd010443.

2. Currie S, Sinclair M, Murphy MH, Madden E, Dunwoody L, Liddle D. Reducing the decline in physical activity during pregnancy: a systematic review of behaviour change interventions. PloS one. 2013;8(6):e66385.

3. Nascimento SL, Surita FG, Cecatti JG. Physical exercise during pregnancy: a systematic review. Current opinion in obstetrics & gynecology. 2012;24(6):387-94.

4. Sherifali D, Nerenberg KA, Wilson S, Semeniuk K, Ali MU, Redman LM, et al. The Effectiveness of eHealth Technologies on Weight Management in Pregnant and Postpartum Women: Systematic Review and Meta-Analysis. Journal of medical Internet research. 2017;19(10):e337.

5. Chan CWH, Au Yeung E, Law BMH. Effectiveness of Physical Activity Interventions on Pregnancy-Related Outcomes among Pregnant Women: A Systematic Review. Int J Environ Res Public Health. 2019;16(10):1840.

6. Gardner B, Wardle J, Poston L, Croker H. Changing diet and physical activity to reduce gestational weight gain: a meta-analysis. Obesity reviews : an official journal of the International Association for the Study of Obesity. 2011;12(7):e602-20.

7. Shepherd E, Gomersall JC, Tieu J, Han S, Crowther CA, Middleton P. Combined diet and exercise interventions for preventing gestational diabetes mellitus. The Cochrane database of systematic reviews. 2017;11:Cd010443.
