# Supplementary material for: The effectiveness of smoking cessation, alcohol reduction, diet and physical activity interventions in changing behaviours during pregnancy: A systematic review of systematic reviews
Source: PLoS One. 2020 May 29;15(5):e0232774. doi: 10.1371/journal.pone.0232774 (PMC7259673; doi:10.1371/journal.pone.0232774)
Supplement: S15 Table — (DOCX) [file pone.0232774.s015.docx]

**S15 Table: Author reported conflicts of interest**

**S15a: Alcohol reviews**

| Author, year | Author reported conflict of interest |
| --- | --- |
| Gilinsky  *et al.* 2011 [1] | Authors declare: “The authors report no conflicts of interest. The authors alone are responsible for the content and writing of this article.” |
| Gebara  *et al.* 2013 [2] | Authors declare: “that they have no competing interests.” |
| Lui  *et al.* 2008 [3] | Authors declare: “Declarations of interest: None Known” |
| Stade  *et al.* 2009 [4] | Authors declare: “Declaration of interest: None known” |

**S15b: Smoking reviews**

| Author, year | Author reported conflict of interest |
| --- | --- |
| Agboola  *et al.* 2010 [5] | Authors declare: “Within the last 5 years, Tim Coleman has undertaken consultancy work for Pierre Fabre Laboratories, France and also Johnson & Johnson. Both companies produce nicotine replacement therapy” |
| Chamberlain  *et al.* 2013 [6] | No statement made of conflict of interest |
| Filion  *et al.* 2011 [7] | Authors declare: “The authors have no relationships to disclose” |
| Hemsing  *et al.* 2012 [8] | Authors declare: “No competing interests to declare” |
| Hettema  *et al.* 2010 [9] | No statement of conflict of interest reported in the paper |
| Kintz  *et al.* 2014 [10] | No statement of conflict of interest reported in the paper |
| Naughton  *et al.* 2008 [11] | No statement of conflict of interest reported in the paper |
| Su *et al.* 2014 [12] | No statement of conflict of interest reported in the paper |
| Washio  *et al.* 2016 [13] | Authors declare: “The authors have no conflicts of interest to disclose.” |
| Chamberlain *et al.* 2017 [14] | Authors declare: “Catherine Chamberlain is receiving an Australian National Health and Medical Research Council Early Career Fellowship (1088813). She was also awarded an NIHR Cochrane Review Incentive Scheme award (15/81/18) to support the preparation of this updated review. Catherine Chamberlain is also an author on the Cochrane Review entitled ’Pharmacological Interventions to promote smoking cessation in pregnancy“ (Coleman 2015). Alison O’Mara-Eves: none known. Jessie Porter: none known. Tim Coleman has received awards from NIHR, HTA and NIHR paid to his institution. These awards have been used to run research projects. He has also received a single payment from Pierre Fabre Laboratories, France, for speaking at an educational meeting arranged by Pierre Fabre Laboratories (who are manufacturers of nicotine replacement therapy). PFL are a manufacturer of transdermal nicotine patches - the content of the presentation was not vetted and no attempt was made to influence the content of the presentation. Tim Coleman is also an author and contact person for the Cochrane Review entitled ’Pharmacological Interventions to promote smoking cessation in pregnancy” (Coleman 2015). Susan M Perlen: none known. James Thomas: none known. Joanne E McKenzie: none known.” |
| Griffiths *et al.* 2018 [15] | Authors declare: “No potential conflict of interest was reported by the author” |
| Hand *et al.* 2017 [16] | No statement made of conflict of interest. |
| Heminger *et al.* 2016 [17] | Authors declare: “The George Washington University/Dr Lorien C Abroms has licensed the Quit4Baby program to Voxiva, Inc.; Dr Lorien C Abroms has stock options in Voxiva, Inc. The authors report no other conflicts of interest in this work.” |
| Veisani *et al.* 2017 [18] | Authors declare: “No potential conflict of interest was reported by the authors.” |
| Hubbard *et al.* 2016 [19] | Authors declare: “The authors declare that they have no competing interests.” |
| Wilson et al 2018 [20] | Authors declare: “The authors have no conflicts of interest to declare” |

**S15c: Diet and/or physical activity reviews**

| Author, year | Author reported conflict of interest |
| --- | --- |
| Bain  *et al.* 2015 [21] | Authors declare: “Emily Bain: none known. Morven Crane: none known. Joanna Tieu: none known. Caroline Crowther was an investigator on the LIMIT Trial (Dodd 2014). All tasks relating to this study (assessment of eligibility for inclusion, assessment of risk of bias, data extraction) were carried out by other members of the review team who were not directly involved in the trial” |
| Brown  *et al.* 2012 [22] | Authors declare: “that no competing interests exist” |
| Flynn  *et al.* 2016 [23] | Authors declare: “The authors have no relevant interests to declare” |
| Gardner  *et al.* 2011 [24] | Authors declare: “BG, JW and HC declare that they have no conflicts of interest. LP has received payment from ILSI Europe as reimbursement of expenses incurred in attending a workshop on obese pregnancy and long-term outcomes, and was paid as a member of the Tate and Lyle Research Advisory Group from 2007 to 2010, prior to submission of this work.” |
| Webb-Girard *et al.* 2011 [25] | Authors declare: “they have no conflicts of interest” |
| Mohd Yusof  *et al.* 2014 [26] | Authors declare: “no conflicts of interest” |
| Muktabhant  *et al.* 2015 [27] | Authors declare: “Declaration of Interest: None known” |
| Nasciment  *et al.* 2012 [28] | Authors declare: “no conflicts of interest. This article was written without any funding sources” |
| O’Brien  *et al.* 2014 [29] | Authors declare: “no conflict of interest” |
| Lau *et al.* 2017 [30] | Authors declare: “no conflict of interest” |
| Shepherd *et al.* 2017 [31] | Authors declare: “Emily Shepherd: none known. Judith Gomersall: none known. Joanna Tieu has received funding for work outside of the scope of this review- NHMRC postgraduate scholarship, Ken Muirden fellowship (administered by Arthritis Australia; jointly funded by Australian Rheumatology Association and Roche). Shanshan Han: Shanshan Han was an investigator on one of the excluded trials (Crowther 2012). Assessment of eligibility for inclusion was carried out by other members of the review team who were not directly involved in the trial. Caroline Crowther: Caroline Crowther was an investigator on one of the included trials (Dodd 2014), and one of the excluded trials (Crowther 2012). All tasks relating to these trials (assessment of eligibility for inclusion, and if applicable, data extraction and assessment of risk of bias) were carried out by other members of the review team who were not directly involved in the trials. Philippa Middleton: Philippa Middleton was an investigator on one of the excluded trials (Crowther 2012). Assessment of eligibility for inclusion was carried out by other members of the review team who were not directly involved in the trial.” |
| Sherifali *et al.* 2017 [32] | Authors declare: “None declared” |
| Tieu *et al.* 2017 [33] | Authors declare: “Declarations of interest: Joanna Tieu: none known. Emily Shepherd: none known. Philippa Middleton: none known. Caroline A Crowther: none known.” |
| Currie *et al*. 2013 [34] | Authors declare: “The authors have declared that no competing interests exist.” |
| Chan et al 2019 [35] | Authors declare: “The authors declare no conflicts of interest” |
| Flannery et al 2019 [36] | Authors declare: “The authors declare that they have no competing interests” |

**S15 References:**

1. Gilinsky A, Swanson V, Power K. Interventions delivered during antenatal care to reduce alcohol consumption during pregnancy: A systematic review. Addiction Research & Theory. 2011;19(3):235-50.

2. Gebara CF, Bhona FM, Ronzani TM, Lourenco LM, Noto AR. Brief intervention and decrease of alcohol consumption among women: a systematic review. Substance abuse treatment, prevention, and policy. 2013;8:31.

3. Lui S, Terplan M, Smith EJ. Psychosocial interventions for women enrolled in alcohol treatment during pregnancy. The Cochrane database of systematic reviews. 2008;(3):Cd006753.

4. Stade BC, Bailey C, Dzendoletas D, Sgro M, Dowswell T, Bennett D. Psychological and/or educational interventions for reducing alcohol consumption in pregnant women and women planning pregnancy. The Cochrane database of systematic reviews. 2009;(2):Cd004228.

5. Agboola S, McNeill A, Coleman T, Leonardi Bee J. A systematic review of the effectiveness of smoking relapse prevention interventions for abstinent smokers. Addiction (Abingdon, England). 2010;105(8):1362-80.

6. Chamberlain C, O'Mara-Eves A, Oliver S, Caird JR, Perlen SM, Eades SJ, et al. Psychosocial interventions for supporting women to stop smoking in pregnancy. The Cochrane database of systematic reviews. 2013;(10):Cd001055.

7. Filion KB, Abenhaim HA, Mottillo S, Joseph L, Gervais A, O'Loughlin J, et al. The effect of smoking cessation counselling in pregnant women: a meta-analysis of randomised controlled trials. BJOG : an international journal of obstetrics and gynaecology. 2011;118(12):1422-8.

8. Hemsing N, Greaves L, O'Leary R, Chan K, Okoli C. Partner support for smoking cessation during pregnancy: a systematic review. Nicotine & tobacco research : official journal of the Society for Research on Nicotine and Tobacco. 2012;14(7):767-76.

9. Hettema JE, Hendricks PS. Motivational interviewing for smoking cessation: a meta-analytic review. Journal of consulting and clinical psychology. 2010;78(6):868-84.

10. Kintz T, Pryor C, Shemami H, Kridli SA-O. Nursing interventions to promote smoking cessation during pregnancy: An integrative review Journal of Nursing Education and Practice. 2014;4(9).

11. Naughton F, Prevost AT, Sutton S. Self-help smoking cessation interventions in pregnancy: a systematic review and meta-analysis. Addiction (Abingdon, England). 2008;103(4):566-79.

12. Su A, Buttenheim AM. Maintenance of smoking cessation in the postpartum period: which interventions work best in the long-term? Maternal and child health journal. 2014;18(3):714-28.

13. Washio Y, Cassey H. Systematic Review of Interventions for Racial/Ethnic-Minority Pregnant Smokers. Journal of smoking cessation. 2016;11(1):12-27.

14. Chamberlain C, O'Mara-Eves A, Porter J, Coleman T, Perlen SM, Thomas J, et al. Psychosocial interventions for supporting women to stop smoking in pregnancy. The Cochrane database of systematic reviews. 2017;2:Cd001055.

15. Griffiths SE, Parsons J, Naughton F, Fulton EA, Tombor I, Brown KE. Are digital interventions for smoking cessation in pregnancy effective? A systematic review and meta-analysis. Health psychology review. 2018;12(4):333-56.

16. Hand D, Ellis J, Carr M, Abatemarco D, Ledgerwood D. Contingency Management Interventions for Tobacco and Other Substance Use Disorders in Pregnancy. Psychology of Addictive Behaviors. 2017;31.

17. Heminger CL, Schindler-Ruwisch JM, Abroms LC. Smoking cessation support for pregnant women: role of mobile technology. Substance abuse and rehabilitation. 2016;7:15-26.

18. Veisani Y, Jenabi E, Delpisheh A, Khazaei S. Effect of prenatal smoking cessation interventions on birth weight: meta-analysis. The journal of maternal-fetal & neonatal medicine : the official journal of the European Association of Perinatal Medicine, the Federation of Asia and Oceania Perinatal Societies, the International Society of Perinatal Obstet. 2019;32(2):332-8.

19. Hubbard G, Gorely T, Ozakinci G, Polson R, Forbat L. A systematic review and narrative summary of family-based smoking cessation interventions to help adults quit smoking. BMC family practice. 2016;17:73.

20. Wilson SM, Newins AR, Medenblik AM, Kimbrel NA, Dedert EA, Hicks TA, et al. Contingency Management Versus Psychotherapy for Prenatal Smoking Cessation: A Meta-Analysis of Randomized Controlled Trials. Women's health issues : official publication of the Jacobs Institute of Women's Health. 2018;28(6):514-23.

21. Bain E, Crane M, Tieu J, Han S, Crowther CA, Middleton P. Diet and exercise interventions for preventing gestational diabetes mellitus. The Cochrane database of systematic reviews. 2015;(4):Cd010443.

22. Brown MJ, Sinclair M, Liddle D, Hill AJ, Madden E, Stockdale J. A systematic review investigating healthy lifestyle interventions incorporating goal setting strategies for preventing excess gestational weight gain. PloS one. 2012;7(7):e39503.

23. Flynn A, Dalrymple K, Barr S, Poston L, Goff L, Rogozińska E, et al. Dietary interventions in overweight and obese pregnant women: A systematic review of the content, delivery, and outcomes of randomized controlled trials. Nutrition Reviews. 2016;74:312-28.

24. Gardner B, Wardle J, Poston L, Croker H. Changing diet and physical activity to reduce gestational weight gain: a meta-analysis. Obesity reviews : an official journal of the International Association for the Study of Obesity. 2011;12(7):e602-20.

25. Girard AW, Olude O. Nutrition education and counselling provided during pregnancy: effects on maternal, neonatal and child health outcomes. Paediatric and perinatal epidemiology. 2012;26 Suppl 1:191-204.

26. Mohd Yusof BN, Firouzi S, Mohd Shariff Z, Mustafa N, Mohamed Ismail NA, Kamaruddin NA. Weighing the evidence of low glycemic index dietary intervention for the management of gestational diabetes mellitus: an Asian perspective. International journal of food sciences and nutrition. 2014;65(2):144-50.

27. Muktabhant B, Lawrie TA, Lumbiganon P, Laopaiboon M. Diet or exercise, or both, for preventing excessive weight gain in pregnancy. The Cochrane database of systematic reviews. 2015;(6):Cd007145.

28. Nascimento SL, Surita FG, Cecatti JG. Physical exercise during pregnancy: a systematic review. Current opinion in obstetrics & gynecology. 2012;24(6):387-94.

29. O'Brien OA, McCarthy M, Gibney ER, McAuliffe FM. Technology-supported dietary and lifestyle interventions in healthy pregnant women: a systematic review. European journal of clinical nutrition. 2014;68(7):760-6.

30. Lau Y, Klainin-Yobas P, Htun TP, Wong SN, Tan KL, Ho-Lim ST, et al. Electronic-based lifestyle interventions in overweight or obese perinatal women: a systematic review and meta-analysis. Obesity reviews : an official journal of the International Association for the Study of Obesity. 2017;18(9):1071-87.

31. Shepherd E, Gomersall JC, Tieu J, Han S, Crowther CA, Middleton P. Combined diet and exercise interventions for preventing gestational diabetes mellitus. The Cochrane database of systematic reviews. 2017;11:Cd010443.

32. Sherifali D, Nerenberg KA, Wilson S, Semeniuk K, Ali MU, Redman LM, et al. The Effectiveness of eHealth Technologies on Weight Management in Pregnant and Postpartum Women: Systematic Review and Meta-Analysis. Journal of medical Internet research. 2017;19(10):e337.

33. Tieu J, Shepherd E, Middleton P, Crowther CA. Dietary advice interventions in pregnancy for preventing gestational diabetes mellitus. The Cochrane database of systematic reviews. 2017;1:Cd006674.

34. Currie S, Sinclair M, Murphy MH, Madden E, Dunwoody L, Liddle D. Reducing the decline in physical activity during pregnancy: a systematic review of behaviour change interventions. PloS one. 2013;8(6):e66385.

35. Chan CWH, Au Yeung E, Law BMH. Effectiveness of Physical Activity Interventions on Pregnancy-Related Outcomes among Pregnant Women: A Systematic Review. Int J Environ Res Public Health. 2019;16(10):1840.

36. Flannery C, Fredrix M, Olander EK, McAuliffe FM, Byrne M, Kearney PM. Effectiveness of physical activity interventions for overweight and obesity during pregnancy: a systematic review of the content of behaviour change interventions. International Journal of Behavioral Nutrition and Physical Activity. 2019;16(1):97.
